# Dietary Assessment Methods to Estimate (Poly)phenol Intake in Epidemiological Studies: A Systematic Review

**DOI:** 10.1093/advances/nmab017

**Published:** 2021-03-03

**Authors:** Yifan Xu, Melanie Le Sayec, Caroline Roberts, Sabine Hein, Ana Rodriguez-Mateos, Rachel Gibson

**Affiliations:** Department of Nutritional Sciences, School of Life Course Sciences, Faculty of Life Sciences and Medicine, King's College London, London, United Kingdom; Department of Nutritional Sciences, School of Life Course Sciences, Faculty of Life Sciences and Medicine, King's College London, London, United Kingdom; Department of Nutritional Sciences, School of Life Course Sciences, Faculty of Life Sciences and Medicine, King's College London, London, United Kingdom; Department of Nutritional Sciences, School of Life Course Sciences, Faculty of Life Sciences and Medicine, King's College London, London, United Kingdom; School of Psychology and Clinical Language Sciences, University of Reading, Reading, United Kingdom; Department of Nutritional Sciences, School of Life Course Sciences, Faculty of Life Sciences and Medicine, King's College London, London, United Kingdom; Department of Nutritional Sciences, School of Life Course Sciences, Faculty of Life Sciences and Medicine, King's College London, London, United Kingdom

**Keywords:** dietary (poly)phenol, dietary intake, dietary assessment method, epidemiology study, method validation, systematic review

## Abstract

Nutritional epidemiological studies have frequently reported associations between higher (poly)phenol intake and a decrease in the risk or incidence of noncommunicable diseases. However, the assessment methods that have been used to quantify the intakes of these compounds in large-population samples are highly variable. This systematic review aims to characterize the methods used to assess dietary (poly)phenol intake in observational studies, report the validation status of the methods, and give recommendations on method selection and data reporting. Three databases were searched for publications that have used dietary assessment methods to measure (poly)phenol intake and 549 eligible full texts were identified. Food-frequency questionnaires were found to be the most commonly used tool to assess dietary (poly)phenol intake (73%). Published data from peer-reviewed journals were the major source of (poly)phenol content data (25%). An increasing number of studies used open-access databases such as Phenol-Explorer and USDA databases on flavonoid content since their inception, which accounted for 11% and 23% of the data sources, respectively. Only 16% of the studies reported a method that had been validated for measuring the target (poly)phenols. For future research we recommend: *1*) selecting a validated dietary assessment tool according to the target compounds and target period of measurement; *2*) applying and combining comprehensive (poly)phenol content databases such as USDA and Phenol-Explorer; *3*) detailing the methods used to assess (poly)phenol intake, including dietary assessment method, (poly)phenol content data source; *4*) follow the Strengthening the Reporting of Observational Studies in Epidemiology—Nutritional Epidemiology (STROBE-nut) framework; and *5*) complementing dietary intake assessment based on questionnaires with measurement of (poly)phenols in biofluids using appropriate and validated analytical methods.

## Introduction

Diet is one of the most important modifiable factors for the prevention and management of noncommunicable diseases (1, 2). In recent decades, the understanding of diet has evolved from what was believed to be a limited combination of 150 identified nutrients into a much wider range of components including non-nutrients and potentially bioactive compounds such as phytochemicals (3). The development of sensitive and high-resolution analytical methods such as ultra-high-performance liquid chromatography (UPLC) coupled with MS has enabled the rapid identification of these compounds in foods in recent years. There are >26,000 definable biochemicals found in foods and this number is still increasing (4). Consistent evidence has shown plant-based foods such as whole grains (5), fruits, vegetables (6, 7), legumes (8, 9), and nuts (8) to be beneficial for overall health. However, to determine the underlying mechanisms of how and why these heterogenous food groups are beneficial to health we need to fully characterize their differing chemical compounds.

**FIGURE 1:**
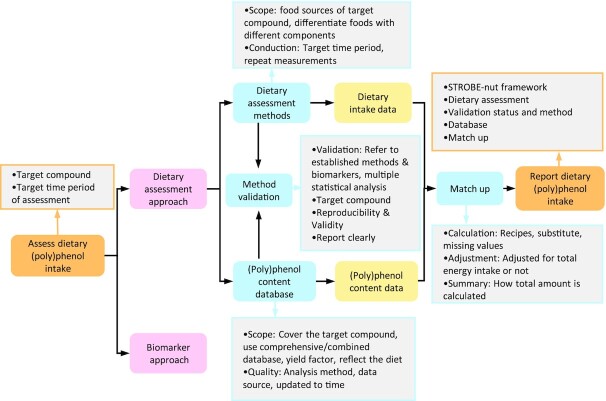
Assessment methods of (poly)phenol intake and key points to notice. Dietary assessment and biomarker are 2 approaches to estimate dietary (poly)phenol intake. In the dietary assessment approach, dietary intakes via food sources of (poly)phenols are estimated by dietary assessment tools such as FFQs, food records, or 24-h recalls. Food content data of (poly)phenols can be obtained from Phenol-Explorer, USDA, some country-based databases, or self-analyzed data. Food intake data are matched with available (poly)phenol content data by individual items and multiplied to calculate (poly)phenol intakes (mg/d). Key points to notice from each step are also listed in the corresponding boxes. FFQ, food-frequency questionnaire; STROBE-nut, Strengthening the Reporting of Observational Studies in Epidemiology—Nutritional Epidemiology.

Nutritional epidemiological studies provide valuable evidence to determine the associations between long-term dietary exposures against a range of health outcomes in free-living populations. The results of these studies are key to identifying dietary components for further testing in nutritional intervention trials (10). Several large prospective studies such as the Nurses’ Health Study (11, 12), the Health Professionals Follow-Up Study (13), and the European Prospective Investigation into Cancer and Nutrition (EPIC) (14) have reported that higher intake of (poly)phenols is associated with a lower risk of cancer and cardiovascular incidence. However, the results of epidemiological studies are based on the assumption that the assessment of the exposure of interest is reliable and accurate. While dietary assessments of various nutrients (macronutrients, fibers, minerals, and vitamins) are well established through routine nutrient database assessment and validated assay methods (15), the assessment of novel bioactives such as (poly)phenols in free-living population groups is still in its infancy (**Figure 1**). Challenges remain in unknown errors from self-reporting, various study designs and tools, unstandardized data coding and processing, and limited sources in food content data.

To better understand the health benefits of (poly)phenols, accurate and reliable methods to measure (poly)phenol intake are required. Given the increasing reporting in nutritional epidemiology of (poly)phenol intake there is an urgent need to understand the strengths and limitations of currently used methods in published studies. Previous systematic reviews investigating the relation between polyphenol intake and health outcomes (16–19) have reported significant heterogeneity across studies reported, which could largely come from the different assessment methods used. To date, no study has described and compared the performance of different tools for estimating (poly)phenol intake.

This systematic review aims to *1*) characterize the observational studies reporting (poly)phenol intake, *2*) report current validation status of the assessment methods of (poly)phenol intake, and *3*) provide recommendations on choosing the right tools and framework on reporting (poly)phenol intake in nutritional epidemiological studies.

## Methods

The methodology applied to this study followed the Preferred Reporting Items for Systematic Reviews and Meta-Analysis (PRISMA) statement (20). Details of the protocol for this systematic review were registered on PROSPERO and can be accessed at https://www.crd.york.ac.uk/prospero/display_record.php?CID=118810.

### Search strategy and study selection

A systematic search was conducted to collect published information on the methods used for assessing dietary polyphenol in health-related observational studies. Three databases—EMBASE, Web of Science, and MEDLINE (obtained from Ovid)—were searched from inception until January 2019. The same strategies were then applied again to include the papers published from the last search until May 2020.

The criteria for inclusion were as follows: *1*) epidemiological observational studies (cross-sectional, cohort, case-control, prospective, or retrospective), *2*) measurement of dietary (poly)phenol intake, *3*) reporting of the distribution of intake or associations between (poly)phenol intake and health-related outcomes, and *4*) having data presented in full texts. Exclusion criteria included *1*) studies conducted in animals or in vitro or *2*) (poly)phenol intake was only measured by urinary or plasma biomarkers.

No restriction on year of publication was applied to the search. Search terms included free texts and subject headings about “Dietary intake” AND “Polyphenol classes and subclasses.” The following filters were applied: English language, human as the subject and Scottish Intercollegiate Guidelines Network filter for observational studies (https://www.sign.ac.uk/search-filters.html). Details of the search terms in this study are shown in **Supplemental Table 1**.

### Screening strategy

Records were screened according to criteria through 3 stages: titles, abstracts, and full texts. The search was conducted by 4 researchers (CR, MLS, SH, and YX). In the first 2 stages, titles and abstracts were reviewed against inclusion and exclusion criteria by 2 groups of researchers (YX with CR, MLS with SH) in parallel. Potentially relevant papers included by both groups were screened in the next stage while inconsistent results were determined together by 2 reviewers from the other group. Full-text reading and information extraction were conducted by 4 reviewers together.

### Quality assessment

The quality of the included papers was assessed by a set of 6 questions adapted from the Strengthening the Reporting of Observational Studies in Epidemiology—Nutritional Epidemiology (STROBE-nut) framework (21). The questions determined study quality over 6 domains: *1*) definition of the target (poly)phenol, *2*) method to obtain and calculate (poly)phenol intake, *3*) dietary assessment methods, *4*) food-composition database, *5*) biomarker measured (if applicable), and *6*) validation of the dietary (poly)phenol assessment method. The papers were rated in the above aspects and overall by “good,” “fair” or “poor” after the full texts were examined by CR, MLS, SH, and YX individually, and the quality rating results were checked by YX. The papers that had a clear and detailed description of the above 6 aspects in the methods section were rated as “good”; papers that reported the above aspects but were lacking some important details were rated as “fair”; and papers that mentioned the above aspects without giving details were rated as “poor.” “NA” was applied to papers when the assessment was not applicable. For example, papers that did not measure biomarker concentrations were not rated “NA” in *5*) biomarker measured and papers that did not validate dietary assessment methods were rated as “NA” in *6*) validation of the dietary assessment method.

### Information extraction and synthesis

An information extraction tool was first developed and tested on pilot data of 3 full texts to refine the tool. Reviewers read the full texts of studies that met the inclusion criteria and retrieved information using a standard database in Microsoft Excel (Microsoft Corporation). The following information was extracted: *1*) first and corresponding author's name; *2*) year of publication; *3*) country or region, study name, study design, and number and characteristics of subjects; *4*) dietary assessment methods (including validation status of the method); *5*) (poly)phenol content database; and *6*) adjustments made in reporting (poly)phenols.

A narrative approach was taken in the synthesis of the results. The included papers from the same study or cohort were grouped. Qualitative analyses were conducted to determine the frequency of different dietary assessment methods and (poly)phenol content databases used in the included papers. For studies that had reported using a validated method to measure (poly)phenol intake, additional information on *1*) reference methods, *2*) statistical analysis method, and *3*) validity of the method was extracted. For papers reporting both dietary intake and biomarker concentrations, the analytical methods and correlations between the 2 measurements were also extracted.

## Results

**FIGURE 2:**
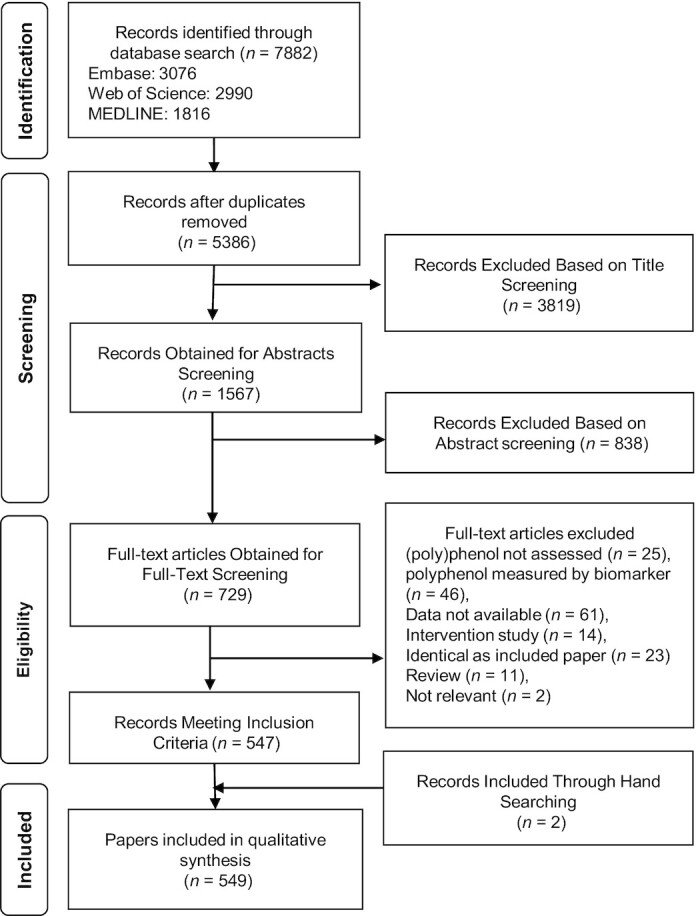
Flow chart of study selection process.

The study selection process of the systematic review is presented in **Figure 2**. Among a total of 7882 records obtained from searching, 5386 unique records were screened for titles and 1567 were screened for abstracts. Then, 729 full texts were examined further and 182 papers were excluded for the following reasons: no (poly)phenol assessment conducted (*n* = 25), (poly)phenol assessment based only on biomarkers (*n* = 46), data not available as a full text (*n* = 61), intervention conducted (*n* = 14), identical as included paper (*n* = 23), review (*n* = 11) and not relevant (*n* = 2). Two papers were included through hand-search. In the end, 549 papers were included in the qualitative synthesis of data. Characteristics of the included studies are shown in **Supplemental Table 2**. Quality of the included papers based on the 6 questions was as follows: 33% were ranked good, 60.5% were fair, and 6.5% were poor (**Table 1**).

**TABLE 1:** Quality rating of the included papers^1^

Quality aspects	Good, *n* (%)	Fair, *n* (%)	Poor, *n* (%)	Not applicable, *n* (%)
Clearly define research question	366 (66.55)	168 (30.55)	15 (2.73)	0 (0.00)
Method description	233 (42.36)	254 (46.18)	62 (11.27)	0 (0.00)
Dietary assessment	234 (42.55)	273 (49.64)	42 (7.64)	0 (0.00)
Food-composition database	200 (36.36)	282 (51.27)	67 (12.18)	0 (0.00)
Biomarker applied	42 (7.64)	14 (2.55)	1 (0.18)	492 (89.45)
Validation of the method	152 (27.64)	214 (38.91)	51 (9.27)	132 (24.00)
Overall quality rating	180 (32.73)	333 (60.55)	36 (6.55)	0 (0.00)

1 *n* = 549. “*n*” indicates the number of papers rated in each grade.

### Dietary assessment methods

**FIGURE 3:**
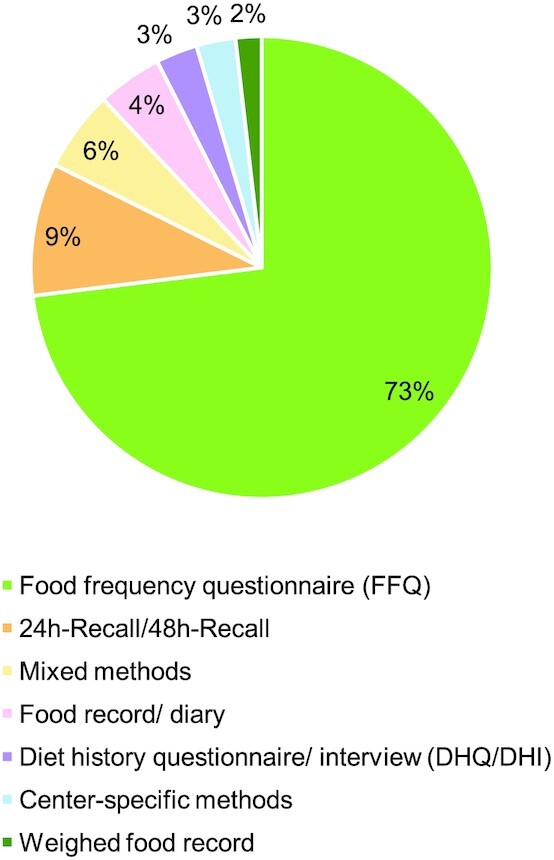
Percentage of dietary assessment methods to measure (poly)phenol intake in the published papers.

To identify the dietary assessment tools used to measure the intake of (poly)phenol food sources, the frequency of different tools used in the 549 included papers was calculated. As shown in **Figure 3**, an FFQ was the most widely used (73%, *n* = 401) dietary assessment tool, followed by the 24-h or 48-h dietary recall (9%, *n* = 51). The number of items measured in the FFQs varied widely, from <10 items in a specific food group (i.e., soy food such as soft and firm tofu, tofu products, soy milk, bean curd products, and soy beans were measured to assess isoflavone intake) (22–31) to >200 detailed food items (i.e., fruits, vegetables, legumes, grains, oils, dairy, fish, eggs, beverages, and commercially processed products) (32–34). In addition, the time period measured by FFQs varied extensively from the past week (35, 36) to the past 20 y (37–41). There were very few (3.5%, *n* = 14) studies using FFQs that were designed to estimate (poly)phenol intake (42–55), whereas the majority of papers used FFQs aimed to estimate food intake or energy and nutrient intake, such as EPIC FFQs (56–59), Block FFQ (60), and Willett FFQ (61). Estimated food diaries or records were reported in 4.6% (*n* = 25) of the included papers, whereas diet history questionnaires/interviews accounted for 3% (*n* = 16) and weighed food records accounted for 2% (*n* = 10). From the studies reviewed, 5.6% (*n* = 31) reported using a combination of different types of tools to measure dietary intake. This was mainly for the purpose of validating the FFQ on measuring (poly)phenol intake (42, 44–46, 50, 62–71). In 15 studies (2.7%) that pooled data from different population samples, such as the EPIC study (72–86), tools that are specific to different research centers were used.

### Food content databases

**FIGURE 4:**
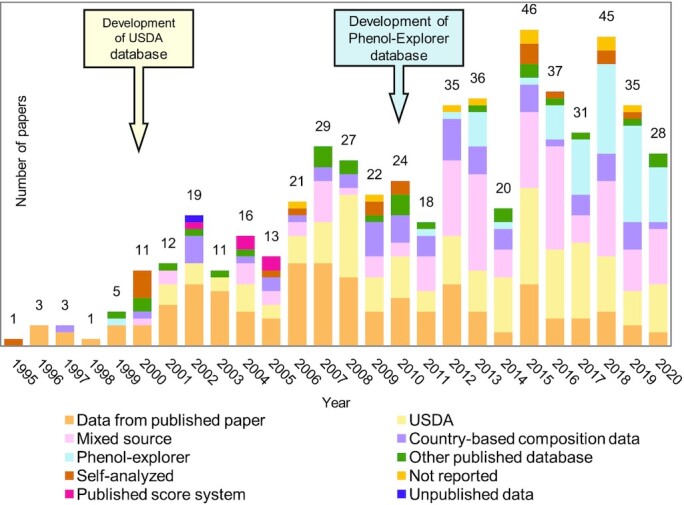
Sources of (poly)phenol content data in the published papers. The number of papers in the area have increased during recent years, especially after the development of USDA databases in the 2000s and the development of Phenol-Explorer database in the 2010s.

**Figure 4** presents the number of papers published over time and sources of food content databases. It is apparent that there is an increasing number of papers published over the years, with a rapid increase after the development of the USDA database of Flavonoids Content in Food in the 2000s and Phenol-Explorer in the 2010s. Overall, Phenol-Explorer and USDA databases were used in 11% (*n* = 59) and 23% (*n* = 125) of the studies we reviewed, respectively. The number of studies using these 2 databases is increasing. In 2019–2020, the percentages of studies reportedly using Phenol-Explorer and USDA databases were 35% (*n* = 22) and 19% (*n* = 12), respectively. It needs to be noted that in this current study we did not specify a different sub-database from USDA such as the USDA Database for the Flavonoid Content of Selected Foods (87), the USDA Database for the Isoflavone Content of Selected Foods (88), and the USDA Database for the Proanthocyanidin Content of Selected Foods (89). In addition, one-quarter of the studies (*n* = 138) we reviewed used (poly)phenol content data previously published in peer-reviewed journals, whereas 3% (*n* = 18) of studies directly analyzed the (poly)phenol content of food in their studies (46, 90–106). Country-based food content databases were used in 10% (*n* = 56) of the studies, mainly from China (32%, *n* = 18) (23, 25, 107–121), Japan (29%, *n* = 16) (30, 122–135), and Singapore (*n* = 9, 16%) (136–144). A mixed source of databases was used in 20% (*n* = 111) of the papers.

### Validation of the method

Of the 549 papers included, 417 (76%) papers reported using validated dietary assessment tools. However, only 86 (16%) reported the validity or reproducibility of the tool to estimate (poly)phenol intake, which referred to 46 validation papers (42, 44, 45, 48–50, 52, 62–64, 66, 68, 69, 90, 94, 114, 145–174). The remaining papers referred to validated methods for nutrients or food intake and not (poly)phenol intake. Details of the (poly)phenol validation papers are shown in **Table 2**. Among all the dietary assessment methods, FFQs were the most frequently reported validated tools (*n* = 39, 85%) (42, 44, 45, 48–50, 52, 62–64, 66, 68, 69, 114, 145–147, 149, 151–153, 155–157, 159–161, 163–174). Other validated dietary collection tools included dietary records or diary (*n* = 7, 15%) (42, 44, 62, 68, 90, 150, 158), 24-h or 48-h recalls (*n* = 6, 13%) (44, 45, 66, 69, 148, 154), brief diet history questionnaire (*n* = 1, 2%) (94), and dietary history interviews (*n* = 1, 2%) (162). These methods were used to measure intakes of isoflavones (*n* = 19, 41%) (48, 49, 52, 63, 64, 68, 69, 145, 148, 149, 153, 155, 160, 161, 166, 167, 170, 171, 174), flavonoids (*n* = 13, 28%) (42, 46, 63, 114, 146, 147, 156, 162–165, 169, 172, 174, 175), lignans (*n* = 3, 7%) (147, 154, 173), phytoestrogens (both isoflavones and lignans) (*n* = 6, 13%) (45, 66, 150, 151, 157, 175), and total (poly)phenols (*n* = 6, 13%) (44, 62, 94, 158, 159, 168), whereas 1 paper (2%) reported validation of tea flavonoids (50).

**TABLE 2:** Validation of the assessment tools for estimating (poly)phenol intake^1^

		Dietary assessment methods		Reference		Number of participants		Validated polyphenol(s)
First author (ref)	Year		Reproducibility	Dietary	Biomarker		Statistical methods	
Chun (148)	2009	24-h recall	−		Urine-spot	2908	Partial correlations after adjusting for sex, age, ethnicity, BMI, income level, alcohol consumption, and cigarette smoking	Isoflavones
Kilkkinen (154)	2003	24-h recall	+	DR, S, 48-h recall, S	Serum-fasting	48-h recall: 233/3DD: 334	Attenuation regression coefficients	Lignans
Cao (90)	2010	7DD	−		Plasma-fasting	92	Correlation coefficients	Flavonoids
Grace (150)	2004	7DD	−	DR, S	Serum-spot + urine spot	248/333	Pearson's correlation coefficients on log-transformed data	Phytoestrogens: isoflavones and lignans
Taguchi (158)	2017	7DD	−	FFQ		37	Correlation coefficients	Total polyphenols
Taguchi (94)	2018	BDHQ	−	DR, S		37	Spearman's correlation coefficients	Total polyphenols
Jarvinen (162)	1993	DHI	+^2^			121	Interclass correlation coefficients	Flavonoids
Budhathoki (145)	2011	FFQ	+	DR, M		28	Pearson's correlation coefficients	Isoflavones
Butchart (146)	2011	FFQ	+	WR, S		83	Spearman's rank correlation coefficients	Flavonoids
Chavez-Suarez (147)	2017	FFQ	+^2^			50	Adjusted correlation coefficients	Flavonoids and lignans
Cuervo (159)	2014	FFQ	−			38	NR	Polyphenols
Frankenfeld (48)	2002	2 FFQs	−		Plasma-fasting	77	Pearson's correlation coefficients	Isoflavones
Frankenfeld (49)	2003	2 FFQs	+		Plasma-fasting	96	Pearson's correlation coefficients	Isoflavones
Fraser (149)	2016	FFQ	+	24-h recall, M	Urine-spot	Urine: 909; questionnaires: 96,116	Deattenuated correlations	Isoflavones
Hankin (160)	2001	FFQ	−	24-h recall, M		858	Correlation coefficients	Isoflavones
Hernandez-Ramirez (151)	2009	FFQ	+^2^			50	Energy adjusted (by means of energy residuals) intraclass correlation coefficients	Phytoestrogens: isoflavone and lignans
Ishihara (63)	2009	FFQ, 4DD, Arizona tea questionnaires	+	DR, M		55	Spearman's rank correlation coefficients	Isoflavones
Iwasaki (153)	2009	FFQ	−	DR, M		215	Spearman's correlation coefficients	Isoflavones
Kurahashi (155)	2009	FFQ	+	DR, S		NR	Spearman's rank correlation coefficients	Isoflavones
Kyle (163)	2002	FFQ	−	WR, S		41 men and 40 women	Energy-adjusted and Spearman rank correlation coefficients, cross-classification	Flavonols, procyanidins, flavon-3-ols, flavanones and flavones
Li (156)	2013	FFQ	+	24-h recall, M		121	Correlation coefficients	Flavonoids and stilbenes
Lin (41)	2013	FFQ	−		Serum-fasting	135	Correlation coefficients adjusted for energy intake	Lignans
Luo (157)	2015	FFQ	−	24-h recall, M		70	Spearman's correlation coefficients	Phytoestrogens: isoflavone and lignans
Yue (164)	2020	FFQ	+	DR, M		641 men and 724 women	Spearman's rank correlation coefficients adjusted and non-adjusted for total energy intake, variance captured by top food contributors	Total flavonoids and subclasses
Pietinen (165)	1988	FFQ	+	DR, M		133 men for validity/190 men for reliability	Pearson's correlation coefficients between log-transformed, energy-adjusted intake values	Total flavonoids and subclasses
Sasaki (166)	2003	FFQ	+			209	Spearman's correlation coefficients	Isoflavones
Segovia-Siapco (167)	2016	FFQ	−	24-h recall, M		55	Pearson's bivariate correlation, cross-classification quartiles, Bland-Altman plots	Soy isoflavones
Shahar (168)	2014	FFQ	−	24-h recall, M		93	Spearman correlation and intraclass correlation, Bland-Altman plot, cross-classification and Cohen's κ	Total polyphenols
Thompson (169)	2008	FFQ	−	24-h recall, M		2053	Deattenuated correlation coefficients	Flavonoids
Tsubono (170)	2003	FFQ	+	DR, M		201	Spearman's correlation coefficients	Isoflavones
Wu (174)	2004	FFQ	−		Plasma-spot	194	ANOVA and ANCOVA between quartiles	Isoflavones
Yamamoto (64)	2001	FFQ	+	WR, S	Serum-fasting + urine-24 h	215	Spearman's correlation coefficients, energy-adjusted correlation coefficients	Isoflavones
Yao (114)	2019	FFQ	+	DR, NR		NR	Spearman's rank correlation coefficients	Quercetin, myricetin
Yokoyama (171)	2016	FFQ	−	WR, M		142	Spearman's correlation coefficients	Isoflavones: daidzein and genistein
Zhang (172)	2009	FFQ	+	DR, M		61	Pearson's correlation coefficients, cross-classification, Bland-Altman plots	Flavonoids
Heald (152)	2006	FFQ	−	WR, S	Serum-spot	Serum: 203	Spearman's correlation coefficients and Pearson's correlation coefficients/energy-adjusted and Spearman's rank correlation coefficient, cross-classification	Serum: phytoestrogen; weighed diet records: flavonoids
Tseng (52)	2008	SFQ, FFQ (DAF)	−	DAF (FFQ)	Urine-24 h/overnight urine-spot, multiple	Questionnaire: 451/urine: 27	Spearman's correlations	Isoflavones: daidzein, genistein, glycitein, ODMA, equol
Bhakta (66)	2005	FFQ, 24-h recall	−	24-h recall, M		133	Cross-classification, energy adjusted and unadjusted Spearman's correlation coefficients, method of triads	Phytoestrogens: isoflavone and lignans
French (45)	2007	FFQ, 48-h recall	−		Urine-24 h	26	Spearman rank correlation coefficients, cross-classification (κ, tertile)	Phytoestrogens: isoflavone and lignans
Huang (69)	2000	FFQ, 48-h recall	−	48-h recall, M	Urine-24 h	61	Spearman's correlations	Isoflavones: daidzein and genistein
Hoge (62)	2019	FFQ, 3DD	−	DR, S	Urine-spot	53	Pearson's correlation, cross-classification by median, Cohen κ coefficient, method of triads	Total polyphenols
Somerset (42)	2014	FFQ and 3DD	+	DR, S		60	Spearman's rank correlations, Bland-Altman plots	Flavonoids
Ishihara (161)	2003	FFQ	+	DR, S		392	Spearman's correlation coefficients	Genistein
Hakim (50)	2001	FFQ, 4DD, Arizona tea questionnaires	+	FFQ, S, DR, S		120	Pearson and Spearman correlation coefficients; precision was examined using intraclass correlation coefficients between the log-transformed (natural log) estimates of black tea polyphenols for the 2 tea questionnaires (ATQ1 and ATQ2)	Total tea polyphenols
Verkasalo (68)	2001	FFQ, 7DD	−	DR, S	Plasma-spot	80	Spearman's correlation coefficients	Isoflavones: daidzein and genistein
Vian (44)	2015	FFQ, 3DD, 24-h recall	+	24-h recall, M + DR, S	Urine-spot	120	Method of triads, Pearson's correlation coefficients, intraclass correlation (κ) and Bland-Altman plots, classification by quartiles of consumption	Total polyphenols

1 ATQ, The Arizina Tea Questionnaire, BDHQ, brief diet history questionnaire; DAF, Harvard Diet Assessment Form; DHI, dietary history interview; DR, dietary records; FFQ, food-frequency questionnaire; M, multiple conduction; NR, not reported; ODMA, O-Desmethylangolensin; , ref, reference; S, single conduction; SFQ soy food questionnaire; WR, weighed records; 3DD, 3-d food diary (records); 7DD, 7-d food diary (records); +, reproducibility was evaluated; −, reproducibility was not evaluated.

2 Only reproducibility of the tools was evaluated in the study.

To determine the validity of the dietary assessment tool, 34 (74%) studies used other dietary assessment methods as references, including multiple (*n* = 7, 21%) (145, 154, 161, 164, 165, 172, 176) or single (*n* = 11, 32%) (42, 44, 62, 68, 94, 150, 154, 155, 161, 166, 170) measurement(s) of dietary records, multiple (*n* = 1, 3%) (171) or single (*n* = 4, 12%) (64, 146, 163, 175) weighed food records, multiple 24-h (*n* = 9, 26%) (44, 66, 149, 156, 157, 160, 167–169) or 48-h (*n* = 1, 3%) recalls (69), or other FFQs (*n* = 3, 9%) (50, 158, 168). Meanwhile, 17 (37%) studies compared dietary assessment methods against (poly)phenol biomarkers, from 24-h urine (*n* = 4, 24%) (45, 52, 69, 170), spot urine (*n* = 6, 35%) (44, 52, 62, 148–150), fasting plasma/serum (*n* = 7, 41%) (48, 49, 66, 90, 154, 170, 173), or nonfasting spot plasma/serum (*n* = 4, 24%) (68, 150, 174, 175). The statistical methods reported in the validations were Spearman's or Pearson's correlation coefficients (*n* = 41, 89%), cross-classification (*n* = 9, 20%) (44, 45, 62, 66, 163, 167, 168, 172, 175), Bland-Altman plots (*n* = 5, 11%) (42, 44, 167, 168, 172), method of triads (*n* = 3, 7%) (44, 62, 66), and ANOVA between different concentrations (*n* = 1, 2%) (174). Validation by sole correlations was reported in 36 out of 46 studies (78%) (48–50, 52, 63, 64, 68, 69, 90, 94, 114, 145, 146, 148–151, 154–158, 160–162, 164–166, 169–171, 173, 176, 177).

### Statistical adjustment in reporting (poly)phenol intake

A total of 197 (36%) papers reported adjusted values of (poly)phenol intake, mostly adjusted by total energy intake (*n* = 188, 95%) using the residual method or nutrient density described by Willett and Stampfer (178). Other factors adjusted for included age (*n* = 12, 6%) (82, 179–189), season (*n* = 6, 3%) (82, 180–184), gender (*n* = 4, 2%) (185, 187–189), ethnicity (*n* = 1, 0.5%) (186), and income (*n* = 1, 0.5%) (186).

### Analysis of (poly)phenol metabolites in biofluids

Among the 549 papers assessing dietary (poly)phenol intake using dietary assessment tools, 57 (10%) papers also reported concentrations of (poly)phenols in biofluids at the same time. The correlations between biomarkers and dietary assessment methods were reported in 43 (75%) studies (44–49, 52, 62, 64–69, 71, 82, 86, 90, 111, 116, 123, 134, 147–150, 152, 154, 190–204). In these studies, correlation coefficients ranged from 0.12 to 0.71 in urine, from 0.06 to 0.80 in plasma, and from 0.08 to 0.43 in serum (Supplemental Table 2). In a few studies among the above, dietary (poly)phenols measured by food records or recalls were found to correlate better with biomarker concentrations than FFQs (44, 45, 62, 67, 68). In addition, plasma or urinary isoflavones showed higher correlation coefficients with dietary intake than lignans (45, 66, 71).

## Discussion

The creditability of nutritional epidemiological research relies on the use of valid and reliable tools to measure dietary exposures. To our knowledge, this is the first systematic review that has characterized and critically evaluated the methods used to measure dietary (poly)phenol intake in epidemiological studies.

A multistage process is used for the estimation of dietary (poly)phenol intake in nutritional epidemiological studies as detailed in Figure 1. Dietary assessment requires the recording of food and beverage intake by participants; however, the method of collection differs in the level of detail. Different dietary assessment tools, such as FFQs, food diaries, and 24-h recalls, vary in their ability to capture the food sources of dietary (poly)phenols according to their design and method of validity (**Table 3**). In this study we found that FFQs are the most popular dietary assessment tools used to measure food sources of (poly)phenol intake. This is likely due to the low burden of the method towards participants and researchers alike, and their ability to measure long-term exposure to dietary factors (205). However, compared with dietary recall and records, FFQs have limited ability to cover the wide range of food sources of (poly)phenols and differentiate the food items due to the predefined list of food groups covered in the questionnaire. Moreover, the structure and food groups included in FFQs can differ between studies depending on the research questions. For example, if an FFQ is used to measure total and subclasses of flavonoid intake, important sources of flavonoids should be covered in the list such as tea, fruits and vegetables, soy products, legumes and beans, cocoa products, and red wine (206). At the same time, each FFQ item should cover only 1 type of food that has a different (poly)phenol content profile, and all items should be listed separately (207). In many FFQs the potential to measure subclasses of polyphenols is hampered by combining of items in FFQ categories—for example, red and white wine (67) and apples and pears (12, 67). Unlike FFQs consisting of a predefined list of food groups and frequencies of intake, dietary recalls or food records are not restricted and allow matching of individual food items with (poly)phenol content data. However, repeat measurements are needed to enable the dietary data to represent the time period of estimation, especially for 24-h dietary recalls (208). For example, 24-h recalls should be repeated 3 times during a 7-d period, including 2 weekdays and 1 weekend day, to represent habitual dietary intake (134, 199, 211). Food records should be conducted in different seasons to be able to represent yearly intake (46, 212). In this review we found that ∼15% of the studies used 24-h/48-h recalls or food diaries to measure food sources of (poly)phenols, which is much lower compared with studies using FFQs. This may result in a higher burden on participants and researchers when using dietary recalls or records (209). Clear instructions on completion and photos of portion sizes (45, 47, 213–215) are recommended to support the participants, while standardized coding protocols and trained coders are needed to interpret the questionnaires in high quality consistently (209). The strengths and limitations of different methods in measuring (poly)phenol intakes are listed in **Table 4**.

**TABLE 3:** Comparison of different methods for assessing dietary (poly)phenol intake

Dietary assessment tools	Characteristics	Strengths	Limitations	Ability to capture food sources of (poly)phenols
Food-frequency questionnaires (FFQs)	Finite food items (10–200+) targeting focused food groups or general diet; able to assess long-term intake (3 mon to 5 y)	Easy to conduct, low burden to participants and researchers; suitable to measure long-term intake (205)	Less able to capture day-to-day variability in diet; lack of specificity when foods were grouped together; prone to misreport and memory bias	Ability depends on the number of food items measured and whether foods with different (poly)phenol contents were distinguished; able to capture intake of nondaily or weekly consumed foods
24-h/48-h Recall	Recall of food intake in last 24 h or 48 h; usually conducted at multiple different time points during a longer period to capture habitual diet	Easy to conduct; not restricted to a predefined list of foods (208)	High participant burden if conducted multiple times; prone to misreport and memory bias; not able to reflect interday variability if only conducted once	More specificity as (poly)phenol content can be linked to individual foods rather than food groups; repeat measurement will increase the ability to capture infrequently consumed foods
Estimated food diary or food records	Record of intake for 3 d, 1 wk, 1 mo, etc.; usually assisted with photos of portion sizes	Able to capture day-to-day variabilities; not limited to a predefined list of foods; repeat measurement will increase the ability to capture infrequently consumed foods (209)	High participant burden; prone to coding error (standard protocol and training is needed for coding); prone to error from misreport	More specificity as (poly)phenol content can be linked to individual foods rather than food groups; able to capture intake of less common foods
Weighed food records (3 d, 7 d)	Weigh and record the portion of every food intake for a consecutive period of time	Accurate in portion size and less memory bias; not restricted to a predefined list of foods; repeat measurement will increase the ability to capture infrequently consumed foods	High participant burden (need weighing tools and instructions); high researcher burden (standard protocol and training is needed for coding)	Able to capture (poly)phenol intakes from less common foods; repeat measurement will increase the ability to capture infrequently consumed foods
Duplicate diet	A duplicated portion of foods consumed is retained, weighed, and chemically analyzed; often referred to as gold standard (210)	Accurate in portion size; not restricted to a predefined list of foods; able to measure dietary intake of food components not available in databases	High participant burden (to collect the food duplicates and preserve of each meal); high researcher burden (standard protocol and training is needed for weighing and coding); expertise and resources for chemical analysis are needed	Able to measure the (poly)phenol intake more precisely than using database; need accurate analytical methods to measure target (poly)phenol content in foods
Diet history questionnaire/interview	Structured questionnaire/interview on food intake frequencies during a specific period with open-ended questions and cross-checked with specific amounts	Not restricted to a predefined list of foods; suitable to measure long-term intake/intake during a specific period	High researcher burden (standard protocol and training is needed for the interview and coding); prone to misreport and memory bias	Able to capture (poly)phenol intake from less common food and infrequently consumed foods

**TABLE 4:** Challenges and recommendations in dietary assessment of (poly)phenol intakes^1^

Challenges	Recommendations/resources needed
Dietary assessment tool not designed to capture (poly)phenol diet sources and variabilities	*1*) Choose a tool that covers the food sources of target compounds, and has foods with different (poly)phenol profiles differentiated*2*) Consider the frequency and timing of measurement to make sure the target time period is represented
	*3*) Use multiple measurements of dietary records rather than FFQs if possible
Dietary assessment methods not validated/insufficiently validated to measure (poly)phenol intakes	*1*) Validate the tool specifically for measuring the intake of target (poly)phenols*2*) Use other well-established dietary assessments and established biomarkers as reference methods*3*) Conduct multiple statistical analysis to reflect validation status: correlation coefficients, cross-classification (Cohen's κ), Bland-Altman
	*4*) Provide evidence of validity and reproducibility
Limited data on (poly)phenol content in foods	*1*) Choose a database that covers the content data of all food sources of the target compound; combine different sources of data to make up the limitations of single databases
	*2*) Choose databases of high quality: with reliable analytical methods and data source, and consistent data between multiple sources; use data from comparable analytical methods if need to summarize the total
	*3*) Choose the data that can match up with the food item in the measured diet, in terms of food origin and species; apply food-processing yield factors if applicable
	*4*) Check the updates of the database and search for newly published data if possible
	*5*) Use standard recipes that can reflect the diet in target population
Insufficient reporting on methods	*1*) Follow STROBE-nut framework (21)
	*2*) Describe the dietary assessment methods used in detail: food groups and number of items measured, whether similar foods are distinguished in items; how the assessment was conducted, time range measured, and validation of the methods
	*3*) Report clearly whether the dietary assessment method is validated for targeted (poly)phenols; if it is validated, describe the reference method used including sample size and characteristics of the population, how the reference method was conducted, statistical analysis methods used and validity/reproducibility results; if biomarkers are used to validate the dietary assessment, report details of the biomarkers and analytical methods applied
	*4*) Report the name of the database used or cite the reference paper; describe the analytical method used to get the food content data and whether compounds were measured individually or in aglycones; report the retention factors used
	*5*) Report how food items were matched, how missing items and missing compound values were analyzed, and the adjustment made on the intake amount

1 FFQ, food frequency questionnaire; STROBE-nut, Strengthening the Reporting of Observational Studies in Epidemiology—Nutritional Epidemiology.

In terms of (poly)phenol content data source, we found that open-access databases are becoming the most widely used resources for estimating (poly)phenol content of foods in the studies we reviewed. The development of the USDA databases in 1999 (216) and Phenol-Explorer in 2010 (217) has led to a growing number of researchers using these comprehensive databases in their studies over the last 20 y. Many papers combined different sources of (poly)phenol data to serve the purpose and scope of the individual studies. For example, many studies applied both USDA and Phenol-Explorer databases to cover the wide range of food items measured in the dietary assessment. Meanwhile, some other studies combined data from domestic databases to match up with the diet of the local population, such as Chinese food (218–221), Korean food (222–225), and UK food (81, 182, 226, 227). Data from published papers are also commonly applied to cover the food sources of (poly)phenols that do not appear in the databases. A systematic review that included 157 studies published between 2004 and 2014 reporting food-composition tools for (poly)phenol intake assessment (228) found that 60% of studies used published accessible databases (including USDA, Phenol-Explorer, country-based databases, and other public databases according to the groupings in the current study), and 33% of the literature applied >1 database. The result is in accordance with our findings, where 49% of studies used publicly accessible databases and 20% of studies used >1 data source of (poly)phenol contents. The Phenol-Explorer database and USDA database are the 2 most comprehensive databases on (poly)phenol content in foods. The Phenol-Explorer database retrieves all classes and subclasses of (poly)phenol content data in foods published in scientific papers, books, and reviews and includes critical evaluations of experiment details on sampling, (poly)phenol extraction, and analytical methods (217). Mean values of each (poly)phenol content are provided in different categories of analytical methods used such as chromatography, chromatography after hydrolysis, and the Folin assay method (217). In addition, retention factors of compounds after food processing are also available (229). The USDA database for flavonoid content is mainly focused on a specific number of flavonoids compounds, which are retrieved from published papers and evaluated for quality using a standardized procedure and scoring system developed by the Nutrient Data Laboratory of the USDA (87). Flavonoid content data from the United States and other countries are included in the database. Only the data generated by acceptable analytical methods that can result in good separation of the target compounds, such as HPLC, capillary zone electrophoresis, and micellar electrokinetic capillary chromatography, are included (87). Different from Phenol-Explorer, which shows content data from different methods separately, the USDA content data are measured as glucosides and converted into aglycones to be comparable and consistent across the database. These 2 databases are free to access for the public, include data with relative acceptable analytical methods, and integrate different sources to provide reliable (poly)phenol content data.

The current available databases have limitations that may hinder the accuracy of (poly)phenol measurement. First, many foods and compounds are missing from the databases due to the lack of analytical data, which would lead to underestimation of the dietary intake of less-studied compounds and foods. In both Phenol-Explorer and USDA databases, frequently, content data of only a small number of phenolic compounds are available for a food item. Therefore, underestimation of intake can occur when calculating total (poly)phenol intake by summarizing the intakes of individual classes and subclasses of compounds. Second, the analytical methods that have been used to measure (poly)phenols in food are not consistent in accuracy. Some of the food content data are only available from spectrophotometric methods such as the Folin-Ciocalteu method (230). The Folin method is a colorimetric method measuring levels of total antioxidant capacity rather than total phenolics (231). Data from these spectrophotometric methods are highly inaccurate compared with the content data from analytical methods that can quantify the compounds individually, such as HPLC. In addition, many (poly)phenols are quantified with standards of their parent compounds (e.g., quantify resveratrol glucosides with resveratrol) or similar compounds (e.g., quantify tyrosol with hydroxytyrosol) (232). Even though this is common practice, especially when authentic standards are not commercially available, quantifying compounds with other standards can lead to inaccurate results (233). In addition, the content data may not be reliable if they are derived from a small number of studies due to interlaboratory variability. Furthermore, the databases are usually updated after long periods; therefore, there is a time lag between newly published values and database update. Last, the information can lack details on the multiple factors influencing polyphenol content of food such as origin, species, storage, and processing procedures. Similar to nutrients, the food contents of phytochemicals can be highly variable under the influence of the above factors (207). Domestic data may be more accurate than using data from other countries; however, there are limited compounds in country-based databases (234, 235) because of the huge expense and difficulties in analysis. Phenol-Explorer has been updated on yield factors related to cooking in recent years (229); however, the data available are still limited. Although more data and improvements in data quality are needed, the establishment of these databases is a very useful step towards more accurate analysis.

While many studies used a validated tool to measure nutrient intake, most of them were not validated for the target (poly)phenols. This limitation may introduce an unknown amount of systematic error in the estimation. The validity of measuring (poly)phenol intake could vary from the validity of measuring other nutrients or foods, especially considering the challenges in dietary assessment tools and food content databases mentioned previously. In addition to the low number of validated studies, we found the quality of the validation studies to be low, with 50% of the studies ranked as “fair” and 13% as “poor.” We identified the following concerns: *1*) most of the validation evidence was provided only by correlation coefficients with estimations derived from other dietary assessment methods, *2*) no evidence of reproducibility was provided in most studies, and *3*) the validation study design and results were insufficiently reported. The poor validation and reporting of (poly)phenol assessment restrict the evaluation of the existing evidence in meta-analysis.

The last data extraction of this study was conducted in May 2020. At the time of writing the manuscript, further papers reporting dietary assessment of (poly)phenol intakes have been published (236–239). In agreement with our findings, most of the papers (236–238) used FFQs to estimate (poly)phenol intake. Phenol-Explorer (236, 239) and USDA databases (236–238) were used as (poly)phenol composition data sources. Yue et al. (236) reported moderate to high validity (Spearman's rank correlation coefficients were 0.4–0.7 or ≥0.7) and high reproducibility (rank interclass correlation coefficients were ∼0.8) of an FFQ on reporting flavonoids compared with two 7-d weighed dietary records with both Phenol-Explorer database and a Harvard database that was mainly based on the USDA database.

Outside the remit of this review, it is important to mention that another approach to estimate (poly)phenol intake in epidemiological studies is the use of biomarkers of (poly)phenol intake in biofluids. This approach is considered to be more objective as it directly reflects “bioavailable” (poly)phenol exposure levels and does not depend on self-reported data and inaccuracies of tools and databases. The dietary assessment polyphenol database method is simple and easy to conduct, although it is prone to errors resulting from misreport (240) and limited information in the current databases (241). Biomarkers of (poly)phenol intake can be used to validate or calibrate the dietary assessment approach. Therefore, the integration of (poly)phenol biomarkers into the dietary assessment can provide a more robust result, especially when linking (poly)phenol intakes to health outcomes (242). However, the biomarker method requires access to specialized analytical techniques such as LC and MS, which are less accessible compared with dietary assessment. The accuracy of the analytical methods depends largely on the availability of authentic chemical standards, and validation of the methods is also needed. In addition, the short half-life of many (poly)phenol metabolites could hamper their potentials to represent habitual diet (242). Despite the fast development in this field, there are very few validated, efficient, and accessible methods that are available for use in epidemiology studies (210). In this study we found a limited number of studies (*n* = 57, 10%) that reported both dietary intake and biomarker concentrations of (poly)phenols and, of these, only 43 (75%) reported the correlation coefficients between the 2 measurements. The correlation coefficients varied widely between different samples, compounds, and analytical methods used to measure biomarkers and dietary assessment methods. Interestingly, better correlations between dietary intake of (poly)phenols and (poly)phenol biomarkers were found between food diaries or recalls than FFQs in a few studies (44, 45, 62, 67, 68), which indicated the advantage of food records or recalls. In future studies that measure dietary intake of (poly)phenols, measurement of biomarkers should be taken into consideration. Also, more efforts are needed in the development of analytical methods that are validated for measuring (poly)phenol biomarkers and, at the same time, are suitable (fast, high-throughput) to use in large epidemiological studies.

There has been an exponential increase in nutritional epidemiology studies reporting associations between (poly)phenol intake and health outcomes (17–19). However, it remains a challenge to be able to advise the public on the likely intake level that is beneficial to health due to the existence of methodological issues in measuring (poly)phenol intake identified in this review, including limited ability and validity of the dietary assessment tools, limited food content data of (poly)phenols, and insufficient reporting of the results (Table 4). To strengthen the quality of evidence on (poly)phenol intake and health, our recommendations on choosing dietary assessment methods are summarized in Table 4. The first step is to describe clearly the scope of the estimation and have a target compound or a group of (poly)phenols and define a target time period of measurement according to the research question. When choosing the dietary assessment tool, careful consideration should be given to select the one that can cover the food sources of the target compounds and represent the diet in the target time range. The dietary assessment tool should be validated for the target compounds with the use of other, more robust dietary assessment tools or ideally provide correlations with biomarkers of (poly)phenol intake. If possible, the use of multiple measurements of dietary records to collect dietary intake data is recommended. The chosen food content database of (poly)phenols should cover the content data of food sources of the target compounds. The combination of USDA and Phenol-Explorer databases is the most comprehensive approach at the moment. The use of domestic databases and recipes to match with the diet of the population if available is also recommended. The reporting of observational studies estimating (poly)phenol intake should follow the STROBE-nut framework (21), including additional details that are specific to (poly)phenol analysis as described in Table 4.

In summary, the findings of this systematic review suggest that further research is needed to develop tools that are specifically designed to measure (poly)phenol intake. Improvements in current food content databases are also essential to provide more reliable, detailed, and up-to-date data. International collaborations on setting up standards and guidance on food content analysis regarding phytochemical compounds are also needed. Validation of the tools, especially combining the biomarker or metabolomics approach to validate or calibrate the dietary assessment methods, could provide more reliable evidence on relations between (poly)phenol intake and health outcomes. Future research should complement the dietary intake data with quantification of biomarkers of (poly)phenol intake. Therefore, development of fast, high-throughput, sensitive, and accurate analytical methods to measure concentrations of phenolic metabolites in biofluids is also needed. Understanding the different methods of measurement and their strengths and limitations, as set out in this review, is an important step towards developing a standardized approach to measurement and reporting dietary (poly)phenol intake. This will enable comparison between studies and future pooling of results in systematic reviews to strengthen the evidence base.

## Supplementary Material

nmab017_Supplemental_Files

## Notes

YX is supported by a King's-China Scholarship (K-CSC).

Author disclosures: The authors report no conflicts of interest.

Supplemental Tables 1 and 2 are available from the “Supplementary data” link in the online posting of the article and from the same link in the online table of contents at https://academic.oup.com/advances/.

Abbreviations used: EPIC, European Prospective Investigation into Cancer and Nutrition; FFQ, food-frequency questionnaire; STROBE-nut, Strengthening the Reporting of Observational Studies in Epidemiology—Nutritional Epidemiology; UPLC, ultra-high-performance liquid chromatography.
