# Supplementary material for: Dietary Assessment Methods to Estimate (Poly)phenol Intake in Epidemiological Studies: A Systematic Review
Source: Adv Nutr. 2021 Mar 3;12(5):1781–801. doi: 10.1093/advances/nmab017 (PMC8483972; doi:10.1093/advances/nmab017)
Supplement: nmab017_Supplemental_Files [file nmab017_supplemental_files.zip › SR1-supplementary table 1-20201219-review3.0.docx]

**Supplementary Table 1: Search strategy (Embase)**

| **No** | **Search terms** |
| --- | --- |
| 1 | exp diet/ |
| 2 | diet.ti,ab. |
| 3 | diets.ti,ab. |
| 4 | dietary.ti,ab. |
| 5 | exp dietary exposure/ |
| 6 | exp dietary supplements/ |
| 7 | exp dietary intake/ |
| 8 | intake*.ab,ti. |
| 9 | exposure.ab,ti. |
| 10 | exposing.ti,ab. |
| 11 | consume.ti,ab. |
| 12 | consumpt*.ti,ab. |
| 13 | supplement*.ab,ti. |
| 14 | exp nutrition supplement/ |
| 15 | 1 or 2 or 3 or 4 or 5 or 6 or 7 or 8 or 9 or 10 or 11 or 12 or 13 or 14 |
| 16 | exp POLYPHENOLS/ |
| 17 | exp FLAVONOIDS/ |
| 18 | exp LIGNANS/ |
| 19 | exp STILBENES/ |
| 20 | exp polyphenol derivative/ |
| 21 | polyphenol*.ab,ti. |
| 22 | (poly adj2 phenol).ab,ti. |
| 23 | flavon*.ab,ti. |
| 24 | flavan*.ab,ti. |
| 25 | ellagitannin*.ab,ti. |
| 26 | phenolic acid*.ab,ti. |
| 27 | coumarin*.ab,ti. |
| 28 | furanocoumarin*.ab,ti. |
| 29 | anthocyan*.ab,ti. |
| 30 | proanthocyan*.ab,ti. |
| 31 | isoflavon*.ab,ti. |
| 32 | (phyto adj2 estrogen*).ab,ti. |
| 33 | phytoestrogen*.ab,ti. |
| 34 | catechin*.ab,ti. |
| 35 | epicatechin*.ab,ti. |
| 36 | ellagic acid.ab,ti. |
| 37 | gallic acid.ab,ti. |
| 38 | caffeic acid.ab,ti. |
| 39 | resveratrol*.ab,ti. |
| 40 | quercetin*.ab,ti. |
| 41 | myrcetin*.ab,ti. |
| 42 | kaempferol*.ab,ti. |
| 43 | apigenin*.ab,ti. |
| 44 | narigenin*.ab,ti. |
| 45 | cyanindin*.ab,ti. |
| 46 | genistein*.ab,ti. |
| 47 | tannin.ab,ti. |
| 48 | tannins.ab,ti. |
| 49 | tannic.ab,ti. |
| 50 | 16 or 17 or 18 or 19 or 20 or 21 or 22 or 23 or 24 or 25 or 26 or 27 or 28 or 29 or 30 or 31 or 32 or 33 or 34 or 35 or 36 or 37 or 38 or 39 or 40 or 41 or 42 or 43 or 44 or 45 or 46 or 47 or 48 or 49 |
| 51 | Clinical study/ |
| 52 | Case control study/ |
| 53 | Family study/ |
| 54 | Longitudinal study/ |
| 55 | Retrospective study/ |
| 56 | Prospective study/ |
| 57 | Randomized controlled trials/ |
| 58 | 56 not 57 |
| 59 | Cohort analysis/ |
| 60 | (Cohort adj (study or studies)).mp. |
| 61 | (Case control adj (study or studies)).tw. |
| 62 | (follow up adj (study or studies)).tw. |
| 63 | (observational adj (study or studies)).tw. |
| 64 | (epidemiologic$ adj (study or studies)).tw. |
| 65 | (cross sectional adj (study or studies)).tw. |
| 66 | 51 or 52 or 53 or 54 or 55 or 58 or 59 or 60 or 61 or 62 or 63 or 64 or 65 |
| 67 | 15 and 50 and 66 |
| 68 | limit 67 to human |
| 69 | limit 68 to english language |
