# Supplementary material for: Dietary Assessment Methods to Estimate (Poly)phenol Intake in Epidemiological Studies: A Systematic Review
Source: Adv Nutr. 2021 Mar 3;12(5):1781–801. doi: 10.1093/advances/nmab017 (PMC8483972; doi:10.1093/advances/nmab017)
Supplement: nmab017_Supplemental_Files [file nmab017_supplemental_files.zip › SR1-supplementary table 2-20201229-review3.0.docx]

**Supplementary Table 2: Summary of methods to assess dietary (poly)phenol intakes (N=549 papers)**

| **Study, year (supplemental refs)** | **Study/Cohort name** | **Country/region** | **Population** | **Dietary assessment methods*** | **(Poly)phenol content database** | **(Poly)phenol measured** | **Validation of (poly)phenol measurement** | **Adjustment in reporting** | **Biomarker measured and the agreement with dietary intake** |
| --- | --- | --- | --- | --- | --- | --- | --- | --- | --- |
| **24h-recalls (p1-8)** | | | | | | | | | |
| Hervert-Hernandez et al., 2012(1) |  | Mexico | 139 subjects, women 100%, age 25-45y | 24h recall (measured period last 24h, repeated 3 24h food recalls from a weekend day and two non-consecutive weekdays were recorded in a month at intervals of 10d, not validated) | self-analysed | total polyphenols | N | not adjusted | NR |
| Kilkkinen et al., 2003(2) |  | Finland | 10000 subjects, NR, age 25-64y | 24h recall (measured period last 24h, repeated once, validated) | published | lignans: matairesinol, secoisolariciresinl | Y | adjusted for energy intake | serum: enterolactone, lignan density(ug/MJ) vs. serum EL concentration in men/women (r=0.19/ 0.16) |
| Otaki et al., 2009(3) |  | Japan | 516 subjects, women 100%, age mean 58y | 24h recall (measured period last 24h, repeated once, not validated) | Japanese food composition tables+ Functional Food Factor database | flavonoids: flavanols, isoflavones, flavonols, flavones, flavanones | N | not adjusted | NR |
| Song et al., 2008(4) |  | Korea | 34 subjects, women 100%, age 20-26y | 24h recall (measured period last 24h, repeated every 4 months, not validated) | Korean Nutrient Database | isoflavones: daidzein, genistein, glycitein | N | not adjusted | NR |
| Lefevre-Arbogast et al., 2018(5) | 3C study | france | 9294 subjects, men 38%, age 65+y | 24h recall (measured period last 24h, repeated once, not validated) | Phenol-Explorer | flavonoids, phenolic acids, stilbenes, lignans, other polyphenols | N | not adjusted | NR |
| Rizzi et al., 2016(6) | ATHENA project | Italy | 443 subjects, men 40%, age 20-85y | 24h recall (measured period last 24h, repeated at least 3 times per year, not validated) | USDA+ Phenol-Explorer+ BDA-IEO | anthocyanins and toal polyphenols | N | not adjusted | NR |
| Zamora-Ros et al., 2012(7) | EPIC | Europe | 36037 subjects, men 36%, age 35–74y | 24h recall (measured period last 24h, repeated once, not validated) | mixed source | phytoestrogens: isoflavones, lignans, enterolignans, coumestans and equol | N | adjusted for age and weighed by season and day of recall | NR |
| Zamora-Ros et al., 2013(8) | EPIC | Europe | 35628 subjects, men 40%, age 35–74y | 24h recall (measured period last 24h, repeated once, not validated) | USDA+ Phenol-Explorer+ UK Food Standards Agency FCDB on isoflavones | flavonoids: anthocyanidins, flavanols, proanthocyanidins and, flavonols, flavones, flavanones and isoflavones | N | adjusted for age and weighed by season and day of recall | NR |
| Zamora-Ros et al., 2013(9) | EPIC | Europe | 36037 subjects, men 36%, age 35-74y | 24h recall (measured period last 24h, repeated once, not validated) | USDA | thearubigins | N | adjusted for age and weighed by season and day of recall | NR |
| Zamora-Ros et al., 2013(10) | EPIC | Europe | 477312 subjects, men 30%, age 35–70 y | 24h recall (measured period NR, repeated once, not validated) | Phenol-Explorer | phenolic acids: hydroxycinnamic acids, hydroxyphenylacetic acids, hydroxyphenylpropanoic acids | N | adjusted for age and weighed by season and day of recall | NR |
| Zamora-Ros et al., 2016(11) | EPIC | Europe | 36037 subjects, men 36%, age 35-74y | 24h recall (measured period last 24h, repeated once, not validated) | Phenol-Explorer | all classes: all subclasses | N | adjusted for age and weighed by season and day of recall | NR |
| Adriouch et al., 2018(12) | French NutriNet-Santé cohort | France | 84158 subjects, men 21%, age mean 44.1y | 24h recall (measured period last 24h, repeated 3 times, validated) | Phenol-Explorer | all classes: all subclasses | N | not adjusted | NR |
| Fleury et al., 2017(13) | French NutriNet-Santé cohort | France | 1766 subjects, men 17%, age 18-64y | 24h recall (measured period last 24h, repeated 3 times, validated) | published | isoflavones, lignans, coumestans: genistein and biochanin A, daidzein, formononetin and glycitein, matairesinol and secoisolaricirecinol, coumestrol | N | not adjusted | NR |
| Kuczmarski et al., 2018(14) | HANLS study | USA | 3418 subjects, men 45%, age 30-64y | 24h recall (measured period last 24h, repeated once, not validated) | USDA | flavonoids: anthocyanidins flavan-3-ols flavanones flavones flavonols isoflavone | N | adjusted for energy, race (asappropriate), income, and age. | NR |
| Nascimento‑Souza et al., 2018(15) | Health conditions, nutrition and use of medication by the elderly in Viçosa (Minas Gerais) | Brazil | 620 subjects, men 47%, age 60-98y | 24h recall (measured period last 24h, repeated once, validated) | Phenol-Explorer | all classes: 14 classes and 23 subclasses | N | adjusted for energy intake | NR |
| Miranda et al., 2016(16) | Health Survey of Sao Paulo (ISA-Capital) | Brazil | 550 subjects, men 46%, age 12+y | 24h recall (measured period last 24h, repeated twice, validated) | Phenol-Explorer | all classes: 502 individual polyphenols | N | not adjusted | NR |
| Miranda et al., 2016(17) | Health Survey of Sao Paulo (ISA-Capital) | Brazil | 1103 subjects, men 46%, age 20+y | 24h recall (348 items, measured period last 24h, conducted twice, validated) | Phenol-Explorer | all classes: all subclasses | N | not adjusted | NR |
| Miranda et al., 2017(18) | Health Survey of Sao Paulo (ISA-Capital) | Brazil | 557 subjects, men 46%, age 12+y | 24h recall (measured period last 24h, repeated twice, validated) | Phenol-Explorer | phenolic acids: hydroxycinnamic acids, alkylmethoxyphenols, catechols, pyrogallols, phenols | N | not adjusted | NR |
| Chan et al., 2007(19) | Hong Kong Perimenopausal Osteoporosis Study (HKPOST) | Hong Kong | 141 subjects, women 100%, age 45-55y | 24h recall (measured period last 24h, repeated 23 times (7 weekend days and 16 weekdays), not validated) | The CU Soy Isoflavone Database | isoflavones | N | not adjusted | NR |
| Laurin et al., 2004(20) | Honolulu Heart Program | Japan | 8006 subjects, men 100%, age 45-68y | 24h recall (measured period last 24h, repeated Y - 3 examinations , not validated) | published | flavonoids | N | adjusted for energy intake | NR |
| Ahn et al., 2020(21) | Korea National Health and Nutrition Examination Survey (KNHANES) | Korea | 10326 subjects, men 39%, age 19-24y | 24h recall (measured period last 24h, repeated once, not validated) | MIxed source | flavonoids: flavonols, flavones, flavanones, flavan-3-ols, isoflavones, anthocyanidins, and proanthocyanidins | N | adjusted for energy intake | NR |
| Ham et al., 2019(22) | Korea National Health and Nutrition Examination Survey (KNHANES) | Korea | 14209 subjects, men 42%, age NR | 24h recall (measured period last 24h, repeated once, not validated) | mixed source | flavonoids: flavonols, flavones, flavanones, flavan-3-ols, isoflavones, and proanthocyanidins | N | adjusted for energy intake | NR |
| Jun et al., 2015(23) | Korea National Health and Nutrition Examination Survey (KNHANES) | Korea | 33581 subjects, men 41%, age 19+y | 24h recall (measured period last 24h, repeated NA, not validated) | USDA+ Phenol-Explorer + Korea Functional Food Composition Table | flavonoids: flavonols, flavones, flavanones, flavan-3-ols, isoflavones, anthocyanidins, and proanthocyanidins | N | adjusted for energy intake | NR |
| Jun et al., 2018(24) | Korea National Health and Nutrition Examination Survey (KNHANES) | Korea | 33581 subjects, men 41%, age 19-75y | 24h recall (measured period last 24h, repeated once, not validated) | mixed source | flavanoids | N | adjusted for energy intake | NR |
| Kim et al., 2020(25) | Korea National Health and Nutrition Examination Survey (KNHANES) | Korea | 23118 subjects, men 40%, age 19+y | 24h recall (measured period last 24h, repeated once, not validated) | mixed source | flavonoids: flavonols, flavones, flavanones, flavan-3-ols, anthocyanidins, isoflavones, and proanthocyanidins | N | adjusted for energy intake | NR |
| Yang et al., 2012(26) | Korea National Health and Nutrition Examination Survey (KNHANES) | Korea | 4745 subjects, men 39%, age 20-69y | 24h recall (measured period last 24h, repeated once, not validated) | mixed source | flavanoids: flavan-3-ol | N | adjusted for energy intake | NR |
| Bai et al., 2014(27) | NHANES | USA | 17900 subjects, men 47%, age 18+y | 24h recall (measured period last 24h, repeated once, not validated) | USDA | flavoniods: flavonols, flavones, flavanoes, flavan-3-ols, anthcyanidins, proanthocyanidins, isoflavones | N | not adjusted | NR |
| Chun et al., 2009(28) | NHANES | USA | 2908 subjects, men 51%, age >19y | 24h recall (measured period last 24h, repeated once, validated) | USDA | isoflavones : daidzein, genistein, glycitein, biochanin A, and formononetin | Y | 0 | urine: isoflavones: genistein, daidzein, ODMA, equol, urinary concentrations vs. dietary isoflavone intakes total isoflavones r=0.48 (genistein 0.45 daidzein 0.44, daidzein metabolites 0.46) (P<0.01) |
| Kim et al., 2016(29) | NHANES | USA | 4041 subjects, men 51%, age 19+y | 24h recall (measured period last 24h, repeated once, not validated) | USDA | flavonoids, anthocyanidins | N | adjusted for energy intake | NR |
| Kim et al., 2016(30) | NHANES | USA | 18634 subjects, men 49%, age 19-70y | 24h recall (measured period last 24h, repeated once, not validated) | USDA | flavonoids: flavonols, fla- vones, flavanones, flavan-3-ols, and anthocyanidins, isoflavones | N | adjusted for energy intake | NR |
| Ock et al., 2008(31) | NHANES | USA | 8335 subjects, men 51%, age 19+y | 24h recall (measured period last 24h, repeated once, not validated) | USDA | flavonoids: flavonols, flavones, flavanones, flavanols, anthocyanidins, if | N | not adjusted | NR |
| Mervish et al., 2013(32) | NIEHS/NCI BCERP | USA | 1178 subjects, women 100%, age 6-8y | 24h recall (measured period last 24h, repeated 2-4 per year, not validated) | Phenol-Explorer | lignans and flavonols | N | adjusted for energy intake | urine: Enterolactone, urinary enterolactone concentrations vs.dietary lignan intake (r= 0.13) |
| Mervish et al., 2017(33) | NIEHS/NCI BCERP | USA | 1044 subjects, women 100%, age 6-8y | 24h recall (measured period last 24h, repeated 2-4 per year, not validated) | USDA | lignans and flavonols | N | adjusted for energy intake | NR |
| Somerset et al., 2008(34) | NNS95 | Australia | 10851 subjects, NR, age 18+y | 24h recall (measured period last 24h, repeated once, not validated) | USDA | flavonoids: anthocyanidins, flavan-3-ols, flavanones, flavones, flavanols | N | not adjusted | NR |
| Waskiewicz et al., 2019(35) | Polish National Multicenter Health Survey (WOBASZ I and II) | Poland | 387 histologically confirmed thyroid cancer cases and 433 population-based controls subjects, women 100%, age 21-84y | 24h recall (measured period last 24h, repeated once, not validated) | Phenol-Explorer | total polyphenols | N | adjusted for age and season | NR |
| Witkowska et al., 2015(36) | Polish National Multicenter Health Survey (WOBASZ I and II) | Poland | 6661 subjects, men 47%, age 20-74y | 24h recall (measured period last 24h, repeated once, not validated) | mixed source | flavonoids: anthocyanins, chalcones, dihydrochalcones, dihydroflavonols, flavanols, flavanones, flavones, flavonols, and isoflavones;  phenolic acids included hydroxybenzoic acids, hydroxycinnamic acids, and hydroxyphenacetic acids; and the remaining polyphenols included lignans, stilbenes, others. | N | not adjusted | NR |
| Witkowska et al., 2018(37) | Polish National Multicenter Health Survey (WOBASZ I and II) | Poland | 2599 subjects, women 100%, age 20–74y | 24h recall (measured period last 24h, repeated once, not validated) | published | lignans | N | not adjusted | NR |
| Zujko et al., 2015(38) | Polish National Multicenter Health Survey (WOBASZ I and II) | Poland | 6661 subjects, men 47%, age 20-74y | 24h recall (measured period last 24h, repeated once, not validated) | self-analysed | flavonoids | N | adjusted for energy intake | NR |
| Zujko et al., 2015(39) | Polish National Multicenter Health Survey (WOBASZ I and II) | Poland | 643 cases and 643 controls subjects, men cases and controls 56%, age 20-74y | 24h recall (measured period last 24h, repeated once, not validated) | self-analysed | total polyphenols, total flavonoids | N | not adjusted | NR |
| Zujko et al., 2018(40) | Polish National Multicenter Health Survey (WOBASZ I and II) | Poland | 5690 subjects, men 45%, age 20+y | 24h recall (measured period last 24h, repeated once, not validated) | Phenol-Explorer +Polish databses | total polyphenols | N | not adjusted | NR |
| Adriouch et al., 2018(41) | SU.VI.MAX study | France | 3903 subjects, men 53%, age 45-60y | 24h recall (measured period last 24h, repeated every 2 months for a total of 6 records per year, not validated) | Phenol-Explorer | all classes: all subclasses | N | adjusted for energy intake | NR |
| Julia et al., 2016(42) | SU.VI.MAX study | France | 6092 subjects, men 42%, age 35–64 y | 24h recall (measured period last 24h, repeated 7 times in 4 seasons, not validated) | Phenol-Explorer | flavonoids and phenolic acids: flavanols, one dihydroflavonol, four anthocyanins, three flavanones, three flavones, eight flavonols, two hydroxybenzoic acids, thirteen hydroxycinnamic acids and one other polyphenols | N | adjusted for energy intake | NR |
| Perez-Jimenez et al., 2011(43) | SU.VI.MAX study | France | 4942 subjects, men 53%, age 45-60y | 24h recall (measured period last 24h, repeated every 2 months for a total of 6 records per year, not validated) | Phenol-Explorer | all classes: all subclasses: 337 individual pp | N | not adjusted | NR |
| Touvier et al., 2013(44) | SU.VI.MAX study | France | 4141 subjects, women 100%, age 55+y | 24h recall (/ items, measured period last 24h, conducted every 2 months for a total of 6 records per year, not validated) | Phenol-Explorer | all classes: all major subclasses | N | adjusted for energy intake | NR |
| Andrews et al., 2015(45) | The BioCycle Study | USA | 246 subjects, women 100%, age 18-44y | 24h recall (measured period last day , repeated 8 times, validated) | USDA | isoflavones | N | not adjusted | NR |
| Filiberto et al., 2013(46) | The BioCycle Study | USA | 259 subjects, women 100%, age 18-44y | 24h recall (measured period last 24h, repeated 8 times, not validated) | USDA | isoflavones: daidzein, genistein, glycitein, biochanin A, and formonontein | N | not adjusted | NR |
| Wisnuwardani et al., 2019(47) | the HELENA study | Europe | 7252 subjects, men 33%, age cohort1 40–59, cohort2 40–69y | 24h recall (measured period last 24h, repeated twice, not validated) | Phenol-Explorer | total polyphenols, flavonoids, phenolic acids : 384 individual polyphenols | N | not adjusted | NR |
| Sebastian et al., 2015(48) | WWEIA NHANES 2007-2008 | USA | 5420 subjects, men 49%, age 20+y | 24h recall (measured period last 24h, repeated once, validated) | USDA | flavonoids: anthocyanidins, flavan-3-ols, flavanones, flavones, flavonols, and isoflavones | N | not adjusted | NR |
| Sebastian et al., 2017(49) | WWEIA NHANES 2007-2009 | USA | 10538 subjects, men 50%, age 20+y | 24h recall (measured period last 24h, repeated once, validated) | USDA | flavonoids: anthocyanidins, flavan-3-ols, flavanones, flavones, flavonols, and isoflavones | N | not adjusted | NR |
| Balbi et al., 2019(50) |  | Brazil | 785 subjects, women 100%, age 20+y | 24h recall (measured period last 24h , repeated twice, not validated) | USDA | flavonoids: flavonols, flavones, flavanones, flavan-3ols and anthocyanidins | N | adjusted for energy intake | NR |
| **48h-recall (p8)** | | | | | | | | | |
| Tseng et al., 2013(51) |  | USA | 224 subjects, women 100%, age 36–58 y | 48-hour recalls (measured period 2 days (at least 1 weekend day), repeated twice, validated) | USDA | isoflavones | N | not adjusted | urine: equol and daidzein, NR |
| **3DD (p8-9)** | | | | | | | | | |
| Arai et al., 2000(52) |  | Japan | 106 subjects, women 100%, age 29-78y | 3DD (measured period last 3 days, repeated once, not validated) | Japanese food composition tables | isoflavones: genistein and daidzein | N | adjusted for energy intake | plasma, urine: genistein and daidzein/ genistein, daidzein, equol and ODMA, energy adjusted dietary intake of daidzein/genistein vs. urinary excretion (r=0.365 /0.346) , vs. plasma concentration ((r=0.335 /0.429) |
| Arai et al., 2000(53) |  | Japan | 115 subjects, women 100%, age mean 57.9y | 3DD (measured period last 3 days, repeated once, not validated) | self-analysed | flavonoids: flavonols, flavon,and isoflavones: myricetin, fisetin, quercetin and kaempferol/ luteolin/ daidzein and genistein | N | not adjusted | NR |
| Glabska et al., 2017(54) |  | Poland | 56 subjects, men 34%, age 18-80y | 3DD (measured period 2 random weekdays and 1 weekend day, repeated once, not validated) | USDA | isoflavones: daidzein, genistein, glycitein | N | adjusted for energy intake | NR |
| Wada et al., 2017(55) |  | Japan | 314 subjects, boys 52% mothers 100%, age children 5.4–7.2, mothers 27.8–47.6y | 3-day child's dietary record (measured period 2 weekdays and 1 weekend, repeated once, not validated) | Japanese food composition tables | isoflavones: genistein and daidzein | N | not adjusted | NR |
| Wada et al., 2017(56) |  | Japan | 159 mother-child pairs subjects, boys 52% mothers 100%, age 3-6y | 3DD (measured period 2 weekdays and 1 weekend, repeated once, not validated) | Japanese food composition tables | isoflavones | N | not adjusted | urine: daidzein, genistein, and equol, dietary intakes vs.urinary daidzein r= 0.155 (P = 0.060)/ genistein r=0.172 (P = 0.037) |
| Pedret et al., 2012(57) | PAScual MEDicina (PASMED) study | Spain | 81 subjects, men 43%, age 18-75y | 3DD (measured period 2 workdays +1 holiday or weekend, repeated once, not validated) | Phenol-Explorer | total polyphenols | N | not adjusted | urine, plasma: total polyphenols from Folin method, total dietary vs. urinary polyphenol (r = 0.281, p = 0.012) |
| Skolmowska et al., 2019(58) |  | Poland | 29079 subjects, men 46%, age 35+y | 3DD (measured period last 3 days, repeated once, not validated) | USDA | isoflavones: daidzein, genistein, glicitein | N | not adjusted | NR |
| **4DD (p9-10)** | | | | | | | | | |
| Barron et al., 2016(59) |  | USA | 118 subjects, women 100%, age 14-23y | 4DD (measured period last 4 days, repeated once, validated) | Nutrient Data System for Research | isoflavones: daidzein, genistrin | N | not adjusted | NR |
| Mursu et al., 2007(60) | Kuopio Ischaemic Heart Disease Risk Factor Study (KIHD) | Finland | 1380 subjects, men 100%, age 42-60y | 4DD (measured period last 4 days, repeated once, not validated) | USDA | flavonoids: anthocyanidins, flavan-3-ols, flavanones, flavones, flavonols, and isoflavones | N | adjusted for energy intake | NR |
| Mursu et al., 2008(61) | Kuopio Ischaemic Heart Disease Risk Factor Study (KIHD) | Finland | 2590 subjects, men 100%, age 42-60y | 4DD (measured period last 4 days, repeated once, not validated) | USDA | flavonoids: anthocyanidins, flavan-3-ols, flavanones, flavones, flavonols, and isoflavones | N | adjusted for energy intake | NR |
| Mursu et al., 2008(62) | Kuopio Ischaemic Heart Disease Risk Factor Study (KIHD) | Finland | 1950 subjects, men 100%, age 42-60y | 4DD (measured period last 4 days, repeated once, not validated) | USDA | flavonoids: anthocyanidins, flavan-3-ols, flavanones, flavones, flavonols, and isoflavones | N | adjusted for energy intake | NR |
| Ziauddeen et al., 2019(63) | UK NDNS Rolling Programme (RP) | UK | 427 subjects, men 61%, age mean men cases 60.1 controls 59.9, women cases 59.0 controls 59.0y | 4DD (measured period last 3 or 4 days, repeated once, not validated) | Phenol-Explorer | flavonoids: anthocyanins, flavan-3-ols (both monomers and proanthocyanidins), flavanones, flavones, flavonols, isoflavones, theaflavins, chalcones, hydroxybenzoic acids, hydroxycinnamic acids, and hydroxyphenylpropanoic acids, as well as stilbenes and total phenol content through the folin assay method. | N | adjusted for energy intake | NR |
|  |  |  |  | **7DD (p10-12)** |  |  |  |  |  |
| Cao et al., 2010(64) |  | China mainland | 92 subjects, NR, age 20-28y | 7DD (measured period last 7 days, repeated once, validated) | self-analysed | flavonoids: quercetin,kaempferol,isorhamnetin,apigenin,luteolin | Y | energy asjusted and unadjusted | plasma: flavonol and flavone, including quercetin, kaempferol, isorhamnetin, apigenin, and luteolin, quercetin intake vs. the sum of plasma flavonolr=0.52, isorhamnetin intake vs. the sum of plasma flavonol 0.39, apigenin intake vs. the sum of plasma flavonoid r=0.55, and luteolin intake vs. the sum of plasma flavonoid concentrations r= 0·56 |
| Radtke et al., 2002(65) |  | Germany | 48 subjects, women 100%, age 22-36y | 7DD (measured period last 7 days, repeated once, not validated) | published | flavonoids: quercetin, kaempferol, naringenin, and hesperetin | N | not adjusted | fasting plasma: plasma quercetin, kaempferol, naringenin, and hesperetin, All four flavonoids significant correlations between 7DD vs. plasma (r = 0.30–0.46, p < 0.05) higher correlation coefficients between intake of last day before blood sampling (r = 0.42–0.64; p < 0.01) |
| Taguchi et al., 2015(66) |  | Japan | 231112 subjects, NR, age 1–99y | 7DD (measured period last week, repeated once, not validated) | published | total polyphenols | N | not adjusted | NR |
| Taguchi et al., 2017(67) |  | Japan | 56 subjects, men 100%, age mean 37.9y | 7DD (measured period last week, repeated once, validated) | published | total polyphenols | Y | not adjusted | NR |
| Wang et al., 2012(68) |  | USA | 40 subjects, women 100%, age 40–70y | 7DD (measured period last 7 days, repeated once, not validated) | USDA | total polyphenols | N | not adjusted | plasma: plasma total phenolics, NR |
| Maras et al., 2011(69) | Baltimore Longitudinal Study on Aging (BLSA | USA | 1638 subjects, men 57% in 1980, 51% in 1990 and 50% in 2000, age 22-88y | 7DD (measured period last 7 days, repeated once, not validated) | USDA | flavoniods: flavonols, flavanones, flavones, flavan-3-ols, anthocyanidin, isoflavoness | N | adjusted for age and sex | NR |
| Grace et al., 2004(70) | EPIC-Norfolk | UK | 333 subjects, women 100%, age 45-75y | 7DD (measured period last week, repeated once, validated) | published | isoflavones: daidzein and genistein | Y | not adjusted | serum, urine: isoflavones and lignans/ isoflavones, urine vs. 7DD isoflavone (r=0.27), serum vs 7DD isoflavone (r=0.31) |
| Kuhnle et al., 2011(71) | EPIC-Norfolk | UK | 7553 subjects, men 66%, age 40-75y | 7DD (measured period last 7 days, repeated once, not validated) | DINER | phytoestrogens: biochanin, daidzein, formononetin, genistein, glycitein, glycitein, matairesinol, secoisolariciresinol, coumestrol,enterodiol, enterolactone, equol | N | not adjusted | NR |
| Mulligan et al., 2007(72) | EPIC-Norfolk | UK | 11843 subjects, men 49%, age 39+y | 7DD (measured period last 7 days, repeated once, validated) | DINER | isofalvones: daidzein, genistein | N | adjusted for energy intake | NR |
| Mulligan et al., 2013(73) | EPIC-Norfolk | UK | 20437 subjects, men 47%, age 40-75y | 7DD (measured period last 7 days, repeated once, validated) | DINER | phytoestrogens: coumesterol, enterolignans, isoflavones, lignans | N | adjusted for energy intake | NR |
| Vogiatzoglou et al., 2015(74) | EPIC-Norfolk | UK | 24885 subjects, men 45%, age 40- 75 y | 7DD (measured period last 7 days, repeated once, validated) | USDA+ Phenol-Explorer | flavan-3-ol : flavan-3-ols monomers, theaflavins and proanthocyanidins | N | not adjusted | NR |
| Ward et al., 2010(75) | EPIC-Norfolk | UK | breast cancer: 244 cases and 941 controls, colorectal cancer: 221 cases and 886 controls, prostate cancer: 204 cases and 812 controls subjects, NR, age 40-79y | 7DD (measured period last 7 days, repeated once, validated) | DINER | phytoeostrogens: isoflavones, lignans, and coumesterols | N | not adjusted | NR |
| **30DD (p12)** | | | | | | | | | |
| Wang et al., 2012(76) |  | USA | 60 subjects, men 33%, age 18-25 y | 30DD (measured period last month, repeated once, not validated) | USDA | flavonoids: 29 flavonoids, three proanthocyanidins | N | not adjusted | NR |
| **3-day weighted dietary records (p12-14)** | | | | | | | | | |
| Pellegrini et al., 2010(77) |  | Italy | 242 subjects, men 62%, age mean 60y | 3-day weighted dietary records (measured period 2 non-consecutive workdays + a weekend day, repeated once, not validated) | European Institute of Oncology (EIO) database | lignans: mat, seco, pino, lari | N | adjusted for energy intake | NR |
| Rosi et al., 2020(78) |  | Italy | 10812 subjects, women 100%, age mean 34.7y | 3-day weight food diary (measured period last 3 days, repeated twice, not validated) | Phenol-Explorer | flavanoid and phenolic acids: anthocyanins, flavan-3-ols—both monomers and proanthocyanidins, flavanones, flavones, flavonols, isoflavones, theaflavins, chalcones, hydroxybenzoic acids, hydroxycinnamic acids, and hydroxyphenylpropanoic acids | N | not adjusted | NR |
| Cheng et al., 2010(79) | Dortmund Nutritional and Anthropometric Longitudinally Designed (DONALD) Study, | Germany | 227 subjects, boy 48%, age mean girl 7.2, boy 8.8y | 3-day weighted dietary records (measured period last 3 days, repeated every year on birthday, not validated) | VENUS database | isoflavones: daidzein, genistein | N | not adjusted | urine: isoflavones (daidzein, genistein, and equol), No correlations between dietary intake (on the third day of the 3-d weighed dietary) and urinary isoflavone excretion in girls (r = 20.06, P = 0.6) or in boys (r = 0.16, P = 0.2) |
| Drossard et al., 2013(80) | Dortmund Nutritional and Anthropometric Longitudinally Designed (DONALD) Study, | Germany | 920 subjects, boy 50%, age 4-18y | 3-day weighted dietary records (measured period last 3 days, repeated every year on birthday, not validated) | USDA | anthocyanidins: cyanidin, delphinidin, malvidin, pelargonidin, peonidin and petunidin | N | adjusted for energy intake | NR |
| Krupp et al., 2016(81) | Dortmund Nutritional and Anthropometric Longitudinally Designed (DONALD) Study, | Germany | 717 subjects, men 47%, age 18-39y | 3-day weighted dietary records (measured period last 3 days, repeated every year on birthday, not validated) | USDA | flavonoids | N | adjusted for energy intake | urine: hippuric acid , NR |
| Penczynski et al., 2017(82) | Dortmund Nutritional and Anthropometric Longitudinally Designed (DONALD) Study, | Germany | 287 subjects, men 48% , age 9-16y | 3-day weighted dietary records (measured period last 3 days, repeated every year on birthday, validated) | USDA | flavonoids: hippuric acid | N | adjusted for energy intake | 24-hour urine: Hipolyphenolsuric acid, NR |
| Penczynski et al., 2018(83) | Dortmund Nutritional and Anthropometric Longitudinally Designed (DONALD) Study, | Germany | 257 subjects, men 48% Diet group/ 49% Urine group, age 9-16y | 3-day weighted dietary records (measured period last 3 days, repeated every year on birthday, validated) | USDA | flavonoids: flavones, flavonols, flavan-3-ol -monomers, flavanones, anthocyanidins and proanthocyanidins | N | adjusted for energy intake | 24-hour urine: Hipolyphenolsuric acid, NR |
| Penczynski et al., 2019(84) | Dortmund Nutritional and Anthropometric Longitudinally Designed (DONALD) Study, | Germany | 268 subjects, men 48%, age 9-16y | 3-day weighed dietary records (measured period last 3 days, repeated every year on birthday, not validated) | USDA | flavonoids, proanthocyanidins: flavones, flavonols, flavan-3-ol-monomers, flavanones and anthocyanidins + proanthocyanidin | N | adjusted for energy intake | urine: hippuric acid , NR |
| Nakamoto et al., 2018(85) | National Institute for Longevity Sciences-Longitudinal Study of Aging (NILS-LSA) | Japan | 776 subjects, men 52%, age 60-81y | 3-day weighted dietary records (measured period 2weekdays + 1 weekend, repeated once, not validated) | not reported | isoflavones: daidzein, genistein, glycetin | N | not adjusted | NR |
| **4-day weighted dietary records (p14)** | | | | | | | | | |
| Kent et al., 2015(86) | Blue Mountains Eye Study (BMES) | Australia | 79 subjects, men 43%, age 49+y | 4-day weighted dietary records (measured period 4 days , repeated 3 times at 4-month intervals, not validated) | USDA | flavonoids: flavan 3-ols, flavonols, flavones, flavanones, anthocyanidins | N | not adjusted | NR |
| **centre-specific methods (p14-16)** | | | | | | | | | |
| Kyro et al., 2015(87) | EPIC | Europe | 11782 subjects, women 100%, age 35-70y | centre-specific methods (measured period Last year, repeated once, validated) | Phenol-Explorer | all classes: lignans, flavonoids, phenolic acids, stilbenes, other polyphenols | N | not adjusted | NR |
| Molina-Montes et al., 2016(88) | EPIC | Europe | 477309 subjects, men 30%, age 25-70y | centre-specific methods (measured period NR, repeated once, validated) | Phenol-Explorer +USDA+UK Food Standards Agency database | flavonoids and lignans: flavanols, flavan-3-ols monomers, proanthocyanidins,theaflavins, anthocyanidins, flavonols, flavanones, flavones, isoflavones, lignans | N | not adjusted | NR |
| Vermeulen et al., 2012(89) | EPIC | Europe | 477312 subjects, men 30%, age 35-70y | centre-specific methods (measured period Last year, repeated once, validated) | mixed source | flavonoids and lignans: flavanols, flavan-3-ols monomers, proanthocyanidins,theaflavins, anthocyanidins, flavonols, flavanones, flavones, isoflavones, lignans | Y | not adjusted | NR |
| Zamora-Ros et al., 2012(90) | EPIC | Europe | 340234 subjects, men 38%, age 35-70y | centre-specific methods (98-266 items, measured period last year, conducted once, not validated) | mixed source | flavonoids and lignans: flavanols, flavan-3-ols monomers, proanthocyanidins,theaflavins, anthocyanidins, flavonols, flavanones, flavones, isoflavones, lignans | N | not adjusted | NR |
| Zamora-Ros et al., 2013(91) | EPIC | Europe | 477312 subjects, men 30%, age 35-70y | centre-specific methods (150–250 items, measured period last year, conducted once, validated) | Phenol-Explorer | flavonoids: flavanols, flavan-3-ols monomers, proanthocyanidins,theaflavins, anthocyanidins, flavonols, flavanones, flavones, isoflavones | Y | not adjusted | NR |
| Zamora-Ros et al., 2013(92) | EPIC | Europe | 26088 subjects, men 38%, age 35-70y | centre-specific methods (measured period NR, repeated once, validated) | mixed source | flavanoids: flavanols (flavan-3-ol monomers, proanthocyanidins, and theaflavins) and flavonols | Y | not adjusted | NR |
| Zamora-Ros et al., 2013(93) | EPIC | Europe | 477312 subjects, men 30%, age 35-70y | centre-specific methods (measured period NR, repeated once, validated) | mixed source | flavonoids: (anthocyanidins, flavanols, flavanones, flavonols, flavones, and isoflavones | N | not adjusted | NR |
| Zamora-Ros et al., 2014(94) | EPIC | Europe | 477312 subjects, men 30%, age 35–70 y | centre-specific methods (measured period NR, repeated once, validated) | USDA+ Phenol-Explorer +UK Food Standards Agency database | flavonoids and lignans: flavanols, flavan-3-ols monomers, proanthocyanidins,theaflavins, anthocyanidins, flavonols, flavanones, flavones, isoflavones, lignans | N | not adjusted | NR |
| Zamora-Ros et al., 2017(95) | EPIC | Europe | 476160 subjects, men 30%, age 35-70y | centre-specific methods (measured period last year, repeated once, validated) | Phenol-Explorer | total polyphenols: all subclasses | Y | not adjusted | NR |
| Zamora-Ros et al., 2017(96) | EPIC | Europe | 475 subjects, men 42%, age 50–61y | 24h recall+centre specific (measured period last 24h, repeated once, not validated) | Phenol-Explorer | stillbenes: resveraterol | N | not adjusted | urine: 24h urine, urinary excre tion vs. dietary stilbenes (24-HDR/DQ) r =0·61/0·55 |
| Zamora-Ros et al., 2018(97) | EPIC | Europe | 477206 subjects, men 30%, age 35-70y | centre-specific methods (measured period NR, repeated once, validated) | mixed source | flavonoids and lignans: flavanols, flavan-3-ols monomers, proanthocyanidins, theaflavins, anthocyanidins, flavonols, flavanones, flavones, isoflavones, lignans | Y | not adjusted | NR |
| Zamora-Ros et al., 2020(98) | EPIC | Europe | 78 cases and 78 controls subjects, women 100%, age 15-45 y | centre-specific methods (measured period NR, repeated once, validated) | Phenol-Explorer | total polyphenols: all subclasses | N | adjusted for energy intake | NR |
| Tahiri et al., 2020(99) | EPIC | Europe | 56 subjects, men 34%, age 18-80y | centre-specific methods (measured period NR, repeated once, not validated) | Phenol-Explorer | flavanones, glycosides and aglycones | N | not adjusted | 24h urine: Naringenin and hesperetin, urinary flavanone excretion vs. acute and habitual intake (r = 0·15–0·20) |
| Nothlings et al., 2008(100) | MEC study (Multi-Ethnic Cohort) and EPIC | USA, Europe | 183513 for MEC, 424978 for EPIC subjects, men 45%, age MEC 45-75, EPIC 35-70y | FFQ, centre specific (measured period MEC: last year, EPIC: NR, repeated once, validated) | food tables developed by the Cancer Research Center of Hawaii for the MEC | flavonols : quercetin / kaempferol / myricetin | N | not adjusted | NR |
| Lako et al., 2006(101) | Naduri Longitudinal study, Suva-Nausori Corridor cross-sectional study, the 1999 Verata cross-sectional study, and the 2001 Fiji Food Choice study. | Fiji | 7752 subjects, NR, age NR | centre-specific: 7-weighed food diary, 24h recall, 7DD (measured period NR, repeated once, not validated) | published | flavonoids: flavonols, anthocyanidins | N | not adjusted | NR |
| **DHI (p17)** | | | | | | | | | |
| Lei et al., 2002(102) |  | Italy | 94 cirrhosis and 32 HCC patients subjects, men cirrhosis 64%, HCC 78%, age mean cirrhotic 56, HCC 65y | dietary history interview (measured period last year, repeated once, not validated) | unpublished data | isoflavones: genistein,daidzein, secoisolariciresinol, matairesinol | N | not adjusted | NR |
| Marniemi et al., 2005(103) |  | Finland | 756 subjects, men 48%, age 65-99y | dietary history interview (measured period last 2 months, repeated once, not validated) | Fineli database | flavonoids: quercetin, kaempferol, myricetin, apigenin and luteolin | N | not adjusted | NR |
| Knekt et al., 1996(104) | Finnish mobile clinic health examination | finland | 5133 subjects, men 54%, age 30-69y | dietary history interview (measured period NR, repeated na, validated) | published | flavonoids: querceti, kaemferol, myricetin, lueolin, apigenin | Y | not adjusted | NR |
| Knekt et al., 1997(105) | Finnish mobile clinic health examination | finland | 9959 subjects, men cases: lung cancer 96%/ other cancer 49%, controls 53%, age 15-99y | dietary history interview (100 items, measured period last year, conducted once, not validated) | published | flavonoids: quercetin, kaempferol, myricetin, luteolin, and apigenin | N | not adjusted | NR |
| Knekt et al., 2000(106) | Finnish mobile clinic health examination | finland | 9208 subjects, men 54%, age >15y | dietary history interview (measured period last year, repeated once, validated) | published | flavonoids: quercetin, kampferol, myricetin, luteolin and apigenin | Y | not adjusted | NR |
| **DHQ (p17-19)** | | | | | | | | | |
| Cui et al., 2020(107) |  | Japan | 1335 subjects, men 100%, age 19-83y | brief diet history questionnaire (BDHQ) (75 (3 soy food used to measure intake) items, measured period last month, conducted once, validated) | Standard Tables of Food Composition in Japan | isoflavones: daidzein and genistein | N | adjusted for energy intake | NR |
| Fukushima et al., 2020(108) |  | Japan | 244 subjects, women 100%, age 30-60y | brief diet history questionnaire (BDHQ) (measured period NR, repeated once, validated) | published | total polyphenols | N | not adjusted | NR |
| Garcia-Closas et al., 1999(109) |  | Spain | 497 cases and 1113 controls subjects, NR, age NR | dietary history questionnaire (60 items, measured period last year, conducted once, validated) | published | flavonoids: quercetin, kaempferol, myricetin, and luteolin | N | not adjusted | NR |
| Garcia-Closas et al., 1999(110) |  | Spain | 354 cases and 354 controls subjects, men 66%, age 31-88y | dietary history questionnaire (77 items, measured period last year, conducted once, validated) | published | flavonoids: quercetin, kaempferol, myricetin, and luteolin | N | not adjusted | NR |
| Taguchi et al., 2018(111) |  | Japan | 7960 subjects, men 100%, age 22–86y | brief diet history questionnaire (BDHQ) (58 items, measured period last month, conducted once, validated) | self-analysed by colleague | total polyphenols | Y | not adjusted | NR |
| Zamora-Ros et al., 2015(112) |  | Spain | 424 subjects, NR, age median 67-68y | DHQ (>600 items, measured period last year, conducted once, validated) | USDA+ Phenol-Explorer | flavonoids and lignans: flavanones, flavonols, anthocyanidins, flavones, and isoflavones | N | adjusted for energy intake | NR |
| Zamora et al., 2013(113) | EPIC-Spain | Spain | 40622 subjects, men 38%, age 26-69y | DHQ (>600 items, measured period NR, conducted once, validated) | USDA+ Phenol-Explorer | flavonoids and lignans: flavanone and flavonol | N | not adjusted | NR |
| Zamora-Ros et al., 2008(114) | EPIC-Spain | Spain | 41440 subjects, men 38%, age 35–64y | DHQ (>600 items, measured period Last year, conducted once, validated) | published | stilbenes: resveratrol and piceid | N | not adjusted | NR |
| Zamora-Ros et al., 2010(115) | EPIC-Spain | Spain | 40683 subjects, men 38%, age 35-64 y | DHQ (>600 items, measured period last year, conducted once, validated) | USDA+published | flavonoids: seven subgroups and their 35 individual flavonoids | N | standardized by sex and age ofthe population | NR |
| Miyake et al., 2018(116) | Kyushu Okinawa Maternal and Child Health Study (KOMCHS) | Japan | 1745 subjects, women 100%, age median 31y | DHQ (150 items, measured period last month, conducted once, validated) | Japanese food composition tables | isoflavones: genistein, daidzein | N | adjusted for energy intake | NR |
| Zamora-Ros et al., 2013(117) | the Bellvitge Colorectal Cancer Study | Spain | 424 cases and 401 controls subjects, men cases 60% controls 52%, age median?+Q39:AB39 cases 65.1, controls 66.2y | DHQ (600 food and beverages+ 150 recipes items, measured period 1 year before diagnosis/ interview, conducted once, validated) | Phenol-Explorer +UK Food Standards Agency database | flavonoids: anthocyanidins, flavanols, proanthocyanidins, theaflavins, flavones,  flavonols, flavanones, isoflavones, and lignans. | N | adjusted for energy intake | NR |
| **FFQ (p19-75)** | | | | | | | | | |
| Lewis et al., 2009(118) |  | USA | 478 cases and 382 controls subjects, men 100%, age 0 incident case 63.3, prevelant cases 66.9, controls 62y | FFQ (100 items, measured period last year, conducted once, not validated) | not reported | isoflavones: (genistein, daidzein) | N | not adjusted | NR |
| Abulimiti et al., 2020(119) |  | China mainland | 2502 cases 2538 controls subjects, men cases and controls 57%, age 30-75y | FFQ (81 items, measured period 1 year before diagnosis/interview, conducted once, validated) | mixed source | flavonoids: flavan-3-ol, flavones, flavonols, flavonones, anthocyanidins,isoflavones | N | not adjusted | NR |
| Alipour et al., 2016(120) |  | Iran | 170 subjects, women 100%, age 20-48y | FFQ (168 items, measured period last year, conducted once, validated) | USDA+ Phenol-Explorer | flavonoids : flavonol, flavones,flavanones, flavan-3-ols, theaflavins, anthocyanidins, and isoflavones | N | adjusted for energy intake | NR |
| Atkinson et al., 2002(121) |  | USA | 360 subjects, women 100%, age 25-59y | FFQ(recall)/FFQ (measured period last 24h/last 2 years, repeated 2 consecutive days/once, validated) | USDA | isoflavones: genistein and daidzein | N | not adjusted | urine: total daidzein, genistein, ODMA, and equol , isoflavone intake (sum of genistein and daidzein) vs. mean excretion (sum of daidzein, genistein, ODMA, and equol) (r=0.39, P = 0.07) over the 2 days for women who consumed soy on both days of the diet recall |
| Bahrami et al., 2019(122) |  | Iran | 129 colorectal cancer cases 130 colorectal adenoma cases and 240 controls subjects, men cases 51%/45% controls 55%, age mean cases 57/56 controls 55y | FFQ (148 (80 used in analysis) items, measured period 1 year before diagnosis/ interview, conducted once, validated) | Phenol-Explorer | total polyphenols: flavonoids, phenolic acids, lignans, stilbenes | N | not adjusted | NR |
| Bobe et al., 2009(123) |  | USA | 161 white men with esophageal adenocarcinoma (EAC), 114 white and 218 black men with esophageal squamous cell carcinoma (ESCC) and 678 white and 557 black male controls subjects, men 100%, age 30-79y | FFQ (57+18 items, measured period last 5 years, conducted once, not validated) | USDA | flavonoids: anthocyanidins,flavan-3-ols, flavanones, flavones,flavonols, isoflavonoids, proanthocyanidin | N | not adjusted | NR |
| Bosetti et al., 2005(124) |  | Italy | 2569 cases and 2588 control subjects, women 100%, age case 23-74, control 20-74y | FFQ (78 items, measured period last 2 years, conducted once, validated) | USDA | flavonoids: flavanones, flavan-3-ols, flavonols, flavones, anthocyanidins, and isoflavones | N | not adjusted | NR |
| Bosetti et al., 2006(125) |  | Italy | 1294 cases and 1451 control subjects, men 100%, age 46-74y | FFQ (78 items, measured period last 2 years, conducted once, validated) | USDA | flavonoids: flavanones, flavan-3-ols, flavonols, flavones, anthocyanidins, and isoflavones | N | not adjusted | NR |
| Chan et al., 2007(126) |  | Hong Kong | 126 subjects, men 69%, age mean 66.5y | FFQ (77 items, measured period last 5 yearsears, conducted once, validated) | Chinese food composition table | isoflavones | N | not adjusted | NR |
| Chan et al., 2011(127) |  | Hong Kong | 2217 subjects, men 55%, age 65+y | FFQ (13 food groups items, measured period last year, conducted once, validated) | Chinese food composition table | isoflavones | N | adjusted for energy intake | NR |
| Chan et al., 2011(128) |  | Hong Kong | 102 subjects, men 78%, age mean 66.5y | FFQ (77 items, measured period last year, conducted once, validated) | Chinese food composition table | isoflavones | N | not adjusted | NR |
| Chan et al., 2012(129) |  | Hong Kong | 127 subjects, men 69%, age mean 66.9y | FFQ (77 items, measured period last year, conducted once, validated) | Chinese food composition table | isoflavones | N | not adjusted | NR |
| Cui et al., 2008(130) |  | USA | 558 cases and 837controls subjects, men case 51%, control 61%, age 18-65y | FFQ (78 items, measured period last year, conducted once, validated) | USDA | flavonoes: all subclasses | N | not adjusted | NR |
| Cui et al., 2015(131) |  | Japan | 1076 subjects, men 77%, age 20-78y | FFQ (75 items, measured period last year, conducted once, validated) | Japanese food composition tables | isoflavones: genistein and daidzein | N | adjusted for energy intake | NR |
| De Stefani et al., 1999(132) |  | Uruguay | 541 cases and 540 controls subjects, men 100%, age 30-89y | FFQ (64 items, measured period last year, conducted once, validated) | published | flavonoids: quercetin, kaempferol | N | not adjusted | NR |
| Djuric et al., 2012(133) |  | USA | 2664 subjects, men case 50% control 43%, age 45-80y | FFQ (120 items, measured period last 2 years, conducted once, validated) | not reported | quercetin: quercetin | N | adjusted for energy intake | NR |
| Ekstrom et al., 2011(134) |  | Sweden | 505 cases and 1116 controls subjects, men 67%, age 40-79y | FFQ (45 items, measured period last 20 years, conducted once, validated) | published | quercetin | N | adjusted for energy intake | NR |
| Endoh et al., 2015(135) |  | Japan | 8305 subjects, men 46%, age 30-60y | FFQ (58 items, measured period NR, conducted once, validated) | not reported | isoflavones: daidzein, genistein | N | adjusted for energy intake | NR |
| Feng et al., 2019(136) |  | China mainland | 1522 cases 1547 controls subjects, women 100%, age 25-70y | FFQ (81 items, measured period 1 year before diagnosis/ interview, conducted once, validated) | mixed source | flavonoids: anthocyanidins, flavanols, flavanones, flavones and flavonols, isoflavones | N | adjusted for energy intake | NR |
| Fernandez-Navarro et al., 2018(137) |  | Spain | 124 subjects, men 31%, age 19-95y | FFQ (160 items, measured period last year, conducted once, validated) | Phenol-Explorer | all classes: all subclasses | Y | not adjusted | NR |
| Ferreira et al., 2019(138) |  | Brazil | 96 subjects, women 100%, age 45-65y | FFQ (121 items, measured period last month, conducted once, validated) | Phenol-Explorer | total polyphenol, isoflavones and lignan | N | not adjusted | NR |
| Fisher et al., 2012(139) |  | USA | 19 subjects, men 42%, age mean 72y | focused FFQ (22 items, measured period last week, conducted once, not validated) | USDA | flavonoids | N | not adjusted | NR |
| Frankenfeld et al., 2002(140) |  | USA | 77 subjects, men 47%, age 20-40y | FFQx2 (122+40 soy food items, measured period last 3 months, conducted once, validated) | USDA | isoflavones: genistein, daidzein | Y | not adjusted | plasma: daidzein and genistein, genistein/ daidzein intakes by soy FFQ vs. plasma concentrations (r=0.53/ 0.45), genistein/ daidzeinby WHI FFQ vs. plasma concentration (r=0.46/ 0.45) |
| Fukushima et al., 2015(141) |  | Japan | 131 subjects, women 100%, age 30-60y | FFQ (58 items, measured period NR, conducted once, validated) | published | total polyphenols | N | not adjusted | NR |
| Galvan-Portillo et al., 2007(142) |  | Mexico | 50 subjects, women 100%, age 17-37y | FFQ (54/100 items, measured period last year, conducted twice, validated) | USDA+Notice plus1998+ published data | phytoestrogens: flavonol, flavones, flavanol , secoisolariciresinol, matairesinol, lariciresinol , pinoresinol , cynamic acid and coumestrol. | Y | adjusted for energy intake | NR |
| Garavello et al., 2007(143) |  | Italy | 460 cases and 1088 control subjects, men case 90% control 79%, age 30-80y | FFQ (78 items, measured period last year, conducted once, validated) | mixed source | flavonoids: isoflavones, anthocyanidins, flavan-3-ols, flavanones, flavones and flavonols | N | adjusted for energy intake | NR |
| Garcia et al., 2005(144) |  | UK | 607 cases and 864 controls subjects, NR, age 16-50y | FFQ (>200 items, measured period last year, conducted once, validated) | published | flavonoids: catechins, flavonols and flavones | N | not adjusted | NR |
| Gates et al., 2009(145) |  | USA | 1141 cases and 1183 control subjects, women 100%, age mean 51y | FFQ (126 items, measured period last year, conducted once, validated) | USDA | flavonoids: myricetin, kaempferol, quercetin, luteolin, and apigenin | N | not adjusted | NR |
| Gonzalez et al., 2014(146) |  | Spain | 304 subjects, men 42%, age mean men 73.2, women 76.8y | FFQ (measured period last year, repeated once, not validated) | Phenol-Explorer | total polyphenols: all subclasses | N | not adjusted | NR |
| Hakim et al., 2000(147) |  | USA | 404 cases and 391 controls subjects, men 48%, age mean 66.4y | Tea questionnaire (measured period last year, repeated once, validated) | self-analysed | catechins, theaflavins and gallic acid | N | not adjusted | NR |
| Hardcastle et al., 2011(148) |  | UK | 5119 subjects, women 100%, age 45-54y | FFQ (98 items, measured period up to last 12 years, conducted once, validated) | USDA+published | flavonoids: flavonols, flavone,procyanidins, catechins, flavanones, | N | adjusted for energy intake | NR |
| Hernandez-Ramirez et al., 2009(149) |  | Mexico | 257 cases and 507 controls subjects, men cases 54% controls 54%, age cases 49-67, controls 49-70y | FFQ (127 items, measured period 3 years before diagnoses/ interview, conducted once, validated) | USDA+Notice plus1998+ published data | flavonoids, lignans: flavonol, flavanol, secoisolariciresinol, matairesinol, lariciresinol, pinoresinol; cinnamic acid and coumestrol | N | not adjusted | NR |
| Hirayama et al., 2010(150) |  | Japan | 278 cases and 340 controls subjects, men cases 87% controls 80%, age mean COPD pateitns men 66.5, women 66.1; controls men 65.15, women 66.12y | FFQ (138 items, measured period last 5 years, conducted NA, validated) | Japanese food composition tables | isoflavones: genistein and daidzein | N | not adjusted | NR |
| Ho et al., 2000(151) |  | Hong Kong | 1010 subjects, men 49%, age 24-75y | FFQ (253 items, measured period NR, conducted once, not validated) | published | isoflavones | N | not adjusted | NR |
| Ho et al., 2003(152) |  | Hong Kong | 454 subjects, women 100%, age 48-62y | FFQ (15 soy food items, measured period NR, conducted once, validated) | published | soy isoflavoness | N | not adjusted | NR |
| Horn-Ross et al., 2000(153) |  | USA | 118 subjects, women 100%, age 35-79y | FFQ (65 items, measured period NR, conducted once, not validated) | self-analysed | isoflavoness, lignans: these included the isoflavones: genistein, daidzein, biochanin A and formononetin; the coumestan: coumestrol; and the lignans: matairesinol and secoisolariciresinol | N | not adjusted | NR |
| Horn-Ross et al., 2000(154) |  | USA | 447 subjects, women 100%, age 50-79y | FFQ (measured period NR, repeated once, not validated) | published | isoflavones, lignans, coumestrol: secoisolariciresinol, matairesinol, coumestrol, formononetin, biochanin A, daidzein, genistein | N | not adjusted | NR |
| Horn-Ross et al., 2003(155) |  | USA | 500 cases and 470 controls subjects, women 100%, age 35-79y | FFQ (100 items, measured period last year, conducted once, not validated) | published | isoflavones, coumestans and lignans: isoflavones (genistein, daidzein, formononetin, and biochanin A), coumestans (coumestrol), and lignans (matairesinol and secoisolariciresinol) | N | adjusted for energy intake | NR |
| Hou et al., 2017(156) |  | Taiwan | 233 cases and 236 controls subjects, women 100%, age 28-83y | FFQ (28 items, measured period last month, conducted once, validated) | Chinese University of Hong Kong Soy Isoflavone Database. | isoflavones | N | not adjusted | NR |
| Iwasaki et al., 2009(157) |  | Japan, Brazil | 472 cases and 472 controls subjects, women 100%, age 20-74y | FFQ (169 Japan/188 Hawaii items, measured period NR, conducted once, validated) | published | isoflavones: daidzein genistein | Y | not adjusted | NR |
| Iwasaki et al., 2010(158) |  | Japan, Brazil | 850 cases and 850 controls subjects, women 100%, age 20-74y | FFQ (169 Japan/188 Hawaii items, measured period NR, conducted once, validated) | published | isoflavones: diadzein, genistein | Y | adjusted for energy intake | NR |
| Iwasaki et al., 2014(159) |  | Japan | 369 cases and 369 controls subjects, women 100%, age 20-74y | FFQ (136 items, measured period last year, conducted once, validated) | Japanese food composition tables | isoflavones | N | not adjusted | NR |
| Jiao et al., 2013(160) |  | USA | 1859 cases and 1500 controls subjects, men cases 97% controls 92%, age 40–80y | FFQ (110 items, measured period last year, conducted once, validated) | USDA | isoflavones | N | adjusted for energy intake | NR |
| Kim et al., 2006(161) |  | USA | 84 subjects, women 100%, age 28-40y | FFQ (17 items, measured period NR, conducted once, validated) | published | isoflavones | N | not adjusted | NR |
| Kreijkamp-Kaspers et al., 2004(162) |  | Netherlands | 301 subjects, women 100%, age 60-75y | FFQ (170 items, measured period last year, conducted once, validated) | mixed source | isoflavones,lignan | N | not adjusted | NR |
| Lammersfeld et al., 2009(163) |  | USA | 100 subjects, women 100%, age 31-70y | FFQ (specific for soy) (40 item soy FFQ items, measured period last 3 months , conducted once, not validated) | Nutrient Data System for Research | isoflavones: genistein and diadzien | N | not adjusted | NR |
| Lee et al., 2003(164) |  | China mainland | 133 cases and 265 controls subjects, women 100%, age 40-70y | FFQ (84 items, measured period last year, conducted once, not validated) | published | isoflavones, : genistein and daidzein | N | not adjusted | NR |
| Lee et al., 2006(165) |  | Korea | 397 subjects, women 100%, age 23-81y | FFQ ( 5 soy food items, measured period last year, conducted once, validated) | not reported | phytoestrogens | N | not adjusted | NR |
| Lee et al., 2014(166) |  | China mainland | 500 cases and 500 control subjects, women 100%, age mean 59y | FFQ (125 items, measured period last 5 years, conducted once, validated) | USDA | isoflavones: daidzein, genistein, glycitin | N | not adjusted | NR |
| Lee et al., 2019(167) |  | Korea | 728 subjects, women 100%, age 20-78y | FFQ (106 items, measured period last year, conducted once, validated) | not reported | flavonoids: flavan-3-ol, flavones, flavonols, flavonones, anthocyanidins, isoflavones | N | adjusted for energy intake | NR |
| Levi et al., 2005(168) |  | Switzerland | 369 cases and 602 controls. subjects, women 100%, age 23-74y | FFQ (79 items, measured period 2 years before diagnosis/admission, conducted once, not validated) | published | stilbenes: resveratrol | N | not adjusted | NR |
| Li et al., 2013(169) |  | China mainland | 1393 subjects, men 32%, age 35-70y | FFQ (76 items, measured period last year, conducted once, validated) | published | flavonoids and stilbenes: anthocyanidins, flavonols, flavones, isoflavones, and stilbenes | Y | not adjusted | NR |
| Li et al., 2013(170) |  | China mainland | 560 cases and 560 controls subjects, men 58%, age mean male 56, female 51y | FFQ (119 items, measured period last year, conducted once, validated) | USDA | isoflavones: daidzein, genistein, glycitein | N | not adjusted | NR |
| Lin et al., 2012(171) |  | Sweden | 181 cases of esophageal adenocarcinoma, 255 cases of gastroesophageol junctional adenocarcinoma, 158 cases of squamous cell carcinoma and 806 controls subjects, men cases: Esophagreal cancer 87%/ gastroesphageal cancer 85%/esophagreal suamous cell cancer 72%, control 83% | FFQ (63 items, measured period 20 years before interview, conducted once, validated) | published | lignans: matairesinol, secoisolariciresinol, lariciresinol, pinoresinol, syringaresinol, and medioresinol, pinoresinol | N | not adjusted | NR |
| Lin et al., 2014(172) |  | Sweden | 181 cases of oesophageal adenocarcinoma (OAC), 158 cases of oesophageal squamous-cell carcinoma (OSCC), 255 cases of gastro-oesophageal junctional adenocarcinoma (JAC) and 806 controls subjects, men cases Oesophageal adenocarcinoma 87%/ Oesophageal squamous-cell carcinoma 72%/ Gastro-oesophageal junctional adenocarcinoma 85%, control: 83% , age <80 y | FFQ (63 items, measured period last 20 years , conducted once, validated) | published | lignans, flavonoids: quercetin, resveratrol, matairesinol; secoisolariciresinol; lariciresinol; pinoresinol; syringaresinol; medioresinol | N | adjusted for energy intake | NR |
| Liu et al., 2013(173) |  | China mainland | 600 subjects, men 75%, age 32-73y | FFQ (78 items, measured period last year, conducted once, validated) | Chinese food composition table | isoflavones, : daidzein, genistein and glycitein | N | adjusted for energy intake | NR |
| Liu et al., 2015(174) |  | China mainland | 208 cases and 208 controls subjects, men cases and controls 53%, age 19-85y | FFQ (128 items, measured period last year, conducted once, not validated) | USDA | isoflavones: daidzein, genistein, glycitein | N | not adjusted | NR |
| Lu et al., 2016(175) |  | Sweden | 594 cases and 806 controls subjects, men cases 83% controls 79%, age 19-80y | FFQ (open answer on frequency (63 items, measured period last 20 years, conducted once, validated) | food content tables provided by the Swedish Food Agency | flavonoids, anthocyanidins, isoflavones | N | not adjusted | NR |
| Luo et al., 2015(176) |  | China mainland | 301 subjects, women 100%, age 45-65y | FFQ (134 items, measured period NR, conducted once, validated) | published | phtyoestrogens: isoflavones, genistein, daidzein, glycitein, lignans, matairesinol, secoisolariciresinol, enterolactone, enterodiol, coumestrol | Y | not adjusted | NR |
| Ma et al., 2015(177) |  | China mainland | 249 cases and 66 controls subjects, men cases 41% controls 46 %, age 50-70y | FFQ (139 items, measured period NR, conducted twice, validated) | USDA | flavonoids: anthocyanidins flavan-3-ols flavanones flavones flavonols and flavanoids | N | adjusted for energy intake | NR |
| Merida-Ortega et al., 2016(178) |  | Mexico | 233 cases and 221 controls subjects, women 100%, age 18+y | FFQ (119 items, measured period last year, conducted once, validated) | USDA | flavonoids: anthocyanidins, flavanols, flavanones, flavones, flavonols | Y | not adjusted | NR |
| Nagata et al., 2002(179) |  | Japan | 87 subjects, postmenopausal women 100%, age 38-68y | FFQ (169 items, measured period last year, conducted once, validated) | published | soy isoflavones: genistein, daidzein, equol | N | adjusted for energy intake | serum: serum dadizein and equol, NR |
| Nagata et al., 2003(180) |  | Japan | 201 subjects, postmenopausal women 100%, age 37-47y | FFQ (169 items, measured period last year, conducted once, validated) | published | soy isoflavones | Y | adjusted for energy intake | NR |
| Nagata et al., 2007(181) |  | Japan | 200 cases and 200 controls subjects, men 100%, age 59-73y | FFQ (169+12 soy food items, measured period 5 years before diagnosis/interview, conducted once, validated) | Japanese food composition tables | isoflavones: genistein, daidzein | N | not adjusted | NR |
| Nagata et al., 2008(182) |  | Japan | 419 subjects, women 100%, age 40-63y | FFQ (169 items, measured period last year, conducted once, validated) | published | soy isoflavones: equol, daidzein | Y | adjusted for energy intake | Spot urine: isoflavonoids, NR |
| Nagata et al., 2009(183) |  | Japan | 285 subjects, women 100%, age 23-56y | FFQ (169 items, measured period last year, conducted once, validated) | Japanese food composition tables | soy isoflavones | Y | adjusted for energy intake | NR |
| Nagata et al., 2016(184) |  | Japan | 56 cases and 56 controls subjects, men 100%, age cases 58-71, controls 55-73y | FFQ (measured period last year, repeated once, validated) | published | isoflavones: genistein, daidzein | Y | not adjusted | serum: serum genistein, daidzein, glycitein, and equol, serum vs. dietary intake for genistein, daidzein were not significantly correlated |
| Nakamoto et al., 2018(185) |  | Japan | 821 subjects, men 67%, age 20-60y | FFQx2 (for soy food consumption/ for energy intake) (12+29 items, measured period Soy FFQ: NR, FFQ: last month, conducted once, validated) | published | isoflavones: daidzein, genistein | N | not adjusted | NR |
| Nguyen et al., 2017(186) |  | Vietnam | 599 cases and 599 controls subjects, NR, age 40-65y | FFQ (128 items, measured period last year, conducted once, validated) | USDA | isoflavones: daidzein, genistein, glycitein | N | adjusted for energy intake | NR |
| Oh et al., 2016(187) |  | Korea | 502 subjects, women 100%, age 30+y | FFQ (144 items, measured period last year, conducted once, validated) | USDA+ Japan Functional Food Factor Database, Rural Development Administration Food Functional Composition Table | flavanones: naringenin, hesperetin and eriodictyol | N | not adjusted | NR |
| Ohfuji et al., 2014(188) |  | Japan | 126 cases and 170 controls subjects, men cases 58% controls 52%, age <80y | FFQ (150 items, measured period last month, conducted once, validated) | Japanese food composition tables | isoflavones: daidzein, genistein | N | adjusted for energy intake | NR |
| Ponzo et al., 2015(189) |  | Italy | 1658 subjects, men 48%, age 45-64y | FFQ (148 items, measured period last year, conducted once, validated) | USDA | flavonoids: flavanones, flavones, flavonols, flavan-3-ols, anthocyanidins, isoflavones and proanthocyanidins | N | adjusted for energy intake | NR |
| Quiller et al., 2018(190) |  | Mexico | 1027 subjects, women 100%, age 20+y | FFQ (119 items, measured period last year, conducted once, validated) | USDA | flavonoids: anthocyanidins, flavanols, flavanones, flavones, flavonols | N | not adjusted | NR |
| Ranka et al., 2008(191) |  | UK | 49 subjects, men 43%, age 40-85y | FFQ (159 items, measured period last year, conducted once, validated) | USDA | quercetin and naringenin | N | not adjusted | 24-hour urine: quercetin and naringenin metabolites, dietary quercetin vs. urinary excretion (r =0.827, 95% CI: 0.712–0.899), naringenin (r =0.251, 95% CI: 􏲝0.032 to 0.497). |
| Reale et al., 2018(192) |  | Italy | 118 cases and 222 controls subjects, men 100%, age mean cases 69, controls 68y | FFQ (110 items, measured period last year, conducted once, validated) | Phenol-Explorer | flavonoids: anthocyanidins, flavanols, flavanones, flavones, flavonols, catechins | N | not adjusted | NR |
| Reed et al., 2013(193) |  | USA | 5634 subjects, women 100%, age 45-58y | FFQ (specific for soy) (20 soy food items, measured period last 3 months, conducted once, validated) | not reported | isoflavones: genistein, daidzein | Y | not adjusted | NR |
| Ronco et al., 2016(194) |  | Uruguay | 572 cases and 889 controls subjects, women 100%, age <85y | FFQ (64 items, measured period last year, conducted once, not validated) | published | flavonols | N | not adjusted | NR |
| Rosli et al., 2019(195) |  | Malaysia | (394 woth total phenolic intake < 221 mg/day and 395 > 221 mg/day) subjects, men 53%, age 40-70 y | FFQ (117 items, measured period last year, conducted once, validated) | Phenol-Explorer | total polyphenols | Y | not adjusted | NR |
| Rossi et al., 2006(196) |  | Italy | 1953 cases and 4154 controls subjects, men cases 58% contorls 50%, age 19-74y | FFQ (78 items, measured period last 2 years, conducted once, validated) | USDA | flavonoids: anthocyanidins, flavan-3-ols, flavanones, flavones, flavanols, isoflavones | N | adjusted for energy intake | NR |
| Rossi et al., 2007(197) |  | Italy | 304 cases and 743 controls subjects, men cases 90% controls 80%, age 36-77y | FFQ (78 items, measured period last 2 years, conducted once, validated) | USDA | flavonoids: anthocyanidins, flavan-3-ols, flavanones, flavones, flavanols | N | not adjusted | NR |
| Rossi et al., 2007(198) |  | Italy | 805 cases and 2081 controls subjects, men cases 82% controls 63%, age 19-79y | FFQ (78 items, measured period last 2 years, conducted once, validated) | USDA | flavonoids: anthocyanidins, flavan-3-ols, flavanones, flavones, flavanols, isoflavones | N | not adjusted | NR |
| Rossi et al., 2008(199) |  | Italy | 1031 cases and 2411 controls subjects, women 100%, age 18-79y | FFQ (78 items, measured period last 2 years, conducted once, validated) | USDA | flavonoids: anthocyanidins, flavan-3-ols, flavanones, flavones, flavanols, isoflavones | N | adjusted for energy intake | NR |
| Rossi et al., 2010(200) |  | Italy | 1953 cases and 4154 controls subjects, men cases 58% controls 50%, age 19-74y | FFQ (83 items, measured period last 2 years, conducted once, validated) | USDA | proanthocyanidins: monomers, dimers, trimers, 4-6, 7-10, >10) | N | adjusted for energy intake | NR |
| Rossi et al., 2010(201) |  | Italy | 230 cases and 547 controls subjects, men cases 62% controls 52%, age 22-80y | FFQ (78 items, measured period last 2 years, conducted once, validated) | USDA | flavonoids, proanthocyanidins: anthocyanidins, flavan-3-ols, flavanones, flavones, flavanols, isoflavones, proanthocyanidin monomers, dimers, trimers, 4–6 mers, 7–10 mers, >10 mers | N | adjusted for energy intake | NR |
| Rossi et al., 2012(202) |  | Italy | 326 cases and 652 controls subjects, men cases and controls 53%, age 34-80y | FFQ (78 items, measured period last 2 years, conducted once, validated) | USDA | flavonoids: anthocyanidins, flavan-3-ols, flavanones, flavones, flavanols | N | adjusted for energy intake | NR |
| Rossi et al., 2013(203) |  | Italy | 454 cases and 908 controls subjects, women 100%, age cases 18-79, controls 19-80y | FFQ (83 items, measured period last 2 years, conducted once, validated) | mixed source | flavonoids: anthocyanidins, flavan-3-ols, flavanones, flavones, flavanols, isoflavones | N | adjusted for energy intake | NR |
| Rossi et al., 2019(204) |  | Italy | 172 subjects, men 48%, age 8-10y | FFQ (95 items, measured period NR, conducted once, validated) | USDA | flavanoids: isoflavones, anthocyanidins, flavan 3 ols, flavanones, flavones. flavanols. | N | adjusted for energy intake | NR |
| Russo et al., 2017(205) |  | Italy | 118 cases and 222 controls subjects, men 100%, age mean cases 69, controls 68y | FFQx2 (110 items, measured period last year, conducted once, validated) | Phenol-Explorer | phenolic acids: hydroxybenzoic acids, hydroxycinnamic acids, hydroxyphenylacetic acids | N | adjusted for energy intake | NR |
| Russo et al., 2018(206) |  | Italy | 118 cases and 222 controls subjects, men 100%, age mean cases 69, controls 68y | FFQx2 (110 items, measured period last year, conducted once, validated) | Phenol-Explorer | phytoestrogens: lignans. isoflavones, lariciresinol, matairesinol, pinoresinol, seco, daidzein, genistein, glycitein, biochanin A | N | adjusted for energy intake | NR |
| Salomone et al., 2020(207) |  | Israel | 9374 subjects, men 46%, age 1.5+y | FFQ (117 items, measured period last year, conducted once, not validated) | Data on the poly-phenol content in foods was obtained from the Phenol-Explorerdatabase (www.phenol-explorer.eu) | phenolic acids, : hydrobenzoic acids, hydroxycinnamic acid | N | not adjusted | NR |
| Schabath et al., 2005(208) |  | USA | 1674 cases and 1735 controls subjects, men cases 54% controls 51%, age mean cases 62.1, controls 61.5y | FFQ (measured period 1 year before diagnosis/ interview, repeated once, validated) | USDA | phytoestrogens: phytosterols, isoflavones, lignans precursors and lignans metabolites | N | not adjusted | NR |
| Seow et al., 2002(209) |  | Singapore | 303 cases and 765 controls subjects, women 100%, age 20-89y | FFQ (39 items, measured period last 3 years, conducted once, validated) | Singapore Food Composition Database | isoflavones | N | not adjusted | NR |
| Shin et al., 2015(210) |  | Korea | 901 cases and 2669 controls subjects, men cases 69% controls 70%, age NR | FFQ (106 items, measured period NR, conducted once, validated) | published | isoflavones: daidzein, genistein, glycitein | N | adjusted for energy intake | NR |
| Silva et al., 2004(211) |  | UK | 240 cases and 477 controls subjects, women 100%, age <75y | FFQ (207 items, measured period 2-3 years before diagnosis, conducted once, validated) | published+unpublished | phytoestrogens: genistein, daidzein, seco and mata | N | not adjusted | NR |
| Sonoda et al., 2010(212) |  | Japan | 180 cases and 177 controls subjects, men 100%, age 46-81y | FFQ (169+12soy items, measured period 5 years before diagnosis/interview, conducted once, validated) | Japanese food composition tables | isoflavones | N | not adjusted | NR |
| Strom et al., 1999(213) |  | USA | 83 cases and 107 controls. subjects, men 100%, age mean cases 61, controls 60.6y | FFQ (measured period 1 year before diagnosis/ interview, repeated once, not validated) | DietSys | phytoestrogens: isoflavones, flavonoids, lignan precursors, and phytosterols | N | not adjusted | NR |
| Sut et al., 2020(214) |  | Poland | 475 subjects, NR, age 33-77y | FFQ (measured period NR, repeated once, validated) | Phenol-Explorer | all classes: phenolic compounds, flavonoids (alkylphenols, flavones, flavanols, catechins, procyanidins, anthocyanins, theaflavins, dihydrochalcones, isoflavonoids), flavan-3-ols, phenolic acids (hydroxybenzoic acid, hydroxycinnamic acid), stilbenes, and lignans. | N | not adjusted | NR |
| Taborelli et al., 2017(215) |  | Italy | 777 subjects, men 100%, age 46–74y | FFQ  (78 items, measured period last 2 years, conducted once, validated) | USDA | flavonoids , proanthocyanidins | N | adjusted for energy intake | NR |
| Takata et al., 2004(216) |  | Japan, Hawaii | 516 subjects, women 100%, age 0 Japanese in Gifu 50.0, Japanese in Hawaii 52.3, Caucasians in Hawaii 53.7y | FFQ (169 Japan/188 Hawaii and  +10 soy food items, measured period NR, conducted once, not validated) | mixed source | flavonoids: isoflavones | N | adjusted for energy intake | NR |
| Tang et al., 2015(217) |  | China mainland | 359 cases and 380 controls subjects, men case 72% control 71%, age mean 61y | FFQ (137 items, measured period last 5 years, conducted once, validated) | USDA | isoflavones : daidzein, genistein and glycitein | N | not adjusted | NR |
| Tavani et al., 2006(218) |  | Italy | 760 cases and 682 controls subjects, men cases 76% controls 64%, age 16-79y | FFQ (78 items, measured period NR, conducted once, validated) | USDA+published | flavonoids: isoflavones, anthocyanidins, flavan-3-ols, flavanones, flavones and flavonols) | N | not adjusted | NR |
| Toi et al., 2013(219) |  | Japan | 306 cases and 662 controls subjects, women 100%, age 40-55y | FFQ (168 items, measured period last year, conducted once, validated) | Japanese food composition tables | flavonoids: isoflavones | Y | not adjusted | NR |
| Torres-Sanchez et al., 2009(220) |  | Mexico | 141 cases and 141 controls subjects, women 100%, age 21-79y | FFQ  (measured period Last year, repeated once, validated) | analysed by colleague | flavanoids: flavonols, flavan3-ols, flavones, cinnamic acid, lignans and coumestrol, | N | not adjusted | NR |
| Tseng et al., 2008(221) |  | USA | 451 subjects, women 100%, age 30-50y | SFQ, FFQ (measured period Last year, repeated once, validated) | published | isoflavones: dadzein, genistein | Y | not adjusted | urine: isoflavones (daidzein, genistein, glycitein) and 2 metabolites of daidzein [O-desmethylangolensin (ODMA) and equol], 24h sample measures were correlated with SQF daidzein/genistein 0.48/0.54, DAF0.38/0.33, first spot urine: 0.84/0.93 (0.61equol-0.93genistein) |
| Walcott et al., 2002(222) |  | USA | 187 cases and 148 controls subjects, men 100%, age 18-55y | FFQ (152 items, measured period last year, conducted once, validated) | published | phytoestrogens: prelignans, lignans, flavonoids, isoflavonoids, phytosterols, and coumestrol | N | adjusted for energy intake | NR |
| Wang et al., 2010(223) |  | China mainland | 176 cases and 176 controls subjects, women 100%, age 40-65y | FFQ (3 soy food items, measured period NR, conducted once, not validated) | published | isoflavones | N | not adjusted | NR |
| Wang et al., 2020(224) |  | USA | 1577 controls and 1472 BC cases, subjects, women 100%, age 20 to 85y | FFQ (measured period NR, repeated once, not validated) | Diet Calc Analysis Software version 1.5.0 | phytoestrogens: genistein ,daidzein , biochanin A , formononetin, glycitein, coumestrol | N | not adjusted | NR |
| Wong et al., 2007(225) |  | Hong Kong | 2000 subjects, men 100%, age 65+y | FFQ (10 soy food items, measured period NR, conducted once, not validated) | published | isoflavones : daidzein, genistein and glycitein | N | adjusted for energy intake | NR |
| Woo et al., 2006(226) |  | Hong Kong | 3999 subjects, men 56%, age 65+y | FFQ (269 items, measured period last week, conducted once, validated) | Chinese food composition table | isoflavones | N | not adjusted | NR |
| Woo et al., 2014(227) |  | Korea | 334 cases and 334 controls subjects, men cases and controls 62%, age 35–75y | FFQ  (103 items, measured period last year, conducted once, validated) | USDA | flavonoids: flavonols, flavones, flavanones, flavan-3-ols, anthocyanidins, and isoflavones | N | adjusted for energy intake | NR |
| Wu et al., 2002(228) |  | USA | 501 cases and 595 controls subjects, women 100%, age 25-74y | FFQ (14 soy food items, measured period MEC: last year, EPIC: NR, conducted once, validated) | Hawaii food composition database | isoflavones: isoflavones (daidzein, glycestein and genistein) | N | adjusted for energy intake | NR |
| Wu et al., 2004(229) |  | USA | 97 cases and 97 contorls subjects, women 100%, age 25-74y | FFQ (14 soy food items, measured period MEC: last year, EPIC: NR, conducted once, validated) | Hawaii food composition database | isoflavones : daidzein, genistein, equol, dihydrodaidzein and dihydrogenistein | Y | not adjusted | plasma: plasma levels of isoflavones: genistein, daidzein, equol, dihydrodaidzein and dihydrogenistein), NR |
| Xu et al., 2004(230) |  | China mainland | 832 cases and 846 control subjects, women 100%, age 30-69y | FFQ (17 soy food items, measured period last 5 years, conducted once, validated) | Chinese food composition table | soy isoflavones | N | not adjusted | NR |
| Xu et al., 2016(231) |  | China mainland | 1632 cases and 1632 controls subjects, men case colon cancer 58% /rectal cancer 53% control 56%, age mean cases 56.5, controls 56.3y | FFQ (81 items, measured period last year, conducted once, validated) | USDA+isoflavones for chinese food composition table | flavonoids: (anthocyanidins, flavan3-ols, flavanones, flavones, flavonols, theaflavins and thearubigins, and proanthocyanidins) | Y | adjusted for energy intake | NR |
| Yang et al., 2018(232) |  | Japan | 1426 subjects, men 74%, age 20-64y | FFQx2 (for soy food consumption/ for energy intake) (12+29 items, measured period Soy FFQ: NR, FFQ: last month, conducted once, validated) | Japanese food composition tables | flavonoids: soy isofavone | N | adjusted for energy intake | NR |
| Yochum et al., 1999(233) |  | USA | 34492 subjects, women 100%, age 55-69 y | FFQ  (127 items, measured period NR, conducted once, not validated) | published | flavonoids : (quercetin, kaempferol, and myricetin) and  flavones (luteolin and apigenin) | N | not adjusted | NR |
| Zhang et al., 2004(234) |  | China mainland | 254 cases and 652 controls subjects, women 100%, age <75y | FFQ (128+9 soy food items, measured period last year, conducted once, validated) | USDA | flavonoids: isofiavones | N | not adjusted | NR |
| Zhang et al., 2009(235) |  | China mainland | 1009 subjects, women 100%, age 20-87y | FFQ (100 items, measured period last year, conducted once, validated) | USDA | isoflavones: daidzein, genistein, glycitein | N | not adjusted | NR |
| Zhang et al., 2010(236) |  | China mainland | 438 cases and 438 controls subjects, women 100%, age 25-70 y | FFQ (81 items, measured period last year, conducted once, validated) | Chinese food composition table | isoflavones | N | not adjusted | NR |
| Zhang et al., 2010(237) |  | China mainland | 756 subjects, women 100%, age 20-87y | FFQ (100 items, measured period 1 year before diagnosis, conducted once, validated) | USDA | isoflavones: daidzein, genistein, glycitein | N | not adjusted | NR |
| Zhang et al., 2012(238) |  | China mainland | 616 subjects, women 100%, age mean 45.7y | FFQ (6 soy food items, measured period NR, conducted once, not validated) | Chinese food composition table | isoflavones: daidzein, genistein and glycitein | N | not adjusted | NR |
| Zhang et al., 2014(239) |  | China mainland | 3317 subjects, men 68%, age 56-67y | FFQ (79 items, measured period NR, conducted once, validated) | USDA +Hong Kong database of isoflavones | flavonoids: flavanones, anthocyanins, flavan-3-ols, flavonols, flavones, and isoflavonones, proanthocyanidin | N | adjusted for energy intake | NR |
| Zhu et al., 2011(240) |  | China mainland | 183 cases and 192 controls subjects, women 100%, age mean 45.7y | FFQ (84 items, measured period last year, conducted once, not validated) | Chinese food composition table | isoflavones | N | not adjusted | NR |
| Woo et al., 2018(241) | Korean Multi-Rural Communities Cohort Study (MRCohort) | Korea | 5509 subjects, men 40%, age 40+y | FFQ (106 items, measured period NR, conducted twice, validated) | USDA +korean database | isoflavones | N | adjusted for energy intake | NR |
| Burkholder-Cooley et al., 2016(242) | Adventist Health Study­2 (AHS­2) | USA, Canada | 77441 subjects, men 35%, age mean 57.1y | FFQ (204 items, measured period NR, conducted once, validated) | mixed source | all classes: flavonoids, phenolic acids, stilbenes, lignans and other phenolics | N | not adjusted | NR |
| Burkholder-Cooley et al., 2017(243) | Adventist Health Study­2 (AHS­2) | USA, Canada | 1011 subjects, men 33%, age mean 58.2y | FFQ (204 items, measured period NR, conducted once, validated) | mixed source | total polyphenol | Y | not adjusted | urine: total polyphenols, 24h recall vs. total urinary polyphenols (r=0.15, P<0.001) |
| Fraser et al., 2016(244) | Adventist Health Study­2 (AHS­2) | USA, Canada | 96116 subjects, men 37%, age mean non-black 60.2, black 53.8y | FFQ (204 items, measured period last year, conducted once, validated) | USDA+Nutrition Data System for Research | isoflavones: genistein, daidzein | Y | not adjusted | urine: daidzein and genistein, soy isoflavones intake from FFQ vs. urinary isoflavones (daidzein/genistein) male 0.28/0.24, female 0.40/0.42 |
| Jacobsen et al., 2014(245) | Adventist Health Study­2 (AHS­2) | Canada | 96000 subjects, women 100%, age 30-50y | FFQ (50 items, measured period NR , conducted once, validated) | Nutrient Data System for Research | isoflavones | N | not adjusted | NR |
| Tan et al., 2020(246) | Adventist Health Study-2 (AHS-2) and Biopsychosocial Religion and Health Cohort studies (BRHS) | USA, Canada | 6633 subjects, men 52% , age men 55-57 women 60-75y | FFQ (200 items, measured period last year, conducted once, validated) | mixed source | flavonoids | N | adjusted for energy intake | NR |
| Nechuta et al., 2012(247) | After Breast Cancer Pooling Project (ABCPP) | USA, China | 9514 subjects, women 100%, age mean 53.9y | FFQ ( 95+14 items, measured period habitual intake after diagnosis, conducted once, validated) | USDA + chinese food composition table | isoflavones | N | not adjusted | NR |
| Root et al., 2015(248) | ARIC study | USA | 10041 subjects, men 45%, age 45-64y | FFQ (66 items, measured period NR, conducted once, not validated) | Willett's nutritional database | flavonols: myricetin, kaempferol, and quercetin | N | not adjusted | NR |
| Bobe et al., 2008(249) | A-Tocopherol, B-Carotene Cancer Prevention Study | Finland | 29133 subjects, men 100%, age 50-69y | FFQ (276 items, measured period last year, conducted once, validated) | published | flavonoids: kaempferol, myricetin, quercetin, apigenin, luteolin, catechin, and epicatechin | Y | adjusted for energy intake | NR |
| Wright et al., 2004(250) | A-Tocopherol, B-Carotene Cancer Prevention Study | Finland | 27111 subjects, men 100%, age 50–69 y | FFQ (276 items, measured period last year, conducted once, validated) | published | flavonoids: kaempferol, myricetin, quercetin, catechin, and epicatechin | Y | not adjusted | NR |
| Lahmann et al., 2012(251) | Australian Ovarian Cancer Study (AOCS, 2002–5), the Australian National Endometrial Cancer Study (ANECS, 2005–7) | Australia | 2078 subjects, women 100%, age 18-79y | FFQ (135 items, measured period last year, conducted once, validated) | published | phytoeostrogens/ isoflavones, lignans, enterolignans, coumestrol: daidzein, genistein, glycitein, biochanin A, formonometin, secoisolariciresinol, matairesinol pinoresinol, laricirsinol, enterolactone, enterodiol, equol, | N | adjusted for energy intake | NR |
| Neill et al., 2014(252) | Australian Ovarian Cancer Study (AOCS, 2002–5), the Australian National Endometrial Cancer Study (ANECS, 2005–7) | Australia | AOCS: 1366 cases and 1414 controls/ ANECS: 1288 and 1435 controls subjects, women 100%, age 18-79y | FFQ (135 items, measured period 1 year before diagnosis/ interview, conducted once, validated) | published | phytoestrogens : isoflavones, lignans, enterolignans, coumesterol | N | adjusted for energy intake | NR |
| Bondonno et al., 2020(253) | Blue Mountains Eye Study (BMES) | Australia | 2349 subjects, men 41%, age 49-97y | FFQ (145 items, measured period last year, conducted once, validated) | USDA | flavonoids: flavonols, flavones, flavanones, flavan-3-ols, isoflavones, anthocyanidins, and proanthocyanidins | N | not adjusted | NR |
| Gopinath et al., 2018(254) | Blue Mountains Eye Study (BMES) | Australia | 2856 subjects, men 44%, age 49+y | FFQ (145 items, measured period last year, conducted once, validated) | USDA | flavonoids: flavonols, flavan-3-ols, flavones, flavanones, anthocyanins, and isoflavones | N | adjusted for energy intake | NR |
| Zhang et al., 2017(255) | Breast Cancer Family Registry | USA, Australia | 4769 subjects, women 100%, age mean 51.8y | FFQ (108 items, measured period NR, conducted once, validated) | USDA | isoflavones: genistein, daidzein, and glycitein | N | not adjusted | NR |
| Ivey et al., 2013(256) | Calcium Intake Fracture Outcome Age Related Extension Study | Australia | 1063 subjects, women 100%, age 75+y | FFQ (121 items, measured period last year, conducted once, validated) | USDA | flavonoids: flavonol, flavan-3-ol, proanthocyanidin flavone, flavanone, anthocyanidin, isoflavone | N | not adjusted | NR |
| Ivey et al., 2015(257) | Calcium Intake Fracture Outcome Age Related Extension Study | Australia | 1136 subjects, NR, age 75+y | FFQ (measured period last year, repeated once, validated) | USDA+ Phenol-Explorer | flavonoids: flavonol, flavanone,flavone,anthocyanidin, isoflavones | N | not adjusted | NR |
| Myers et al., 2015(258) | Calcium Intake Fracture Outcome Age Related Extension Study | Australia | 1188 subjects, women 100%, age 75+y | FFQ (measured period NR, repeated once, validated) | mixed source | flavonoids: flavonols, flavan3-ols, proanthocyanidins, anthocyanins, flavanones, flavones, and isoflavones | N | not adjusted | NR |
| Chang et al., 2007(259) | California Teachers Study (CTS) | USA | 97275 subjects, women 100%, age <85y | FFQ (103 items, measured period last year, conducted once, validated) | published | isoflavones: genistein and daidzein | N | adjusted for energy intake | NR |
| Chang et al., 2011(260) | California Teachers Study (CTS) | USA | 110215 subjects, women 100%, age 20-84y | FFQ (103 items, measured period last year, conducted once, validated) | published | isoflavones, lignans | N | not adjusted | NR |
| Horn-Ross et al., 2002(261) | California Teachers Study (CTS) | USA | 111526 subjects, women 100%, age 21-103y | FFQ (103 items, measured period last year, conducted once, validated) | published | phytoestrogens: genistein, diadzien, biochanin, formononetin, coumetrol, matairesinol, secoisolariciresiniol | N | not adjusted | NR |
| Wang et al., 2014(262) | Cancer Prevention Study II (CPS-II) Nutrition Cohort | USA | 56630 subjects, women 100%, age mean 68.6y | FFQ (152 items, measured period last year, conducted once, validated) | USDA | flavonoids: anthocyanidins, flavones, flavanones, flavan-3-ols, flavonols, isoflavones, and proanthocyanidins | N | adjusted for energy intake | NR |
| Hedelin et al., 2006(263) | Cancer Prostate in Sweden (CAPS) | Sweden | 1499 cases and 1130 controls subjects, men 100%, age 35-79y | FFQ (261 items, measured period last year, conducted once, validated) | published | phytoestrogens: genistein, daidzein, biochanin A, formononetin, coumestrol, mat and seco; isolariciresinol, lariciresinol, pinoresinol, syringaresinol and medioresinol | N | not adjusted | serum: serum enterolactone, no significant correlation dietary intake of total lignans vs. serum enterolactone (r = 0.13, p = 0.09). Among those with low animal fat intake serum enterolactone vs. dietary intake of total lignans (r= 0.24, p= 0.03) |
| Ilow et al., 2008(264) | Cardiovascular Disease Prevention Program | Poland | 203 subjects, men 40%, age mean 50y | FFQ (39 items, measured period last 3 months, conducted once, not validated) | USDA | flavonoids: flavanones, flavones, flavonols and anthocyanidins | N | not adjusted | NR |
| Akhter et al., 2009(265) | Colorectal Adenoma Study in Tokyo (CAST) | Japan | 721 cases and 697 controls subjects, men cases 68% controls 66%, age 40-79y | FFQ (145 items, measured period NR, conducted once, not validated) | Japanese food composition tables | isoflavones: genistein and daidzein | N | adjusted for energy intake | NR |
| Richardson et al., 2016(266) | Coronary Artery Risk Development in Young Adults (CARDIA) study | USA | 3142 subjects, men 43% , age 18-30y | FFQ (100 items, measured period NR, conducted once, validated) | USDA | isoflavones: daidzein, genistein, glycitein | N | not adjusted | NR |
| McCullough et al., 2012(267) | CPS-II Nutrition Cohort | USA | 98479 subjects, men 38%, age mean men 70, women 69y | FFQ (52 items, measured period last year, conducted every year, validated) | USDA | flavonoids: anthocyanidins, flavan-3-ols, flavanones, flavones, flavonols, proanthocyanidins, isoflavones | N | adjusted for energy intake | NR |
| Wang et al., 2014(268) | CPS-II Nutrition Cohort | USA | 43268 subjects, men 100%, age 50–74y | FFQ (152 items, measured period last year, conducted every year, not validated) | USDA | flavanoids: anthocyanidins, flavones, flavanones, flavan-3-ols, flavonols, isoflavones, and proanthocyanidins. proanthocyanidins were the sum of monomers, dimers, trimers, 4–6 mers, 7–10 mers, and >10 mers | N | adjusted for energy intake | NR |
| Bondonno et al., 2019(269) | Danish Diet, Cancer and Health Study | Denmark | 56048 subjects, men 48%, age 50-65y | FFQ (192 items, measured period last year, conducted once, validated) | Phenol-Explorer | flavonoids: flavonols, flavanol monomers, flavanol oligo + polymers, flavanones, flavones, anthocyanins, isoflavones, dihydrochalcones, dihydroflavonols and chalcones | N | not adjusted | NR |
| Bondonno et al., 2020(270) | Danish Diet, Cancer and Health Study | Denmark | 55631 subjects, men 47%, age 50-65y | FFQ (192 items, measured period last year, conducted once, validated) | Phenol-Explorer | flavonoids: flavonols, flavones, flavanols (flavanol monomers and flavanol oligo þ polymers), flavanones, isoflavones, anthocyanins, chalcones, dihydrochalcones and dihydroflavonols | N | not adjusted | NR |
| Dalgaard et al., 2019(271) | Danish Diet, Cancer and Health Study | Denmark | 53552 subjects, men 47%, age 50-65y | FFQ (192 items, measured period last year, conducted once, validated) | Phenol-Explorer | flavonoids: flavonols, flavones, flavanols (flavanol monomers and flavanol oligo þ polymers), flavanones, isoflavones, anthocyanins, chalcones, dihydrochalcones and dihydroflavonols | N | not adjusted | NR |
| Swann et al., 2013(272) | DietCompLyf study | UK | 3159 subjects, women 100%, age mean 54.4y | FFQ (130 items, measured period last year/last week, conducted once, validated) | database built in CAFÉ programme | phytoestrogens: genistein, daidzein, glycitein, biochanin A, formononetin, secoisolariciresinol, shonanin, matairesinol, coumestrol, enterolactone, equol, enterodiol | N | not adjusted | NR |
| Nooyens et al., 2015(273) | Doetinchem Cohort Study (prospective part of MORGREN study) | Netherlands | 2613 subjects, men 49%, age 43-70y | FFQ (178 items, measured period last year, conducted at baseline and repeated after follow up (5y), validated) | published | flavonoids and lignans: flavonoids: flavonols, flavones, catchins // lignans: lari, pico, seco, mat | N | not adjusted | NR |
| Siedlinski et al., 2012(274) | Doetinchem Cohort Study (prospective part of MORGREN study) | Netherlands | 3224 subjects, men 48%, age 30+y | FFQ (178 items, measured period NR, conducted once, validated) | published | resveratrol | N | not adjusted | NR |
| Lajous et al., 2016(275) | E3N | france | 40574 subjects, women 100%, age 45–58y | FFQ (208 items, measured period NR, conducted once, validated) | Phenol-Explorer | flavonoids: flavonols, flavones, flavanones, anthocyanins, flavanol monomers and flavonoid oligomers and polymers | N | adjusted for energy intake | NR |
| Touillaud et al., 2007(276) | E3N | France | 58049 subjects, women 100%, age 45–58y | FFQ (208 items, measured period NR, conducted once, validated) | published | lignans: pinoresinol, lariciresinol, secoisolariciresinol, and matairesinol | N | adjusted for energy intake | NR |
| Minguez-Alaron et al., 2015(277) | EARTH Study | USA | 182 subjects, men 100%, age 18-51y | FFQx2 (131+15 soy food items, measured period last 3 months, conducted once, validated) | USDA | isoflavones: genistein, daidzein | Y | not adjusted | NR |
| Vanegas et al., 2015(278) | EARTH Study | USA | 315 subjects, women 100%, age 18-46y | FFQx2 (15+131 items, measured period last year, conducted once, validated) | USDA | isoflavones | N | not adjusted | NR |
| Bandera et al., 2009(279) | EDGE Study | USA | 424 cases and 389 controls subjects, women 100%, age 21+y | FFQ (110+22 items, measured period last 7 months, conducted once, not validated) | USDA | phytoestrogens: daidzein, genistein, formononetin, glycitein/ matairesinol, lariciresinol. pinoresinol. secoisolariciresinol.coumestrol.quecetin, total phytoestrogens | N | not adjusted | NR |
| Dilis et al., 2010(280) | EPIC-Greek | Greece | 28572 subjects, men 42%, age median men 51, women 54y | FFQ (215 items, measured period last year, conducted once, validated) | USDA | flavonoids, proanthocyanidins: flavones,flavonoles, flavanones, flavan-3-ols, anthocyanidins, isoflavones, proanthocyanidins | N | not adjusted | NR |
| Lu et al., 2017(281) | EPIC-IBD | Europe | 354 cases and 1416 controls subjects, men Chrons disease (CD) cases 27%, controls 24%/ ulcerative colitis (UC) cases: 43 % controls 42%, age 20-80y | FFQ (200 items, measured period NR , conducted once, validated) | Phenol-Explorer | total polyphenols: flavanoids, anthocyanins, flavanols, flavanones, flavones, isoflavones, lignans, stilbenes, phenolic acids | N | not adjusted | NR |
| Boker et al., 2002(282) | EPIC-Netherlands | Netherlands | 17140 subjects, women 100%, age 49-70y | FFQ (227 items, measured period last year, conducted once, validated) | published score | phytoestrogens: isoflavones daidzein, genistein, formononetin, biochanin A, the coumestan coumestrol and the lignans matairesinol and secoisolariciresinol | N | not adjusted | NR |
| Keinan-Boker et al., 2004(283) | EPIC-Netherlands | Netherlands | 17357 subjects, women 100%, age 50-69y | FFQ (178 items, measured period last year, conducted once, validated) | published score | phytoestrogens: daidzein genistein formononetin biochanin A enterolactone enterodiol isoflavones lignans | N | not adjusted | NR |
| Kreijkamp-Kaspers et al., 2007(284) | EPIC-Netherlands | Netherlands, Denmark | 301 subjects, women 100%, age 60-75y | FFQ (measured period last year, repeated once, validated) | mixed source | isoflavones, lignans | N | adjusted for energy intake | NR |
| Van Der Schouw et al., 2005(285) | EPIC-Netherlands | Netherlands | 17357 subjects, women 100%, age 49-70y | ffq (178 items, measured period NR, conducted once, validated) | published score | flavonoids: phytoepstrogens | N | adjusted for energy intake | NR |
| Vrieling et al., 2004(286) | EPIC-Netherlands | Netherlands | premenopausal 224 postmenopausal 162 subjects, Women, age 49–69 y | FFQ (178 items, measured period Last year, conducted once, validated) | published | phytoestrogens: daidzein, genistein, formononetin, biochanin A,matairesinol, secoisolariciresinol | N | not adjusted | NR |
| Travis et al., 2008(287) | EPIC-Oxford | UK | 37643 subjects, women 100%, age 20-89y | FFQ (measured period last year, repeated once, validated) | USDA | isoflavones | Y | not adjusted | NR |
| Verkasalo et al., 2001(288) | EPIC-Oxford | UK | 1092 subjects, women 100%, age >20y | FFQ (130 items, measured period last year, conducted once, validated) | USDA | isoflavones | Y | not adjusted | NR |
| Zamora et al., 2013(289) | EPIC-Spain | Spain | 334850 subjects, women 100%, age 35-70 y | FFQ (measured period NR, repeated once, validated) | USDA+ Phenol-Explorer +UK Food Standards Agency database | flavanoids: flavanols,flavan-3-ols, proanthocyanidins,teaflavins,anthocyanidins,flavonols,flavanones,flavones, | N | adjusted for energy intake | NR |
| Jacques et al., 2015(290) | Framingham Heart Study | USA | 2880 subjects, men 45%, age 28-62y | FFQ (measured period last year, repeated 4 times, validated) | USDA | flavonoids: flavonols flavones  flavanones  flavan-3-ols anthocyanins  polymeric flavonoids | N | not adjusted | NR |
| Shishtar et al., 2020(291) | Framingham heart study and offspring study | USA | 329 gastric cancer cases, 2700 controls subjects, men cases 73% control 56%, age 20–85y | FFQ (126 items, measured period last year, conducted once, validated) | USDA | flavonoids: flavonols, flavones, flavanones, flavan-3-ols, anthocyanins, and flavonoid polymers | N | adjusted for age and sex | NR |
| Shishtar et al., 2020(292) | Framingham heart study and offspring study | USA | 227 subjects, men 39%, age 40-59y | FFQ (126 items, measured period last year, conducted up to 4 times, validated) | USDA | flavonoids: flavanols, flavones, flavanones, flavan- 3-ols, anthocyanins and flavonoid polymers | N | adjusted for age and sex | NR |
| Cassidy et al., 2015(293) | Framingham Heart Study Offspring cohort | USA | 2375 subjects, men 45%, age median 61y | FFQ (131 items, measured period last year, conducted once, validated) | USDA+EuroFIR | flavoniods: anthocyanins, flavonols, flavanones, flavan-3-ols, polymers, and flavones | N | adjusted for energy intake | NR |
| de Kleijn et al., 2002(294) | Framingham Heart Study Offspring cohort | USA | 939 subjects, women 100%, age mean 59y | FFQ (130 items, measured period last year, conducted once, validated) | published | isoflavones and lignans: daidzein, genistein,coumestrol, matairesinol, secoisolariciresinol | N | not adjusted | NR |
| Jacques et al., 2013(295) | Framingham Heart Study Offspring cohort | USA | 2915 subjects, men 46%, age mean 54.2y | FFQ (measured period NR, repeated 4 times, validated) | USDA+EuroFIR | flavanoids: flavonols flavones flavanones flavan-3-ols anthocyanins polymeric flavonoids | N | not adjusted | NR |
| Budhathoki et al., 2011(296) | Fukuoka Colorectal Cancer Study | Japan | 816 cases and 815 control subjects, men cases 60% control 62%, age 20-74y | FFQ (148 items, measured period last year, conducted once, validated) | mixed source | isoflavones: isoflavones | Y | adjusted for energy intake | NR |
| Wang et al., 2013(297) | Fukuoka Colorectal Cancer Study | Japan | 840 subjects, men cases 60% controls 62%, age 20-74y | FFQ (148 items, measured period last year, conducted once, validated) | USDA+ Phenol-Explorer | total polyphenols | Y | adjusted for energy intake | NR |
| Garcia-Larsen et al., 2018(298) | GA2LEN screening survey | Europe | 2599 subjects, men 42%, age 15-74y | FFQ (250 items, measured period NR, conducted once, validated) | USDA | flavonoids: flavanones, anthocyanins, flavan-3-ols, flavonols, flavones; and polymers proanthocyanidins, theaflavins, and thearubigins. | N | not adjusted | NR |
| Mattioli et al., 2020(299) | Genes Environment Interaction in Respiratory Diseases (GEIRD) study | Italy | 990: 397 controls and 593 cases subjects, men 49%, age 20-84y | FFQ (measured period NR, repeated once, validated) | USDA | flavonoids: flavanones, anthocyanins, flavan-3-ols, flavonols, flavones, polymers and proanthocyanidins | N | not adjusted | NR |
| Qu et al., 2018(300) | Harbin Cohort Study on Diet, Nutrition, and Chronic Non-Communicable Diseases (HDNNCDS) | China mainland | 2635 cases and 6473 controls subjects, men 36%, age 20-75y | FFQ (103 items, measured period last year, conducted once, validated) | Chinese food composition table | flavonoids: kaempferol, luteolin | N | not adjusted | NR |
| Alhassani et al., 2020(301) | Health Professionals Follow-Up Study | USA | 34490 subjects, men 100%, age 40-75y | FFQ (131 items, measured period last year, conducted 6 times (every 4 years), validated) | USDA | flavonoids: flavonoid polymers, anthocyanins, flavan3-ols, flavanones, flavones, and flavonols | N | not adjusted | NR |
| Cassidy et al., 2016(302) | Health Professionals Follow-Up Study | USA | 43880 subjects, men 100%, age 32-81y | FFQ (131 items, measured period last year, conducted 6 times (every 4 years), validated) | USDA+EuroFIR | flavanones, anthocyanins : eriodictyol, hesperetin, naringenin, cyanidin, delphinidin, malvidin, pelargonidin, petunidin, peonidin | N | not adjusted | NR |
| Cassidy et al., 2016(303) | Health Professionals Follow-Up Study | USA | 25096 subjects, men 100%, age 40-75y | FFQ (131 items, measured period last year, conducted 6 times (every 4 years), validated) | mixed source | flavoniods: flavanones, anthocyanins, flavan-3-ols (monomers), flavonols, flavones, and polymers/oligomers (including proanthocyanidins, theaflavins, and thearubigins). | N | not adjusted | NR |
| Rimm et al., 1996(304) | Health Professionals Follow-Up Study | USA | 34789 subjects, men 100%, age 40-75y | FFQ (131 items, measured period last year, conducted twice, validated) | published | flavonoids: 3 flavonols and 2 flavones | N | adjusted for energy intake | NR |
| van der Schouw et al., 2005(305) | Health Professionals Follow-Up Study | USA | 51529 subjects, men 100%, age 40–75y | FFQ (131 items, measured period last year, conducted every 2 years, validated) | published score | phytoestrogens: daidzein, genistein, formononetin, biochanin A, matairesinol, and secoisolariciresinol | N | adjusted for energy intake | NR |
| Grosso et al., 2017(306) | Health, Alcohol and Psychosocial factors In Eastern Europe (HAPIEE) study | Poland | 8821 subjects, men 49%, age 45-69y | FFQ (148 items, measured period last 3 months, conducted once, validated) | Phenol-Explorer | total polyphenols: all subclasses | N | not adjusted | NR |
| Grosso et al., 2018(307) | Health, Alcohol and Psychosocial factors In Eastern Europe (HAPIEE) study | Poland | 2725 subjects, men 42%, age 45-69y | FFQ (148 items, measured period last 3 months, conducted once, validated) | Phenol-Explorer | total polyphenols: all subclasses | N | not adjusted | NR |
| Lei et al., 2020(308) | Hong Kong NTEC-KWC Breast Cancer Survival Study (HKNKBCSS) | Hong kong | 1462 subjects, women 100%, age mean 52.4 (first follow-up)y | FFQ (109 items, measured period last year, conducted 4 times, validated) | Isofavonoid Content of Hong Kong Soy Foods | isoflavones | N | not adjusted | NR |
| Rabassa et al., 2015(309) | InCHIANTI Study | Italy | 652 subjects, men 45%, age 65+y | FFQ (236 items, measured period last year, conducted once, validated) | USDA+ Phenol-Explorer | all classes: for flavonoids: anthocyanidins, flavonols, flavanones, flavones, flavanols including flavan-3-ol monomers, theaflavins and proanthocyanidins and isoflavones | N | not adjusted | 24-hour urine: total polyphenols, total dietary vs. urinary polyphenols (r=0.131; P<0.001) |
| Rabassa et al., 2015(310) | InCHIANTI Study | Italy | 769 subjects, men 31%, age 65+y | FFQ (measured period last year, repeated repeated at follow up, validated) | mixed source | resveratrol | N | not adjusted | 24-hour urine: total resveratrol, total dietary vs. urinary polyphenols (r=0.713; P<0.0001) |
| Rabassa et al., 2016(311) | InCHIANTI Study | Italy | 368 subjects, men 46%, age 65+y | FFQ (236 items, measured period last year, conducted once, validated) | mixed source | all classes: for flavonoids: anthocyanidins, flavonols, flavanones, flavones, flavanols including flavan-3-ol monomers, theaflavins and proanthocyanidins and isoflavones | N | not adjusted | 24-hour urine: total polyphenols, total dietary vs. urinary polyphenol r=0.140 (P<0.001) |
| Urpi-Sarda et al., 2015(312) | InCHIANTI Study | Italy | 811 subjects, men 45%, age 65+y | FFQ (236 items, measured period last year, conducted once, validated) | mixed source | total polyphenols | N | not adjusted | urine: 24h, NR |
| Zamora-Ros et al., 2011(313) | InCHIANTI Study | Italy | 928 subjects, men 45%, age mean 70y | FFQ  (measured period last year, repeated once, validated) | mixed source | total polyphenols: all subclasses | N | not adjusted | urine: 24h, total polyphenol intake vs. excretions expressed by 24-h volume (r = 0.211; P < 0.001), total polyphenol is not correlated with urinary creatinine-corrected value (r = 0.014; P = 0.692) |
| Zamora-Ros et al., 2013(314) | InCHIANTI Study | Italy | 807 subjects, men 55%, age 65+y | FFQ (measured period last year, repeated y, validated) | mixed source | phenolic acids: phenolic acids, flavonoids (anthocyanidins, flavonols, flavanones, flavones, flavanols, and isoflavones), lignans, stilbenes, and other polyphenols, | N | not adjusted | urine: 24h, NR |
| Nakamoto et al., 2015(315) | Japan Multi-Institutional Collaborative Cohort (J-MICC) | Japan | 1148 subjects, men 49%, age 34-70y | FFQ (47 items, measured period last year, conducted once, validated) | Japanese food composition tables | isoflavones | N | not adjusted | NR |
| Uemura et al., 2018(316) | Japan Multi-Institutional Collaborative Cohort (J-MICC) | Japan | 652 subjects, men 100%, age 35–69 y | FFQ (47 items, measured period last year, conducted once, validated) | Japanese food composition tables | flavonoids: soy isofavone | N | not adjusted | NR |
| Wilunda et al., 2019(317) | Japan Public Health based (JPHC) diabetes study | Japan | 4880 PRESTO women and 2898 SF women subjects, women 100%, age PRESTO 21–45, SF 18–45y | FFQ (from 8 soy food items, measured period last year, conducted 3 times, validated) | published | isofavones: genistein and daidzein | N | adjusted for energy intake | NR |
| Michikawa et al., 2018(318) | JECS (Japan Environment and Children's study) | Japan | 41578 subjects, women 100%, age NR | FFQ (measured period last year, repeated twice, validated) | Japanese food composition tables | isofalvones: genistein | N | not adjusted | NR |
| Michikawa et al., 2019(319) | JECS (Japan Environment and Children's study) | Japan | 41578 subjects, women 100%, age NR | FFQ (10 soy food items, measured period last year, conducted twice, validated) | published | isoflavones: genistein | Y | not adjusted | NR |
| Cao et al., 2017(320) | Jiangsu Nutrition Study cohort | China mainland | 1474 subjects, men 44%, age 20+y | FFQ (3 soy food items, measured period last year, conducted once, validated) | Chinese food composition table | isoflavones: total isoflavones | N | not adjusted | NR |
| Kim et al., 2019(321) | Korean Genome and Epidemiology Study | Korea | 13701: 8599 men (cases: 640 / controls: 7959), 5102 women (cases: 875 / controls: 4227) subjects, men 63%, age 40+y | FFQ (106 items, measured period NR, conducted once, validated) | mixed source | flavonoids: isoflavones, flavan-3-ol, flavanols, flavones | N | not adjusted | NR |
| Guha et al., 2009(322) | LACE study | USA | 1954 subjects, women 100%, age 18-79y | FFQx2 (>100 items, measured period last year, conducted once, validated) | self-analysed | isofalvones: daidzein, genistein, glycetin | N | not adjusted | NR |
| Fink et al., 2006(323) | LIBCSP | USA | 1500 subjects, women 100%, age 20-95y | FFQ (100 items, measured period last year, conducted once, validated) | USDA+published | flavonoids: flavonols, flavones, flavan-3-ols, flavanones, anthocyanidins, isoflavones, and lignans | N | not adjusted | NR |
| Fink et al., 2007(324) | LIBCSP | USA | 1210 subjects, women 100%, age 25-98y | FFQ (100 items, measured period last year, conducted once, validated) | mixed source | flavonoids: flavonols, flavones, flavan-3-ols, flavanones, anthocyanidins, isoflavones, and lignans | N | not adjusted | NR |
| Fink et al., 2007(325) | LIBCSP | USA | 1434 cases and 1440 controls subjects, women 100%, age <65y | FFQ (100 items, measured period last year, conducted once, validated) | mixed source | flavonoids: flavonols, flavones, flavan-3-ols, flavanones, anthocyanidins, isoflavones, and lignans | N | not adjusted | NR |
| Hanna et al., 2010(326) | Longitudinal Assessment of Ageing in Women (LAW) | Australia | 511 subjects, women 100%, age 40-79y | FFQ (112 soy foods items, measured period last month, conducted once, not validated) | published + food manufacturers | phytoestrogens: isoflavones, lignans | N | not adjusted | NR |
| Butchart et al., 2011(327) | Lothian Birth Cohort 1936 | UK | 882 subjects, men 48%, age mean 70y | FFQ (168 items, measured period last 2-3 months, conducted once, validated) | published | flavonoids: flavanones, flavonols, flavones, catechins, anthocyanidins | Y | adjusted for energy intake | NR |
| Buck et al., 2011(328) | MARIE (Mammary Carcinoma Risk Factor Investigation) | Germany | 2653 subjects, women 100%, age 50-74y | FFQ (176 items, measured period last year, conducted once, not validated) | published | lignans: enterolactone, enterodiol | N | not adjusted | NR |
| Zaineddin et al., 2012(329) | MARIE (Mammary Carcinoma Risk Factor Investigation) | Germany | 3919 cases and 7521 controls subjects, women 100%, age 50-74y | FFQ (176 items, measured period last year, conducted once, validated) | published | lignans: secoisolariciresinol and matairesinol, enterolactone and enterodiol, | N | not adjusted | NR |
| Vitelli-Storelli et al., 2020(330) | MCC Spain study | Spain | #N/A | FFQ (154 items, measured period last year, conducted once, validated) | mixed source | all classes | N | adjusted for energy intake | NR |
| Vitelli-Storelli, 2019(331) | MCC Spain study | Spain | 1779 subjects, men 44%, age 45+y | FFQ (140 items, measured period last year, conducted once, validated) | mixed source | flavonoids: anthocyanincs, chalcones, dihydrochalcones, dihydroflavonols, flavan 3 ols, flavanones, flavonols, isoflavonoids. proanthocyanidins, | N | adjusted for energy intake | NR |
| Maskarinec et al., 2018(332) | MEC (multiethnic cohort study) | USA | 151691 subjects, men 46%, age 45-75y | FFQ (0 items, measured period NR, conducted twice, validated) | Phenol-Explorer | cocoa flavanols | N | not adjusted | NR |
| Maskarinec et al., 2019(333) | MEC (multiethnic cohort study) | USA | 151691 subjects, men 46%, age 45-75y | FFQ (measured period NR, repeated once, validated) | Phenol-Explorer | flavonoids: flavanols | N | not adjusted | NR |
| Morimoto et al., 2014(334) | MEC (multiethnic cohort study) | USA | 84450 subjects, women 100%, age 45-75y | FFQ (measured period last year, repeated once, validated) | USDA +MEC | isoflavones | N | not adjusted | NR |
| Nothlings et al., 2007(335) | MEC (multiethnic cohort study) | USA | 183518 subjects, men 80%, age 45-75y | FFQ (180 items, measured period last year, conducted once, validated) | USDA +MEC | flavonols: quercetin, kaempferol, and myricetin | N | not adjusted | NR |
| Ollberding et al., 2012(336) | MEC (multiethnic cohort study) | USA | 489 subjects, women 100%, age 45-75y | FFQ (measured period last year, repeated once, validated) | USDA +MEC | isoflavones: daidzein, genistein, glycitein | N | adjusted for energy intake | NR |
| Park et al., 2008(337) | MEC (multiethnic cohort study) | USA | 82483 subjects, men 100%, age 45-75y | FFQ (>180 items, measured period last year, conducted once, validated) | USDA +MEC | isoflavones: genistein, daidzein, glycitein | N | adjusted for energy intake | NR |
| Rohrmann et al., 2018(338) | MEC (multiethnic cohort study) | USA | 1287 subjects, men 25%, age 45-75y | FFQ (180 items, measured period last year , conducted once, validated) | USDA +MEC | flavonoids: flavonols, flavanones, isoflavones | N | not adjusted | NR |
| Godos et al., 2017(339) | Mediterranean healthy eating, aging, and lifestyle (MEAL) study | Italy | 1937 subjects, NR, age 18+y | FFQ (110 items, measured period last 6 months, conducted once, validated) | Phenol-Explorer | all classes: all subclasses | N | not adjusted | NR |
| Godos et al., 2017(340) | Mediterranean healthy eating, aging, and lifestyle (MEAL) study | Italy | 1937 subjects, men 42%, age 18+y | FFQ (110 items, measured period last 6 months, conducted once, validated) | Phenol-Explorer | all classes: all subclasses | N | adjusted for energy intake | NR |
| Godos et al., 2018(341) | Mediterranean healthy eating, aging, and lifestyle (MEAL) study | Italy | 1936 subjects, men 42%, age 18+y | FFQ (110 items, measured period last 6 months, conducted once, validated) | Phenol-Explorer | isoflavones, and lignans | N | adjusted for energy intake | NR |
| Godos et al., 2018(342) | Mediterranean healthy eating, aging, and lifestyle (MEAL) study | Italy | 1572 subjects, men 42%, age 18-92y | FFQ (110 items, measured period last 6 months, conducted once, validated) | Phenol-Explorer | all classes: all subclasses | N | adjusted for energy intake | NR |
| Godos et al., 2020(343) | Mediterranean healthy eating, aging, and lifestyle (MEAL) study | Italy | 1936 subjects, men 42%, age 18+y | FFQ (110 items, measured period NR, conducted once, validated) | Phenol-Explorer | all classes: flavonoids, phenolic acids, stilbenes, lignans | N | adjusted for energy intake | NR |
| Marranzano et al., 2018(344) | Mediterranean healthy eating, aging, and lifestyle (MEAL) study | Italy | 2044 subjects, men 42%, age 18+ y | FFQ (110/18 items, measured period last year, conducted Participants completed two separate FFQs, validated) | Phenol-Explorer | flavanoids: total flavanoids, anthocyanins, flavonols, flavanols, flavanones, flavones, dihydroflavonols | N | adjusted for energy intake | NR |
| Agarwal et al., 2019(345) | Memory and Aging Project | USA | 925 subjects, men 25%, age 58-98y | FFQ (144 items, measured period last year, conducted once, validated) | mixed source | flavonoids: anthycyanidins, proanthocyanidins, total flavonoids | N | adjusted for energy intake | NR |
| Holland et al., 2020(346) | Memory and Aging Project | USA | 921 subjects, men 25%, age mean 81.2y | FFQ (144 items, measured period last year, conducted every year, validated) | mixed source | flavonols: kaempferol, quercetin, myricetin, and isorhamnetin | N | adjusted for energy intake | NR |
| Morris et al., 2018(347) | Memory and Aging Project | USA | 960 subjects, men 26%, age 58-99y | FFQ (144 items, measured period last year, conducted every year, validated) | not reported | kaempferol | N | adjusted for energy intake | NR |
| Paller et al., 2015(348) | Men Take Prostate Cancer Screening Program | USA | 90 cases and 62 controls subjects, men 100%, age 40-70y | FFQ (98 items, measured period NR, conducted once, validated) | not reported | quercetin | N | not adjusted | NR |
| Ivey et al., 2019(349) | Men’s Lifestyle Validation Study | USA | 247 subjects, men 100%, age 45-80y | FFQ (measured period last year, repeated once, validated) | mixed source | flavonoids: flavonols; flavanol monomers (excluding proanthocyanidinsn); flavanol polymers; flavones; flavanones and anthocyanins | N | not adjusted | NR |
| Pounis et al., 2016(350) | Moli-sani study | Italy | 14029 subjects, men 50%, age 35+y | FFQ (164 items, measured period last year, conducted once, validated) | USDA+ Eurofir-eBASIS | phenolic acids, flavonoids, stilbenes, lignans, derivatides of benzoic and cinnamic acids, flavonoids: (flavonols, flavones, flavanones, flavanols, anthocyanins, isoflavones) | N | not adjusted | NR |
| Pounis et al., 2016(351) | Moli-sani study | Italy | 21302 subjects, men 48%, age 35+y | FFQ (164 items, measured period last year, conducted once, validated) | mixed source | phenolic acids, flavonoids, stilbenes, lignans, derivatides of benzoic and cinnamic acids, flavonoids: (flavonols, flavones, flavanones, flavanols, anthocyanins, isoflavones) | N | not adjusted | NR |
| Pounis et al., 2018(352) | Moli-sani study | Italy | 11913 subjects, men 50%, age 35+y | FFQ (164 items, measured period last year, conducted once, validated) | mixed source | phenolic acids, flavonoids, stilbenes, lignans, derivatides of benzoic and cinnamic acids, flavonoids: (flavonols, flavones, flavanones, flavanols, anthocyanins, isoflavones) | N | not adjusted | NR |
| Pounis et al., 2018(353) | Moli-sani study | Italy | 9659 subjects, men 53%, age 35+y | FFQ (164 items, measured period last year, conducted once, validated) | mixed source | phenolic acids, flavonoids, stilbenes, lignans, derivatides of benzoic and cinnamic acids, flavonoids: (flavonols, flavones, flavanones, flavanols, anthocyanins, isoflavones) | N | not adjusted | NR |
| Nikkhah-Bodaghi et al., 2019(354) | NAFLD case-control study | Iran | 999: 196 cases (95 women, 101 men) and 803 controls (474 women, 329 men) subjects, men 43%, age 20-60y | FFQ (measured period NR, repeated once, validated) | Phenol-Explorer | total polyphenols: flavonoids, lignans, stilbenes and phenolic acids | N | not adjusted | NR |
| Carmichael et al., 2011(355) | National Birth Defects Prevention Study (NBDPS) | USA | 6584 subjects, women 100%, age NR | FFQ (58 items, measured period last year/last 6months for seasonal food, conducted once, validated) | published | isoflavones, lignans and coumestrol: total isoflavones genistein, biochanin A daidzein, glycetin formononetin, total lignans, seco, matairesinol | N | not adjusted | NR |
| Wadhwa et al., 2016(356) | National Birth Defects Prevention Study (NBDPS) | USA | 409 subjects, women 100%, age NR | FFQ (58 items, measured period 1 year before pregNRnt, conducted once, validated) | published | phytoestrogens: coumestrol; isoflavones biochanin A, daidzein, formonoetin, genistein, and glycetin; lignans mataresinol and secoisolariciresinol | N | not adjusted | NR |
| Geybels et al., 2013(357) | Netherlands Cohort Study | Netherlands | 58279 subjects, men 100%, age 55-69y | FFQ (150 items, measured period last year, conducted once, validated) | published | flavonoids: catechin, epicatechin, kaempferol, and myricetin | N | not adjusted | NR |
| Hughes et al., 2008(358) | Netherlands Cohort Study | Netherlands | 4280 subjects, men 49%, age 55-69y | FFQ (150 items, measured period last year, conducted NA, validated) | published | flavonoids: catechin, epicatechin, epicatechin gallate, galllocatechin, apigallocatechin, epigallocatachin gallate, quercetin, kaemferol, myricetin | N | not adjusted | NR |
| Simons et al., 2009(359) | Netherlands Cohort Study | Netherlands | 2485 cases and 4438 controls subjects, men cases 58% controls 49%, age 55-69y | FFQ (150 items, measured period last year , conducted once, validated) | published | flavonols, flavones and catechin: quercetin, kaempferol, myricetin, catechin and derivatives | N | not adjusted | NR |
| Vernarelli et al., 2017(360) | NHANES | USA | 9551 subjects, men 50%, age 18+y | FFQ (measured period last 24h, repeated once, validated) | USDA | flavonoids: flavonols, flavones, flavanones, flavan-3-ols and anthocyanidins | N | not adjusted | NR |
| Sun et al., 2017(361) | NIH–AARP Diet and Health Study | USA | 469008 subjects, men 59%, age 50–71y | FFQ (124 items, measured period last year, conducted once, validated) | USDA | flavonoids: (anthocyanidins, flavan-3-ols, flavanones, flavones, flavonols, and isoflavones) | Y | not adjusted | NR |
| Xiao et al., 2014(362) | NIH–AARP Diet and Health Study | USA | 491840 subjects, men 48%, age 50-71y | FFQ (measured period Last year, repeated once, validated) | USDA | flavonoids: (flavan-3-ols (catechins), flavanones, flavonols, anthocyanidins, flavones, and isoflavones | Y | adjusted for energy intake | NR |
| Mehta et al., 2016(363) | Normative Aging Study | USA | 1281 subjects, men 100%, age 49-92y | FFQ (61 items, measured period last year, conducted at least twice between 1992 - 2008, validated) | USDA | flavonoids: anthocyanins, flavanones, flavan-3-ols, flavonols , flavones, and polymers | N | not adjusted | NR |
| Adebamowo et al., 2005(364) | Nurses' Health Study (I & II) | USA | 90638 subjects, women 100%, age 25-46y | FFQ (133/142 items, measured period last year, conducted twice, validated) | self-analysed | flavonols: quercetin, kaempferol, myricetin | N | not adjusted | NR |
| Cassidy et al., 2013(365) | Nurses' Health Study (I & II) | USA | 93600 subjects, women 100%, age 25-42y | FFQ (131 items, measured period last year, conducted every 4 years, validated) | mixed source | flavoniods: flavanones (eriodictyol, hesperetin, naringenin), anthocyanins (cyanidin, delphinidin, malvidin, pelargonidin, petunidin, peonidin), flavan-3-ols (catechins, epicatachin), flavonols (quercetin, kaempferol, myricetin, isohamnetin), flavones (luteolin, apigenin) and polymers (including proanthocyanidins, theaflavins and thearubigins). | N | adjusted for energy intake | NR |
| Cassidy et al., 2014(366) | Nurses' Health Study (I & II) | USA | 171940 subjects, women 100%, age NHS I 30-55, NHS II 25-42y | FFQ (131 items, measured period last year, conducted every 4 years, validated) | mixed source | flavoniods: flavanones (eriodictyol, hesperetin, and naringenin), anthocyanins (cyanidin, delphinidin, malvidin, pelargonidin, petunidin, and peonidin), flavan-3-ols (catechins and epicatachin), flavonols (quercetin, kaempferol, myricetin, and isohamnetin), flavones (luteolin and apigenin), flavonoid polymers (proanthocyanidins, theaflavins, and thearubigins), and proanthocyanidins alone. | N | adjusted for energy intake | NR |
| Chang et al., 2016(367) | Nurses' Health Study (I & II) | USA | 82643 subjects, women 100%, age NHS I 30-55, NHS II 25-42y | FFQ (131 items, measured period last year, conducted every 4 years, validated) | mixed source | flavoniods: flavonols (quercetin, kaempferol, myricetin, and isohamnetin), flavones (luteolin and apigenin), flavanones (eriodictyol, hesperetin, and naringenin), flavan-3-ols monomers (catechins, gallocatechins, epicatechins, epigallocatechin, epicatechin-3-gallate, and epigallocatechin-3-gallate), anthocya- nins (cyanidin, delphinidin, malvidin, pelargonin, petunidin, and peonidin), and polymers (proanthocyanidins, theaflavins, and thearubigins) | N | adjusted for energy intake | NR |
| Devore et al., 2012(368) | Nurses' Health Study (I & II) | USA | 16010 subjects, women 100%, age 30-55y | FFQ (61/130 items, measured period last year, conducted every 4 years, validated) | USDA+ Eurofir-eBASIS | flavonoids: anthocyanidins, flavonols, flavones, flava- nones, flavan-3-ols, and polymeric flavonoids | N | not adjusted | NR |
| Gates et al., 2007(369) | Nurses' Health Study (I & II) | USA | 66940 subjects, women 100%, age 30-55y | FFQ (126 items, measured period last year, conducted every 4 years, validated) | published | flavonoids: flavonols (myricetin, kaempferol and quercetin) and 2 flavones (luteolin and apigenin) | N | not adjusted | NR |
| Samieri et al., 2014(370) | Nurses' Health Study (I & II) | USA | 13818 subjects, women 100%, age 30-55y | FFQ (61/116 items, measured period NR, conducted every 4 years, validated) | USDA+ Eurofir-eBASIS | flavonoids: flavonols (quercetin, kaempferol, myricetin, and isohamnetin), flavones (luteolin and apigenin), flavanones (eriodictyol, hesperetin, and naringenin), flavan-3-ol monomers (catechins and epicatechins), flavan-3-ol polymers (including proanthocyanidins, theflavins, and thearubigins), and anthocyanins (cyanidin, delphinidin, malvidin, pelargonin, petunidin, and peonidin) | N | not adjusted | NR |
| Wu et al., 2019(371) | Nurses' Health Study (I & II) | USA | 3528 subjects, boys 47%, age 12.5–17.5y | FFQ (131 items, measured period NR, conducted once, validated) | mixed source | flavonoids:  flavanones, flavones, flavan-3-ols, anthocyanins, and isoflavones. | Y | adjusted for energy intake | NR |
| Zhong et al., 2015(372) | Nurses' Health Study (I & II) | USA | 573 subjects, men 100%, age 55+y | FFQ (measured period last year, repeated once, validated) | USDA | flavonoids: anthocyanins (cyanidin, delphinidin, malvidin, pelargonidin, petunidin, peonidin), flavanones (eriodictyol, hesperetin, naringenin), flavan‐3‐ols (catechins, gallocatechins, epicatachin, epigallocatechin, epicatachin‐3‐gallate, epigallocatechin‐3‐gallate), flavonols (quercetin, kaempferol, myricetin, isohamnetin), flavones (luteolin, apigenin), and polymers including proanthocyanidins | N | not adjusted | NR |
| Cassidy et al., 2011(373) | Nurses’ Health Study II &I, Health Professionals Follow-Up Study | USA | 156957 subjects, men 15%, age NHS I 30-55, NHS II 25-42, HPFS 40-75y | FFQ (131 items, measured period last year, conducted every 4 years, validated) | mixed source | flavoniods: flavanones (eriodictyol, hesperetin, and nar- ingenin), anthocyanins (cyanidin, delphinidin, malvidin, pelargonidin, petunidin, and peonidin), flavan-3-ols (catechins and epicatachins), flavonoid polymers (including proanthocyanidins, theaflavins, and thearubigins), flavonols (quercetin, kaempferol, myricetin, and isohamnetin), and flavones (luteolin and apigenin). | N | adjusted for energy intake | NR |
| Ding et al., 2016(374) | Nurses’ Health Study II &I, Health Professionals Follow-Up Study | USA | 163457 subjects, men 13%, age NHS I 30-55, NHS II 25-42, HPFS 40-75y | FFQ (131 items, measured period last year, conducted every 4 years, validated) | USDA | isoflavones: genistein, daidzein and glycecin | N | not adjusted | NR |
| Gao et al., 2012(375) | Nurses’ Health Study II &I, Health Professionals Follow-Up Study | USA | 129617 subjects, men 38%, age men 40-75, women 30-55y | FFQ (131 items, measured period last year, conducted every 4 years, validated) | mixed source | flavonoids: flavanones, anthocyanins, flavan-3-ols, flavonols, flavones, and polymers | N | adjusted for energy intake | NR |
| Kang et al., 2018(376) | Nurses’ Health Study II &I, Health Professionals Follow-Up Study | USA | 107672 subjects, men 39%, age 40-75y | FFQ (116 items, measured period last year, conducted every 4 years, validated) | published | flavonoids: flavonols, anthocuyanidins, polymeric flavanols, flavones | N | adjusted for energy intake | NR |
| Lin et al., 2007(377) | Nurses’ Health Study II &I, Health Professionals Follow-Up Study | USA | 66360 subjects, women 100%, age 30-55y | FFQ (131 items, measured period last year, conducted 3 times, validated) | published | flavonols, flavones: quercetin, kaemferol, myricetin | N | adjusted for energy intake | NR |
| Ma et al., 2020(378) | Nurses’ Health Study II &I, Health Professionals Follow-Up Study | USA | 210700: 74241 women from the NHS, 94233 women from the NHSII 42226 men from the Health Professionals Follow-Up Study subjects, men 20%, age 25-75y | FFQ (130 items, measured period last year, conducted every 4 years, validated) | USDA | isoflavones: daidzein, genistein, glycitein | N | adjusted for energy intake | NR |
| Nimptsch et al., 2016(379) | Nurses’ Health Study II &I, Health Professionals Follow-Up Study | USA | 48842 subjects, 100% women for NHS / 100% men for HPFS, age HPFS 40-75, NHS 30-55y | FFQ (measured period last year, repeated every 4y, validated) | mixed source | flavonoids: flavonols, flavones, flavanones, flavan-3-ols, anthocyanins, and polymeric/oligomeric flavonoids (including proanthocyanidins, theaflavins, and thearubigins) | N | adjusted for energy intake | NR |
| Wedick et al., 2012(380) | Nurses’ Health Study II &I, Health Professionals Follow-Up Study | USA | 200894 subjects, women 100%, age 30-55y | FFQ  (118/131-166 items, measured period last year, conducted every 2 years, validated) | mixed source | flavonoids : flavonols, flavones, flavanones, flavan-3-ols, and anthocyanins | N | not adjusted | NR |
| Thanos et al., 2006(381) | Ontario Women’s Diet and Health Study | Canada | 6464 subjects, women 100%, age 25–74y | FFQx2 (55 items, measured period habitual intake during adolescence and adults, conducted once, not validated) | published | isoflavones and lignans: total isoflavone (genistein and daidzein, as well as formononetin and biochanin A when available) and lignan (secoisolariciresinol and matairesinol) | N | not adjusted | NR |
| Letenneur et al., 2007(382) | PAQUID (Personnes Age´es Quid: ‘‘what about older persons’’) study | France | 1640 subjects, men 42%, age 65+y | FFQ (15 items, measured period NR, conducted once, not validated) | published | flavonoids | N | not adjusted | NR |
| Heald et al., 2006(383) | PCANDIET Study | UK | 203 subjects, men 100%, age 50-75y | FFQ (155 items, measured period last 2-3 months, conducted once, validated) | published | isoflavones: genistein, daizein | N | not adjusted | serum: serum phytooestrogen: genistein and daidzein and equol, Isoflavone vs. serum daidzein (r=0.24, P<0.001)/ genistein (r=0.26, P<0.001) /total isoflavonoids (sum of daidzein, genistein and equol) (r =0.27, P<0.001) |
| Heald et al., 2007(384) | PCANDIET Study | UK | 604 cases and 911 controls subjects, men 100%, age 50-74y | FFQ (155 items, measured period last 2-3 months, conducted once, validated) | published | phytoestrogens: genistein and daidzein, the daidzein metabolite equol and the lignan enterolactone | N | adjusted for energy intake | serum: serum phytooestrogen: genistein and daidzein and equol, NR |
| Milder et al., 2007(385) | POLIEP study | Netherlands | 637 subjects, men 58%, age men 21-75, women 19-75y | FFQ (178 items, measured period last year, conducted once, validated) | published | lignans: lari, pino, seco, mat | N | not adjusted | Plasma: plasma enterolactone and enterodiol, lignan intake vs. plasma enterodiol (r = 0.09, P = 0.03) / enterolactone (r=0.18, P =0.001) |
| Jennings et al., 2020(386) | PopGen control cohort | Germany | 618 subjects, men 60%, age 25-83y | FFQ (112 items, measured period last year, conducted once, validated) | mixed source | flavonoids: flavanones, anthocyanidins, flavan-3-ols, flavonols, flavones, polymers, proanthocyanidinsn | N | not adjusted | NR |
| Creus-Cuadros et al., 2017(387) | PREDIMED | Spain | 7169 subjects, men 43%, age mean 67.1y | FFQ (137 items, measured period last year, conducted once, validated) | Phenol-Explorer | lignans: pinoresinol, 1-acetoxypinoresinol , lariciresinol, syringaresinol , secoisolariciresinol ,isolariciresinol, medioresinol , and matairesinol. | N | adjusted for energy intake | NR |
| Tresserra-Rimbau et al., 2013(388) | PREDIMED | Spain | 7200 subjects, NR, age mean 67.1y | FFQ  (137 items, measured period last year, conducted once, validated) | Phenol-Explorer | all classes: all subclasses | N | not adjusted | NR |
| Tresserra-Rimbau et al., 2019(389) | PREDIMED | Spain | 2132 subjects, men 39%, age mean 60.6y | FFQ, adherence questionnaire (143 items, measured period last year, conducted once, validated) | Phenol-Explorer | all classes: proanthocyanins, flavanones, flavones, flavonols, anthocyanidins, catechins, hydroxycinnamic acids, hydroxybenzoic acids, stilbenes, lignans | N | adjusted for energy intake | NR |
| Wesselink et al., 2020(390) | Pregnancy Study Online (PRESTO) and Snart Foraeldre (SF) | North America, Denmark | 5689 subjects, men 45%, age 20+y | FFQ (277 items, measured period last year, conducted once, validated) | mixed source | phytoestrogens: isoflavones, lignans, and coumestans | N | adjusted for energy intake | NR |
| Reger et al., 2018(391) | Prostate, Lung, Colorectal and Ovarian Cancer Screening Trial | USA | 27004 subjects, women 100%, age mean 62.7y | FFQ (137 items, measured period last year , conducted once, validated) | USDA | phytoestrogens: genistein, daidzein, glycitein, formononetin, biochanin A and coumestrol. | N | not adjusted | NR |
| Goetz et al., 2016(392) | REGARDS (REasons for Geographic and Racial Differences in Stroke) study | USA | 16678 subjects, men 45%, age >45y | FFQ (107 items, measured period last year, conducted once, validated) | USDA | flavonoids: anthocyanidins, flavan-3-ols, flavanones, flavone, flavonols, and proanthocyanidins | N | not adjusted | NR |
| Goetz et al., 2016(393) | REGARDS (REasons for Geographic and Racial Differences in Stroke) study | USA | 20024 subjects, men 44%, age >45y | FFQ (107 items, measured period last year, conducted once, validated) | USDA | flavonoids: anthocyanidins, flavan-3-ols, flavanones, flavones, flavonols, isoflavones, and proanthocyanidins | N | not adjusted | NR |
| De Rijk et al., 1997(394) | Rotterdam Study | Netherlands | 5342 subjects, men cases 52% controls 41%, age 55-95y | FFQ (170 items, measured period NR, conducted once, validated) | Dutch Food Composition Table. | flavanoids | N | not adjusted | NR |
| Devore et al., 2010(395) | Rotterdam Study | Netherlands | 5395 subjects, NR, age 55-95y | FFQ (170 items, measured period NR, conducted once, validated) | Dutch Food Composition Table. | flavanoids | N | not adjusted | NR |
| Engelhart et al., 2002(396) | Rotterdam Study | Netherlands | 5395 subjects, men 41%, age 55+y | FFQ (measured period NR, repeated once, validated) | Dutch Food Composition Table. | flavanoids | N | not adjusted | NR |
| Geleijnse et al., 2002(397) | Rotterdam Study | Netherlands | 4807 subjects, men 28%, age 55+y | FFQ (170 items, measured period last year, conducted once, validated) | published | flavonoids: flavonols quercetin, kaempferol, and myricetin | N | not adjusted | NR |
| Pantavos et al., 2015(398) | Rotterdam Study | Netherlands | 3209 subjects, women 100%, age 55+y | FFQ (170 items, measured period last year, conducted once, validated) | Oslo Antioxidant Food Table | flavonoids | N | adjusted for energy intake | NR |
| Ramdas et al., 2012(399) | Rotterdam Study | Netherlands | 3502 subjects, men 41%, age 55+y | FFQ (170 items, measured period NR, conducted once, validated) | Dutch Food Composition Table. | flavonoids | N | adjusted for energy intake | NR |
| Horn-Ross et al., 2002(400) | San Francisco Bay Area thyroid cancer study | USA | 608 cases and 558 controls subjects, women 100%, age 20-74y | FFQ (measured period last year, repeated once, not validated) | USDA | phytoestrogens: isoflavones: genistein, daidzein, biochanin A, and formononetin; the coumestan: coumestrol; and the lignans: matairesinol and secoisolariciresinol. | N | not adjusted | NR |
| Tedeschi-Blok et al., 2006(401) | San Francisco Bay Area thyroid cancer study | USA | 802 cases and 846 controls subjects, men cases 57% controls 55%, age 0 cases 55.4, controls 55.1y | FFQ (79 items, measured period NR, conducted once, validated) | USDA | phytoestrogens: coumestrol isoflavones, lignans | N | adjusted for energy intake | NR |
| Ursin et al., 2006(402) | SCHS and the Singapore Breast Screening Project (SBSP) | Singapore | 406 subjects, women 100%, age SCHS 45-74, SBSP 50-69y | ffq (165 items, measured period last year, conducted once, validated) | published | isoflavones: genistein, daidzein, and glycitein | Y | not adjusted | NR |
| Portman et al., 2016(403) | Seattle Children's Hospital (SCH) Kawasaki cohort, | USA | 181 cases and 193 controls subjects, men cases 61% controls 51%, age mean cases 4.0, controls 5.2y | FFQx2 (soy for mothers, another FFQ for children) (soy FFQ 20, children's FFQ 89 items, measured period soy FFQ: during pregNRncy /children's FFQ: NR, conducted once, validated) | USDA | isoflavones: genistein | Y | not adjusted | NR |
| Gardeazabal et al., 2019(404) | Seguimiento Universidad de Navarra’ (SUN) project | Spain | 10713 subjects, women 100%, age mean 34.7y | FFQ (136 items, measured period NR, conducted once, validated) | Phenol-Explorer | all classes: flavonoids, lignans, stilbenes, phenolic acids, alkylphenols, tyrosols, hydroxybenzaldehydes, hydroxybenxoketones, hydroxycoumarins and methoxyphenols) | N | adjusted for energy intake | NR |
| Mendonca et al., 2019(405) | Seguimiento Universidad de Navarra’ (SUN) project | Spain | 22279 subjects, men 39%, age 20-89y | FFQ (136 items, measured period NR, conducted once, validated) | Phenol-Explorer | flavanoids, stilbenes, lignans and (other polyphenols) | N | adjusted for energy intake | NR |
| Romanos-Nanclares et al., 2020(406) | Seguimiento Universidad de Navarra’ (SUN) project | Spain | 105 subjects, men 100%, age mean 64.9y | FFQ (136 items, measured period last year, conducted twice, validated) | Phenol-Explorer | phenolic acids: hydrobenzoic acids, hydrocunnamic acid, chlorogenic acid | N | adjusted for energy intake | NR |
| Dai et al., 2001(407) | Shanghai Breast Cancer Study | China mainland | 1459 case1559 control subjects, women 100%, age 25-64y | FFQ (measured period NR, repeated once, validated) | Chinese food composition table+published | isoflavones | N | not adjusted | NR |
| Baglia et al., 2015(408) | Shanghai Breast Cancer Survival Study | China mainland | 3976 subjects, women 100%, age 20-75y | FFQ (77 items, measured period NR, conducted 3 times, validated) | Chinese food composition table | isoflavones | N | not adjusted | NR |
| Lee et al., 2009(409) | Shanghai Women’s Health Study (SWHS) | China mainland | 73223 subjects, women 100%, age 40-70y | FFQ (77 items, measured period last year, conducted twice, validated) | Chinese food composition table | isoflavones | N | not adjusted | NR |
| Wu et al., 2012(410) | Shanghai Women’s Health Study (SWHS) | China mainland | 1005 subjects, women 100%, age 40-70y | FFQ, FFQ of soy food during last 24h (11 soy food items, measured period last year, conducted once, validated) | Chinese food composition table | isoflavones | N | not adjusted | NR |
| Wu et al., 2012(411) | Shanghai Women’s Health Study (SWHS) | China mainland | 74942  subjects, women 100%, age 40-70y | FFQ (71 items, measured period NR, conducted twice, validated) | Chinese food composition table | isoflavones | N | not adjusted | urine: spot, urine excretions of isoflavones, daidzein, genistein, and glycitein were significantly associated with the average dietary intakes of isoflavones, daidzein, genistein, and glycitein from FFQs (0.13 ≤ r ≤ 0.19). |
| Yang et al., 2009(412) | Shanghai Women’s Health Study (SWHS) | China mainland | 68412 subjects, women 100%, age 40-70y | FFQ  (measured period last year, repeated twice, not validated) | Chinese food composition table | isoflavones | N | not adjusted | NR |
| Yang et al., 2013(413) | Shanghai Women’s Health Study (SWHS) | China mainland | 444 subjects, women 100%, age 40-70y | FFQ (measured period NR, repeated twice, not validated) | Chinese food composition table | isoflavones | N | not adjusted | NR |
| Yu et al., 2015(414) | Shanghai Women’s Health Study (SWHS) | China mainland | 66832 subjects, women 100%, age 40-70y | FFQ (77 items, measured period last year, conducted twice, validated) | Chinese food composition table | isoflavones | Y | adjusted for energy intake | urine: spot, dietary vs. urinary isoflavonoids (r = 0.25) |
| Zhang et al., 2005(415) | Shanghai Women’s Health Study (SWHS) | China mainland | 24403 subjects, women 100%, age 40-70y | FFQ (measured period last year, repeated twice, validated) | Chinese food composition table | isoflavones | N | not adjusted | NR |
| Clark et al., 2013(416) | Singapore Chinese Health Study | Singapore | 29579 subjects, men 23%, age 45-74y | FFQ (165 items, measured period last year, conducted once, validated) | Singapore Food Composition Tables | isoflavones | N | adjusted for energy intake | NR |
| Koh et al., 2009(417) | Singapore Chinese Health Study | Singapore | 63257 subjects, men 44%, age 45-74y | FFQ (measured period NR, repeated once, validated) | Singapore Food Composition Tables | isoflavones: diadzein, genistein | Y | not adjusted | NR |
| Mueller et al., 2012(418) | Singapore Chinese Health Study | Singapore | 43176 subjects, men 42%, age 45-74y | FFQ (165 items, measured period NR, conducted once, validated) | Singapore Food Composition Database | isoflavones: daidzein, genistein, glycetin | N | adjusted for energy intake | NR |
| Paul et al., 2019(419) | Singapore Chinese Health Study | Singapore | 30744 subjects, women 100%, age 45-74y | FFQ (165 items, measured period NR, conducted once, validated) | Singapore Food Composition Database | isoflavones: daidzein, genistein, glycitein | N | adjusted for energy intake | NR |
| Sun et al., 2002(420) | Singapore Chinese Health Study | Singapore | 63257 subjects, men 44%, age 45–74y | ffq (165 items, measured period last year, conducted once, validated) | Singapore Food Composition Tables | isoflavones : genistein, daidzein, and glycitein | N | not adjusted | NR |
| Talaei et al., 2014(421) | Singapore Chinese Health Study | singapore | 63257 subjects, men 44%, age 45–74y | ffq (165 items, measured period last year, conducted once, validated) | Singapore Food Composition Tables | isoflavones | Y | not adjusted | NR |
| Wu et al., 2002(422) | Singapore Chinese Health Study | Singapore | 63257 subjects, women 100%, age 45–74 y | FFQ (165 items, measured period last year, conducted once, validated) | Singapore Food Composition Tables | isoflavones: daidzein, genistein, and glycitein | N | not adjusted | NR |
| Wu et al., 2008(423) | Singapore Chinese Health Study | Singapore | 27954 subjects, NR, age 45–74 y | FFQ (165 items, measured period last year, conducted once, validated) | Singapore Food Composition Tables | isoflavones | Y | not adjusted | NR |
| Goodman-Gruen et al., 2003(424) | Soy Health Effects (SHE) Study | USA | 208 subjects, women 100%, age 45-74y | FFQ (8 soy food items, measured period last year, conducted once, validated) | published | isoflavones: genistein and daidzein | N | not adjusted | NR |
| Kritz-Silverstein et al., 2002(425) | Soy Health Effects (SHE) Study | USA | 208 subjects, women 100%, age 45-74y | FFQ (measured period last year, repeated once, validated) | published | isoflavones: daidzein and genistein | N | not adjusted | NR |
| Kyle et al., 2010(426) | Study of Colorectal Cancer in Scotland | UK | 264 cases and 408 controls subjects, men cases 57% controls 52% , age cases 39-92, controls 32-88y | FFQ (175 items, measured period last 2-3 months, conducted once, validated) | published | non-tea flavonol, flavon-3-ol, procyanidin and flavanone : flavonols – quercetin, kaempferol and myricetin; flavones – lutein and apigenin; flavon-3-ols – catechin, epicatechin, epigallocatechin, epigallocatechin gallate, epicatechin gallate and gallocatechin; procyanidins – types bi-iv; flavanones – hesperidin and naringenin. | Y | not adjusted | NR |
| Theodoratou et al., 2007(427) | Study of Colorectal Cancer in Scotland | UK | 1456 cases and 1456 controls subjects, men cases and controls 58%, age 16-79y | FFQ  (150 items, measured period 1 year before diagnosis/ interview, conducted once, validated) | published | flavonoids : flavonols, flavones, flavan-3-ols (catechins), procyanidins, flavanones, and phytoestrogens | Y | not adjusted | NR |
| Petrick et al., 2015(428) | Study of Reflux Disease | USA | 170 cases and 183 controls subjects, NR, age 20-80y | FFQ (131 items, measured period last year, conducted once, validated) | USDA | flavonoids: anthocyanidins, flavan-3-ols, flavanones, flavones, flavanols, isoflavones | N | not adjusted | NR |
| Elaine Waetjen et al., 2012(429) | Study of Women’s Health Across the Nation (SWAN) | USA | 1459 subjects, women 100%, age 42-52y | FFQ (103+12~16 items, measured period last year, conducted 3 times, validated) | published | phytoestrogens: 4 isoflavones, 4 lignans and coumestrol | Y | not adjusted | NR |
| Gold et al., 2007(430) | Study of Women’s Health Across the Nation (SWAN) | USA | 3013 subjects, women 100%, age 42-52y | FFQ (103 items, measured period last year, conducted once, validated) | USDA | isoflavones: genistein | N | not adjusted | NR |
| Gold et al., 2013(431) | Study of Women’s Health Across the Nation (SWAN) | USA | 3302 subjects, women 100%, age 42-52y | FFQ (103 items, measured period last year, conducted 3 times, validated) | published | isoflavones and lignans | N | not adjusted | NR |
| Greendale et al., 2002(432) | Study of Women’s Health Across the Nation (SWAN) | USA | 1927 subjects, women 100%, age 42-52y | FFQ (103 items, measured period last year, conducted once, not validated) | published | isoflavones: daidzein and genistein | N | not adjusted | NR |
| Greendale et al., 2012(433) | Study of Women’s Health Across the Nation (SWAN) | USA | 2721 subjects, women 100%, age 42-52y | FFQ (103+12~16 items, measured period last year, conducted 3 times, validated) | published | isoflavones and lignans: daidzein, genistein, formononetin, glycetin/lariciresinol, pinoresinol, secoisolariciresinol, matariesinol | Y | not adjusted | NR |
| Greendale et al., 2015(434) | Study of Women’s Health Across the Nation (SWAN) | USA | 853 subjects, women 100%, age 42-52y | FFQ (103+12~16 items, measured period last year, conducted 3 times, not validated) | published | isoflavones | N | not adjusted | NR |
| Huang et al., 2006(435) | Study of Women’s Health Across the Nation (SWAN) | USA | NR subjects, NR, age 42-52y | FFQ (103 items, measured period last year, conducted once, validated) | published | isoflavones: diadzein, genistein | Y | adjusted for energy intake | NR |
| Huang et al., 2012(436) | Study of Women’s Health Across the Nation (SWAN) | USA | 2721 subjects, women 100%, age 42-52y | FFQ (103+12~16 items, measured period last year, conducted 3 times, not validated) | published | phytoestrogens: daidzein, genistein, glycitein, formononetin, matairesinol, lariciresinol, pinoreinol, secoisolariciresinol | N | not adjusted | NR |
| Sowers et al., 2006(437) | Study of Women’s Health Across the Nation (SWAN) | USA | 1881 subjects, women 100%, age 42–52y | FFQ (103 items, measured period last year, conducted once, not validated) | published | all classes: hydroxybenzoic acids, hydroxycinnamic acids, anthocyanidins, flavonols, monomeric flavanols, flavanones, isoflavones (daidzein and genistein), flavones | N | not adjusted | NR |
| Frankenfeld et al., 2008(438) | Surveillance, Epidemiology, and End Results (SEER) study | USA | 466 cases and 90 controls subjects, men cases 54% controls 50%, age 20-74y | FFQ (117 items, measured period last year, conducted once, not validated) | USDA | flavonoids: flavonols, flavones, flavanones, catechins, epicatechins, anthocyanidins, proanthocyanidins, and isoflavones | N | not adjusted | NR |
| Le Marchand et al., 2000(439) | Surveillance, Epidemiology, and End Results (SEER) study | USA | 582 cases and 582 controls subjects, men 50%, age mean male cases 65.5 controls 65.4, female cases 66 controls 65.6 y | FFQ (242 items, measured period last year, conducted once, validated) | USDA+published | flavonoids : quercetin, kaempferol, myricetin, hesperidin, naringin | N | adjusted for energy intake | NR |
| Konishi et al., 2019(440) | Takayama Study | Japan | 13521 subjects, men 44%, age 35-69y | FFQ (169 items, measured period last year, conducted once, validated) | published | isoflavones: daidzein, genistein | N | adjusted for energy intake | NR |
| Nagata et al., 2001(441) | Takayama Study | Japan | 101 subjects, postmenopausal women 100%, age 35-54y | FFQ (169 items, measured period last year, conducted once, validated) | published | soy isoflavones | N | adjusted for energy intake | NR |
| Nagata et al., 2001(442) | Takayama Study | Japan | 29079 subjects, men 46%, age 35+y | FFQ (169 items, measured period last year, conducted once, validated) | published | soy isoflavones | N | adjusted for energy intake | NR |
| Nagata et al., 2008(443) | Takayama Study | Japan | 31 subjects, premenopausal women 100%, age 35-54y | FFQ (169 items, measured period last year, conducted once, validated) | published | soy isoflavones: genistein, daidzein | N | adjusted for energy intake | NR |
| Nagata et al., 2017(444) | Takayama Study | Japan | 11229 subjects, men 46%, age 35-69y | FFQ (169 items, measured period last year, conducted once, validated) | published | soy isoflavones | N | adjusted for energy intake | NR |
| Oba et al., 2007(445) | Takayama Study | Japan | 30221 subjects, men 46%, age 35+y | FFQ (169 items, measured period last year, conducted once, validated) | published | isoflavones | N | adjusted for energy intake | NR |
| Taguchi et al., 2020(446) | Takayama Study | Japan | 6404 subjects, men 33%, age 30+y | FFQ (169 items, measured period last year, conducted once, validated) | published | total polyphenols | N | adjusted for energy intake | NR |
| Wada et al., 2013(447) | Takayama Study | Japan | 15607 subjects, women 100%, age 35+y | FFQ (169 items, measured period last year, conducted once, validated) | published | flavonoids: isoflavone | N | adjusted for energy intake | NR |
| Wada et al., 2015(448) | Takayama Study | Japan | 16573 subjects, women 100%, age 35+y | FFQ (169 items, measured period last year, conducted once, validated) | published | flavonoids: isoflavone | N | adjusted for energy intake | NR |
| Wada et al., 2018(449) | Takayama Study | Japan | 30817 subjects, men 46%, age 35+y | FFQ (169 items, measured period last year, conducted once, validated) | published | flavonoids: isoflavone | N | adjusted for energy intake | NR |
| Segovia-Siapco et al., 2018(450) | Teen Food and Development Study | USA | 248 subjects, men 100%, age 12-18y | FFQ (151 items, measured period last 6 months, conducted once, validated) | Nutrition Data Systems for Research + Institutional developed database | isoflavones: genistein, daidzein | Y | adjusted for energy intake | NR |
| Sohrab et al., 2013(451) | Tehran Lipid and Glucose Study | Iran | 2618 subjects, men 44%, age 19-84y | FFQ (168 items, measured period last year, conducted once, validated) | USDA+ Phenol-Explorer | all classes: flavonoids, phenolic acids, lignans, stilbenes | N | adjusted for energy intake | NR |
| Sohrab et al., 2018(452) | Tehran Lipid and Glucose study | Iran | 1265 subjects, men 44%, age 19-74y | FFQ (168 items, measured period last year, conducted once, validated) | mixed source | all classes: flavonoids, phenolic acids, lignans, stilbenes | N | adjusted for energy intake | NR |
| Horn-Ross et al., 2001(453) | The Bay Area Breast Cancer Study | USA | 1326 cases and 1657 controls subjects, women 100%, age 35-79y | FFQ (measured period last year, repeated once, not validated) | published | isoflavones: genistein, daidzein, biochanin, formonetin | N | not adjusted | NR |
| Fuhrman et al., 2008(454) | The Biomarkers for Breast Cancer Prevention Study(B4BCP) | USA | 232 subjects, women 100%, age 48-82y | FFQ (10+109 items, measured period NR, conducted once, not validated) | published | isoflavones | N | not adjusted | urine: equol, NR |
| Arts et al., 2001(455) | The Iowa Women’s Health Study (IWHS) | USA | 34492 subjects, women 100%, age 55-69y | FFQ (127 items, measured period NR, conducted once, validated) | published +self-analysed | catechin: (+ )-catechin, ( +)-gallocatechin, ( -)-epicatechin, ( -)-epigallocat- echin, ( -)-epicatechin gallate and ( -)-epigallocatechin gallate | N | not adjusted | NR |
| Arts et al., 2002(456) | The Iowa Women’s Health Study (IWHS) | USA | 21502 subjects, women 100%, age 55-69y | FFQ (127 items, measured period NR, conducted once, validated) | published | catechin: (+ )-catechin, ( +)-gallocatechin, ( -)-epicatechin, ( -)-epigallocat- echin, ( -)-epicatechin gallate and ( -)-epigallocatechin gallate | N | not adjusted | NR |
| Cutler et al., 2008(457) | The Iowa Women’s Health Study (IWHS) | USA | 34708 subjects, women 100%, age 55-69y | FFQ (127 items, measured period last year, conducted once, validated) | USDA | flavanoids: anthocyanidins, flavones, flavanones, flavonols, flavan-3-ols (monomers), isoflavones and proanthocyanidins (condensed tannins or flavan-3-ol polymers) | N | not adjusted | NR |
| Mink et al., 2007(458) | The Iowa Women’s Health Study (IWHS) | USA | 34489 subjects, women 100%, age 55-69y | FFQ (127 items, measured period NR, conducted once, validated) | USDA | flavonoids: anthocyanidins, flavanones, flavones, flavonols, isoflavones, flavanols, proanthocyanidins | N | adjusted for energy intake | NR |
| Thompson et al., 2010(459) | The Iowa Women’s Health Study (IWHS) | USA | 35159 subjects, women 100%, age 55–69 y | FFQ (127 items, measured period last year, conducted once, validated) | USDA | flavonoids: isoflavones, flavonols, and anthocyanidins, proanthocyanidins | N | not adjusted | NR |
| Akhter et al., 2008(460) | The Japan Public Health Center-based Prospective Study (JPHC Study) | Japan | 83063 subjects, men 47%, age 45-74y | FFQ (138 items, measured period last year, conducted once, validated) | published | isoflavones: genistein and daidzein | Y | adjusted for energy intake | NR |
| Budhathoki et al., 2015(461) | The Japan Public Health Center-based Prospective Study (JPHC Study) | Japan | 49121 subjects, women 100%, age 45-74y | FFQ (138 items, measured period NR, conducted once, validated) | published | isoflavones: daidzein, genistein | Y | adjusted for energy intake | NR |
| Hara et al., 2012(462) | The Japan Public Health Center-based Prospective Study (JPHC Study) | Japan | 84881 subjects, men 47%, age 45-74y | FFQ (138 items, measured period last year, conducted twice, validated) | published | isoflavones: genisteinand daidzein | Y | adjusted for energy intake | NR |
| Kokubo et al., 2007(463) | The Japan Public Health Center-based Prospective Study (JPHC Study) | Japan | 40462 subjects, men 26%, age 40-59y | FFQ (1990 3 soy food/1995 8 soy food items, measured period last month, conducted once, validated) | published | isoflavones: genistein, daidzein | Y | not adjusted | NR |
| Kurahashi et al., 2007(464) | The Japan Public Health Center-based Prospective Study (JPHC Study) | Japan | 43509 subjects, men 100%, age 45-74y | FFQ (147 items, measured period last year, conducted once, validated) | published | phytoestrogens: genistein, daidzein, | Y | adjusted for energy intake | NR |
| Kurahashi et al., 2009(465) | The Japan Public Health Center-based Prospective Study (JPHC Study) | Japan | 68974 subjects, men 49%, age 40-69y | FFQ (52 items, measured period last year, conducted once, validated) | published | phytoestrogens: genistein, daidzein, | Y | adjusted for energy intake | NR |
| Nanri et al., 2010(466) | The Japan Public Health Center-based Prospective Study (JPHC Study) | Japan | 59791 subjects, men 43%, age 45-75y | FFQ (147 items, measured period last year, conducted once, validated) | published | isoflavones: genistein, daidzein | Y | adjusted for energy intake | NR |
| Nozue et al., 2017(467) | The Japan Public Health Center-based Prospective Study (JPHC Study) | Japan | 4165 subjects, men 22%, age 40-69y | FFQ (52 items, measured period NR, conducted once, validated) | published | isoflavones: genistein, daidzein | N | adjusted for energy intake | NR |
| Shimazu et al., 2010(468) | The Japan Public Health Center-based Prospective Study (JPHC Study) | Japan | 76661 subjects, men 47%, age 45-74y | FFQ (138 items, measured period last year, conducted twice, validated) | published | isoflavones: genistein, daidzein | N | adjusted for energy intake | NR |
| Suzuki et al., 2010(469) | The Japan Public Health Center-based Prospective Study (JPHC Study) | Japan | 50757 subjects, women 100%, age 40–69y | FFQ (44/52 baseline survey, 46/138 in the 5‐year follow‐up survey items, measured period last year, conducted 3 times, validated) | Japanese food composition tables | isoflavones | Y | adjusted for energy intake | NR |
| Yamamoto et al., 2003(470) | The Japan Public Health Center-based Prospective Study (JPHC Study) | Japan | 21852 subjects, women 100%, age 40-59y | FFQ (38 (calculated only from2 soy items) items, measured period NR, conducted once, validated) | published | isoflavones: genistein | Y | not adjusted | NR |
| Rice et al., 2001(471) | the Kame Project | USA | 274 subjects, women 100%, age 65-93y | FFQ (14 items, measured period last year, conducted once, not validated) | USDA | isoflavones: daidzein, genistein | N | not adjusted | NR |
| Zamora-Ros et al., 2018(472) | The Mexican Teachers’ Cohort (MTC) study | Mexico | 115315 subjects, women 100%, age 25+y | FFQ  (140 items, measured period 1 year before diagnosis, conducted once, validated) | Phenol-Explorer | all classes: all subclasses | N | adjusted for energy intake | NR |
| Tabak et al., 2001(473) | The MORGEN study (Monitoring Project on Risk Factors and Health in the Netherlands) | Netherlands | 17453 subjects, men 46%, age 20 to 59y | FFQ (178 items, measured period Last year, conducted once, validated) | published | flavonoids: catechin, flavonol, and flavone | N | not adjusted | NR |
| Bandera et al., 2011(474) | The NJ Ovarian Cancer Study+EDGE Study | USA | 233 case and 467 controls subjects, women 100%, age 21+y | FFQ (110+21 items, measured period last 6 months, conducted once, not validated) | USDA | phytoestrogens: daidzein, genistein, formononetin, glycitein/ matairesinol, lariciresinol. pinoresinol. secoisolariciresinol.coumestrol | N | adjusted for energy intake | NR |
| Zhang et al., 2019(475) | The Shanghai Women’s Health Study(SWHS) and The Shanghai Men’s Health (SMHS) | China mainland | 476108 subjects, NR, age 35-70y | FFQ (measured period NR, repeated once, validated) | Chinese Food Composition Table | isoflavones | N | not adjusted | urine: daidzein, genistein, glycitein, and equol, NR |
| Suzuki et al., 2008(476) | The Swedish Mammography Cohort (SMC) | Sweden | 51823 subjects, women 100%, age <70y | FFQ  (measured period last 20 years, repeated once, validated) | published | lignans: secoisolariciresinol, matairesinol, lariciresinol, and pinoresinol | Y | not adjusted | NR |
| Lin et al., 2013(477) | The Swedish Mammography Cohort (SMC) and the Cohort of Swedish Men (COSM) | Sweden | 594 subjects, men cases Esophageal adenocarcinoma 87%/ Gastroesophageal junctional adenocarcinoma 85%/ Esophageal squamous cell carcinoma 72%, Control 83% , age <80y | FFQ (96 items, measured period last 20 years, conducted once, validated) | published | lignans: lariciresinol, pinoresinol, syringaresinol, and medioresinol | Y | not adjusted | NR |
| McCann et al., 2012(478) | theDBBR at the RPCI | USA | 683 cases and 611 controls subjects, women 100%, age 26-89y | FFQ (110 items, measured period last year, conducted once, not validated) | USDA | lignans: matairesinol, lariciresinol, pinoresinol, and secoisolariciresinol | N | not adjusted | NR |
| Williams et al., 2015(479) | theDBBR at the RPCI | USA | 216 subjects, women 100%, age mean cases 56, controls 53y | FFQ (110 items, measured period last year, conducted once, not validated) | USDA | lignans | N | not adjusted | NR |
| Yao et al., 2019(480) | Tianjin Chronic Low-grade Systemic Inflammation and Health (TCLSIHealth) cohort | China mainland | 1989 subjects, women 100%, age 25-42y | FFQ (100 items, measured period last month, conducted once, validated) | Chinese Food Composition Table | flavonoids: quercetin | Y | not adjusted | NR |
| Yao et al., 2019(481) | Tianjin Chronic Low-grade Systemic Inflammation and Health (TCLSIHealth) cohort | China mainland | 14711 subjects, men 55%, age mean 42y | FFQ (100 items, measured period last month, conducted once, validated) | Chinese Food Composition Table | flavonoids: myrcetin | Y | not adjusted | NR |
| Vitale et al., 2017(482) | TOSCA.IT trial | Italy | 2573 subjects, men 60%, age 50–75 y | FFQ  (248 items, measured period NR , conducted once, validated) | USDA | all classes | N | adjusted for energy intake | NR |
| Vitale et al., 2018(483) | TOSCA.IT trial | Italy | 2573 subjects, men 60%, age 50–75 y | FFQ  (248 items, measured period NR , conducted once, validated) | USDA+ Phenol-Explorer | all classes: flavonoids, phenolic acids, stilbenes, lignans other | N | adjusted for energy intake | NR |
| Jennings et al., 2012(484) | Twins UK | UK | 1898 subjects, women 100%, age 18-75y | FFQ (131 items, measured period last year, conducted once, validated) | USDA+ Phenol-Explorer | flavonoids: flavanones (eriodictyol, hesperetin, and naringenin), anthocyanins (cyanidin, delphinidin, malvidin, pelargonidin, petunidin, and peonidin), flavan-3-ols (catechins and epicatechins), flavonols (quercetin, kaempferol, myricetin, and isohamnetin), flavones (luteolin and apigenin), and polymers (including proanthocyanidins, theaflavins, and thearubigins). | N | adjusted for energy intake | NR |
| Jennings et al., 2017(485) | Twins UK | UK | 2734 subjects, women 100%, age 18-83y | FFQ (131 items, measured period last year, conducted once, validated) | mixed source | flavonoids: flavanones, anthocyanina, flavan-3-ols, flavonols, flavones, polymers, proanthocyanidins. | N | not adjusted | NR |
| Welch et al., 2012(486) | Twins UK | UK | 3160 subjects, women 100%, age 18-79y | FFQ (131 items, measured period last year, conducted once, validated) | mixed source | flavonoids: (flavanones, anthocyanins, flavan-3-ols,polymers, flavonols, and flavones | N | not adjusted | NR |
| Noorwali et al., 2018(487) | UK Women’s Cohort study (UKWCS) | UK | 13958 subjects, women 100%, age mean 52y | FFQ (217 items, measured period last year, conducted once, validated) | Phenol-Explorer | all classes: flavonoids, phenolic acids, stilbenes, lignans, and other polyphenols | N | not adjusted | NR |
| Petrick et al., 2015(488) | USA Multicenter Study | USA | 1054 cases and 662 controls subjects, NR, age 30-79y | FFQ (104 items, measured period last 4-5 years, conducted once, validated) | USDA | flavonoids: anthocyanidins, flavan-3-ols, flavanones, flavones, flavanols, isoflavones | N | not adjusted | NR |
| McCann et al., 2004(489) | WEB Study | USA | 1122 cases and 2036 controls subjects, women 100%, age 35-79y | FFQ (147 items, measured period last 1-2 years, conducted once, validated) | published | lignans | Y | not adjusted | NR |
| McCann et al., 2006(490) | WEB Study | USA | 851 cases and 2036 controls subjects, women 100%, age NR | FFQ (measured period last 1-2 years, repeated once, not validated) | published | lignans | N | not adjusted | NR |
| McCann et al., 2010(491) | WEB Study | USA | 1122 subjects, women 100%, age 35-79y | FFQ (147 items, measured period last 1-2 years, conducted once, validated) | published | lignans: secoisolariciresinol (122.4 ± 76.4 mcg/d), followed by lariciresinol (63.1 ± 35.5 mcg/d), pinoresinol (52.6 ± 39.7 mcg/d), and matairesinol (7.2 ± 4.5 mcg/d) | N | not adjusted | NR |
| McCann et al., 2003(492) | Western New York Diet Study | USA | 696 controls and 126 cases subjects, women 100%, age 40-85y | FFQ (172 items, measured period last 2 years, conducted once, validated) | published | phytoestrogens: quercetin; kaempferol; ecoisolariciresinol | N | not adjusted | NR |
| McCann et al., 2005(493) | Western New York Diet Study | USA | 433 cases and 538 controls subjects, men 100%, age NRy | FFQ (172 items, measured period last 2 years, conducted once, validated) | published | phytoestrogens: lignans, quercetin, kaempferol | N | not adjusted | NR |
| Sesso et al., 2003(494) | Women’s Health Study (WHS) | USA | 38445 subjects, women 100%, age 45+y | FFQ (131 items, measured period last year , conducted once, validated) | food tables maintained by the Department of Nutrition, Harvard School of Public Health | flavonols and flavones: quercetin, kaempferol, myricetin, apigenin, and luteolin | N | adjusted for energy intake | NR |
| Song et al., 2005(495) | Women’s Health Study (WHS) | USA | 38018 subjects, women 100%, age 45+y | FFQ (131 items, measured period last year, conducted once, validated) | published | flavonols and flavones: quercetin, kaempferol, myricetin, apigenin, and luteolin | N | not adjusted | NR |
| Wang et al., 2009(496) | Women’s Health Study (WHS) | USA | 38408 subjects, women 100%, age 45+y | FFQ (131 items, measured period NR, conducted once, validated) | published +self-analysed | flavonoids: flavonols (quercetin, kaempferol, and myricetin) and flavones (apigenin and luteolin) | N | adjusted for energy intake | NR |
| Caan et al., 2011(497) | Women’s Healthy Eating and Living (WHEL) study | USA | 3088 subjects, women 100%, age 18-70y | FFQ (152 items, measured period last year, conducted once, validated) | USDA | isoflavones | N | not adjusted | NR |
| Hedelin et al., 2008(498) | Women’s Lifestyle and Health Cohort (WLH study) | Sweden | 96000 subjects, women 100%, age 30-49y | FFQ (80 items, measured period last 6 months, conducted once, validated) | published | isoflavones, coumestans, lignans: (genistein, daidzein, biochanin A, formononetin, and equol, coumestrol,mat and seco) | N | not adjusted | NR |
| Hedelin et al., 2011(499) | Women’s Lifestyle and Health Cohort (WLH study) | Sweden | 47140 subjects, women 100%, age 30-49y | FFQ (80 items, measured period last 6 months, conducted once, validated) | published | isoflavones, coumestans, lignans: genistein, daidzein, biochania, formononetin, equol, coumestrol. mat, seco, ariciresinol, pinoresinol, syringaresinol, and medioresinol | N | not adjusted | NR |
| Hedelin et al., 2016(500) | Women’s Lifestyle and Health Cohort (WLH study) | Sweden | 49258 subjects, women 100%, age 30–49y | FFQ (80 items, measured period last 6 months, conducted once, validated) | published | total lignans, isoflavones, coumestrol, enterolignans and equol | N | adjusted for energy intake | NR |
| Frankenfeld et al., 2003(501) | Women's Health Initiative WHI FFQ | USA | 96 subjects, women 100%, age 50-79y | FFQx2 (122+20 soy foods items, measured period last 3 months, conducted once, validated) | USDA | isoflavones: genistein, daidzein | Y | not adjusted | plasma: daidzein and genistein, daidzein intake from soy FFQ / comprehensive FFQ vs. plasma r = 0.37/ 0.35; genistein intake from soy FFQ / comprehensive FFQ vs. plasma r = 0.43/0.38 |
| Boucher et al., 2018(502) |  | Canada | 278 subjects, women 100%, age 25-74y | FFQ (17+3 items, measured period last 2 months, conducted once, not validated) | published | phytoestrogens: isoflavones: genistein daidzein formononetin glycitein and lignans: secoisolariciresinol, pinoresinol, lariciresinol, matairesinol | N | not adjusted | NR |
| Christensen et al., 2012(503) |  | Canada | 1061 cases and 1425 controls subjects, men case 61% control 49%, age 35-75y | FFQ (42 items, measured period last 2 years, conducted once, not validated) | USDA | flavonoids: anthocyanidins , flavan-3-ols , flavones , flavonols and flavanones | N | adjusted for energy intake | NR |
| Garcia-Larsen et al., 2015(504) |  | Chile | 1232 subjects, men 46%, age 22-28y | FFQ (65 items, measured period NR, conducted once, validated) | published | flavonoids: flavonols, flavones and catechins | N | not adjusted | NR |
| Guthrie et al., 2000(505) |  | Australia | 354 subjects, women 100%, age 45-55y | FFQ, isoflavone FFQ (measured period last year, repeated once, validated) | database from Commonwealth Scientific and Industrial Research Organization (CSIRO) | isoflavones | N | not adjusted | NR |
| Lagiou et al., 2004(506) |  | Greece | 154 cases and 145 controls subjects, women 100%, age 35+y | FFQ (47 items, measured period NR, conducted once, not validated) | USDA | flavonoids: flavanones, flavan-3-ols, flavonols | N | not adjusted | NR |
| Lagiou et al., 2004(507) |  | Greece | 110 cases and 100 controls subjects, men cases 52% controls 49% , age mean cases 64.5, controls 59.8y | FFQ (80 items, measured period last 5 years, conducted once, not validated) | USDA | flavonoids: flavanones, flavan-3-ols, flavonols, flavones, anthocyanidins and isoflavones | N | not adjusted | NR |
| Lagiou et al., 2004(508) |  | Greece | 329 cases and 570 controls subjects, men cases 86% controls 57%, age mean cases 57.9, controls 56.5y | FFQ (110 items, measured period 1 year before diagnosis/interview, conducted once, validated) | USDA | total flavonoids: flavanones, flavan 3 ols, flavonols, flavones, athocyanidins, isoflavones, | N | not adjusted | NR |
| Lagiou et al., 2006(509) |  | Greece | 100 cases and 100 controls subjects, men cases 88% controls 87%, age NRy | FFQ (110 items, measured period last year, conducted once, not validated) | USDA | flavonoids: flavanones, flavan 3ols, flavonols, flavones, isoflavones, | N | not adjusted | NR |
| Lagiou et al., 2008(510) |  | Greece | 339 cases and 360 controls subjects, men cases HCC virus positive 88%/ HCC virus negative 74%/ Cholangiocarcinoma 6%, controls 83%, age mean cases: HCC virus positive 62.9, HCC virus negative67.1, Cholangiocarcinoma 62.2,  controls 64y | FFQ (110 items, measured period 1 year before diagnosis/interview, conducted once, not validated) | USDA | flavonoids: flavanones, flavan-3-ols, flavonols, anthocy- anidins, flavones, and isoflavones | N | not adjusted | NR |
| Linseisen et al., 2004(511) |  | Germany | 278 cases and 666 controls subjects, women 100%, age <50y | FFQ (176 items, measured period last year, conducted once, validated) | published | isoflavones coumestanes and lignans: daidzein, genistein, formononetin, biochanin A, coumestrol secoisolariciresinol, matairesinol | N | not adjusted | NR |
| Murphy et al., 2019(512) |  | Australia | 1183 subjects, men 37%, age 39-65y | FFQ (215 items, measured period NR, conducted once, validated) | USDA | flavonoids and subclasses: flavanols, isoflavones, flavonols, flavones, flavanones, anthocyanidins and proanthocyanidins | N | not adjusted | NR |
| Mykoniatis et al., 2018(513) |  | Greece | 350 subjects, men 100%, age 18-40y | FFQ (67 items, measured period NR, conducted once, not validated) | USDA | flavonoids: anthocyanidins, flavanols, flavnones, flavones, flavonols | N | not adjusted | NR |
| Peterson et al., 2003(514) |  | Greece | 820 cases and 1548 controls subjects, women 100%, age mean cases 56.4, controls 54.4y | FFQ (115 items, measured period last year, conducted once, validated) | USDA | flavonoids: flavonols, flavones, flavanones, flavanols, anthocyanidins, if | N | not adjusted | NR |
| Praud et al., 2018(515) |  | Italy | 1294 cases and 1451 controls subjects, men 100%, age 46-74y | FFQ (78 items, measured period NR, conducted once, not validated) | European Institute of Oncology (EIO) database | lignans: mat, seco, pino, lari | N | adjusted for energy intake | |
| Rossi et al., 2010(516) |  | Italy | 9622 cases and 16050 controls subjects, NR, age NR | FFQ (78 items, measured period last 2 years, conducted once, validated) | USDA | flavonoids: anthocyanidins, flavan-3-ols, flavanones, flavones, flavanols, isoflavones | N | not adjusted | NR |
| Veldink et al., 2007(517) |  | Netherlands | 132 cases and 220 controls subjects, men cases 35% controls 62%, age 25-81y | FFQ (104+37 items, measured period 1 year before diagnosis/same year for controls, conducted once, not validated) | USDA | flavonols, phytoestrogens | N | adjusted for energy intake | NR |
| Youseflu et al., 2020(518) |  | Iran | 24138 subjects, men 54%, age mean 40.5y | FFQ (147 items, measured period last year, conducted once, validated) | USDA | phytoestrogens: total isoflavones, formononetin and glycitein, total lignans, secoisolariciresinol and lariciresinol and matairesinol, and total coumestrol | N | not adjusted | NR |
| **mixed methods (p75-81)** | | | | | | | | | |
| Yahya et al., 2016(519) | Leeds Women’s Wellbeing Study (LWW) and the Diet and Health Study (DH) | UK | 246 subjects, women 100%, age 18–50y | 3DD, 7DD (/ items, measured period last 3 and 7 days, conducted once, not validated) | USDA+Phenol-explorer | all classes | N | not adjusted | NR |
| Hertog et al., 1995(520) | seven countries studies cohorts | European countries, Japan, US | 12763 subjects, men 100%, age 40-59y | 7DD, 24h recall (in US only) (measured period last 7 days/ last 24h, repeated once, not validated) | self-analysed | flavonoids: quercetin, kaemferol, myricetin, luteolin, apigenin | N | not adjusted | NR |
| Lin et al., 2006(521) | Nurses’ Health Study II &I, Health Professionals Follow-Up Study | USA | 107401 subjects, men 49%, age 30-55y | FFQ, 24h recall (131 items, measured period last year, conducted 3 times, validated) | published | toal flavonoids, flavonols: quercetin. kaemfrol, myricetin | N | not adjusted | NR |
| Carrion-Garcia et al., 2017(522) |  | Spain | 50 subjects, men 44%, age 23-62y | FFQ, 24h recall (136 items, measured period last year/last day, conducted once, validated) | USDA | total polyphenols | N | not adjusted | plasma: plasma total polyphenol from FCR(Folin-Ciocalteu), no significant correlation between total dietary polyphenol from FFQ /24h recall and plasma total polyphneol |
| Chavez-Suarez et al., 2017(523) |  | Mexico | 100 subjects, women 100%, age 25-80y | FFQ, 24h recall (162 items, measured period NR, conducted once, validated) | USDA+ Mexico database | lignans, isoflavones, flavonoids, and resveratrol: daidzein genistein glicitein biochanin A formononetin equol total isoflavones coumestrol resveratrol secoisolariciresinol matairesinol enterodiol enterolactone total lignans naringenin luteolin kaempferol quercetin total flavonoids | N | adjusted for energy intake | urine: 12h, dietary (FFQ), vs. urinary resveratrol (r = 0.337, p < 0.01). total phytoestrogens (r = 0.730, p < 0.001)/ 24 h recall vs urinay genistein (r = 0.374, p < 0.01), naringenin (r = 0.620, p < 0.0001), isoflavones (r = 0.460, p < 0.001), lignans (r = 0.550, p < 0.0001), flavonoids (r = 0.240, p < 0.05), and total phytoestrogens (r = 0.410, p < 0.001) |
| Bhakta et al., 2005(524) |  | UK | 108 subjects, women 100%, age 25-75y | FFQ, 24h recall (277 items, measured period last 24h/last year, conducted once, validated) | published | isoflavones and lignans: genistein, daidzein, secoisolariciresinol. matairesinol | Y | adjusted for energy intake | serum: daidzein, genistein, enterolactone, FFQ vs plasma levels genistein (r= 0.21, P =0.12) daidzein (r= 0.32, P =0.02), enterolactone (r=0.10, P =0.43), 24-h recalls vs. h plasma levels were 0.43 (P<0.001), 0.40 (P =0.002), and 0.08 (P =0.50), respectively. |
| Maskarinec et al., 1998(525) | Hawaii Honolulu mammography clinicts women | USA | 106 subjects, women 100%, age 36-80y | FFQ, 24h recall (50 items, measured period last year / last 24h, conducted once, not validated) | published | soy isoflavoness : daidzien, genistein, dma, equol, commestrol | N | not adjusted | Urine: daidzein, glycitein, DMA and coumestrol, urinary excretion of isoflavones vs. annual dietary soy isoflavone (r=0.31 P<0.0016) |
| Horn-Ross et al., 2006(526) | California Teachers Study (CTS) | USA | 195 subjects, women 100%, age <85y | FFQ, 24h recall (measured period last year/ last 24h, repeated twice, validated) | published | isoflavones, lignans, coumestrol : biochanin A, formononetin, genistein, daidzein, matairesino, secoisolariciresinol | Y | not adjusted | Urine: phytoestrogens, dietary vs. urinary levels isoflavones/lignans validity range 0.41- 0.55/ 0.16 to 0.21 |
| Goni et al., 2019(527) | Garrucha Older Health Study | Spain | 109 subjects, men 45%, age 80+y | FFQ, 24h recall (measured period NR, repeated 3 times, validated) | self-analysed | total phenolics | N | not adjusted | NR |
| Nishimuro et al., 2015(528) | The Tanno-Sobetsu study | Japan | 570 subjects, 2DFR: 8 women , FFQ1: men 37%, FFQ2: men 40%, age FFQ1: 20-93, FFQ2: 41-91y | FFQ, 2-day weighted food record (15/14 items, measured period weighed record: 2 consecutive days / FFQ: NR, conducted once, not validated) | self-analysed | quercetin | N | not adjusted | NR |
| Hoge et al., 2019(529) | STROXYBIO project | Belgium | 53 subjects, men 47%, age 20-60y | FFQ, 3DD (167 items, measured period last 3 months, last 3 days, conducted once, validated) | Phenol-Explorer | total polyphenols | Y | adjusted for energy intake | Urine: total polyphenols, FFQ/FR vs. urinary total polyphenol r=0.32/ 0.57 |
| Wilson et al., 2009(530) | Alpha-Tocopherol Beta-Carotene Cancer Prevention Study | USA | 27111 subjects, men 100%, age 50–69y | FFQ, 3DD (203 items, measured period Last year, conducted once, validated) | Finland food composition database | flavonoids | Y | adjusted for energy intake | NR |
| Somerset et al., 2014(531) |  | Australia | 60 subjects, NR, age 18-55y | FFQ, 3DD (62 items, measured period NR, conducted once, validated) | USDA | flavonoids: flavan 3-ols, flavonols, flavones, flavanones, anthocyanidins | Y | not adjusted | NR |
| Huang et al., 2000(532) |  | USA | 69 subjects, women 100%, age NA Japanese 48.4, Caucasian 45.8y | FFQ, 48h recall (126 items, measured period NR, conducted once, validated) | published | isoflavones: daidzein, genistein | Y | not adjusted | urine: daidzein or genistein, dietary intake from modified Block FFQ vs. urinary concentration of daidzein/genistein (r=0.49/0.30) |
| French et al., 2007(533) | Toronto Osteoporosis Prevention Study | Canada | 26 subjects, women 100%, age 23-42y | FFQ, 48h recall (53 items, measured period last 2 months/ last 2 days, conducted once, validated) | published | isoflavones and lignans : isoflavones (genistein and daidzein) and lignans (secoisolariciresinol and matairesinol) | Y | not adjusted | urine: Isoflavone (genistein, daidzein) and lignan (enterolactone, enterodiol and secoisolariciresinol), 48h recall/FFQ isoflavone vs. urinary excretion of metabolites (r =0.64, p <0.001 and r=0.54, p<0.004, respectively). Lignans (r =0.46, p =0.02 and r =0.40, p <0.05, respectively) |
| Ishihara et al., 2009(534) |  | Brazil | 55 subjects, women 100%, age NR | FFQ, 4DD (118 items, measured period last year, conducted FFQ twice at one-year interval/DR twice, validated) | published | isoflavones: daidzein, genistein | Y | not adjusted | NR |
| Hakim et al., 2001(535) |  | USA | 120 subjects, men 46%, age 40-82y | FFQ, 4DD, Arizona Tea Questionnaires. (measured period NR, 4 days, repeated once, validated) | The Arizona Tea Questionnaire (ATQ) database | flavonoids: catechins, theaflavins, thearubigens, gallic acid | Y | not adjusted | NR |
| Cao et al., 2010(536) |  | China mainland | 164 (128 have recall, 92 have plasma) subjects, NR, age 20-28y | FFQ, 7DD (126 items, measured period last year/last week, conducted FFQ twice, 7day record twice 4months apart, validated) | self-analysed | flavonoids: quercetin,kaempferol,isorhamnetin,apigenin,luteolin | Y | energy asjusted and unadjusted | plasma: flavonol and flavone, including quercetin, kaempferol, isorhamnetin, apigenin, and luteolin, FFQ vs. plasma r=0.52 for flavonol and 0.41 for flavone and ranged from 0.32 (quercetin) to 0.44 (kaempferol) (all P <0 .05). The complete and partial agreement by quartiles ranged from 70% to 89%. |
| Bingham et al., 2008(537) | EPIC-Norfolk | UK | 12474 subjects, NR, age 45-75y | FFQ, 7DD (130 items, measured period last year, conducted twice, validated) | DINER | isoflavones and lignans: daidzein, genistrin | N | not adjusted | plasma, urine: daidzein and genistein, food diary vs. urine(genistein/daidzein): 0.060/0.071, and serum: 0.053/0.070 , between FFQ and urine:0.016/0.024 and serum:0.015/0.021 (all P<0.05) |
| Yamamoto et al., 2001(538) | The Japan Public Health Center-based Prospective Study (JPHC Study) | Japan | 247 subjects, men 83%, age mean men 55.6, women 55.3y | FFQ, 7DD (147 items, measured period last year/last week, conducted once, validated) | published | flavonoids: isoflavones | Y | adjusted for energy intake | serum and urine: daidzein, energy-adjusted intakes of daidzein from FFQ vs. DR/serum concentration/ creatinine-adjusted urinary excretion (r= 0.64/ 0.31/ 0.43) |
| Yang et al., 2014(539) |  | USA | 40 subjects, women 100%, age 40–70 y | FFQ, 7DD (74 items, measured period last month, conducted once, validated) | NDSR and the Flavonoid/Proanthocyanidin  Provisional Table | flavonoids: isoflavones,anthocyanins,flavan-3-ols,flavanones,flavones,flavonols | Y | not adjusted | NR |
| Verkasalo et al., 2001(540) | EPIC-Oxford | UK | 80 subjects, women 100%, age 20-39y | FFQ, 7DD (measured period last year, repeated once, validated) | USDA | isoflavones | Y | not adjusted | plasma: daidzein and genistein , daidzein FFQ vs.plasma r=0.74, 7DD vs plasma r=0.79/ genistein FFQ vs plasma r=0.69, 7DD vs plasma r=0.80 |
| Kreijkamp-Kaspers et al., 2005(541) |  | Netherlands | 301 subjects, women 100%, age 60-75y | FFQ, dietary history interview (170 items, measured period last year, conducted once, validated) | published+ consult | isoflavones: isoflavones daidzein, genistein, formononetin and biochanin A, and for the lignans matairesinol and secoisolariciresinol | N | adjusted for energy intake | NR |
| Vian et al., 2015(542) |  | Brazil | 120 subjects, women 100%, age mean 27y | FFQ, 3DD, 24h recall (52 items, measured period NR, conducted twice, validated) | USDA+ Phenol-Explorer+analysed | total polyphenols | Y | adjusted for energy intake | Urine: total polyphenols, FFQ/24HR vs. urinary total polyphenols r=0.22/ 0.23 (P < 0.05). |
| Kalmijn et al., 1997(543) | Zutphen study | Netherlands | 1266 subjects, men 100%, age 64-84y | cross-check dietary history method (measured period last 2-4 weeks, repeated twice, not validated) | published | flavonoids: quercetin, kaempferol, myricetin, apigenin, and luteolin, the five major antioxidant food flavonoid | N | not adjusted | NR |
| Keli et al., 1996(544) | Zutphen study | Netherlands | 552 subjects, men 100%, age 40-59y | cross-check dietary history method (measured period last 6 -12 months, repeated 3 times, not validated) | published | flavonoids: quercetin, kaempferol, myricetin, luteolin, and apigenin. | N | adjusted for energy intake | NR |
| Dower et al., 2016(545) | Zutphen Elderly Study | Netherlands | 774 subjects, men 100%, age 65-84y | cross-check dietary history method (measured period last month, repeated 4 times, validated) | self-analysed | catechin: (-)-epicatechin, (+)-catechin, (-)-epigallocatechin, (-)-epicatechin gallate (ecg), (-)-epigallocatechin gallate (egcg), and (+)-gallocatechin | N | not adjusted | NR |
| Arts et al., 2001(546) | Zutphen Elderly Study | Netherlands | 728 subjects, men 100%, age 65-84y | cross-check dietary history method (measured period last month, repeated once, validated) | published | catechin: (+ )-catechin, ( +)-gallocatechin, ( -)-epicatechin, ( -)-epigallocat- echin, ( -)-epicatechin gallate and ( -)-epigallocatechin gallate | N | not adjusted | NR |
| Milder et al., 2006(547) | Zutphen study | Netherlands | 570 subjects, men 100%, age 65-84y | cross-check dietary history method (measured period weekends and weekdays, repeated 3 times, not validated) | published | flavonoids, lignans: flavonols, flavones, catechins, lari, pino, seco, mat | N | not adjusted | NR |
| Ruidavets et al., 2000(548) |  | France | 182 subjects, men 55%, age 35-65y | dietary recall of evening meal, 3DD (measured period last evening meal, repeated once, validated) | published | catechin | N | not adjusted | fasted plasma: (+)-catechin, dietary vs plasma catechin (r=0.58, P<0.001) |
| Brat et al., 2006(549) | SECODIP, SUVIMAX | France | 6639 subjects, men 42%, age 35-60y | purchase log, 24h recall (measured period last week/ last 24h, repeated log: 13 times per year/24h recall: every 2months for a total of 6 per year, validated) | self-analysed | total polyphenols | N | not adjusted | NR |

1. Abbreviations: 3DD: 3-day dietary record/diary, 4DD: 4-day dietary record/diary; 7DD: 7-day dietary record/diary; 30DD: 30-day dietary record/diary; FFQ: food frequency questionnaire, DHI: diet history interview; DHQ: diet history questionnaire; NR: not reported; N: no; Y: yes.

2.* The table is sorted by dietary assessment methods, study/cohort name and first author. The starting and ending page numbers of each dietary assessment method were listed at the start of each dietary assessment methods in the table and here: 24h-recall (p1-8), 48h-recall (p8), 3DD (p8-9), 4DD (p9-10), 7DD (p10-12), 30DD (p12), 3-day weighted food records (p12-14), 4-day weighted food records (p14), centre-specific methods (p14-16), DHI (p17), DHQ (p17-19), FFQ (p19-75), mixed methods (p75-81).

**Supplemental References:**

1. Hervert-Hernandez D, Goni I. Contribution of beverages to the intake of polyphenols and antioxidant capacity in obese women from rural Mexico. Public Health Nutrition 2012;15(1):6-12. doi: 10.1017/s1368980011001753.

2. Kilkkinen A, Valsta LM, Virtamo J, Stumpf K, Adlercreutz H, Pietinen P. Intake of lignans is associated with serum enterolactone concentration in Finnish men and women. Journal of Nutrition 2003;133(6):1830-3.

3. Otaki N, Kimira M, Katsumata SI, Uehara M, Watanabe S, Suzuki K. Distribution and major sources of flavonoid intakes in the middle-aged Japanese women. Journal of Clinical Biochemistry and Nutrition 2009;44(3):231-8. doi: <http://dx.doi.org/10.3164/jcbn.08-231>.

4. Song Y, Paik HY, Joung H. Soybean and soy isoflavone intake indicate a positive change in bone mineral density for 2 years in young Korean women. Nutrition Research 2008;28(1):25-30. doi: 10.1016/j.nutres.2007.11.001.

5. Lefevre-Arbogast S, Gaudout D, Bensalem J, Letenneur L, Dartigues JF, Hejblum BP, Feart C, Delcourt C, Samieri C. Pattern of polyphenol intake and the long-term risk of dementia in older persons. Neurology 2018;90(22):e1979-e88. doi: <http://dx.doi.org/10.1212/WNL.0000000000005607>.

6. Rizzi F, Conti C, Dogliotti E, Terranegra A, Salvi E, Braga D, Ricca F, Lupoli S, Mingione A, Pivari F, et al. Interaction between polyphenols intake and PON1 gene variants on markers of cardiovascular disease: a nutrigenetic observational study. J Transl Med 2016;14:10. doi: 10.1186/s12967-016-0941-6.

7. Zamora-Ros R, Knaze V, Lujan-Barroso L, Kuhnle GGC, Mulligan AA, Touillaud M, Slimani N, Romieu I, Powell N, Tumino R, et al. Dietary intakes and food sources of phytoestrogens in the European Prospective Investigation into Cancer and Nutrition (EPIC) 24-hour dietary recall cohort. European Journal of Clinical Nutrition 2012;66(8):932-41. doi: <http://dx.doi.org/10.1038/ejcn.2012.36>.

8. Zamora-Ros R, Knaze V, Lujan-Barroso L, Romieu I, Scalbert A, Slimani N, Hjartaker A, Engeset D, Skeie G, Overvad K, et al. Differences in dietary intakes, food sources and determinants of total flavonoids between Mediterranean and non-Mediterranean countries participating in the European Prospective Investigation into Cancer and Nutrition (EPIC) study. British Journal of Nutrition 2013;109(8):1498-507. doi: <http://dx.doi.org/10.1017/S0007114512003273>.

9. Zamora-Ros R, Knaze V, Romieu I, Scalbert A, Slimani N, Clavel-Chapelon F, Touillaud M, Perquier F, Skeie G, Engeset D, et al. Impact of thearubigins on the estimation of total dietary flavonoids in the European Prospective Investigation into Cancer and Nutrition (EPIC) study. European Journal of Clinical Nutrition 2013;67(7):779-82. doi: <https://dx.doi.org/10.1038/ejcn.2013.89>.

10. Zamora-Ros R, Rothwell JA, Scalbert A, Knaze V, Romieu I, Slimani N, Fagherazzi G, Perquier F, Touillaud M, Molina-Montes E, et al. Dietary intakes and food sources of phenolic acids in the European Prospective Investigation into Cancer and Nutrition (EPIC) study. British Journal of Nutrition 2013;110(8):1500-11. doi: <https://dx.doi.org/10.1017/S0007114513000688>.

11. Zamora-Ros R, Knaze V, Rothwell JA, Hemon B, Moskal A, Overvad K, Tjonneland A, Kyro C, Fagherazzi G, Boutron-Ruault MC, et al. Dietary polyphenol intake in Europe: the European Prospective Investigation into Cancer and Nutrition (EPIC) study. European Journal of Nutrition 2016;55(4):1359-75. doi: 10.1007/s00394-015-0950-x.

12. Adriouch S, Lampure A, Nechba A, Baudry J, Assmann K, Kesse-Guyot E, Hercberg S, Scalbert A, Touvier M, Fezeu LK. Prospective Association between Total and Specific Dietary Polyphenol Intakes and Cardiovascular Disease Risk in the Nutrinet-Sante French Cohort. Nutrients 2018;10(11):15. doi: 10.3390/nu10111587.

13. Fleury S, Riviere G, Alles B, Kesse-Guyot E, Mejean C, Hercberg S, Touvier M, Bemrah N. Exposure to contaminants and nutritional intakes in a French vegetarian population. Food Chem Toxicol 2017;109(Pt 1):218-29. doi: <https://dx.doi.org/10.1016/j.fct.2017.07.048>.

14. Kuczmarski MF, Sebastian RS, Goldman JD, Murayi T, Steinfeldt LC, Eosso JR, Moshfegh AJ, Zonderman AB, Evans MK. Dietary flavonoid intakes are associated with race but not income in an urban population. Nutrients 2018;10 (11) (no pagination)(1749). doi: <http://dx.doi.org/10.3390/nu10111749>.

15. Nascimento-Souza MA, de Paiva PG, Perez-Jimenez J, do Carmo Castro Franceschini S, Ribeiro AQ. Estimated dietary intake and major food sources of polyphenols in elderly of Vicosa, Brazil: a population-based study. European Journal of Nutrition 2018;57(2):617-27. doi: <https://dx.doi.org/10.1007/s00394-016-1348-0>.

16. Miranda AM, Steluti J, Fisberg RM, Marchioni DM. Association between Polyphenol Intake and Hypertension in Adults and Older Adults: A Population-Based Study in Brazil. PLoS One 2016;11(10):14. doi: 10.1371/journal.pone.0165791.

17. Miranda AM, Steluti J, Fisberg RM, Marchioni DM. Dietary intake and food contributors of polyphenols in adults and elderly adults of Sao Paulo: a population-based study. British Journal of Nutrition 2016;115(6):1061-70. doi: <https://dx.doi.org/10.1017/S0007114515005061>.

18. Miranda AM, Steluti J, Fisberg RM, Marchioni DM. Association between Coffee Consumption and Its Polyphenols with Cardiovascular Risk Factors: A Population-Based Study. Nutrients 2017;9(3):14. doi: <https://dx.doi.org/10.3390/nu9030276>.

19. Chan SG, Ho SC, Kreiger N, Darlington G, So KF, Chong PY. Dietary sources and determinants of soy isoflavone intake among midlife Chinese Women in Hong Kong. Journal of Nutrition 2007;137(11):2451-5.

20. Laurin D, Masaki KH, Foley DJ, White LR, Launer LJ. Midlife dietary intake of antioxidants and risk of late-life incident dementia - The Honolulu-Asia Aging Study. American Journal of Epidemiology 2004;159(10):959-67. doi: 10.1093/aje/kwh124.

21. Ahn S, Jun S, Joung H. Association of Total Flavonoid Intake with Hypo-HDL-Cholesterolemia among Korean Adults: Effect Modification by Polyunsaturated Fatty Acid Intake. Nutrients 2020;12(1). doi: 10.3390/nu12010195.

22. Ham D, Jun S, Kang M, Paik HY, Joung H, Shin S. Consumption of Korean Foods with High Flavonoid Contents Reduces the Likelihood of Having Elevated C-Reactive Protein Levels: Data from the 2015-2017 Korea National Health and Nutrition Examination Survey. Nutrients 2019;11(10):04. doi: <https://dx.doi.org/10.3390/nu11102370>.

23. Jun S, Shin S, Joung H. Estimation of dietary flavonoid intake and major food sources of Korean adults. British Journal of Nutrition 2015;115(3):480-9. doi: <http://dx.doi.org/10.1017/S0007114515004006>.

24. Jun S, Chun OK, Joung H. Estimation of dietary total antioxidant capacity of Korean adults. European Journal of Nutrition 2018;57(4):1615-25. doi: <https://dx.doi.org/10.1007/s00394-017-1447-6>.

25. Kim SA, Kim J, Jun S, Wie GA, Shin S, Joung H. Association between dietary flavonoid intake and obesity among adults in Korea. Appl Physiol Nutr Metab 2020;45(2):203-12. doi: <https://dx.doi.org/10.1139/apnm-2019-0211>.

26. Yang YJ, Kim YJ, Yang YK, Kim JY, Kwon O. Dietary flavan-3-ols intake and metabolic syndrome risk in Korean adults. Nutr Res Pract 2012;6(1):68-77. doi: 10.4162/nrp.2012.6.1.68.

27. Bai W, Wang C, Ren C. Intakes of total and individual flavonoids by US adults. Int J Food Sci Nutr 2014;65(1):9-20. doi: <https://dx.doi.org/10.3109/09637486.2013.832170>.

28. Chun OK, Chung SJ, Song WO. Urinary isoflavones and their metabolites validate the dietary isoflavone intakes in US adults. J Am Diet Assoc 2009;109(2):245-54. doi: <https://dx.doi.org/10.1016/j.jada.2008.10.055>.

29. Kim K, Vance TM, Chun OK. Greater flavonoid intake is associated with improved CVD risk factors in US adults. British Journal of Nutrition 2016;115(8):1481-8. doi: 10.1017/s0007114516000519.

30. Kim K, Vance TM, Chun OK. Estimated intake and major food sources of flavonoids among US adults: changes between 1999-2002 and 2007-2010 in NHANES. European Journal of Nutrition 2016;55(2):833-43. doi: 10.1007/s00394-015-0942-x.

31. Ock KC, Chung SJ, Claycombe KJ, Song WO. Serum C-reactive protein concentrations are inversely associated with dietary flavonoid intake in U.S. adults. Journal of Nutrition 2008;138(4):753-60.

32. Mervish NA, Gardiner EW, Galvez MP, Kushi LH, Windham GC, Biro FM, Pinney SM, Rybak ME, Teitelbaum SL, Wolff MS. Dietary flavonol intake is associated with age of puberty in a longitudinal cohort of girls. Nutrition Research 2013;33(7):534-42. doi: <http://dx.doi.org/10.1016/j.nutres.2013.04.005>.

33. Mervish NA, Teitelbaum SL, Pajak A, Windham GC, Pinney SM, Kushi LH, Biro FM, Wolff MS. Peripubertal dietary flavonol and lignan intake and age at menarche in a longitudinal cohort of girls. Pediatr Res 2017;82(2):201-8. doi: <https://dx.doi.org/10.1038/pr.2017.34>.

34. Somerset SM, Johannot L. Dietary flavonoid sources in Australian adults. Nutr Cancer 2008;60(4):442-9. doi: 10.1080/01635580802143836.

35. Waskiewicz A, Zujko ME, Szczesniewska D, Tykarski A, Kwasniewska M, Drygas W, Witkowska AM. Polyphenols and dietary antioxidant potential, and their relationship with arterial hypertension: A cross-sectional study of the adult population in Poland (WOBASZ II). Adv Clin Exp Med 2019;28(6):797-806. doi: 10.17219/acem/91487.

36. Witkowska AM, Zujko ME, Waskiewicz A, Terlikowska KM, Piotrowski W. Comparison of Various Databases for Estimation of Dietary Polyphenol Intake in the Population of Polish Adults. Nutrients 2015;7(11):9299-308. doi: <https://dx.doi.org/10.3390/nu7115464>.

37. Witkowska AM, Waskiewicz A, Zujko ME, Szczesniewska D, Stepaniak U, Pajak A, Drygas W. Are Total and Individual Dietary Lignans Related to Cardiovascular Disease and Its Risk Factors in Postmenopausal Women? A Nationwide Study. Nutrients 2018;10(7):04. doi: <https://dx.doi.org/10.3390/nu10070865>.

38. Zujko ME, Witkowska AM, Waskiewicz A, Mironczuk-Chodakowska I. Dietary Antioxidant and Flavonoid Intakes Are Reduced in the Elderly. Oxidative Med Cell Longev 2015:8. doi: 10.1155/2015/843173.

39. Zujko ME, Witkowska AM, Waskiewicz A, Piotrowski W, Terlikowska KM. Dietary antioxidant capacity of the patients with cardiovascular disease in a cross-sectional study. Nutr J 2015;14:13. doi: 10.1186/s12937-015-0005-4.

40. Zujko ME, Waskiewicz A, Witkowska AM, Szczesniewska D, Zdrojewski T, Kozakiewicz K, Drygas W. Dietary Total Antioxidant Capacity and Dietary Polyphenol Intake and Prevalence of Metabolic Syndrome in Polish Adults: A Nationwide Study. Oxidative Med Cell Longev 2018;2018:7487816. doi: <https://dx.doi.org/10.1155/2018/7487816>.

41. Adriouch S, Kesse-Guyot E, Feuillet T, Touvier M, Olie V, Andreeva V, Hercberg S, Galan P, Fezeu LK. Total and specific dietary polyphenol intakes and 6-year anthropometric changes in a middle-aged general population cohort. Int J Obes 2018;42(3):310-7. doi: 10.1038/ijo.2017.227.

42. Julia C, Touvier M, Lassale C, Fezeu L, Galan P, Hercberg S, Kesse-Guyot E. Cluster analysis of polyphenol intake in a French middle-aged population (aged 35-64 years). Journal of Nutritional Science 2016;5:9. doi: 10.1017/jns.2016.16.

43. Perez-Jimenez J, Fezeu L, Touvier M, Arnault N, Manach C, Hercberg S, Galan P, Scalbert A. Dietary intake of 337 polyphenols in French adults. American Journal of Clinical Nutrition 2011;93(6):1220-8. doi: 10.3945/ajcn.110.007096.

44. Touvier M, Druesne-Pecollo N, Kesse-Guyot E, Andreeva VA, Fezeu L, Galan P, Hercberg S, Latino-Martel P. Dual association between polyphenol intake and breast cancer risk according to alcohol consumption level: a prospective cohort study. Breast Cancer Res Treat 2013;137(1):225-36. doi: 10.1007/s10549-012-2323-y.

45. Andrews MA, Schliep KC, Wactawski-Wende J, Stanford JB, Zarek SM, Radin RG, Sjaarda LA, Perkins NJ, Kalwerisky RA, Hammoud AO, et al. Dietary factors and luteal phase deficiency in healthy eumenorrheic women. Hum Reprod 2015;30(8):1942-51. doi: 10.1093/humrep/dev133.

46. Filiberto AC, Mumford SL, Pollack AZ, Zhang CL, Yeung EH, Perkins NJ, Wactawski-Wende J, Schisterman EF. Habitual Dietary Isoflavone Intake Is Associated with Decreased C-Reactive Protein Concentrations among Healthy Premenopausal Women. Journal of Nutrition 2013;143(6):900-6. doi: 10.3945/jn.112.173187.

47. Wisnuwardani RW, De Henauw S, Androutsos O, Forsner M, Gottrand F, Huybrechts I, Knaze V, Kersting M, Le Donne C, Marcos A, et al. Estimated dietary intake of polyphenols in European adolescents: the HELENA study. European Journal of Nutrition 2019;58(6):2345-63. doi: 10.1007/s00394-018-1787-x.

48. Sebastian RS, Enns CW, Goldman JD, Martin CL, Steinfeldt LC, Murayi T, Moshfegh AJ. A New Database Facilitates Characterization of Flavonoid Intake, Sources, and Positive Associations with Diet Quality among US Adults. Journal of Nutrition 2015;145(6):1239-48. doi: 10.3945/jn.115.213025.

49. Sebastian RS, Wilkinson Enns C, Goldman JD, Moshfegh AJ. Dietary flavonoid intake is inversely associated with cardiovascular disease risk as assessed by body mass index and waist circumference among adults in the united states. Nutrients 2017;9 (8) (no pagination)(827). doi: <http://dx.doi.org/10.3390/nu9080827>.

50. Balbi MA, Crivellenti LC, Zuccolotto DCC, Franco LJ, Sartorelli DS. The relationship of flavonoid intake during pregnancy with excess body weight and gestational diabetes mellitus. Arch 2019;63(3):241-9. doi: <https://dx.doi.org/10.20945/2359-3997000000143>.

51. Tseng M, Byrne C, Kurzer MS, Fang CY. Equol-producing status, isoflavone intake, and breast density in a sample of U.S. Chinese women. Cancer Epidemiol Biomarkers Prev 2013;22(11):1975-83. doi: <https://dx.doi.org/10.1158/1055-9965.EPI-13-0593>.

52. Arai Y, Uehara M, Sato Y, Kimira M, Eboshida A, Adlercreutz H, Watanabe S. Comparison of isoflavones among dietary intake, plasma concentration and urinary excretion for accurate estimation of phytoestrogen intake. Journal of epidemiology / Japan Epidemiological Association 2000;10(2):127-35.

53. Arai Y, Watanabe S, Kimira M, Shimoi K, Mochizuki R, Kinae N. Dietary intakes of flavonols, flavones and isoflavones by Japanese women and the inverse correlation between quercetin intake and plasma LDL cholesterol concentration. Journal of Nutrition 2000;130(9):2243-50.

54. Glabska D, Guzek D, Grudzinska D, Lech G. Influence of dietary isoflavone intake on gastrointestinal symptoms in ulcerative colitis individuals in remission. World J Gastroenterol 2017;23(29):5356-63. doi: 10.3748/wjg.v23.i29.5356.

55. Wada K, Tamura T, Konishi K, Goto Y, Mizuta F, Koda S, Tsuji M, Nagata C. Soy isoflavone intake and the risk of bladder cancer: the Takayama study. Cancer Science Conference: 76th Annual Meeting of the Japanese Cancer Association, JCA 2017;109(Supplement 1).

56. Wada K, Ueno T, Uchiyama S, Abiru Y, Tsuji M, Konishi K, Mizuta F, Goto Y, Tamura T, Shiraki M, et al. Relationship of equol production between children aged 5-7 years and their mothers. European Journal of Nutrition 2017;56(5):1911-7. doi: 10.1007/s00394-016-1233-x.

57. Pedret A, Valls RM, Fernandez-Castillejo S, Catalan U, Romeu M, Giralt M, Lamuela-Raventos RM, Medina-Remon A, Arija V, Aranda N, et al. Polyphenol-rich foods exhibit DNA antioxidative properties and protect the glutathione system in healthy subjects. Mol Nutr Food Res 2012;56(7):1025-33. doi: 10.1002/mnfr.201100676.

58. Skolmowska D, Glabska D, Guzek D, Lech G. Association between Dietary Isoflavone Intake and Ulcerative Colitis Symptoms in Polish Caucasian Individuals. Nutrients 2019;11(8). doi: 10.3390/nu11081936.

59. Barron E, Cano Sokoloff N, Maffazioli GDN, Ackerman KE, Woolley R, Holmes TM, Anderson EJ, Misra M. Diets High in Fiber and Vegetable Protein Are Associated with Low Lumbar Bone Mineral Density in Young Athletes with Oligoamenorrhea. J Acad Nutr Diet 2016;116(3):481-9. doi: <https://dx.doi.org/10.1016/j.jand.2015.10.022>.

60. Mursu J, Nurmi T, Tuomainen TP, Ruusunen A, Salonen JT, Voutilainen S. The intake of flavonoids and carotid atherosclerosis: the Kuopio ischaemic heart disease risk factor study. British Journal of Nutrition 2007;98(4):814-8. doi: 10.1017/s0007114507744410.

61. Mursu J, Nurmi T, Tuomainen TP, Salonen JT, Pukkala E, Voutilainen S. Intake of flavonoids and risk of cancer in Finnish men: The Kuopio Ischaemic Heart Disease Risk Factor Study. International Journal of Cancer 2008;123(3):660-3. doi: 10.1002/ijc.23421.

62. Mursu J, Voutilainen S, Nurmi T, Tuomainen TP, Kurl S, Salonen JT. Flavonoid intake and the risk of ischaemic stroke and CVD mortality in middle-aged Finnish men: The Kuopio Ischaemic Heart Disease Risk Factor Study. British Journal of Nutrition 2008;100(4):890-5. doi: <http://dx.doi.org/10.1017/S0007114508945694>.

63. Ziauddeen N, Rosi A, Del Rio D, Amoutzopoulos B, Nicholson S, Page P, Scazzina F, Brighenti F, Ray S, Mena P. Dietary intake of (poly)phenols in children and adults: cross-sectional analysis of UK National Diet and Nutrition Survey Rolling Programme (2008-2014). European Journal of Nutrition 2019;58(8):3183-98. doi: 10.1007/s00394-018-1862-3.

64. Cao J, Zhang Y, Chen W, Zhao X. The relationship between fasting plasma concentrations of selected flavonoids and their ordinary dietary intake. British Journal of Nutrition 2010;103(2):249-55. doi: <http://dx.doi.org/10.1017/S000711450999170X>.

65. Radtke J, Linseisen J, Wolfram G. Fasting plasma concentrations of selected flavonoids as markers of their ordinary dietary intake. European Journal of Nutrition 2002;41(5):203-9. doi: 10.1007/s00394-002-0377-z.

66. Taguchi C, Fukushima Y, Kishimoto Y, Saita E, Suzuki-Sugihara N, Yoshida D, Kondo K. Polyphenol Intake from Beverages in Japan over an 18-Year Period (1996-2013): Trends by Year, Age, Gender and Season. J Nutr Sci Vitaminol 2015;61(4):338-44. doi: 10.3177/jnsv.61.338.

67. Taguchi C, Kishimoto Y, Fukushima Y, Saita E, Tanaka M, Takahashi Y, Masuda Y, Goda T, Kondo K. Dietary Polyphenol Intake Estimated by 7-Day Dietary Records among Japanese Male Workers: Evaluation of the Within- and Between-Individual Variation. J Nutr Sci Vitaminol (Tokyo) 2017;63(3):180-5. doi: <https://dx.doi.org/10.3177/jnsv.63.180>.

68. Wang Y, Yang M, Lee SG, Davis CG, Kenny A, Koo SI, Chun OK. Plasma total antioxidant capacity is associated with dietary intake and plasma level of antioxidants in postmenopausal women. J nutr biochem 2012;23(12):1725-31. doi: <https://dx.doi.org/10.1016/j.jnutbio.2011.12.004>.

69. Maras JE, Talegawkar SA, Qiao N, Lyle B, Ferrucci L, Tucker KL. Flavonoid intakes in the Baltimore Longitudinal Study of Aging. J Food Compos Anal 2011;24(8):1103-9. doi: 10.1016/j.jfca.2011.04.007.

70. Grace PB, Taylor JI, Low YL, Luben RN, Mulligan AA, Botting NP, Dowsett M, Welch AA, Khaw KT, Wareham NJ, et al. Phytoestrogen concentrations in serum and spot urine as biomarkers for dietary phytoestrogen intake and their relation to breast cancer risk in European prospective investigation of cancer and nutrition-norfolk. Cancer Epidemiol Biomarkers Prev 2004;13(5):698-708.

71. Kuhnle GGC, Ward HA, Vogiatzoglou A, Luben RN, Mulligan A, Wareham NJ, Forouhi NG, Khaw KT. Association between dietary phyto-oestrogens and bone density in men and postmenopausal women. British Journal of Nutrition 2011;106(7):1063-9. doi: 10.1017/s0007114511001309.

72. Mulligan AA, Welch AA, McTaggart AA, Bhaniani A, Bingham SA. Intakes and sources of soya foods and isoflavones in a UK population cohort study (EPIC-Norfolk). European Journal of Clinical Nutrition 2007;61(2):248-54. doi: 10.1038/sj.ejcn.1602509.

73. Mulligan AA, Kuhnle GG, Lentjes MA, van Scheltinga V, Powell NA, McTaggart A, Bhaniani A, Khaw KT. Intakes and sources of isoflavones, lignans, enterolignans, coumestrol and soya-containing foods in the Norfolk arm of the European Prospective Investigation into Cancer and Nutrition (EPIC-Norfolk), from 7 d food diaries, using a newly updated database. Public Health Nutrition 2013;16(8):1454-62. doi: <https://dx.doi.org/10.1017/S1368980012003904>.

74. Vogiatzoglou A, Mulligan AA, Bhaniani A, Lentjes MAH, McTaggart A, Luben RN, Heiss C, Kelm M, Merx MW, Spencer JPE, et al. Associations between flavan-3-ol intake and CVD risk in the Norfolk cohort of the European Prospective Investigation into Cancer (EPIC-Norfolk). Free Radic Biol Med 2015;84:1-10. doi: <https://dx.doi.org/10.1016/j.freeradbiomed.2015.03.005>.

75. Ward HA, Kuhnle GGC, Mulligan AA, Lentjes MAH, Luben RN, Khaw KT. Breast, colorectal, and prostate cancer risk in the European Prospective Investigation into Cancer and Nutrition-Norfolk in relation to phytoestrogen intake derived from an improved database. American Journal of Clinical Nutrition 2010;91(2):440-8. doi: 10.3945/ajcn.2009.28282.

76. Wang Y, Yang M, Lee SG, Davis CG, Koo SI, Chun OK. Dietary total antioxidant capacity is associated with diet and plasma antioxidant status in healthy young adults. J Acad Nutr Diet 2012;112(10):1626-35. doi: <https://dx.doi.org/10.1016/j.jand.2012.06.007>.

77. Pellegrini N, Valtuena S, Ardigo D, Brighenti F, Franzini L, Del Rio D, Scazzina F, Piatti PM, Zavaroni I. Intake of the plant lignans matairesinol, secoisolariciresinol, pinoresinol, and lariciresinol in relation to vascular inflammation and endothelial dysfunction in middle age-elderly men and post-menopausal women living in Northern Italy. Nutr Metab Carbiovasc Dis 2010;20(1):64-71. doi: 10.1016/j.numecd.2009.02.003.

78. Rosi A, Mena P, Castello F, Del Rio D, Scazzina F. Comprehensive dietary evaluation of Italian primary school children: food consumption and intake of energy, nutrients and phenolic compounds. Int J Food Sci Nutr 2020. doi: 10.1080/09637486.2020.1754768.

79. Cheng G, Remer T, Prinz-Langenohl R, Blaszkewicz M, Degen GH, Buyken AE. Relation of isoflavones and fiber intake in childhood to the timing of puberty. American Journal of Clinical Nutrition 2010;92(3):556-64. doi: <https://dx.doi.org/10.3945/ajcn.2010.29394>.

80. Drossard C, Bolzenius K, Kunz C, Kersting M. Anthocyanins in the diet of children and adolescents: intake, sources and trends. European Journal of Nutrition 2013;52(2):667-76. doi: <https://dx.doi.org/10.1007/s00394-012-0371-z>.

81. Krupp D, Remer T, Penczynski KJ, Bolzenius K, Wudy SA, Buyken AE. Relevance of fruits, vegetables and flavonoids from fruits and vegetables during early life, mid-childhood and adolescence for levels of insulin-like growth factor (IGF-1) and its binding proteins IGFBP-2 and IGFBP-3 in young adulthood. British Journal of Nutrition 2016;115(3):527-37. doi: <https://dx.doi.org/10.1017/S0007114515004742>.

82. Penczynski KJ, Krupp D, Bring A, Bolzenius K, Remer T, Buyken AE. Relative validation of 24-h urinary hippuric acid excretion as a biomarker for dietary flavonoid intake from fruit and vegetables in healthy adolescents. European Journal of Nutrition 2017;56(2):757-66. doi: <https://dx.doi.org/10.1007/s00394-015-1121-9>.

83. Penczynski KJ, Remer T, Herder C, Kalhoff H, Rienks J, Markgraf DF, Roden M, Buyken AE. Habitual Flavonoid Intake from Fruit and Vegetables during Adolescence and Serum Lipid Levels in Early Adulthood: A Prospective Analysis. Nutrients 2018;10(4):14. doi: <https://dx.doi.org/10.3390/nu10040488>.

84. Penczynski KJ, Herder C, Krupp D, Rienks J, Egert S, Wudy SA, Roden M, Remer T, Buyken AE. Flavonoid intake from fruit and vegetables during adolescence is prospectively associated with a favourable risk factor profile for type 2 diabetes in early adulthood. European Journal of Nutrition 2019;58(3):1159-72. doi: <https://dx.doi.org/10.1007/s00394-018-1631-3>.

85. Nakamoto M, Otsuka R, Nishita Y, Tange C, Tomida M, Kato Y, Imai T, Sakai T, Ando F, Shimokata H. Soy food and isoflavone intake reduces the risk of cognitive impairment in elderly Japanese women. European Journal of Clinical Nutrition 2018;72(10):1458-62. doi: 10.1038/s41430-017-0061-2.

86. Kent K, Charlton KE, Russell J, Mitchell P, Flood VM. Estimation of Flavonoid Intake in Older Australians: Secondary Data Analysis of the Blue Mountains Eye Study. J Nutr Gerontol Geriatr 2015;34(4):388-98. doi: <https://dx.doi.org/10.1080/21551197.2015.1088917>.

87. Kyro C, Zamora-Ros R, Scalbert A, Tjonneland A, Dossus L, Johansen C, Bidstrup PE, Weiderpass E, Christensen J, Ward H, et al. Pre-diagnostic polyphenol intake and breast cancer survival: the European Prospective Investigation into Cancer and Nutrition (EPIC) cohort. Breast Cancer Res Treat 2015;154(2):389-401. doi: <https://dx.doi.org/10.1007/s10549-015-3595-9>.

88. Molina-Montes E, Sanchez MJ, Zamora-Ros R, Bueno-de-Mesquita HB, Wark PA, Obon-Santacana M, Kuhn T, Katzke V, Travis RC, Ye WM, et al. Flavonoid and lignan intake and pancreatic cancer risk in the European prospective investigation into cancer and nutrition cohort. International Journal of Cancer 2016;139(7):1480-92. doi: 10.1002/ijc.30190.

89. Vermeulen E, Zamora-Ros R, Duell EJ, Lujan-Barroso L, Boeing H, Aleksandrova K, Bueno-de-Mesquita HB, Scalbert A, Romieu I, Fedirko V, et al. Dietary Flavonoid Intake and Esophageal Cancer Risk in the European Prospective Investigation into Cancer and Nutrition Cohort. American Journal of Epidemiology 2013;178(4):570-81. doi: 10.1093/aje/kwt026.

90. Zamora-Ros R, Agudo A, Lujan-Barroso L, Romieu I, Ferrari P, Knaze V, Bueno-de-Mesquita HB, Leenders M, Travis RC, Navarro C, et al. Dietary flavonoid and lignan intake and gastric adenocarcinoma risk in the European Prospective Investigation into Cancer and Nutrition (EPIC) study. American Journal of Clinical Nutrition 2012;96(6):1398-408. doi: 10.3945/ajcn.112.037358.

91. Zamora-Ros R. The association between dietary flavonoid and lignan intakes and incident type 2 diabetes in European populations: The EPIC-InterAct study. Diabetologia 2013;1):S160. doi: <http://dx.doi.org/10.1007/s00125-013-3012-z>.

92. Zamora-Ros R, Fedirko V, Trichopoulou A, Gonzalez CA, Bamia C, Trepo E, Nothlings U, Duarte-Salles T, Serafini M, Bredsdorff L, et al. Dietary flavonoid, lignan and antioxidant capacity and risk of hepatocellular carcinoma in the European prospective investigation into cancer and nutrition study. International Journal of Cancer 2013;133(10):2429-43. doi: 10.1002/ijc.28257.

93. Zamora-Ros R, Forouhi NG, Sharp SJ, Gonzalez CA, Buijsse B, Guevara M, van der Schouw YT, Amiano P, Boeing H, Bredsdorff L, et al. Dietary Intakes of Individual Flavanols and Flavonols Are Inversely Associated with Incident Type 2 Diabetes in European Populations. Journal of Nutrition 2014;144(3):335-43. doi: 10.3945/jn.113.184945.

94. Zamora-Ros R, Sacerdote C, Ricceri F, Weiderpass E, Roswall N, Buckland G, St-Jules DE, Overvad K, Kyro C, Fagherazzi G, et al. Flavonoid and lignan intake in relation to bladder cancer risk in the European Prospective Investigation into Cancer and Nutrition (EPIC) study. British Journal of Cancer 2014;111(9):1870-80. doi: <https://dx.doi.org/10.1038/bjc.2014.459>.

95. Zamora-Ros R, Barupal DK, Rothwell JA, Jenab M, Fedirko V, Romieu I, Aleksandrova K, Overvad K, Kyro C, Tjonneland A, et al. Dietary flavonoid intake and colorectal cancer risk in the European prospective investigation into cancer and nutrition (EPIC) cohort. International Journal of Cancer 2017;140(8):1836-44. doi: 10.1002/ijc.30582.

96. Zamora-Ros R, Rothwell JA, Achaintre D, Ferrari P, Boutron-Ruault MC, Mancini FR, Affret A, Kuhn T, Katzke V, Boeing H, et al. Evaluation of urinary resveratrol as a biomarker of dietary resveratrol intake in the European Prospective Investigation into Cancer and Nutrition (EPIC) study. British Journal of Nutrition 2017;117(11):1596-602. doi: 10.1017/s0007114517001465.

97. Zamora-Ros R, Cayssials V, Jenab M, Rothwell JA, Fedirko V, Aleksandrova K, Tjonneland A, Kyro C, Overvad K, Boutron-Ruault MC, et al. Dietary intake of total polyphenol and polyphenol classes and the risk of colorectal cancer in the European Prospective Investigation into Cancer and Nutrition (EPIC) cohort. European Journal of Epidemiology 2018;33(11):1063-75. doi: 10.1007/s10654-018-0408-6.

98. Zamora-Ros R, Cayssials V, Franceschi S, Kyro C, Weiderpass E, Hennings J, Sandstrom M, Tjonneland A, Olsen A, Overvad K, et al. Polyphenol intake and differentiated thyroid cancer risk in the European Prospective Investigation into Cancer and Nutrition (EPIC) cohort. International Journal of Cancer 2020;146(7):1841-50. doi: <https://dx.doi.org/10.1002/ijc.32589>.

99. Tahiri I, Garro-Aguilar Y, Cayssials V, Achaintre D, Mancini FR, Mahamat-Saleh Y, Boutron-Ruault MC, Kuhn T, Katzke V, Boeing H, et al. Urinary flavanone concentrations as biomarkers of dietary flavanone intakes in the European Prospective Investigation into Cancer and Nutrition (EPIC) study. British Journal of Nutrition 2020;123(6):691-8. doi: <http://dx.doi.org/10.1017/S0007114519003131>.

100. Nothlings U, Murphy SP, Wilkens LR, Boeing H, Schulze MB, Bueno-De-Mesquita HB, Michaud DS, Roddam A, Rohrmann S, Tjonneland A, et al. A food pattern that is predictive of flavonol intake and risk of pancreatic cancer. American Journal of Clinical Nutrition 2008;88(6):1653-62. doi: 10.3945/ajcn.2008.26398.

101. Lako J, Wattanapenpaiboon N, Wahlqvist M, Trenerry C. Phytochemical intakes of the Fijian population. Asia Pacific Journal of Clinical Nutrition 2006;15(2):275-85.

102. Lei B, Roncaglia V, Vigano R, Cremonini C, De Maria N, Del Buono MG, Manenti F, Villa E. Phytoestrogens and liver disease. Mol Cell Endocrinol 2002;193(1-2):81-4. doi: 10.1016/s0303-7207(02)00099-0.

103. Marniemi J, Alanen E, Impivaara O, Seppanen R, Hakala P, Rajala T, Ronnemaa T. Dietary and serum vitamins and minerals as predictors of myocardial infarction and stroke in elderly subjects. Nutr Metab Carbiovasc Dis 2005;15(3):188-97. doi: 10.1016/j.numced.2005.01.001.

104. Knekt P, Jarvinen R, Reunanen A, Maatela J. Flavonoid intake and coronary mortality in Finland: A cohort study. Br Med J 1996;312(7029):478-81. doi: 10.1136/bmj.312.7029.478.

105. Knekt P, Jarvinen R, Seppanen R, Heliovaara M, Teppo L, Pukkala E, Aromaa A. Dietary flavonoids and the risk of lung cancer and other malignant neoplasms. American Journal of Epidemiology 1997;146(3):223-30.

106. Knekt P, Isotupa S, Rissanen H, Heliovaara M, Jarvinen R, Hakkinen S, Aromaa A, Reunanen A. Quercetin intake and the incidence of cerebrovascular disease. European Journal of Clinical Nutrition 2000;54(5):415-7. doi: 10.1038/sj.ejcn.1600974.

107. Cui Y, Huang C, Momma H, Niu K, Nagatomi R. Daily dietary isoflavone intake in relation to lowered risk of depressive symptoms among men. J Affect Disord 2020;261:121-5. doi: <http://dx.doi.org/10.1016/j.jad.2019.10.001>.

108. Fukushima Y, Takahashi Y, Kishimoto Y, Taguchi C, Suzuki N, Yokoyama M, Kondo K. Consumption of Polyphenols in Coffee and Green Tea Alleviates Skin Photoaging in Healthy Japanese Women. Clin Cosmet Investig Dermatol 2020;13:165-72. doi: 10.2147/ccid.S225043.

109. Garcia R, Gonzalez CA, Agudo A, Riboli E. High intake of specific carotenoids and flavonoids does not reduce the risk of bladder cancer. Nutr Cancer 1999;35(2):212-4. doi: 10.1207/s15327914nc352_18.

110. Garcia-Closas R, Gonzalez CA, Agudo A, Riboli E. Intake of specific carotenoids and flavonoids and the risk of gastric cancer in Spain. Cancer Causes Control 1999;10(1):71-5. doi: 10.1023/a:1008867108960.

111. Taguchi C, Kishimoto Y, Kondo K, Tohyama K, Goda T. Serum gamma-glutamyltransferase is inversely associated with dietary total and coffee-derived polyphenol intakes in apparently healthy Japanese men. European Journal of Nutrition 2018;57(8):2819-26. doi: 10.1007/s00394-017-1549-1.

112. Zamora-Ros R, Guino E, Alonso MH, Vidal C, Barenys M, Soriano A, Moreno V. Dietary flavonoids, lignans and colorectal cancer prognosis. Sci Rep 2015;5:5. doi: 10.1038/srep14148.

113. Zamora-Ros R, Jimenez C, Cleries R, Agudo A, Sanchez MJ, Sanchez-Cantalejo E, Molina-Montes E, Navarro C, Chirlaque MD, Huerta JM, et al. Dietary Flavonoid and Lignan Intake and Mortality in a Spanish Cohort. Epidemiology 2013;24(5):726-33. doi: 10.1097/EDE.0b013e31829d5902.

114. Zamora-Ros R, Andres-Lacueva C, Lamuela-Raventos RM, Berenguer T, Jakszyn P, Martiez C, Sanchez MJ, Navarro C, Chirlaque MD, Tormo MJ, et al. Concentrations of resveratrol and derivatives in foods and estimation of dietary intake in a Spanish population: European Prospective Investigation into Cancer and Nutrition (EPIC)-Spain cohort. British Journal of Nutrition 2008;100(1):188-96. doi: <http://dx.doi.org/10.1017/S0007114507882997>.

115. Zamora-Ros R, Andres-Lacueva C, Lamuela-Raventos RM, Berenguer T, Jakszyn P, Barricarte A, Ardanaz E, Amiano P, Dorronsoro M, Larranaga N, et al. Estimation of Dietary Sources and Flavonoid Intake in a Spanish Adult Population (EPIC-Spain). J Am Diet Assoc 2010;110(3):390-8. doi: 10.1016/j.jada.2009.11.024.

116. Miyake Y, Tanaka K, Okubo H, Sasaki S, Furukawa S, Arakawa M. Soy isoflavone intake and prevalence of depressive symptoms during pregnancy in Japan: baseline data from the Kyushu Okinawa Maternal and Child Health Study. European Journal of Nutrition 2018;57(2):441-50. doi: 10.1007/s00394-016-1327-5.

117. Zamora-Ros R, Not C, Guino E, Lujan-Barroso L, Garcia RM, Biondo S, Salazar R, Moreno V. Association between habitual dietary flavonoid and lignan intake and colorectal cancer in a Spanish case-control study (the Bellvitge Colorectal Cancer Study). Cancer Causes Control 2013;24(3):549-57. doi: 10.1007/s10552-012-9992-z.

118. Lewis JE, Soler-Vila H, Clark PE, Kresty LA, Allen GO, Hu JJ. Intake of Plant Foods and Associated Nutrients in Prostate Cancer Risk. Nutr Cancer 2009;61(2):216-24. doi: 10.1080/01635580802419756.

119. Abulimiti A, Zhang X, Shivappa N, Hebert JR, Fang YJ, Huang CY, Feng XL, Chen YM, Zhang CX. The dietary inflammatory index is positively associated with colorectal cancer risk in a Chinese case-control study. Nutrients 2020;12(1). doi: <http://dx.doi.org/10.3390/nu12010232>.

120. Alipour B, Rashidkhani B, Edalati S. Dietary flavonoid intake, total antioxidant capacity and lipid oxidative damage: A cross-sectional study of Iranian women. Nutrition 2016;32(5):566-72. doi: 10.1016/j.nut.2015.11.011.

121. Atkinson C, Skor HE, Fitzgibbons ED, Scholes D, Chen C, Wahala K, Schwartz SM, Lampe JW. Overnight urinary isoflavone excretion in a population of women living in the United States, and its relationship to isoflavone intake.[Erratum appears in Cancer Epidemiol Biomarkers Prev 2002 Nov;11(11):1511]. Cancer Epidemiol Biomarkers Prev 2002;11(3):253-60.

122. Bahrami A, Jafari S, Rafiei P, Beigrezaei S, Sadeghi A, Hekmatdoost A, Rashidkhani B, Hejazi E. Dietary intake of polyphenols and risk of colorectal cancer and adenoma-A case-control study from Iran. Complement Ther Med 2019;45:269-74. doi: <https://dx.doi.org/10.1016/j.ctim.2019.04.011>.

123. Bobe G, Peterson JJ, Gridley G, Hyer M, Dwyer JT, Brown LM. Flavonoid consumption and esophageal cancer among black and white men in the United States. International Journal of Cancer 2009;125(5):1147-54. doi: 10.1002/ijc.24421.

124. Bosetti C, Spertini L, Parpinel M, Gnagnarella P, Lagiou P, Negri E, Franceschi S, Montella M, Peterson J, Dwyer J, et al. Flavonoids and breast cancer risk in Italy. Cancer Epidemiol Biomarkers Prev 2005;14(4):805-8. doi: 10.1158/1055-9965.Epi-04-0838.

125. Bosetti C, Bravi F, Talamini R, Parpinel M, Gnagnarella P, Negri E, Montella M, Lagiou P, Franceschi S, La Vecchia C. Flavonoids and prostate cancer risk: A study in Italy. Nutr Cancer 2006;56(2):123-7. doi: 10.1207/s15327914nc5602_1.

126. Chan YH, Lau KK, Yiu KH, Li SW, Chan HT, Tam S, Shu XO, Lau CP, Tse HF. Isoflavone intake in persons at high risk of cardiovascular events: Implications for vascular endothelial function and the carotid atherosclerotic burden. American Journal of Clinical Nutrition 2007;86(4):938-45.

127. Chan R, Woo J, Leung J. EFFECTS OF FOOD GROUPS AND DIETARY NUTRIENTS ON BONE LOSS IN ELDERLY CHINESE POPULATION. J Nutr Health Aging 2011;15(4):287-94. doi: 10.1007/s12603-010-0279-3.

128. Chan YH, Lam TH, Lau KK, Yiu KH, Siu CW, Li SW, Chan HT, Tam S, Lau CP, Tse HF. Dietary intake of phytoestrogen is associated with increased circulating endothelial progenitor cells in patients with cardiovascular disease. Eur J Cardiovasc Prev Rehabil 2011;18(3):360-8. doi: <https://dx.doi.org/10.1177/1741826710389385>.

129. Chan YH, Lau KK, Yiu KH, Siu CW, Chan HT, Li SW, Tam S, Lam TH, Lau CP, Tse HF. Prospective observational study of isoflavone and the risk of stroke recurrence: potential clinical implications beyond vascular function. J Nutr Health Aging 2012;16(4):383-8.

130. Cui Y, Morgenstern H, Greenland S, Tashkin DP, Mao JT, Cai L, Cozen W, Mack TM, Lu QY, Zhang ZF. Dietary flavonoid intake and lung cancer - A population-based case-control study. Cancer 2008;112(10):2241-8. doi: 10.1002/cncr.23398.

131. Cui YF, Niu K, Huang C, Momma H, Guan L, Kobayashi Y, Guo H, Chujo M, Otomo A, Nagatomi R. Relationship between daily isoflavone intake and sleep in Japanese adults: a cross-sectional study. Nutr J 2015;14:7. doi: 10.1186/s12937-015-0117-x.

132. De Stefani E, Boffetta P, Deneo-Pellegrini H, Mendilaharsu M, Carzoglio JC, Ronco A, Olivera L. Dietary antioxidants and lung cancer risk: A case-control study in Uruguay. Nutr Cancer 1999;34(1):100-10. doi: 10.1207/s15327914nc340114.

133. Djuric Z, Severson RK, Kato I. Association of Dietary Quercetin With Reduced Risk of Proximal Colon Cancer. Nutr Cancer 2012;64(3):351-60. doi: 10.1080/01635581.2012.658950.

134. Ekstrom AM, Serafini M, Nyren O, Wolk A, Bosetti C, Bellocco R. Dietary quercetin intake and risk of gastric cancer: results from a population-based study in Sweden. Ann Oncol 2011;22(2):438-43. doi: 10.1093/annonc/mdq390.

135. Endoh K, Kuriki K, Kasezawa N, Tohyama K, Goda T. Association between Smoking Status and Food and Nutrient Consumption in Japanese: a Large-Scale Cross-Sectional Study. Asian Pac J Cancer Prev 2015;16(15):6527-34.

136. Feng XL, Ho SC, Mo XF, Lin FY, Zhang NQ, Luo H, Zhang X, Zhang CX. Association between flavonoids, flavonoid subclasses intake and breast cancer risk: a case-control study in China. European journal of cancer prevention : the official journal of the European Cancer Prevention Organisation 2019;14. doi: <http://dx.doi.org/10.1097/CEJ.0000000000000561>.

137. Fernandez-Navarro T, Salazar N, Gutierrez-Diaz I, Sanchez B, Ruas-Madiedo P, de Los Reyes-Gavilan CG, Margolles A, Gueimonde M, Gonzalez S. Bioactive compounds from regular diet and faecal microbial metabolites. European Journal of Nutrition 2018;57(2):487-97. doi: <https://dx.doi.org/10.1007/s00394-016-1332-8>.

138. Ferreira LL, Silva TR, Maturana MA, Spritzer PM. Dietary intake of isoflavones is associated with a lower prevalence of subclinical cardiovascular disease in postmenopausal women: cross-sectional study. J Hum Nutr Diet 2019;32(6):810-8. doi: 10.1111/jhn.12683.

139. Fisher ND, Hurwitz S, Hollenberg NK. Habitual flavonoid intake and endothelial function in healthy humans. Journal of the American College of Nutrition 2012;31(4):275-9.

140. Frankenfeld CL, Patterson RE, Kalhorn TF, Skor HE, Howald WN, Lampe JW. Validation of a soy food frequency questionnaire with plasma concentrations of isoflavones in US adults. J Am Diet Assoc 2002;102(10):1407-13. doi: 10.1016/s0002-8223(02)90313-5.

141. Fukushima Y, Takahashi Y, Hori Y, Kishimoto Y, Shiga K, Tanaka Y, Masunaga E, Tani M, Yokoyama M, Kondo K. Skin photoprotection and consumption of coffee and polyphenols in healthy middle-aged Japanese females. Int J Dermatol 2015;54(4):410-8. doi: 10.1111/ijd.12399.

142. Galvan-Portillo MV, Wolff MS, Torres-Sanchez LE, Lopez-Cervantes M, Lopez-Carrillo L. Assessing phytochemical intake in a group of Mexican women. Salud Publica Mexico 2007;49(2):126-31. doi: 10.1590/s0036-36342007000200008.

143. Garavello W, Rossi M, McLaughlin JK, Bosetti C, Negri E, Lagiou P, Talamini R, Franceschi S, Parpinel M, Dal Maso L, et al. Flavonoids and laryngeal cancer risk in Italy. Ann Oncol 2007;18(6):1104-9. doi: 10.1093/annonc/mdm078.

144. Garcia V, Arts ICW, Sterne JAC, Thompson RL, Shaheen SO. Dietary intake of flavonoids and asthma in adults. European Respiratory Journal 2005;26(3):449-52. doi: 10.1183/09031936.05.00142104.

145. Gates MA, Vitonis AF, Tworoger SS, Rosner B, Titus-Ernstow L, Hankinson SE, Cramer DW. Flavonoid intake and ovarian cancer risk in a population-based case-control study. International Journal of Cancer 2009;124(8):1918-25. doi: 10.1002/ijc.24151.

146. Gonzalez S, Fernandez M, Cuervo A, Lasheras C. Dietary intake of polyphenols and major food sources in an institutionalised elderly population. J Hum Nutr Diet 2014;27(2):176-83. doi: 10.1111/jhn.12058.

147. Hakim IA, Weisgerber UM, Harris RB, Balentine D, van-Mierlo CAJ, Paetau-Robinson I. Preparation, composition and consumption patterns of tea-based beverages in Arizona. Nutrition Research 2000;20(12):1715-24. doi: 10.1016/s0271-5317(00)00275-x.

148. Hardcastle AC, Aucott L, Reid DM, Macdonald HM. Associations Between Dietary Flavonoid Intakes and Bone Health in a Scottish Population. J Bone Miner Res 2011;26(5):941-7. doi: 10.1002/jbmr.285.

149. Hernandez-Ramirez RU, Galvan-Portillo MV, Ward MH, Agudo A, Gonzalez CA, Onate-Ocana LF, Herrera-Goepfert R, Palma-Coca O, Lopez-Carrillo L. Dietary intake of polyphenols, nitrate and nitrite and gastric cancer risk in Mexico City. International Journal of Cancer 2009;125(6):1424-30. doi: 10.1002/ijc.24454.

150. Hirayama F, Lee AH, Binns CW, Hiramatsu N, Mori M, Nishimura K. Dietary intake of isoflavones and polyunsaturated fatty acids associated with lung function, breathlessness and the prevalence of chronic obstructive pulmonary disease: Possible protective effect of traditional Japanese diet. Mol Nutr Food Res 2010;54(7):909-17. doi: 10.1002/mnfr.200900316.

151. Ho SC, Woo JLF, Leung SSF, Sham ALK, Lam TH, Janus ED. Intake of soy products is associated with better plasma lipid profiles in the Hong Kong Chinese population. Journal of Nutrition 2000;130(10):2590-3.

152. Ho SC, Woo J, Lam S, Chen Y, Sham A, Lau J. Soy protein consumption and bone mass in early postmenopausal Chinese women. Osteoporosis International 2003;14(10):835-42. doi: 10.1007/s00198-003-1453-9.

153. Horn-Ross PL, Barnes S, Lee M, Coward L, Mandel JE, Koo J, John EM, Smith M. Assessing phytoestrogen exposure in epidemiologic studies: development of a database (United States). Cancer Causes Control 2000;11(4):289-98. doi: 10.1023/a:1008995606699.

154. Horn-Ross PL, Lee M, John EM, Koo J. Sources of phytoestrogen exposure among non-Asian women in California, USA. Cancer Causes Control 2000;11(4):299-302. doi: 10.1023/a:1008968003575.

155. Horn-Ross PL, John EM, Canchola AJ, Stewart SL, Lee MM. Phytoestrogen intake and endometrial cancer risk. Journal of the National Cancer Institute 2003;95(15):1158-64. doi: 10.1093/jnci/djg015.

156. Hou YC, Chen LJ, Wu JH, Wu CC, Chang YJ, Chung KP. Is vegetarian diet associated with a lower risk of breast cancer in Taiwanese women? BMC public health 2017;17(1):800. doi: <http://dx.doi.org/10.1186/s12889-017-4819-1>.

157. Iwasaki M, Hamada GS, Nishimoto IN, Netto MM, Motola Jr J, Laginha FM, Kasuga Y, Yokoyama S, Onuma H, Nishimura H, et al. Dietary isoflavone intake and breast cancer risk in case-control studies in Japanese, Japanese Brazilians, and non-Japanese Brazilians. Breast Cancer Res Treat 2009;116(2):401-11. doi: <http://dx.doi.org/10.1007/s10549-008-0168-1>.

158. Iwasaki M, Hamada GS, Nishimoto IN, Netto MM, Motola Jr J, Laginha FM, Kasuga Y, Yokoyama S, Onuma H, Nishimura H, et al. Dietary isoflavone intake, polymorphisms in the CYP17, CYP19, 17-HSD1, and SHBG genes, and risk of breast cancer in case-control studies in Japanese, Japanese Brazilians, and Non-Japanese Brazilians. Nutrition and Cancer 2010;62(4):466-75. doi: <http://dx.doi.org/10.1080/01635580903441279>.

159. Iwasaki M, Mizusawa J, Kasuga Y, Yokoyama S, Onuma H, Nishimura H, Kusama R, Tsugane S. Green Tea Consumption and Breast Cancer Risk in Japanese Women: A Case-Control Study. Nutr Cancer 2014;66(1):57-67. doi: 10.1080/01635581.2014.847963.

160. Jiao L, Kramer JR, Rugge M, Parente P, Verstovsek G, Alsarraj A, El-Serag HB. Dietary intake of vegetables, folate, and antioxidants and the risk of Barrett's esophagus. Cancer Causes Control 2013;24(5):1005-14. doi: 10.1007/s10552-013-0175-3.

161. Kim HW, Kwon MK, Kim NS, Reame NE. Intake of dietary soy isoflavones in relation to perimenstrual symptoms of Korean women living in the USA. Nurs Health Sci 2006;8(2):108-13.

162. Kreijkamp-Kaspers S, Kok L, Bots ML, Grobbee DE, van der Schouw YT. Dietary phytoestrogens and vascular function in postmenopausal women: a cross-sectional study. J Hypertens 2004;22(7):1381-8. doi: 10.1097/01.hjh.0000125435.28861.d2.

163. Lammersfeld CA, King J, Walker S, Vashi PG, Grutsch JF, Lis CG, Gupta D. Prevalence, sources, and predictors of soy consumption in breast cancer. Nutr J 2009;8:7. doi: 10.1186/1475-2891-8-2.

164. Lee MM, Gomez SL, Chang JS, Wey M, Wang RT, Hsing AW. Soy and isoflavone consumption in relation to prostate cancer risk in China. Cancer Epidemiol Biomarkers Prev 2003;12(7):665-8.

165. Lee SA, Choi JY, Shin CS, Hong YC, Chung H, Kang D. SULT1E1 genetic polymorphisms modified the association between phytoestrogen consumption and bone mineral density in healthy Korean women. Calcif Tissue Int 2006;79(3):152-9.

166. Lee AH, Su DD, Pasalich M, Tang L, Binns CW, Qiu LQ. Soy and isoflavone intake associated with reduced risk of ovarian cancer in southern Chinese women. Nutrition Research 2014;34(4):302-7. doi: 10.1016/j.nutres.2014.02.005.

167. Lee S, Quiambao AL, Lee J, Ro J, Lee ES, Jung SY, Sung MK, Kim J. Dietary inflammatory index and risk of breast cancer based on hormone receptor status: A case-control study in Korea. Nutrients 2019;11(8). doi: <http://dx.doi.org/10.3390/nu11081949>.

168. Levi F, Pasche C, Lucchini F, Ghidoni R, Ferraroni M, La Vecchia C. Resveratrol and breast cancer risk. Eur J Cancer Prev 2005;14(2):139-42. doi: 10.1097/00008469-200504000-00009.

169. Li GL, Zhu YN, Zhang Y, Lang J, Chen YM, Ling WH. Estimated Daily Flavonoid and Stilbene Intake from Fruits, Vegetables, and Nuts and Associations with Lipid Profiles in Chinese Adults. J Acad Nutr Diet 2013;113(6):786-94. doi: 10.1016/j.jand.2013.01.018.

170. Li L, Zhang M, Holman CDJ. Population Versus Hospital Controls in the Assessment of Dietary Intake of Isoflavone for Case-Control Studies on Cancers in China. Nutr Cancer 2013;65(3):390-7. doi: 10.1080/01635581.2013.767915.

171. Lin YL, Yngve A, Lagergren J, Lu YX. Dietary intake of lignans and risk of adenocarcinoma of the esophagus and gastroesophageal junction. Cancer Causes Control 2012;23(6):837-44. doi: 10.1007/s10552-012-9952-7.

172. Lin Y, Yngve A, Lagergren J, Lu Y. A dietary pattern rich in lignans, quercetin and resveratrol decreases the risk of oesophageal cancer. British Journal of Nutrition 2014;112(12):2002-9. doi: <https://dx.doi.org/10.1017/S0007114514003055>.

173. Liu YT, Fan YY, Xu CH, Lin XL, Lu YK, Zhang XL, Zhang CX, Chen YM. Habitual Consumption of Soy Products and Risk of Nasopharyngeal Carcinoma in Chinese Adults: A Case-Control Study. PLoS One 2013;8(10):8. doi: 10.1371/journal.pone.0077822.

174. Liu P, Holman CD, Jin J, Zhang M. Dietary isoflavone intake is associated with a reduced risk of myelodysplastic syndromes. British Journal of Nutrition 2015;114(12):2110-5. doi: <https://dx.doi.org/10.1017/S0007114515003931>.

175. Lu Y, Shivappa N, Lin Y, Lagergren J, Hebert JR. Diet-related inflammation and oesophageal cancer by histological type: a nationwide case-control study in Sweden. European Journal of Nutrition 2016;55(4):1683-94. doi: <http://dx.doi.org/10.1007/s00394-015-0987-x>.

176. Luo D, Liu Y, Zhou Y, Chen Z, Yang L, Liu Y, Xu Q, Xu H, Kuang H, Huang Q, et al. Association between dietary phytoestrogen intake and bone mineral density varied with estrogen receptor alpha gene polymorphisms in southern Chinese postmenopausal women. Food Funct 2015;6(6):1977-83. doi: <https://dx.doi.org/10.1039/c5fo00295h>.

177. Ma YN, Gao WQ, Wu K, Bao YP. Flavonoid intake and the risk of age-related cataract in China's Heilongjiang Province. Food Nutr Res 2015;59:9. doi: 10.3402/fnr.v59.29564.

178. Merida-Ortega A, Hernandez-Alcaraz C, Hernandez-Ramirez RU, Garcia-Martinez A, Trejo-Valdivia B, Salinas-Rodriguez A, Svensson K, Cebrian ME, Franco-Marina F, Lopez-Carrillo L. Phthalate exposure, flavonoid consumption and breast cancer risk among Mexican women. Environ Int 2016;96:167-72. doi: <https://dx.doi.org/10.1016/j.envint.2016.08.023>.

179. Nagata C, Shimizu H, Takami R, Hayashi M, Takeda N, Yasuda K. Soy product intake and serum isoflavonoid and estradiol concentrations in relation to bone mineral density in postmenopausal Japanese women. Osteoporosis International 2002;13(3):200-4. doi: 10.1007/s001980200014.

180. Nagata C, Shimizu H, Takami R, Hayashi M, Takeda N, Yasuda K. Soy product intake is inversely associated with serum homocysteine level in premenopausal Japanese women. Journal of Nutrition 2003;133(3):797-800.

181. Nagata Y, Sonoda T, Mori M, Miyanaga N, Okumura K, Goto K, Naito S, Fujimoto K, Hirao Y, Takahashi A, et al. Dietary isoflavones may protect against prostate cancer in Japanese men. Journal of Nutrition 2007;137(8):1974-9.

182. Nagata C, Ueno T, Uchiyama S, Nagao Y, Yamamoto S, Shibuya C, Kashiki Y, Shimizu H. Dietary and lifestyle correlates of urinary excretion status of equol in Japanese women. Nutr Cancer 2008;60(1):49-54. doi: 10.1080/01635580701525885.

183. Nagata C, Nakamura K, Oba S, Hayashi M, Takeda N, Yasuda K. Association of intakes of fat, dietary fibre, soya isoflavones and alcohol with uterine fibroids in Japanese women. British Journal of Nutrition 2009;101(10):1427-31. doi: 10.1017/s0007114508083566.

184. Nagata Y, Sugiyama Y, Fukuta F, Takayanagi A, Masumori N, Tsukamoto T, Akasaka H, Ohnishi H, Saitoh S, Miura T, et al. Relationship of serum levels and dietary intake of isoflavone, and the novel bacterium Slackia sp strain NATTS with the risk of prostate cancer: a case-control study among Japanese men. Int Urol Nephrol 2016;48(9):1453-60. doi: 10.1007/s11255-016-1335-7.

185. Nakamoto M, Shuto E, Nakamoto A, Hata A, Aki N, Shikama Y, Bando Y, Ichihara T, Minagawa T, Tamura A, et al. Soy product and isoflavone intake associations with allergic diseases in Japanese workers: rhinitis, dermatitis and asthma. Asia Pacific journal of clinical nutrition 2018;27(6):1277-85. doi: <http://dx.doi.org/10.6133/apjcn.201811_27%286%29.0015>.

186. Nguyen CT, Pham NM, Do VV, Binns CW, Hoang VM, Dang DA, Lee AH. Soyfood and isoflavone intake and risk of type 2 diabetes in Vietnamese adults. European Journal of Clinical Nutrition 2017;71(10):1186-92. doi: <https://dx.doi.org/10.1038/ejcn.2017.76>.

187. Oh JS, Kim H, Vijayakumar A, Kwon O, Choi YJ, Huh KB, Chang N. Association between dietary flavanones intake and lipid profiles according to the presence of metabolic syndrome in Korean women with type 2 diabetes mellitus. Nutr Res Pract 2016;10(1):67-73. doi: 10.4162/nrp.2016.10.1.67.

188. Ohfuji S, Fukushima W, Watanabe K, Sasaki S, Yamagami H, Nagahori M, Watanabe M, Hirota Y, Japanese Case-Control Study Group for Ulcerative C. Pre-illness isoflavone consumption and disease risk of ulcerative colitis: a multicenter case-control study in Japan. PLoS ONE 2014;9(10):e110270. doi: <https://dx.doi.org/10.1371/journal.pone.0110270>.

189. Ponzo V, Goitre I, Fadda M, Gambino R, De Francesco A, Soldati L, Gentile L, Magistroni P, Cassader M, Bo S. Dietary flavonoid intake and cardiovascular risk: a population-based cohort study. J Transl Med 2015;13:13. doi: 10.1186/s12967-015-0573-2.

190. Quiller G, Merida-Ortega A, Rothenberg SJ, Cebrian ME, Gandolfi AJ, Franco-Marina F, Lopez-Carrillo L. Dietary flavonoids improve urinary arsenic elimination among Mexican women. Nutrition Research 2018;55:65-71. doi: 10.1016/j.nutres.2018.04.012.

191. Ranka S, Gee JM, Biro L, Brett G, Saha S, Kroon P, Skinner J, Hart AR, Cassidy A, Rhodes M, et al. Development of a food frequency questionnaire for the assessment of quercetin and naringenin intake. European Journal of Clinical Nutrition 2008;62(9):1131-8. doi: 10.1038/sj.ejcn.1602827.

192. Reale G, Russo GI, Di Mauro M, Regis F, Campisi D, Giudice AL, Marranzano M, Ragusa R, Castelli T, Cimino S, et al. Association between dietary flavonoids intake and prostate cancer risk: A case-control study in Sicily. Complement Ther Med 2018;39:14-8. doi: <https://dx.doi.org/10.1016/j.ctim.2018.05.002>.

193. Reed SD, Lampe JW, Qu C, Gundersen G, Fuller S, Copeland WK, Newton KM. Self-reported menopausal symptoms in a racially diverse population and soy food consumption. Maturitas 2013;75(2):152-8. doi: 10.1016/j.maturitas.2013.03.003.

194. Ronco AL, Stefani ED, Mendoza B, Vazquez A, Abbona E, Sanchez G, Rosa AD. Mate and Tea Intake, Dietary Antioxidants and Risk of Breast Cancer: a Case-Control Study. Asian Pac J Cancer Prev 2016;17(6):2923-33.

195. Rosli H, Kee Y, Shahar S. Dietary polyphenol intake associated with adiposity indices among adults from low to medium socioeconomic status in a suburban area of Kuala Lumpur: A preliminary findings. Malaysian Journal of Medical Sciences 2019;26(6):67-76. doi: <http://dx.doi.org/10.21315/mjms2019.26.6.7>.

196. Rossi M, Negri E, Talamini R, Bosetti C, Parpinel M, Gnagnarella P, Franceschi S, Dal Maso L, Montella M, Giacosa A, et al. Flavonoids and colorectal cancer in Italy. Cancer Epidemiol Biomarkers Prev 2006;15(8):1555-8. doi: 10.1158/1055-9965.Epi-06-0017.

197. Rossi M, Garavello W, Talamini R, La Vecchia C, Franceschi S, Lagiou P, Zambon P, Dal Maso L, Bosetti C, Negri E. Flavonoids and risk of squamous cell esophageal cancer. International Journal of Cancer 2007;120(7):1560-4. doi: 10.1002/ijc.22499.

198. Rossi M, Garavello W, Talamini R, Negri E, Bosetti C, Dal Maso L, Lagiou P, Tavani A, Polesel J, Barzan L, et al. Flavonoids and the risk of oral and pharyngenal cancer: A case-control study from Italy. Cancer Epidemiol Biomarkers Prev 2007;16(8):1621-5. doi: 10.1158/1055-9965.Epi-07-0168.

199. Rossi M, Negri E, Lagiou P, Talamini R, Dal Maso L, Montella M, Franceschi S, La Vecchia C. Flavonoids and ovarian cancer risk: A case-control study in Italy. International Journal of Cancer 2008;123(4):895-8. doi: 10.1002/ijc.23549.

200. Rossi M, Negri E, Parpinel M, Lagiou P, Bosetti C, Talamini R, Montella M, Attilio G, Franceschi S, Vecchia CL. Proanthocyanidins and the risk of colorectal cancer in Italy. Cancer Causes and Control 2010;21(2):243-50. doi: <http://dx.doi.org/10.1007/s10552-009-9455-3>.

201. Rossi M, Rosato V, Bosetti C, Lagiou P, Parpinel M, Bertuccio P, Negri E, La Vecchia C. Flavonoids, proanthocyanidins, and the risk of stomach cancer. Cancer Causes Control 2010;21(10):1597-604. doi: 10.1007/s10552-010-9588-4.

202. Rossi M, Lugo A, Lagiou P, Zucchetto A, Polesel J, Serraino D, Negri E, Trichopoulos D, La Vecchia C. Proanthocyanidins and other flavonoids in relation to pancreatic cancer: a case-control study in Italy. Ann Oncol 2012;23(6):1488-93. doi: 10.1093/annonc/mdr475.

203. Rossi M, Edefonti V, Parpinel M, Lagiou P, Franchi M, Ferraroni M, Decarli A, Zucchetto A, Serraino D, Dal Maso L, et al. Proanthocyanidins and other flavonoids in relation to endometrial cancer risk: a case-control study in Italy. British Journal of Cancer 2013;109(7):1914-20. doi: 10.1038/bjc.2013.447.

204. Rossi M, Strikoudi P, Spei ME, Parpinel M, Serraino D, Montella M, Libra M, La Vecchia C, Rosato V. Flavonoids and bladder cancer risk. Cancer Causes Control 2019;30(5):527-35. doi: 10.1007/s10552-019-01158-2.

205. Russo GI, Campisi D, Di Mauro M, Regis F, Reale G, Marranzano M, Ragusa R, Solinas T, Madonia M, Cimino S, et al. Dietary Consumption of Phenolic Acids and Prostate Cancer: A Case-Control Study in Sicily, Southern Italy. Molecules 2017;22(12):9. doi: 10.3390/molecules22122159.

206. Russo GI, Di Mauro M, Regis F, Reale G, Campisi D, Marranzano M, Lo Giudice A, Solinas T, Madonia M, Cimino S, et al. Association between dietary phytoestrogens intakes and prostate cancer risk in Sicily. Aging Male 2018;21(1):48-54. doi: 10.1080/13685538.2017.1365834.

207. Salomone F, Ivancovsky-Wajcman D, Fliss-Isakov N, Webb M, Grosso G, Godos J, Galvano F, Shibolet O, Kariv R, Zelber-Sagi S. Higher phenolic acid intake independently associates with lower prevalence of insulin resistance and non-alcoholic fatty liver disease. JHEP Reports 2020;2(2). doi: <http://dx.doi.org/10.1016/j.jhepr.2020.100069>.

208. Schabath MB, Hernandez LM, Wu XF, Pillow PC, Spitz MR. Dietary phytoestrogens and lung cancer risk. JAMA-J Am Med Assoc 2005;294(12):1493-504. doi: 10.1001/jama.294.12.1493.

209. Seow A, Poh WT, Teh M, Eng P, Wang YT, Tan WC, Chia KS, Yu MC, Lee HP. Diet, reproductive factors and lung cancer risk among Chinese women in Singapore: Evidence for a protective effect of soy in nonsmokers. International Journal of Cancer 2002;97(3):365-71. doi: 10.1002/ijc.1615.

210. Shin A, Lee J, Lee J, Park MS, Park JW, Park SC, Oh JH, Kim J. Isoflavone and Soyfood Intake and Colorectal Cancer Risk: A Case-Control Study in Korea. PLoS One 2015;10(11):17. doi: 10.1371/journal.pone.0143228.

211. Silva IDS, Mangtani P, McCormack V, Bhakta D, McMichael AJ, Sevak L. Phyto-oestrogen intake and breast cancer risk in South Asian women in England: Findings from a population-based case-control study. Cancer Causes and Control 2004;15(8):805-18. doi: <http://dx.doi.org/10.1023/B:CACO.0000043431.85706.d8>.

212. Sonoda T, Suzuki H, Mori M, Tsukamoto T, Yokomizo A, Naito S, Fujimoto K, Hirao Y, Miyanaga N, Akaza H. Polymorphisms in estrogen related genes may modify the protective effect of isoflavones against prostate cancer risk in Japanese men. Eur J Cancer Prev 2010;19(2):131-7. doi: 10.1097/CEJ.0b013e328333fbe2.

213. Strom SS, Yamamura Y, Duphorne CM, Spitz MR, Babaian RJ, Pillow PC, Hursting SD. Phytoestrogen intake and prostate cancer: A case-control study using a new database. Nutr Cancer 1999;33(1):20-5. doi: 10.1080/01635589909514743.

214. Sut A, Chizynski K, Rozalski M, Golanski J. Dietary intake of omega fatty acids and polyphenols and its relationship with the levels of inflammatory markers in men with chronic coronary syndrome after percutaneous coronary intervention. Kardiologia Polska 2020;78(2):117-23. doi: <http://dx.doi.org/10.33963/KP.15078>.

215. Taborelli M, Polesel J, Parpinel M, Stocco C, Birri S, Serraino D, Zucchetto A. Fruit and vegetables consumption is directly associated to survival after prostate cancer. Mol Nutr Food Res 2017;61(4):8. doi: 10.1002/mnfr.201600816.

216. Takata Y, Maskarinec G, Franke A, Nagata C, Shimizu H. A comparison of dietary habits among women in Japan and Hawaii. Public Health Nutrition 2004;7(2):319-26. doi: 10.1079/phn2003531.

217. Tang L, Lee AH, Xu FL, Zhang TT, Lei J, Binns CW. Soya and isoflavone intakes associated with reduced risk of oesophageal cancer in north-west China. Public Health Nutrition 2015;18(1):130-4. doi: 10.1017/s1368980013003443.

218. Tavani A, Spertini L, Bosetti C, Parpinel M, Gnagnarella P, Bravi F, Peterson J, Dwyer J, Lagiou P, Negri E, et al. Intake of specific flavonoids and risk of acute myocardial infarction in Italy. Public Health Nutrition 2006;9(3):369-74. doi: 10.1079/phn2006859.

219. Toi M, Hirota S, Tomotaki A, Sato N, Hozumi Y, Anan K, Nagashima T, Tokuda Y, Masuda N, Ohsumi S, et al. Probiotic beverage with soy isoflavone consumption for breast cancer prevention: A case-control study. Current Nutrition and Food Science 2013;9(3):194-200. doi: <http://dx.doi.org/10.2174/15734013113099990001>.

220. Torres-Sanchez L, Galvan-Portillo M, Wolff MS, Lopez-Carrillo L. Dietary consumption of phytochemicals and breast cancer risk in Mexican women. Public Health Nutrition 2009;12(6):825-31. doi: 10.1017/s136898000800325x.

221. Tseng M, Olufade T, Kurzer MS, Wahala K, Fang CY, van der Schouw YT, Daly MB. Food Frequency Questionnaires and Overnight Urines Are Valid Indicators of Daidzein and Genistein Intake in US Women Relative to Multiple 24-h Urine Samples. Nutr Cancer 2008;60(5):619-26. doi: 10.1080/01635580801993751.

222. Walcott FL, Hauptmann M, Duphorne CM, Pillow PC, Strom SS, Sigurdson AJ. A case-control study of dietary phytoestrogens and testicular cancer risk. Nutr Cancer 2002;44(1):44-51. doi: 10.1207/s15327914nc441_6.

223. Wang Q, Wang YP, Li JY, Yuan P, Yang F, Li H. Polymorphic catechol-O-methyltransferase gene, soy isoflavone intake and breast cancer in postmenopausal women: a case-control study. Chin 2010;29(7):683-8.

224. Wang Q, Huang H, Zhao N, Ni X, Udelsman R, Zhang YW. Phytoestrogens and Thyroid Cancer Risk: A Population-Based Case-Control Study in Connecticut. Cancer Epidemiol Biomarkers Prev 2020;29(2):500-8. doi: 10.1158/1055-9965.Epi-19-0456.

225. Wong SYS, Lau WWY, Leung PC, Leung JCS, Woo J. The association between isoflavone and lower urinary tract symptoms in elderly men. British Journal of Nutrition 2007;98(6):1237-42. doi: 10.1017/s0007114507787433.

226. Woo J, Lynn H, Lau WY, Leung J, Lau E, Wong SYS, Kwok T. Nutrient intake and psychological health in an elderly Chinese population. Int J Geriatr Psychiatr 2006;21(11):1036-43. doi: 10.1002/gps.1603.

227. Woo HD, Lee J, Choi IJ, Kim CG, Lee JY, Kwon O, Kim J. Dietary Flavonoids and Gastric Cancer Risk in a Korean Population. Nutrients 2014;6(11):4961-73. doi: 10.3390/nu6114961.

228. Wu AH, Wan P, Hankin J, Tseng CC, Yu MC, Pike MC. Adolescent and adult soy intake and risk of breast cancer in Asian-Americans. Carcinogenesis 2002;23(9):1491-6. doi: 10.1093/carcin/23.9.1491.

229. Wu AH, Yu MC, Tseng CC, Twaddle NC, Doerge DR. Plasma isoflavone levels versus self-reported soy isoflavone levels in Asian-American women in Los Angeles County. Carcinogenesis 2004;25(1):77-81. doi: 10.1093/carcin/bgg189.

230. Xu WH, Zheng W, Xiang YB, Ruan ZM, Cheng JR, Dai Q, Gao YT, Shu XO. Soya food intake and risk of endometrial cancer among Chinese women in Shanghai: population based case-control study. BMJ-British Medical Journal 2004;328(7451):1285-8. doi: 10.1136/bmj.38093.646215.AE.

231. Xu M, Chen YM, Huang J, Fang YJ, Huang WQ, Yan B, Lu MS, Pan ZZ, Zhang CX. Flavonoid intake from vegetables and fruits is inversely associated with colorectal cancer risk: a case-control study in China. British Journal of Nutrition 2016;116(7):1275-87. doi: 10.1017/s0007114516003196.

232. Yang X, Nakamoto M, Shuto E, Hata A, Aki N, Shikama Y, Bando Y, Ichihara T, Minamigawa T, Kuwamura Y, et al. Associations between intake of dietary fermented soy food and concentrations of inflammatory markers : a cross-sectional study in Japanese workers. J Med Investig 2018;65(1-2):74-80.

233. Yochum L, Kushi LH, Meyer K, Folsom AR. Dietary flavonoid intake and risk of cardiovascular disease in postmenopausal women.[Erratum appears in Am J Epidemiol 1999 Aug 15;150(4):432]. American Journal of Epidemiology 1999;149(10):943-9.

234. Zhang M, Xie X, Lee AH, Binns CW. Soy and isoflavone intake are associated with reduced risk of ovarian cancer in Southeast China. Nutr Cancer 2004;49(2):125-30. doi: 10.1207/s15327914nc4902_2.

235. Zhang M, Yang H, Holman CD. Dietary intake of isoflavones and breast cancer risk by estrogen and progesterone receptor status. Breast Cancer Res Treat 2009;118(3):553-63. doi: <https://dx.doi.org/10.1007/s10549-009-0354-9>.

236. Zhang CX, Ho SC, Lin FY, Cheng SZ, Fu JH, Chen YM. Soy product and isoflavone intake and breast cancer risk defined by hormone receptor status. Cancer Sci 2010;101(2):501-7. doi: 10.1111/j.1349-7006.2009.01376.x.

237. Zhang M, Liu X, Holman CDJ. Effect of dietary intake of isoflavones on the estrogen and progesterone receptor status of breast cancer. Nutrition and Cancer 2010;62(6):765-73. doi: <http://dx.doi.org/10.1080/01635581003605979>.

238. Zhang YF, Kang HB, Li BL, Zhang RM. Positive Effects of Soy Isoflavone Food on Survival of Breast Cancer Patients in China. Asian Pac J Cancer Prev 2012;13(2):479-82. doi: 10.7314/apjcp.2012.13.1.479.

239. Zhang ZQ, He LP, Liu YH, Liu J, Su YX, Chen YM. Association between dietary intake of flavonoid and bone mineral density in middle aged and elderly Chinese women and men. Osteoporosis International 2014;25(10):2417-25. doi: <https://dx.doi.org/10.1007/s00198-014-2763-9>.

240. Zhu YY, Zhou L, Jiao SC, Xu LZ. Relationship Between Soy Food Intake and Breast Cancer in China. Asian Pac J Cancer Prev 2011;12(11):2837-40.

241. Woo HW, Kim MK, Lee YH, Shin DH, Shin MH, Choi BY. Habitual consumption of soy protein and isoflavones and risk of metabolic syndrome in adults >= 40 years old: a prospective analysis of the Korean Multi-Rural Communities Cohort Study (MRCohort). European Journal of Nutrition 2018. doi: <http://dx.doi.org/10.1007/s00394-018-1833-8>.

242. Burkholder-Cooley N, Rajaram S, Haddad E, Fraser GE, Jaceldo-Siegl K. Comparison of polyphenol intakes according to distinct dietary patterns and food sources in the Adventist Health Study-2 cohort. British Journal of Nutrition 2016;115(12):2162-9. doi: 10.1017/s0007114516001331.

243. Burkholder-Cooley NM, Rajaram SS, Haddad EH, Oda K, Fraser GE, Jaceldo-Siegl K. Validating polyphenol intake estimates from a food-frequency questionnaire by using repeated 24-h dietary recalls and a unique method-of-triads approach with 2 biomarkers. American Journal of Clinical Nutrition 2017;105(3):685-94. doi: <http://dx.doi.org/10.3945/ajcn.116.137174>.

244. Fraser GE, Jaceldo-Siegl K, Henning SM, Fan J, Knutsen SF, Haddad EH, Sabate J, Lawrence Beeson W, Bennett H. Biomarkers of dietary intake are correlated with corresponding measures from repeated dietary recalls and food-frequency questionnaires in the Adventist Health Study-2. Journal of Nutrition 2016;146(3):586-94. doi: <http://dx.doi.org/10.3945/jn.115.225508>.

245. Jacobsen BK, Jaceldo-Siegl K, Knutsen SF, Fan J, Oda K, Fraser GE. Soy isoflavone intake and the likelihood of ever becoming a mother: The adventist health study-2. International Journal of Women's Health 2014;6(1):377-84. doi: <http://dx.doi.org/10.2147/IJWH.S57137>.

246. Tan A, Morton KR, Lee JW, Hartman R, Lee G. Adverse childhood experiences and depressive symptoms: Protective effects of dietary flavonoids. Journal of Psychosomatic Research 2020;131 (no pagination). doi: <http://dx.doi.org/10.1016/j.jpsychores.2020.109957>.

247. Nechuta SJ, Caan BJ, Chen WY, Lu W, Chen Z, Kwan ML, Flatt SW, Zheng Y, Zheng W, Pierce JP, et al. Soy food intake after diagnosis of breast cancer and survival: an in-depth analysis of combined evidence from cohort studies of US and Chinese women. American Journal of Clinical Nutrition 2012;96(1):123-32. doi: 10.3945/ajcn.112.035972.

248. Root M, Ravine E, Harper A. Flavonol Intake and Cognitive Decline in Middle-Aged Adults. Journal of Medicinal Food 2015;18(12):1327-32. doi: <http://dx.doi.org/10.1089/jmf.2015.0010>.

249. Bobe G, Weinstein SJ, Albanes D, Hirvonen T, Ashby J, Taylor PR, Virtamo J, Stolzenberg-Solomoni RZ. Flavonoid intake and risk of pancreatic cancer in male smokers (Finland). Cancer Epidemiol Biomarkers Prev 2008;17(3):553-62. doi: 10.1158/1055-9965.Epi-07-2523.

250. Wright ME, Mayne ST, Stolzenberg-Solomon RZ, Li Z, Pietinen P, Taylor PR, Virtamo J, Albanes D. Development of a comprehensive dietary antioxidant index and application to lung cancer risk in a cohort of male smokers. American Journal of Epidemiology 2004;160(1):68-76.

251. Lahmann PH, Hughes MC, Ibiebele TI, Mulligan AA, Kuhnle GGC, Webb PM. Estimated intake of dietary phyto-oestrogens in Australian women and evaluation of correlates of phyto-oestrogen intake. Journal of Nutritional Science 2012;1 (no pagination)(e11). doi: <http://dx.doi.org/10.1017/jns.2012.11>.

252. Neill AS, Ibiebele TI, Lahmann PH, Hughes MC, Nagle CM, Webb PM. Dietary phyto-oestrogens and the risk of ovarian and endometrial cancers: Findings from two Australian case-control studies. British Journal of Nutrition 2014;111(8):1430-40. doi: <http://dx.doi.org/10.1017/S0007114513003899>.

253. Bondonno NP, Lewis JR, Blekkenhorst LC, Bondonno CP, Shin JH, Croft KD, Woodman RJ, Wong G, Lim WH, Gopinath B, et al. Association of flavonoids and flavonoid-rich foods with all-cause mortality: The Blue Mountains Eye Study. Clin Nutr 2020;39(1):141-50. doi: <http://dx.doi.org/10.1016/j.clnu.2019.01.004>.

254. Gopinath B, Liew G, Kifley A, Flood VM, Joachim N, Lewis JR, Hodgson JM, Mitchell P. Dietary flavonoids and the prevalence and 15-y incidence of age-related macular degeneration. American Journal of Clinical Nutrition 2018;108(2):381-7. doi: 10.1093/ajcn/nqy114.

255. Zhang FF, Haslam DE, Terry MB, Knight JA, Andrulis IL, Daly MB, Buys SS, John EM. Dietary isoflavone intake and all-cause mortality in breast cancer survivors: The Breast Cancer Family Registry. Cancer 2017;123(11):2070-9. doi: <https://dx.doi.org/10.1002/cncr.30615>.

256. Ivey KL, Lewis JR, Prince RL, Hodgson JM. Tea and non-tea flavonol intakes in relation to atherosclerotic vascular disease mortality in older women. British Journal of Nutrition 2013;110(9):1648-55. doi: <https://dx.doi.org/10.1017/S0007114513000780>.

257. Ivey KL, Hodgson JM, Croft KD, Lewis JR, Prince RL. Flavonoid intake and all-cause mortality. American Journal of Clinical Nutrition 2015;101(5):1012-20. doi: <https://dx.doi.org/10.3945/ajcn.113.073106>.

258. Myers G, Prince RL, Kerr DA, Devine A, Woodman RJ, Lewis JR, Hodgson JM. Tea and flavonoid intake predict osteoporotic fracture risk in elderly Australian women: a prospective study. American Journal of Clinical Nutrition 2015;102(4):958-65. doi: 10.3945/ajcn.115.109892.

259. Chang ET, Lee VS, Canchola AJ, Clarke CA, Purdie DM, Reynolds P, Anton-Culver H, Bernstein L, Deapen D, Peel D, et al. Diet and risk of ovarian cancer in the California teachers study cohort. American Journal of Epidemiology 2007;165(7):802-13. doi: 10.1093/aje/kwk065.

260. Chang ET, Canchola AJ, Clarke CA, Lu YN, West DW, Bernstein L, Wang SS, Horn-Ross PL. Dietary phytocompounds and risk of lymphoid malignancies in the California Teachers Study cohort. Cancer Causes Control 2011;22(2):237-49. doi: 10.1007/s10552-010-9692-5.

261. Horn-Ross PL, Hoggatt KJ, West DW, Krone MR, Stewart SL, Anton-Culver H, Bernstein L, Deapen D, Peel D, Pinder R, et al. Recent diet and breast cancer risk: The California Teachers Study (USA). Cancer Causes and Control 2002;13(5):407-15. doi: <http://dx.doi.org/10.1023/A:1015786030864>.

262. Wang Y, Gapstur SM, Gaudet MM, Peterson JJ, Dwyer JT, McCullough ML. Evidence for an Association of Dietary Flavonoid Intake with Breast Cancer Risk by Estrogen Receptor Status Is Limited. Journal of Nutrition 2014;144(10):1603-11. doi: 10.3945/jn.114.196964.

263. Hedelin M, Klint A, Chang ET, Bellocco R, Johansson JE, Andersson SO, Heinonen SM, Adlercreutz H, Adami HO, Gronberg H, et al. Dietary phytoestrogen, serum enterolactone and risk of prostate cancer: the Cancer Prostate Sweden Study (Sweden). Cancer Causes Control 2006;17(2):169-80. doi: 10.1007/s10552-005-0342-2.

264. Ilow R, Regulska-Ilow B, Walkiewicz G, Biernat J, Kowalisko A. Evaluation of bioflavonoid intake in the diets of 50-year-old inhabitants of Wroclaw. Adv Clin Exp Med 2008;17(3):327-36.

265. Akhter M, Iwasaki M, Yamaji T, Sasazuki S, Tsugane S. Dietary isoflavone and the risk of colorectal adenoma: a case-control study in Japan. British Journal of Cancer 2009;100(11):1812-6. doi: 10.1038/sj.bjc.6605088.

266. Richardson SI, Steffen LM, Swett K, Smith C, Burke L, Zhou X, Shikany JM, Rodriguez CJ. Dietary Total Isoflavone Intake Is Associated With Lower Systolic Blood Pressure: The Coronary Artery Risk Development in Young Adults (CARDIA) Study. J Clin Hypertens (Greenwich) 2016;18(8):778-83. doi: <https://dx.doi.org/10.1111/jch.12760>.

267. McCullough ML, Peterson JJ, Patel R, Jacques PF, Shah R, Dwyer JT. Flavonoid intake and cardiovascular disease mortality in a prospective cohort of US adults. American Journal of Clinical Nutrition 2012;95(2):454-64. doi: <https://dx.doi.org/10.3945/ajcn.111.016634>.

268. Wang Y, Stevens VL, Shah R, Peterson JJ, Dwyer JT, Gapstur SM, McCullough ML. Dietary Flavonoid and Proanthocyanidin Intakes and Prostate Cancer Risk in a Prospective Cohort of US Men. American Journal of Epidemiology 2014;179(8):974-86. doi: 10.1093/aje/kwu006.

269. Bondonno NP, Dalgaard F, Kyro C, Murray K, Bondonno CP, Lewis JR, Croft KD, Gislason G, Scalbert A, Cassidy A, et al. Flavonoid intake is associated with lower mortality in the Danish Diet Cancer and Health Cohort. Nat Commun 2019;10(1):3651. doi: <https://dx.doi.org/10.1038/s41467-019-11622-x>.

270. Bondonno NP, Murray K, Bondonno CP, Lewis JR, Croft KD, Kyro C, Gislason G, Tjonneland A, Scalbert A, Cassidy A, et al. Flavonoid intake and its association with atrial fibrillation. Clinical Nutrition 2020. doi: <http://dx.doi.org/10.1016/j.clnu.2020.04.025>.

271. Dalgaard F, Bondonno NP, Murray K, Bondonno CP, Lewis JR, Croft KD, Kyro C, Gislason G, Scalbert A, Cassidy A, et al. Associations between habitual flavonoid intake and hospital admissions for atherosclerotic cardiovascular disease: a prospective cohort study. Lancet Planet Health 2019;3(11):E450-E9. doi: 10.1016/s2542-5196(19)30212-8.

272. Swann R, Perkins KA, Velentzis LS, Ciria C, Dutton SJ, Mulligan AA, Woodside JV, Cantwell MM, Leathem AJ, Robertson CE, et al. The DietCompLyf study: A prospective cohort study of breast cancer survival and phytoestrogen consumption. Maturitas 2013;75(3):232-40. doi: 10.1016/j.maturitas.2013.03.018.

273. Nooyens ACJ, Milder IEJ, van Gelder BM, Bueno-de-Mesquita HB, van Boxtel MPJ, Verschuren WMM. Diet and cognitive decline at middle age: the role of antioxidants. British Journal of Nutrition 2015;113(9):1410-7. doi: 10.1017/s0007114515000720.

274. Siedlinski M, Boer JMA, Smit HA, Postma DS, Boezen HM. Dietary factors and lung function in the general population: Wine and resveratrol intake. European Respiratory Journal 2012;39(2):385-91. doi: <http://dx.doi.org/10.1183/09031936.00184110>.

275. Lajous M, Rossignol E, Fagherazzi G, Perquier F, Scalbert A, Clavel-Chapelon F, Boutron-Ruault MC. Flavonoid intake and incident hypertension in women. American Journal of Clinical Nutrition 2016;103(4):1091-8. doi: <https://dx.doi.org/10.3945/ajcn.115.109249>.

276. Touillaud MS, Thiebaut AC, Fournier A, Niravong M, Boutron-Ruault MC, Clavel-Chapelon F. Dietary lignan intake and postmenopausal breast cancer risk by estrogen and progesterone receptor status. Journal of the National Cancer Institute 2007;99(6):475-86.

277. Minguez-Alarcon L, Afeiche MC, Chiu YH, Vanegas JC, Williams PL, Tanrikut C, Toth TL, Hauser R, Chavarro JE. Male soy food intake was not associated with in vitro fertilization outcomes among couples attending a fertility center. Andrology 2015;3(4):702-8. doi: <https://dx.doi.org/10.1111/andr.12046>.

278. Vanegas JC, Afeiche MC, Gaskins AJ, Minguez-Alarcon L, Williams PL, Wright DL, Toth TL, Hauser R, Chavarro JE. Soy food intake and treatment outcomes of women undergoing assisted reproductive technology. Fertil Steril 2015;103(3):749-U467. doi: 10.1016/j.fertnstert.2014.12.104.

279. Bandera EV, Williams MG, Sima C, Bayuga S, Pulick K, Wilcox H, Soslow R, Zauber AG, Olson SH. Phytoestrogen consumption and endometrial cancer risk: a population-based case-control study in New Jersey. Cancer Causes Control 2009;20(7):1117-27. doi: 10.1007/s10552-009-9336-9.

280. Dilis V, Trichopoulou A. Antioxidant intakes and food sources in Greek adults. Journal of Nutrition 2010;140(7):1274-9. doi: <https://dx.doi.org/10.3945/jn.110.121848>.

281. Lu YX, Zamora-Ros R, Chan S, Cross AJ, Ward H, Jakszyn P, Luben R, Opstelten JL, Oldenburg B, Hallmans G, et al. Dietary Polyphenols in the Aetiology of Crohn's Disease and Ulcerative Colitis-A Multicenter European Prospective Cohort Study (EPIC). Inflamm Bowel Dis 2017;23(12):2072-82. doi: 10.1097/mib.0000000000001108.

282. Boker LK, Van der Schouw YT, De Kleijn MJ, Jacques PF, Grobbee DE, Peeters PH. Intake of dietary phytoestrogens by Dutch women. Journal of Nutrition 2002;132(6):1319-28.

283. Keinan-Boker L, Van Der Schouw YT, Grobbee DE, Peeters PHM. Dietary phytoestrogens and breast cancer risk. American Journal of Clinical Nutrition 2004;79(2):282-8.

284. Kreijkamp-Kaspers S, Kok L, Grobbee DE, de Haan EHF, Aleman A, van der Schouw YT. Dietary phytoestrogen intake and cognitive function in older women. J Gerontol Ser A-Biol Sci Med Sci 2007;62(5):556-62. doi: 10.1093/gerona/62.5.556.

285. Van Der Schouw YT, Kreijkamp-Kaspers S, Peeters PHM, Keinan-Boker L, Rimm EB, Grobbee DE. Prospective study on usual dietary phytoestrogen intake and cardiovascular disease risk in Western women. Circulation 2005;111(4):465-71. doi: <http://dx.doi.org/10.1161/01.CIR.0000153814.87631.B0>.

286. Vrieling A, Voskuil DW, Mesquita HBBD, Kaaks R, Noord PAHV, Keinan-Boker L, Gils CHV, Peeters PHM. Dietary determinants of circulating insulin-like growth factor (IGF)-I and IGF binding proteins 1, -2 and -3 in women in the Netherlands. Cancer Causes and Control 2004;15(8):787-96. doi: <http://dx.doi.org/10.1023/B:CACO.0000043429.51915.c6>.

287. Travis RC, Allen NE, Appleby PN, Spencer EA, Roddam AW, Key TJ. A prospective study of vegetarianism and isoflavone intake in relation to breast cancer risk in British women. International Journal of Cancer 2008;122(3):705-10. doi: 10.1002/ijc.23141.

288. Verkasalo PK, Appleby PN, Davey GK, Key TJ. Soy milk intake and plasma sex hormones: A cross-sectional study in pre- and postmenopausal women (EPIC-Oxford). Nutr Cancer 2001;40(2):79-86. doi: 10.1207/s15327914nc402_1.

289. Zamora-Ros R, Ferrari P, Gonzalez CA, Tjonneland A, Olsen A, Bredsdorff L, Overvad K, Touillaud M, Perquier F, Fagherazzi G, et al. Dietary flavonoid and lignan intake and breast cancer risk according to menopause and hormone receptor status in the European Prospective Investigation into Cancer and Nutrition (EPIC) Study. Breast Cancer Res Treat 2013;139(1):163-76. doi: 10.1007/s10549-013-2483-4.

290. Jacques PF, Cassidy A, Rogers G, Peterson JJ, Dwyer JT. Dietary flavonoid intakes and CVD incidence in the Framingham Offspring Cohort. British Journal of Nutrition 2015;114(9):1496-503. doi: 10.1017/s0007114515003141.

291. Shishtar E, Rogers GT, Blumberg JB, Au R, DeCarli C, Jacques PF. Flavonoid Intake and MRI Markers of Brain Health in the Framingham Offspring Cohort. The Journal of nutrition 2020;25. doi: <http://dx.doi.org/10.1093/jn/nxaa068>.

292. Shishtar E, Rogers GT, Blumberg JB, Au RD, Jacques PF. Long-term dietary flavonoid intake and change in cognitive function in the Framingham Offspring cohort. Public Health Nutrition 2020;23(9):1576-88. doi: 10.1017/s136898001900394x.

293. Cassidy A, Rogers G, Peterson JJ, Dwyer JT, Lin HH, Jacques PF. Higher dietary anthocyanin and flavonol intakes are associated with anti-inflammatory effects in a population of US adults. American Journal of Clinical Nutrition 2015;102(1):172-81. doi: 10.3945/ajcn.115.108555.

294. de Kleijn MJJ, van der Schouw YT, Wilson PWF, Grobbee DE, Jacques PF. Dietary intake of phytoestrogens is associated with a favorable metabolic cardiovascular risk profile in postmenopausal US women: The Framingham Study. Journal of Nutrition 2002;132(2):276-82.

295. Jacques PF, Cassidy A, Rogers G, Peterson JJ, Meigs JB, Dwyer JT. Higher Dietary Flavonol Intake Is Associated with Lower Incidence of Type 2 Diabetes. Journal of Nutrition 2013;143(9):1474-80. doi: 10.3945/jn.113.177212.

296. Budhathoki S, Joshi AM, Ohnaka K, Yin G, Toyomura K, Kono S, Mibu R, Tanaka M, Kakeji Y, Maehara Y, et al. Soy food and isoflavone intake and colorectal cancer risk: The Fukuoka Colorectal Cancer Study. Scand J Gastroenterol 2011;46(2):165-72. doi: 10.3109/00365521.2010.522720.

297. Wang ZJ, Ohnaka K, Morita M, Toyomura K, Kono S, Ueki T, Tanaka M, Kakeji Y, Maehara Y, Okamura T, et al. Dietary polyphenols and colorectal cancer risk: The Fukuoka colorectal cancer study. World J Gastroenterol 2013;19(17):2683-90. doi: 10.3748/wjg.v19.i17.2683.

298. Garcia-Larsen V, Thawer N, Charles D, Cassidy A, van Zele T, Thilsing T, Ahlstrom M, Haahtela T, Keil T, Matricardi PM, et al. Dietary Intake of Flavonoids and Ventilatory Function in European Adults: A GA2LEN Study. Nutrients 2018;10(1):15. doi: <https://dx.doi.org/10.3390/nu10010095>.

299. Mattioli V, Zanolin ME, Cazzoletti L, Bono R, Cerveri I, Ferrari M, Pirina P, Garcia-Larsen V. Dietary flavonoids and respiratory diseases: a population-based multi-case-control study in Italian adults. Public health nutrition 2020:1-9. doi: <http://dx.doi.org/10.1017/S1368980019003562>.

300. Qu RG, Jia YB, Liu JY, Jin SS, Han TS, Na LX. Dietary Flavonoids, Copper Intake, and Risk of Metabolic Syndrome in Chinese Adults. Nutrients 2018;10(8):11. doi: 10.3390/nu10080991.

301. Alhassani AA, Hu FB, Rimm EB, Li YP, Rosner BA, Willett WC, Joshipura KJ. Dietary flavonoid intake and risk of periodontitis. J Periodont 2020. doi: 10.1002/jper.19-0463.

302. Cassidy A, Bertoia M, Chiuve S, Flint A, Forman J, Rimm EB. Habitual intake of anthocyanins and flavanones and risk of cardiovascular disease in men. American Journal of Clinical Nutrition 2016;104(3):587-94. doi: 10.3945/ajcn.116.133132.

303. Cassidy A, Franz M, Rimm EB. Dietary flavonoid intake and incidence of erectile dysfunction. American Journal of Clinical Nutrition 2016;103(2):534-41. doi: <https://dx.doi.org/10.3945/ajcn.115.122010>.

304. Rimm EB, Katan MB, Ascherio A, Stampfer MJ, Willett WC. Relation between intake of flavonoids and risk for coronary heart disease in male health professionals. Ann Intern Med 1996;125(5):384-9. doi: 10.7326/0003-4819-125-5-199609010-00005.

305. van der Schouw YT, Sampson L, Willett WC, Rimm EB. The usual intake of lignans but not that of isoflavones may be related to cardiovascular risk factors in US men. Journal of Nutrition 2005;135(2):260-6.

306. Grosso G, Stepaniak U, Micek A, Stefler D, Bobak M, Pajak A. Dietary polyphenols are inversely associated with metabolic syndrome in Polish adults of the HAPIEE study. European Journal of Nutrition 2017;56(4):1409-20. doi: 10.1007/s00394-016-1187-z.

307. Grosso G, Stepaniak U, Micek A, Kozela M, Stefler D, Bobak M, Pajak A. Dietary polyphenol intake and risk of hypertension in the Polish arm of the HAPIEE study. European Journal of Nutrition 2018;57(4):1535-44. doi: <https://dx.doi.org/10.1007/s00394-017-1438-7>.

308. Lei YY, Ho SC, Cheng A, Kwok C, Cheung KL, He YQ, Lee R, Yeo W. The association between soy isoflavone intake and menopausal symptoms after breast cancer diagnosis: a prospective longitudinal cohort study on Chinese breast cancer patients. Breast Cancer Res Treat 2020;181(1):167-80. doi: <http://dx.doi.org/10.1007/s10549-020-05616-3>.

309. Rabassa M, Cherubini A, Zamora-Ros R, Urpi-Sarda M, Bandinelli S, Ferrucci L, Andres-Lacueva C. Low Levels of a Urinary Biomarker of Dietary Polyphenol Are Associated with Substantial Cognitive Decline over a 3-Year Period in Older Adults: The Invecchiare in Chianti Study. J Am Geriatr Soc 2015;63(5):938-46. doi: 10.1111/jgs.13379.

310. Rabassa M, Zamora-Ros R, Urpi-Sarda M, Bandinelli S, Ferrucci L, Andres-Lacueva C, Cherubini A. Association of habitual dietary resveratrol exposure with the development of frailty in older age: The Invecchiare in Chianti study1,2. American Journal of Clinical Nutrition 2015;102(6):1534-42. doi: <http://dx.doi.org/10.3945/ajcn.115.118976>.

311. Rabassa M, Zamora-Ros R, Andres-Lacueva C, Urpi-Sarda M, Bandinelli S, Ferrucci L, Cherubini A. Association between both total baseline urinary and dietary polyphenols and substantial physical performance decline risk in older adults: A 9-year follow-up of the InCHIANTI study. J Nutr Health Aging 2016;20(5):478-84. doi: 10.1007/s12603-015-0600-2.

312. Urpi-Sarda M, Andres-Lacueva C, Rabassa M, Ruggiero C, Zamora-Ros R, Bandinelli S, Ferrucci L, Cherubini A. The Relationship Between Urinary Total Polyphenols and the Frailty Phenotype in a Community-Dwelling Older Population: The InCHIANTI Study. J Gerontol Ser A-Biol Sci Med Sci 2015;70(9):1141-7. doi: 10.1093/gerona/glv026.

313. Zamora-Ros R, Rabassa M, Cherubini A, Urpi-Sarda M, Llorach R, Bandinelli S, Ferrucci L, Andres-Lacueva C. Comparison of 24-h volume and creatinine-corrected total urinary polyphenol as a biomarker of total dietary polyphenols in the Invecchiare InCHIANTI study. Anal Chim Acta 2011;704(1-2):110-5. doi: 10.1016/j.aca.2011.07.035.

314. Zamora-Ros R, Rabassa M, Cherubini A, Urpi-Sarda M, Bandinelli S, Ferrucci L, Andres-Lacueva C. High Concentrations of a Urinary Biomarker of Polyphenol Intake Are Associated with Decreased Mortality in Older Adults. Journal of Nutrition 2013;143(9):1445-50. doi: 10.3945/jn.113.177121.

315. Nakamoto M, Uemura H, Sakai T, Katsuura-Kamano S, Yamaguchi M, Hiyoshi M, Arisawa K. Inverse association between soya food consumption and insulin resistance in Japanese adults. Public Health Nutrition 2015;18(11):2031-40. doi: 10.1017/s136898001400247x.

316. Uemura H, Katsuura-Kamano S, Nakamoto M, Yamaguchi M, Fujioka M, Iwasaki Y, Arisawa K. Inverse association between soy food consumption, especially fermented soy products intake and soy isoflavone, and arterial stiffness in Japanese men. Sci Rep 2018;8:9. doi: 10.1038/s41598-018-28038-0.

317. Wilunda C, Sawada N, Goto A, Yamaji T, Iwasaki M, Tsugane S, Noda M. Soy food and isoflavones are not associated with changes in serum lipids and glycohemoglobin concentrations among Japanese adults: a cohort study. European Journal of Nutrition 2019. doi: <http://dx.doi.org/10.1007/s00394-019-02057-7>.

318. Michikawa T, Yamazaki S, Ono M, Kuroda T, Nakayama SF, Suda E, Isobe T, Iwai-Shimada M, Kobayashi Y, Yonemoto J, et al. Isoflavone Intake in Early Pregnancy and Hypospadias in the Japan Environment and Children's Study. Urology 2018. doi: <http://dx.doi.org/10.1016/j.urology.2018.11.008>.

319. Michikawa T, Yamazaki S, Ono M, Kuroda T, Nakayama SF, Suda E, Isobe T, Iwai-Shimada M, Kobayashi Y, Yonemoto J, et al. Isoflavone Intake in Early Pregnancy and Hypospadias in the Japan Environment and Children's Study. Urology 2019;124:229-36. doi: <http://dx.doi.org/10.1016/j.urology.2018.11.008>.

320. Cao Y, Taylor AW, Zhen S, Adams R, Appleton S, Shi Z. Soy Isoflavone Intake and Sleep Parameters over 5 Years among Chinese Adults: Longitudinal Analysis from the Jiangsu Nutrition Study. J Acad Nutr Diet 2017;117(4):536-44.e2. doi: <https://dx.doi.org/10.1016/j.jand.2016.10.016>.

321. Kim HS, Kwon M, Lee HY, Shivappa N, Hebert JR, Sohn C, Na W, Kim MK. Higher Pro-Inflammatory Dietary Score is Associated with Higher Hyperuricemia Risk: Results from the Case-Controlled Korean Genome and Epidemiology Study_Cardiovascular Disease Association Study. Nutrients 2019;11(8). doi: 10.3390/nu11081803.

322. Guha N, Kwan ML, Quesenberry Jr CP, Weltzien EK, Castillo AL, Caan BJ. Soy isoflavones and risk of cancer recurrence in a cohort of breast cancer survivors: The Life after Cancer Epidemiology study. Breast Cancer Res Treat 2009;118(2):395-405. doi: <http://dx.doi.org/10.1007/s10549-009-0321-5>.

323. Fink BN, Steck SE, Wolff MS, Kabat GC, Gammon MD. Construction of a flavonoid database for assessing intake in a population-based sample of women on Long Island, New York. Nutr Cancer 2006;56(1):57-66. doi: 10.1207/s15327914nc5601_8.

324. Fink BN, Steck SE, Wolff MS, Britton JA, Kabat GC, Gaudet MM, Abrahamson PE, Bell P, Schroeder JC, Teitelbaum SL, et al. Dietary flavonoid intake and breast cancer survival among women on long island. Cancer Epidemiol Biomarkers Prev 2007;16(11):2285-92. doi: 10.1158/1055-9965.Epi-07-0245.

325. Fink BN, Steck SE, Wolff MS, Britton JA, Kabat GC, Schroeder JC, Teitelbaum SL, Neugut AI, Gammon MD. Dietary flavonoid intake and breast cancer risk among women on long island. American Journal of Epidemiology 2007;165(5):514-23. doi: 10.1093/aje/kwk033.

326. Hanna KL, O'Neill S, Lyons-Wall PM. Intake of isoflavone and lignan phytoestrogens and associated demographic and lifestyle factors in older Australian women. Asia Pacific Journal of Clinical Nutrition 2010;19(4):540-9.

327. Butchart C, Kyle J, McNeill G, Corley J, Gow AJ, Starr JM, Deary IJ. Flavonoid intake in relation to cognitive function in later life in the Lothian Birth Cohort 1936. British Journal of Nutrition 2011;106(1):141-8. doi: 10.1017/s0007114510005738.

328. Buck K, Zaineddin AK, Vrieling A, Heinz J, Linseisen J, Flesch-Janys D, Chang-Claude J. Estimated enterolignans, lignan-rich foods, and fibre in relation to survival after postmenopausal breast cancer. British Journal of Cancer 2011;105(8):1151-7. doi: 10.1038/bjc.2011.374.

329. Zaineddin AK, Buck K, Vrieling A, Heinz J, Flesch-Janys D, Linseisen J, Chang-Claude J. The Association Between Dietary Lignans, Phytoestrogen-Rich Foods, and Fiber Intake and Postmenopausal Breast Cancer Risk: A German Case-Control Study. Nutr Cancer 2012;64(5):652-65. doi: 10.1080/01635581.2012.683227.

330. Vitelli-Storelli F, Zamora-Ros R, Molina AJ, Fernandez-Villa T, Castello A, Barrio JP, Amiano P, Ardanaz E, Obon-Santacana M, Gomez-Acebo I, et al. Association between polyphenol intake and breast cancer risk by menopausal and hormone receptor status. Nutrients 2020;12(4). doi: <http://dx.doi.org/10.3390/nu12040994>.

331. Vitelli Storelli F, Molina AJ, Zamora-Ros R, Fernandez-Villa T, Roussou V, Romaguera D, Aragones N, Obon-Santacana M, Guevara M, Gomez-Acebo I, et al. Flavonoids and the Risk of Gastric Cancer: An Exploratory Case-Control Study in the MCC-Spain Study. Nutrients 2019;11(5):27. doi: <https://dx.doi.org/10.3390/nu11050967>.

332. Maskarinec G, Jacobs S, Shvetsov Y, Boushey CJ, Setiawan VW, Kolonel LN, Haiman CA, Le Marchand L. Intake of cocoa products and risk of type-2 diabetes: the multiethnic cohort. European Journal of Clinical Nutrition 2018:1-8. doi: <http://dx.doi.org/10.1038/s41430-018-0188-9>.

333. Maskarinec G, Jacobs S, Shvetsov Y, Boushey CJ, Setiawan VW, Koloner LN, Haiman CA, Le Marchand L. Intake of cocoa products and risk of type-2 diabetes: the multiethnic cohort. European Journal of Clinical Nutrition 2019;73(5):671-8. doi: 10.1038/s41430-018-0188-9.

334. Morimoto Y, Maskarinec G, Park SY, Ettienne R, Matsuno RK, Long C, Steffen AD, Henderson BE, Kolonel LN, Le Marchand L, et al. Dietary isoflavone intake is not statistically significantly associated with breast cancer risk in the Multiethnic Cohort. British Journal of Nutrition 2014;112(6):976-83. doi: <https://dx.doi.org/10.1017/S0007114514001780>.

335. Nothlings U, Murphy SP, Wilkens LR, Henderson BE, Kolonel LN. Flavonols and pancreatic cancer risk - The multiethnic cohort study. American Journal of Epidemiology 2007;166(8):924-31. doi: 10.1093/aje/kwm172.

336. Ollberding NJ, Lim U, Wilkens LR, Setiawan VW, Shvetsov YB, Henderson BE, Kolonel LN, Goodman MT. Legume, Soy, Tofu, and Isoflavone Intake and Endometrial Cancer Risk in Postmenopausal Women in the Multiethnic Cohort Study. Journal of the National Cancer Institute 2012;104(1):67-76. doi: 10.1093/jnci/djr475.

337. Park SY, Murphy SP, Wilkens LR, Henderson BE, Kolonel LN. Legume and isoflavone intake and prostate cancer risk: The multiethnic cohort study. International Journal of Cancer 2008;123(4):927-32. doi: 10.1002/ijc.23594.

338. Rohrmann S, Shvetsov YB, Morimoto Y, Wilkens LR, Monroe KR, Le Marchand L, Franke AA, Kolonel LN, Maskarinec G. Self-reported dietary flavonoid intake and serum markers of inflammation: the multiethnic cohort. Cancer Causes Control 2018;29(6):601-7. doi: 10.1007/s10552-018-1034-z.

339. Godos J, Marventano S, Mistretta A, Galvano F, Grosso G. Dietary sources of polyphenols in the Mediterranean healthy Eating, Aging and Lifestyle (MEAL) study cohort. Int J Food Sci Nutr 2017;68(6):750-6. doi: 10.1080/09637486.2017.1285870.

340. Godos J, Sinatra D, Blanco I, Mule S, La Verde M, Marranzano M. Association between dietary phenolic acids and hypertension in a mediterranean cohort. Nutrients 2017;9 (10) (no pagination)(1069). doi: <http://dx.doi.org/10.3390/nu9101069>.

341. Godos J, Bergante S, Satriano A, Pluchinotta FR, Marranzano M. Dietary Phytoestrogen Intake is Inversely Associated with Hypertension in a Cohort of Adults Living in the Mediterranean Area. Molecules 2018;23(2):11. doi: 10.3390/molecules23020368.

342. Godos J, Castellano S, Ray S, Grosso G, Galvano F. Dietary Polyphenol Intake and Depression: Results from the Mediterranean Healthy Eating, Lifestyle and Aging (MEAL) Study. Molecules 2018;23(5):15. doi: 10.3390/molecules23050999.

343. Godos J, Ferri R, Castellano S, Angelino D, Mena P, Del Rio D, Caraci F, Galvano F, Grosso G. Specific dietary (Poly)phenols are associated with sleep quality in a cohort of Italian adults. Nutrients 2020;12(5). doi: <http://dx.doi.org/10.3390/nu12051226>.

344. Marranzano M, Ray S, Godos J, Galvano F. Association between dietary flavonoids intake and obesity in a cohort of adults living in the Mediterranean area. Int J Food Sci Nutr 2018;69(8):1020-9. doi: 10.1080/09637486.2018.1452900.

345. Agarwal P, Holland TM, Wang Y, Bennett DA, Morris MC. Association of strawberries and anthocyanidin intake with alzheimer's dementia risk. Nutrients 2019;11(12). doi: <http://dx.doi.org/10.3390/nu11123060>.

346. Holland TM, Agarwal P, Wang Y, Leurgans SE, Bennett DA, Booth SL, Morris MC. Dietary flavonols and risk of Alzheimer dementia. Neurology 2020;29. doi: <http://dx.doi.org/10.1212/WNL.0000000000008981>.

347. Morris MC, Wang Y, Barnes LL, Bennett DA, Dawson-Hughes B, Booth SL. Nutrients and bioactives in green leafy vegetables and cognitive decline: Prospective study. Neurology 2018;90(3):e214-e22. doi: <http://dx.doi.org/10.1212/WNL.0000000000004815>.

348. Paller CJ, Kanaan YM, Beyene DA, Naab TJ, Copeland RL, Tsai HL, Kanarek NF, Hudson TS. Risk of Prostate Cancer in African-American Men: Evidence of Mixed Effects of Dietary Quercetin by Serum Vitamin D Status. Prostate 2015;75(13):1376-83. doi: 10.1002/pros.23018.

349. Ivey KL, Chan AT, Izard J, Cassidy A, Rogers GB, Rimma EB. Role of dietary flavonoid compounds in driving patterns of microbial community assembly. mBio 2019;10(5). doi: <http://dx.doi.org/10.1128/mBio.01205-19>.

350. Pounis G, Bonaccio M, Di Castelnuovo A, Costanzo S, de Curtis A, Persichillo M, Sieri S, Donati MB, Cerletti C, de Gaetano G, et al. Polyphenol intake is associated with low-grade inflammation, using a novel data analysis from the Moli-sani study. Thromb Haemost 2016;115(2):344-52. doi: <https://dx.doi.org/10.1160/TH15-06-0487>.

351. Pounis G, Di Castelnuovo A, Bonaccio M, Costanzo S, Persichillo M, Krogh V, Donati MB, de Gaetano G, Iacoviello L. Flavonoid and lignan intake in a Mediterranean population: proposal for a holistic approach in polyphenol dietary analysis, the Moli-sani Study. European Journal of Clinical Nutrition 2016;70(3):338-45. doi: <https://dx.doi.org/10.1038/ejcn.2015.178>.

352. Pounis G, Arcari A, Costanzo S, Di Castelnuovo A, Bonaccio M, Persichillo M, Donati MB, de Gaetano G, Iacoviello L. Favorable association of polyphenol-rich diets with lung function: Cross-sectional findings from the Moli-sani study. Respir Med 2018;136:48-57. doi: 10.1016/j.rmed.2017.12.007.

353. Pounis G, Costanzo S, Bonaccio M, Di Castelnuovo A, De Curtis A, Ruggiero E, Persichillo M, Cerletti C, Donati MB, de Gaetano G, et al. Reduced mortality risk by a polyphenol-rich diet: An analysis from the Moli-sani study. Nutrition 2018;48:87-95. doi: <http://dx.doi.org/10.1016/j.nut.2017.11.012>.

354. Nikkhah-Bodaghi M, Ghanavati M, Hekmatdoost A. Polyphenol intakes and risk of impaired lipid profile, elevated hepatic enzymes and nonalcoholic fatty liver disease. Nutr Food Sci 2019;49(5):903-10. doi: 10.1108/nfs-12-2018-0338.

355. Carmichael SL, Gonzalez-Feliciano AG, Ma C, Shaw GM, Cogswell ME. Estimated dietary phytoestrogen intake and major food sources among women during the year before pregnancy. Nutr J 2011;10:9. doi: 10.1186/1475-2891-10-105.

356. Wadhwa EL, Ma C, Shaw GM, Carmichael SL. Gastroschisis and maternal intake of phytoestrogens. American Journal of Medical Genetics, Part A 2016;170(8):2078-82. doi: <http://dx.doi.org/10.1002/ajmg.a.37659>.

357. Geybels MS, Verhage BAJ, Arts ICW, van Schooten FJ, Goldbohm RA, van den Brandt PA. Dietary Flavonoid Intake, Black Tea Consumption, and Risk of Overall and Advanced Stage Prostate Cancer. American Journal of Epidemiology 2013;177(12):1388-98. doi: 10.1093/aje/kws419.

358. Hughes LAE, Arts ICW, Ambergen T, Brants HAM, Dagnelie PC, Goldbohm RA, van den Brandt PA, Weijenberg MP. Higher dietary flavone, flavonol, and catechin intakes are associated with less of an increase in BMI over time in women: a longitudinal analysis from the Netherlands Cohort Study. American Journal of Clinical Nutrition 2008;88(5):1341-52. doi: 10.3945/ajcn.2008.26058.

359. Simons CCJM, Hughes LAE, Arts ICW, Goldbohm RA, Van Den Brandt PA, Weijenberg MP. Dietary flavonol, flavone and catechin intake and risk of colorectal cancer in the Netherlands Cohort Study. International Journal of Cancer 2009;125(12):2945-52. doi: <http://dx.doi.org/10.1002/ijc.24645>.

360. Vernarelli JA, Lambert JD. Flavonoid intake is inversely associated with obesity and C-reactive protein, a marker for inflammation, in US adults. Nutr Diabetes 2017;7(5):e276. doi: <https://dx.doi.org/10.1038/nutd.2017.22>.

361. Sun L, Subar AF, Bosire C, Dawsey SM, Kahle LL, Zimmerman TP, Abnet CC, Heller R, Graubard BI, Cook MB, et al. Dietary Flavonoid Intake Reduces the Risk of Head and Neck but Not Esophageal or Gastric Cancer in US Men and Women. Journal of Nutrition 2017;147(9):1729-38. doi: 10.3945/jn.117.251579.

362. Xiao Q, Park Y, Hollenbeck AR, Kitahara CM. Dietary flavonoid intake and thyroid cancer risk in the NIH-AARP diet and health study. Cancer Epidemiol Biomarkers Prev 2014;23(6):1102-8. doi: <https://dx.doi.org/10.1158/1055-9965.EPI-13-1150>.

363. Mehta AJ, Cassidy A, Litonjua AA, Sparrow D, Vokonas P, Schwartz J. Dietary anthocyanin intake and age-related decline in lung function: longitudinal findings from the VA Normative Aging Study. American Journal of Clinical Nutrition 2016;103(2):542-50. doi: 10.3945/ajcn.115.121467.

364. Adebamowo CA, Cho E, Sampson L, Katan MB, Spiegelman D, Willett WC, Holmes MD. Dietary flavonols and flavonol-rich foods intake and the risk of breast cancer. International Journal of Cancer 2005;114(4):628-33. doi: 10.1002/ijc.20741.

365. Cassidy A, Mukamal KJ, Liu L, Franz M, Eliassen AH, Rimm EB. High Anthocyanin Intake Is Associated With a Reduced Risk of Myocardial Infarction in Young and Middle-Aged Women. Circulation 2013;127(2):188-96. doi: 10.1161/circulationaha.112.122408.

366. Cassidy A, Huang TY, Rice MS, Rimm EB, Tworoger SS. Intake of dietary flavonoids and risk of epithelial ovarian cancer. American Journal of Clinical Nutrition 2014;100(5):1344-51. doi: 10.3945/ajcn.114.088708.

367. Chang SC, Cassidy A, Willett WC, Rimm EB, O'Reilly EJ, Okereke OI. Dietary flavonoid intake and risk of incident depression in midlife and older women. American Journal of Clinical Nutrition 2016;104(3):704-14. doi: 10.3945/ajcn.115.124545.

368. Devore EE, Kang JH, Breteler MMB, Grodstein F. Dietary intakes of berries and flavonoids in relation to cognitive decline. Annals of Neurology 2012;72(1):135-43. doi: <http://dx.doi.org/10.1002/ana.23594>.

369. Gates MA, Tworoger SS, Hecht JL, De Vivo I, Rosner B, Hankinson SE. A prospective study of dietary flavonoid intake and incidence of epithelial ovarian cancer. International Journal of Cancer 2007;121(10):2225-32.

370. Samieri C, Sun Q, Townsend MK, Rimm EB, Grodstein F. Dietary flavonoid intake at midlife and healthy aging in women. American Journal of Clinical Nutrition 2014;100(6):1489-97. doi: 10.3945/ajcn.114.085605.

371. Wu Y, Hankinson SE, Smith-Warner SA, Wang M, Eliassen AH. Flavonoid Intake and Plasma Sex Steroid Hormones, Prolactin, and Sex Hormone-Binding Globulin in Premenopausal Women. Nutrients 2019;11(11):05. doi: <https://dx.doi.org/10.3390/nu11112669>.

372. Zhong J, Colicino E, Lin X, Mehta A, Kloog I, Zanobetti A, Byun HM, Bind MA, Cantone L, Prada D, et al. Cardiac autonomic dysfunction: particulate air pollution effects are modulated by epigenetic immunoregulation of Toll-like receptor 2 and dietary flavonoid intake.[Erratum appears in J Am Heart Assoc. 2016;5(1). pii: e002008. doi: 10.1161/JAHA.115.002008; PMID: 26819248]. J Am Heart Assoc 2015;4(1):e001423. doi: <https://dx.doi.org/10.1161/JAHA.114.001423>.

373. Cassidy A, O'Reilly EJ, Kay C, Sampson L, Franz M, Forman JP, Curhan G, Rimm EB. Habitual intake of flavonoid subclasses and incident hypertension in adults. American Journal of Clinical Nutrition 2011;93(2):338-47. doi: 10.3945/ajcn.110.006783.

374. Ding M, Pan A, Manson JE, Willett WC, Malik V, Rosner B, Giovannucci E, Hu FB, Sun Q. Consumption of soy foods and isoflavones and risk of type 2 diabetes: a pooled analysis of three US cohorts. European Journal of Clinical Nutrition 2016;70(12):1381-7. doi: 10.1038/ejcn.2016.117.

375. Gao X, Cassidy A, Schwarzschild MA, Rimm EB, Ascherio A. Habitual intake of dietary flavonoids and risk of Parkinson disease. Neurology 2012;78(15):1138-45. doi: 10.1212/WNL.0b013e31824f7fc4.

376. Kang JH, Ivey KL, Boumenna T, Rosner B, Wiggs JL, Pasquale LR. Prospective study of flavonoid intake and risk of primary open-angle glaucoma. Acta Ophthalmol 2018;96(6):E692-E700. doi: 10.1111/aos.13705.

377. Lin J, Rexrode KM, Hu F, Albert CM, Chae CU, Rimm EB, Stampfer MJ, Manson JE. Dietary intakes of flavonols and flavones and coronary heart disease in US women. American Journal of Epidemiology 2007;165(11):1305-13.

378. Ma L, Liu G, Ding M, Zong G, Hu FB, Willett WC, Rimm EB, Manson JE, Sun Q. Isoflavone Intake and the Risk of Coronary Heart Disease in US Men and Women Results From 3 Prospective Cohort Studies. Circulation 2020;141(14):1127-37. doi: 10.1161/circulationaha.119.041306.

379. Nimptsch K, Zhang X, Cassidy A, Song M, O'Reilly EJ, Lin JH, Pischon T, Rimm EB, Willett WC, Fuchs CS, et al. Habitual intake of flavonoid subclasses and risk of colorectal cancer in 2 large prospective cohorts. American Journal of Clinical Nutrition 2016;103(1):184-91. doi: <https://dx.doi.org/10.3945/ajcn.115.117507>.

380. Wedick NM, Pan A, Cassidy A, Rimm EB, Sampson L, Rosner B, Willett W, Hu FB, Sun Q, van Dam RM. Dietary flavonoid intakes and risk of type 2 diabetes in US men and women. American Journal of Clinical Nutrition 2012;95(4):925-33. doi: 10.3945/ajcn.111.028894.

381. Thanos J, Cotterchio M, Boucher BA, Kreiger N, Thompson LU. Adolescent dietary phytoestrogen intake and breast cancer risk (Canada). Cancer Causes Control 2006;17(10):1253-61. doi: 10.1007/s10552-006-0062-2.

382. Letenneur L, Proust-Lima C, Le Gouge A, Dartigues JF, Barberger-Gateau P. Flavonoid intake and cognitive decline over a 10-year period. American Journal of Epidemiology 2007;165(12):1364-71. doi: 10.1093/aje/kwm036.

383. Heald CL, Bolton-Smith C, Ritchie MR, Morton MS, Alexander FE. Phyto-oestrogen intake in Scottish men: use of serum to validate a self-administered food-frequency questionnaire in older men. European Journal of Clinical Nutrition 2006;60(1):129-35. doi: 10.1038/sj.ejcn.1602277.

384. Heald CL, Ritchie MR, Bolton-Smith C, Morton MS, Alexander FE. Phyto-oestrogens and risk of prostate cancer in Scottish men. British Journal of Nutrition 2007;98(2):388-96. doi: 10.1017/s0007114507700703.

385. Milder IEJ, Kuijsten A, Arts ICW, Feskens EJM, Kampman E, Hollman PCH, Van't Veer P. Relation between plasma enterodiol and enterolactone and dietary intake of lignans in a dutch endoscopy-based population. Journal of Nutrition 2007;137(5):1266-71.

386. Jennings A, Koch M, Jensen MK, Bang C, Kassubek J, Muller HP, Nothlings U, Franke A, Lieb W, Cassidy A. The role of the gut microbiome in the association between habitual anthocyanin intake and visceral abdominal fat in population-level analysis. American Journal of Clinical Nutrition 2020;111(2):340-50. doi: 10.1093/ajcn/nqz299.

387. Creus-Cuadros A, Tresserra-Rimbau A, Quifer-Rada P, Martinez-Gonzalez MA, Corella D, Salas-Salvado J, Fito M, Estruch R, Gomez-Gracia E, Lapetra J, et al. Associations between Both Lignan and Yogurt Consumption and Cardiovascular Risk Parameters in an Elderly Population: Observations from a Cross-Sectional Approach in the PREDIMED Study. J Acad Nutr Diet 2017;117(4):609-22.e1. doi: <https://dx.doi.org/10.1016/j.jand.2016.11.003>.

388. Tresserra-Rimbau A, Medina-Remon A, Perez-Jimenez J, Martinez-Gonzalez MA, Covas MI, Corella D, Salas-Salvado J, Gomez-Gracia E, Lapetra J, Aros F, et al. Dietary intake and major food sources of polyphenols in a Spanish population at high cardiovascular risk: the PREDIMED study. Nutr Metab Cardiovasc Dis 2013;23(10):953-9. doi: <https://dx.doi.org/10.1016/j.numecd.2012.10.008>.

389. Tresserra-Rimbau A, Castro-Barquero S, Vitelli-Storelli F, Becerra-Tomas N, Vazquez-Ruiz Z, Diaz-Lopez A, Corella D, Castaner O, Romaguera D, Vioque J, et al. Associations between Dietary Polyphenols and Type 2 Diabetes in a Cross-Sectional Analysis of the PREDIMED-Plus Trial: Role of Body Mass Index and Sex. Antioxidants 2019;8(11). doi: 10.3390/antiox8110537.

390. Wesselink AK, Hatch EE, Mikkelsen EM, Trolle E, Willis SK, McCann SE, Valsta L, Lundqvist A, Tucker KL, Rothman KJ, et al. Dietary phytoestrogen intakes of adult women are not strongly related to fecundability in 2 preconception cohort studies. The Journal of nutrition 2020;14. doi: <http://dx.doi.org/10.1093/jn/nxz335>.

391. Reger MK, Zollinger TW, Liu ZY, Jones JF, Zhang JJ. Dietary intake of isoflavones and coumestrol and the risk of prostate cancer in the Prostate, Lung, Colorectal and Ovarian Cancer Screening Trial. International Journal of Cancer 2018;142(4):719-28. doi: 10.1002/ijc.31095.

392. Goetz ME, Judd SE, Hartman TJ, McClellan W, Anderson A, Vaccarino V. Flavanone Intake Is Inversely Associated with Risk of Incident Ischemic Stroke in the REasons for Geographic and Racial Differences in Stroke (REGARDS) Study. Journal of Nutrition 2016;146(11):2233-43. doi: 10.3945/jn.116.230185.

393. Goetz ME, Judd SE, Safford MM, Hartman TJ, McClellan WM, Vaccarino V. Dietary flavonoid intake and incident coronary heart disease: the REasons for Geographic and Racial Differences in Stroke (REGARDS) study. American Journal of Clinical Nutrition 2016;104(5):1236-44. doi: 10.3945/ajcn.115.129452.

394. De Rijk MC, Breteler MMB, Den Breeijen JH, Launer LJ, Grobbee DE, Van der Meche FGA, Hofman A. Dietary antioxidants and Parkinson disease: The Rotterdam Study. Archives of Neurology 1997;54(6):762-5.

395. Devore EE, Grodstein F, van Rooij FJA, Hofman A, Stampfer MJ, Witteman JCM, Breteler MMB. Dietary Antioxidants and Long-term Risk of Dementia. Archives of Neurology 2010;67(7):819-25.

396. Engelhart MJ, Geerlings MI, Ruitenberg A, Van Swieten JC, Hofman A, Witteman JCM, Breteler MMB. Dietary intake of antioxidants and risk of Alzheimer disease. Journal of the American Medical Association 2002;287(24):3223-9.

397. Geleijnse JM, Launer LJ, van der Kuip DAM, Hofman A, Witteman JCM. Inverse association of tea and flavonoid intakes with incident myocardial infarction: the Rotterdam Study. American Journal of Clinical Nutrition 2002;75(5):880-6.

398. Pantavos A, Ruiter R, Feskens EF, de Keyser CE, Hofman A, Stricker BH, Franco OH, Kiefte-de Jong JC. Total dietary antioxidant capacity, individual antioxidant intake and breast cancer risk: The Rotterdam study. International Journal of Cancer 2015;136(9):2178-86. doi: 10.1002/ijc.29249.

399. Ramdas WD, Wolfs RCW, Kiefte-De Jong JC, Hofman A, De Jong PTVM, Vingerling JR, Jansonius NM. Nutrient intake and risk of open-Angle glaucoma: The Rotterdam Study. European Journal of Epidemiology 2012;27(5):385-93. doi: <http://dx.doi.org/10.1007/s10654-012-9672-z>.

400. Horn-Ross PL, Hoggatt KJ, Lee MM. Phytoestrogens and thyroid cancer risk: The San Francisco Bay Area thyroid cancer study. Cancer Epidemiol Biomarkers Prev 2002;11(1):43-9.

401. Tedeschi-Blok N, Lee M, Sison JD, Miike R, Wrensch M. Inverse association of antioxidant and phytoestrogen nutrient intake with adult glioma in the San Francisco Bay Area: a case-control study. BMC Cancer 2006;6:12. doi: 10.1186/1471-2407-6-148.

402. Ursin G, Sun CL, Koh WP, Khoo KS, Gao F, Wu AH, Yu MC. Associations between soy, diet, reproductive factors, and mammographic density in Singapore Chinese women. Nutr Cancer 2006;56(2):128-35. doi: 10.1207/s15327914nc5602_2.

403. Portman MA, Navarro SL, Bruce ME, Lampe JW. Soy isoflavone intake is associated with risk of Kawasaki disease. Nutrition Research 2016;36(8):827-34. doi: 10.1016/j.nutres.2016.04.002.

404. Gardeazabal I, Romanos-Nanclares A, Martinez-Gonzalez MA, Sanchez-Bayona R, Vitelli-Storelli F, Gaforio JJ, Aramendia-Beitia JM, Toledo E. Total polyphenol intake and breast cancer risk in the Seguimiento Universidad de Navarra (SUN) cohort. British Journal of Nutrition 2019;122(5):542-51. doi: <http://dx.doi.org/10.1017/S0007114518003811>.

405. Mendonca RD, Carvalho NC, Martin-Moreno JM, Pimenta AM, Lopes ACS, Gea A, Martinez-Gonzalez MA, Bes-Rastrollo M. Total polyphenol intake, polyphenol subtypes and incidence of cardiovascular disease: The SUN cohort study. Nutr Metab Carbiovasc Dis 2019;29(1):69-78. doi: 10.1016/j.numecd.2018.09.012.

406. Romanos-Nanclares A, Sanchez-Quesada C, Gardeazabal I, Martinez-Gonzalez MA, Gea A, Toledo E. Phenolic Acid Subclasses, Individual Compounds, and Breast Cancer Risk in a Mediterranean Cohort: The SUN Project. Journal of the Academy of Nutrition and Dietetics 2020;22. doi: <http://dx.doi.org/10.1016/j.jand.2019.11.007>.

407. fengDai Q, Shu XO, Jin F, Potter JD, Kushi LH, Teas J, Gao YT, Zheng W. Population-based case-control study of soyfood intake and breast cancer risk in Shanghai. British Journal of Cancer 2001;85(3):372-8. doi: 10.1054/bjoc.2001.1873.

408. Baglia ML, Gu K, Zhang X, Zheng Y, Peng P, Cai H, Bao PP, Zheng W, Lu W, Shu XO. Soy isoflavone intake and bone mineral density in breast cancer survivors. Cancer Causes Control 2015;26(4):571-80. doi: <https://dx.doi.org/10.1007/s10552-015-0534-3>.

409. Lee SA, Shu XO, Li HL, Yang G, Cai H, Wen WQ, Ji BT, Gao J, Gao YT, Zheng W. Adolescent and adult soy food intake and breast cancer risk: results from the Shanghai Women's Health Study. American Journal of Clinical Nutrition 2009;89(6):1920-6. doi: 10.3945/ajcn.2008.27361.

410. Wu SH, Shu XO, Chow WH, Xiang YB, Zhang XL, Li HL, Cai QY, Ji BT, Cai H, Rothman N, et al. Soy Food Intake and Circulating Levels of Inflammatory Markers in Chinese Women. J Acad Nutr Diet 2012;112(7):996-1004. doi: 10.1016/j.jand.2012.04.001.

411. Wu X, Cai H, Gao YT, Dai Q, Li H, Cai Q, Yang G, Franke AA, Zheng W, Shu XO. Correlations of urinary phytoestrogen excretion with lifestyle factors and dietary intakes among middle-aged and elderly Chinese women. International Journal of Molecular Epidemiology and Genetics 2012;3(1):18-29.

412. Yang G, Shu XO, Li H, Chow WH, Cai H, Zhang X, Gao YT, Zheng W. Prospective cohort study of soy food intake and colorectal cancer risk in women. American Journal of Clinical Nutrition 2009;89(2):577-83. doi: <https://dx.doi.org/10.3945/ajcn.2008.26742>.

413. Yang G, Shu XO, Li HL, Chow WH, Wen W, Xiang YB, Zhang X, Cai H, Ji BT, Gao YT, et al. Prediagnosis soy food consumption and lung cancer survival in women. J Clin Oncol 2013;31(12):1548-53. doi: <https://dx.doi.org/10.1200/JCO.2012.43.0942>.

414. Yu DX, Shu XO, Li HL, Yang G, Cai QY, Xiang YB, Ji BT, Franke AA, Gao YT, Zheng W, et al. Dietary isoflavones, urinary isoflavonoids, and risk of ischemic stroke in women. American Journal of Clinical Nutrition 2015;102(3):680-6. doi: 10.3945/ajcn.115.111591.

415. Zhang X, Shu XO, Li H, Yang G, Li Q, Gao YT, Zheng W. Prospective cohort study of soy food consumption and risk of bone fracture among postmenopausal women. Arch Intern Med 2005;165(16):1890-5.

416. Clark ML, Butler LM, Koh WP, Wang R, Yuan JM. Dietary fiber intake modifies the association between secondhand smoke exposure and coronary heart disease mortality among Chinese non-smokers in Singapore. Nutrition 2013;29(11-12):1304-9. doi: <https://dx.doi.org/10.1016/j.nut.2013.04.003>.

417. Koh WP, Wu AH, Wang RW, Ang LW, Heng D, Yuan JM, Yu MC. Gender-specific Associations Between Soy and Risk of Hip Fracture in the Singapore Chinese Health Study. American Journal of Epidemiology 2009;170(7):901-9. doi: 10.1093/aje/kwp220.

418. Mueller NT, Odegaard AO, Gross MD, Koh WP, Yu MC, Yuan JM, Pereira MA. Soy intake and risk of type 2 diabetes mellitus in Chinese Singaporeans Soy intake and risk of type 2 diabetes. European Journal of Nutrition 2012;51(8):1033-40. doi: 10.1007/s00394-011-0276-2.

419. Paul P, Koh WP, Jin A, Michel A, Waterboer T, Pawlita M, Wang R, Yuan JM, Butler LM. Soy and tea intake on cervical cancer risk: the Singapore Chinese Health Study. Cancer Causes Control 2019;30(8):847-57. doi: <https://dx.doi.org/10.1007/s10552-019-01173-3>.

420. Sun CL, Yuan JM, Arakawa K, Low SH, Lee HP, Yu MC. Dietary soy and increased risk of bladder cancer: The Singapore Chinese health study. Cancer Epidemiol Biomarkers Prev 2002;11(12):1674-7.

421. Talaei M, Koh WP, van Dam RM, Yuan JM, Pan A. Dietary soy intake is not associated with risk of cardiovascular disease mortality in Singapore Chinese adults. Journal of Nutrition 2014;144(6):921-8. doi: <https://dx.doi.org/10.3945/jn.114.190454>.

422. Wu AH, Stanczyk FZ, Seow A, Lee HP, Yu MC. Soy intake and other lifestyle determinants of serum estrogen levels among postmenopausal chinese women in Singapore. Cancer Epidemiol Biomarkers Prev 2002;11(9):844-51.

423. Wu AH, Koh WP, Wang R, Lee HP, Yu MC. Soy intake and breast cancer risk in Singapore Chinese Health Study. British Journal of Cancer 2008;99(1):196-200. doi: <https://dx.doi.org/10.1038/sj.bjc.6604448>.

424. Goodman-Gruen D, Kritz-Silverstein D. Usual dietary isoflavone intake and body composition in postmenopausal women. Menopause-J N Am Menopause Soc 2003;10(5):427-32. doi: 10.1097/01.Gme.0000058866.35869.B4.

425. Kritz-Silverstein D, Goodman-Gruen DL. Usual dietary isoflavone intake, bone mineral density, and bone metabolism in postmenopausal women. J Womens Health Gender-Based Med 2002;11(1):69-78. doi: 10.1089/152460902753473480.

426. Kyle JAM, Sharp L, Little J, Duthie GG, McNeill G. Dietary flavonoid intake and colorectal cancer: A case-control study. British Journal of Nutrition 2010;103(3):429-36. doi: <http://dx.doi.org/10.1017/S0007114509991784>.

427. Theodoratou E, Kyle J, Cetnarskyj R, Farrington SM, Tenesa A, Barnetson R, Porteous M, Dunlop M, Campbell H. Dietary flavonoids and the risk of colorectal cancer. Cancer Epidemiol Biomarkers Prev 2007;16(4):684-93. doi: 10.1158/1055-9965.Epi-06-0785.

428. Petrick JL, Steck SE, Bradshaw PT, Chow WH, Engel LS, He K, Risch HA, Vaughan TL, Gammon MD. Dietary flavonoid intake and Barrett's esophagus in western Washington State. Ann Epidemiol 2015;25(10):730-5. doi: 10.1016/j.annepidem.2015.05.010.

429. Elaine Waetjen L, Leung K, Crawford SL, Huang MH, Gold EB, Greendale GA. Relationship between dietary phytoestrogens and development of urinary incontinence in midlife women. Menopause 2012;22. doi: <http://dx.doi.org/10.1097/gme.0b013e3182703c9c>.

430. Gold EB, Bair Y, Block G, Greendale GA, Harlow SD, Johnson S, Kravitz HM, Rasor MO, Siddiqui A, Sternfeld B, et al. Diet and lifestyle factors associated with premenstrual symptoms in a racially diverse community sample: Study of Women's Health Across the Nation (SWAN).[Erratum appears in J Womens Health (Larchmt). 2007 Jul-Aug;16(6):934]. J Womens Health (Larchmt) 2007;16(5):641-56.

431. Gold EB, Leung K, Crawford SL, Huang MH, Waetjen LE, Greendale GA. Phytoestrogen and fiber intakes in relation to incident vasomotor symptoms: results from the Study of Women's Health Across the Nation. Menopause 2013;20(3):305-14. doi: <https://dx.doi.org/10.1097/GME.0b013e31826d2f43>.

432. Greendale GA, FitzGerald G, Huang MH, Sternfeld B, Gold E, Seeman T, Sherman S, Sowers M. Dietary soy isoflavones and bone mineral density: Results from the Study of Women's Health Across the Nation. American Journal of Epidemiology 2002;155(8):746-54. doi: 10.1093/aje/155.8.746.

433. Greendale GA, Huang MH, Leung K, Crawford SL, Gold EB, Wight R, Waetjen E, Karlamangla AS. Dietary phytoestrogen intakes and cognitive function during the menopausal transition: results from the Study of Women's Health Across the Nation Phytoestrogen Study. Menopause-J N Am Menopause Soc 2012;19(8):894-903. doi: 10.1097/gme.0b013e318242a654.

434. Greendale GA, Tseng CH, Han W, Huang MH, Leung K, Crawford S, Gold EB, Waetjen LE, Karlamangla AS. Dietary isoflavones and bone mineral density during midlife and the menopausal transition: cross-sectional and longitudinal results from the Study of Women's Health Across the Nation Phytoestrogen Study. Menopause 2015;22(3):279-88. doi: <https://dx.doi.org/10.1097/GME.0000000000000305>.

435. Huang MH, Luetters C, Buckwalter GJ, Seeman TE, Gold EB, Sternfeld B, Greendale GA. Dietary genistein intake and cognitive performance in a multiethnic cohort of midlife women. Menopause-J N Am Menopause Soc 2006;13(4):621-30. doi: 10.1097/01.gme.0000227336.35620.8f.

436. Huang MH, Norris J, Han WJ, Block T, Gold E, Crawford S, Greendale GA. Development of an Updated Phytoestrogen Database for Use With the SWAN Food Frequency Questionnaire: Intakes and Food Sources in a Community-Based, Multiethnic Cohort Study. Nutr Cancer 2012;64(2):228-44. doi: 10.1080/01635581.2012.638434.

437. Sowers MR, Crawford S, McConnell DS, Randolph Jr JF, Gold EB, Wilkin MK, Lasley B. Selected diet and lifestyle factors are associated with estrogen metabolites in a multiracial/ethnic population of women. Journal of Nutrition 2006;136(6):1588-95.

438. Frankenfeld CL, Cerhan JR, Cozen W, Davis S, Schenk M, Morton LM, Hartge P, Ward MH. Dietary flavonoid intake and non-Hodgkin lymphoma risk. American Journal of Clinical Nutrition 2008;87(5):1439-45.

439. Le Marchand L, Murphy SP, Hankin JH, Wilkens LR, Kolonel LN. Intake of flavonoids and lung cancer. JNCI-J Natl Cancer Inst 2000;92(2):154-60. doi: 10.1093/jnci/92.2.154.

440. Konishi K, Wada K, Yamakawa M, Goto Y, Mizuta F, Koda S, Uji T, Tsuji M, Nagata C. Dietary Soy Intake Is Inversely Associated with Risk of Type 2 Diabetes in Japanese Women but Not in Men. Journal of Nutrition 2019;149(7):1208-14. doi: 10.1093/jn/nxz047.

441. Nagata C, Takatsuka N, Kawakami N, Shimizu H. Soy product intake and hot flashes in Japanese women: Results from a community-based prospective study. American Journal of Epidemiology 2001;153(8):790-3. doi: 10.1093/aje/153.8.790.

442. Nagata C, Takatsuka N, Kawakami N, Shimizu H. Soy product intake and premenopausal hysterectomy in a follow-up study of Japanese women. European Journal of Clinical Nutrition 2001;55(9):773-7. doi: 10.1038/sj.ejcn.1601223.

443. Nagata C, Nakamura K, Fujii K, Kawachi T, Takatsuka N, Oba S, Shimizu H. Soy isoflavone intake is not associated with the development of cedar pollinosis in adults. Journal of Nutrition 2008;138(7):1372-6.

444. Nagata C, Wada K, Tamura T, Konishi K, Goto Y, Koda S, Kawachi T, Tsuji M, Nakamura K. Dietary soy and natto intake and cardiovascular disease mortality in Japanese adults: the Takayama study. American Journal of Clinical Nutrition 2017;105(2):426-31. doi: 10.3945/ajcn.116.137281.

445. Oba S, Nagata C, Shimizu N, Shimizu H, Kametani M, Takeyama N, Ohnuma T, Matsushita S. Soy product consumption and the risk of colon cancer: A prospective study in Takayama, Japan. Nutr Cancer 2007;57(2):151-7. doi: 10.1080/01635580701274475.

446. Taguchi C, Kishimoto Y, Fukushima Y, Kondo K, Yamakawa M, Wada K, Nagata C. Dietary intake of total polyphenols and the risk of all-cause and specific-cause mortality in Japanese adults: the Takayama study. European Journal of Nutrition 2020;59(3):1263-71. doi: 10.1007/s00394-019-02136-9.

447. Wada K, Nakamura K, Tamai Y, Tsuji M, Kawachi T, Hori A, Takeyama N, Tanabashi S, Matsushita S, Tokimitsu N, et al. Soy isoflavone intake and breast cancer risk in Japan: From the Takayama study. International Journal of Cancer 2013;133(4):952-60. doi: 10.1002/ijc.28088.

448. Wada K, Tsuji M, Tamura T, Konishi K, Kawachi T, Hori A, Tanabashi S, Matsushita S, Tokimitsu N, Nagata C. Soy isoflavone intake and stomach cancer risk in Japan: From the Takayama study. International Journal of Cancer 2015;137(4):885-92. doi: 10.1002/ijc.29437.

449. Wada K, Tsuji M, Tamura T, Konishi K, Goto Y, Mizuta F, Koda S, Uji T, Hori A, Tanabashi S, et al. Soy isoflavone intake and bladder cancer risk in Japan: From the takayama study. Cancer Epidemiology Biomarkers and Prevention 2018;27(11):1371-5. doi: <http://dx.doi.org/10.1158/1055-9965.EPI-18-0283>.

450. Segovia-Siapco G, Pribis P, Oda K, Sabate J. Soy isoflavone consumption and age at pubarche in adolescent males. European Journal of Nutrition 2018;57(6):2287-94. doi: <https://dx.doi.org/10.1007/s00394-017-1504-1>.

451. Sohrab G, Hosseinpour-Niazi S, Hejazi J, Yuzbashian E, Mirmiran P, Azizi F. Dietary polyphenols and metabolic syndrome among Iranian adults. Int J Food Sci Nutr 2013;64(6):661-7. doi: 10.3109/09637486.2013.787397.

452. Sohrab G, Ebrahimof S, Hosseinpour-Niazi S, Yuzbashian E, Mirmiran P, Azizi F. The Association of Dietary Intakes of Total Polyphenol and Its Subclasses with the Risk of Metabolic Syndrome: Tehran Lipid and Glucose Study. Metab Syndr Relat Disord 2018;16(6):274-81. doi: 10.1089/met.2017.0140.

453. Horn-Ross PL, John EM, Lee M, Stewart SL, Koo J, Sakoda LC, Shiau AG, Goldstein J, Davis P, Perez-Stable EJ. Phytoestrogen consumption and breast cancer risk in a multiethnic population - The Bay Area Breast Cancer Study. American Journal of Epidemiology 2001;154(5):434-41. doi: 10.1093/aje/154.5.434.

454. Fuhrman BJ, Teter BE, Barba M, Byrne C, Cavalleri A, Grant BJ, Horvath PJ, Morelli D, Venturelli E, Muti PC. Equol status modifies the association of soy intake and mammographic density in a sample of postmenopausal women. Cancer Epidemiol Biomarkers Prev 2008;17(1):33-42. doi: 10.1158/1055-9965.Epi-07-0193.

455. Arts ICW, Jacobs Jr DR, Harnack LJ, Gross M, Folsom AR. Dietary catechins in relation to coronary heart disease death among postmenopausal women. Epidemiology 2001;12(6):668-75. doi: <http://dx.doi.org/10.1097/00001648-200111000-00015>.

456. Arts ICW, Jacobs Jr DR, Gross M, Harnack LJ, Folsom AR. Dietary catechins and cancer incidence among postmenopausal women: the Iowa Women's Health Study (United States). Cancer Causes Control 2002;13(4):373-82. doi: 10.1023/a:1015290131096.

457. Cutler GJ, Nettleton JA, Ross JA, Harnack LJ, Jacobs Jr DR, Scrafford CG, Barraj LM, Mink PJ, Robien K. Dietary flavonoid intake and risk of cancer in postmenopausal women: The Iowa Women's Health Study. International Journal of Cancer 2008;123(3):664-71. doi: <http://dx.doi.org/10.1002/ijc.23564>.

458. Mink PJ, Scrafford CG, Barraj LM, Harnack L, Hong CP, Nettleton JA, Jacobs DR, Jr. Flavonoid intake and cardiovascular disease mortality: a prospective study in postmenopausal women. American Journal of Clinical Nutrition 2007;85(3):895-909.

459. Thompson CA, Habermann TM, Wang AH, Vierkant RA, Folsom AR, Ross JA, Cerhan JR. Antioxidant intake from fruits, vegetables and other sources and risk of non-Hodgkin's lymphoma: the Iowa Women's Health Study. International Journal of Cancer 2010;126(4):992-1003. doi: 10.1002/ijc.24830.

460. Akhter M, Inoue M, Kurahashi N, Iwasaki M, Sasazuki S, Tsugane S, Japan Public Health Center-Based Prospective Study G. Dietary soy and isoflavone intake and risk of colorectal cancer in the Japan public health center-based prospective study. Cancer Epidemiol Biomarkers Prev 2008;17(8):2128-35. doi: <https://dx.doi.org/10.1158/1055-9965.EPI-08-0182>.

461. Budhathoki S, Iwasaki M, Sawada N, Yamaji T, Shimazu T, Sasazuki S, Inoue M, Tsugane S. Soy food and isoflavone intake and endometrial cancer risk: The Japan Public Health Center-based prospective study. BJOG: An International Journal of Obstetrics and Gynaecology 2015;122(3):304-11. doi: <http://dx.doi.org/10.1111/1471-0528.12853>.

462. Hara A, Sasazuki S, Inoue M, Iwasaki M, Shimazu T, Sawada N, Yamaji T, Tsugane S. Isoflavone intake and risk of gastric cancer: A population-based prospective cohort study in Japan. American Journal of Clinical Nutrition 2012;95(1):147-54. doi: <http://dx.doi.org/10.3945/ajcn.111.020479>.

463. Kokubo Y, Iso H, Ishihara J, Okada K, Inoue M, Tsugane S, Grp JS. Association of dietary intake of soy, beans, and isoflavones with risk of cerebral and myocardial infarctions in Japanese populations - The Japan Public Health Center-Based (JPHC) Study Cohort I. Circulation 2007;116(22):2553-62. doi: 10.1161/circulationaha.106.683755.

464. Kurahashi N, Iwasaki M, Sasazuki S, Otani T, Inoue M, Tsugane S. Soy product and isoflavone consumption in relation to prostate cancer in Japanese men. Cancer Epidemiology Biomarkers and Prevention 2007;16(3):538-45. doi: <http://dx.doi.org/10.1158/1055-9965.EPI-06-0517>.

465. Kurahashi N, Inoue M, Iwasaki M, Tanaka Y, Mizokami M, Tsugane S. Isoflavone consumption and subsequent risk of hepatocellular carcinoma in a population-based prospective cohort of Japanese men and women. International Journal of Cancer 2009;124(7):1644-9. doi: <http://dx.doi.org/10.1002/ijc.24121>.

466. Nanri A, Mizoue T, Takahashi Y, Kirii K, Inoue M, Noda M, Tsugane S. Soy product and isoflavone intakes are associated with a lower risk of type 2 diabetes in overweight Japanese women. Journal of Nutrition 2010;140(3):580-6. doi: <http://dx.doi.org/10.3945/jn.109.116020>.

467. Nozue M, Shimazu T, Sasazuki S, Charvat H, Mori N, Mutoh M, Sawada N, Iwasaki M, Yamaji T, Inoue M, et al. Fermented Soy Product Intake Is Inversely Associated with the Development of High Blood Pressure: The Japan Public Health Center-Based Prospective Study. Journal of Nutrition 2017;147(9):1749-56. doi: 10.3945/jn.117.250282.

468. Shimazu T, Inoue M, Sasazuki S, Iwasaki M, Sawada N, Yamaji T, Tsugane S. Isoflavone intake and risk of lung cancer: A prospective cohort study in Japan. American Journal of Clinical Nutrition 2010;91(3):722-8. doi: <http://dx.doi.org/10.3945/ajcn.2009.28161>.

469. Suzuki R, Iwasaki M, Inoue M, Sasazuki S, Sawada N, Yamaji T, Shimazu T, Tsugane S, Sobue T, Hanaoka T, et al. Alcohol consumption-associated breast cancer incidence and potential effect modifiers: The Japan Public Health Center-based prospective study. International Journal of Cancer 2010;127(3):685-95. doi: <http://dx.doi.org/10.1002/ijc.25079>.

470. Yamamoto S, Kobayashi M, Tsugane S, Sasaki S, Sobue T, Ogata J, Baba S, Miyakawa K, Saito F, Koizumi A, et al. Soy, isoflavones, and breast cancer risk in Japan. Journal of the National Cancer Institute 2003;95(12):906-13.

471. Rice MM, LaCroix AZ, Lampe JW, van Belle G, Kestin M, Sumitani M, Graves AB, Larson EB. Dietary soy isoflavone intake in older Japanese American women. Public Health Nutrition 2001;4(5):943-52. doi: 10.1079/phn2001150.

472. Zamora-Ros R, Biessy C, Rothwell JA, Monge A, Lajous M, Scalbert A, Lopez-Ridaura R, Romieu I. Dietary polyphenol intake and their major food sources in the Mexican Teachers' Cohort. British Journal of Nutrition 2018;120(3):353-60. doi: <http://dx.doi.org/10.1017/S0007114518001381>.

473. Tabak C, Arts IC, Smit HA, Heederik D, Kromhout D. Chronic obstructive pulmonary disease and intake of catechins, flavonols, and flavones: the MORGEN Study. Am J Respir Crit Care Med 2001;164(1):61-4.

474. Bandera EV, King M, Chandran U, Paddock LE, Rodriguez-Rodriguez L, Olson SH. Phytoestrogen consumption from foods and supplements and epithelial ovarian cancer risk: a population-based case control study. BMC Womens Health 2011;11:9. doi: 10.1186/1472-6874-11-40.

475. Zhang W, Wang J, Gao J, Li HL, Han LH, Lan Q, Rothman N, Zheng W, Shu XO, Xiang YB. Prediagnostic Level of Dietary and Urinary Isoflavonoids in Relation to Risk of Liver Cancer in Shanghai, China. Cancer Epidemiol Biomarkers Prev 2019;28(10):1712-9. doi: 10.1158/1055-9965.Epi-18-1075.

476. Suzuki R, Rylander-Rudqvist T, Saji S, Bergkvist L, Adlercreutz H, Wolk A. Dietary lignans and postmenopausal breast cancer risk by oestrogen receptor status: a prospective cohort study of Swedish women. British Journal of Cancer 2008;98(3):636-40. doi: 10.1038/sj.bjc.6604175.

477. Lin Y, Wolk A, Hakansson N, Lagergren J, Lu Y. Dietary intake of lignans and risk of esophageal and gastric adenocarcinoma: a cohort study in Sweden. Cancer Epidemiol Biomarkers Prev 2013;22(2):308-12. doi: <https://dx.doi.org/10.1158/1055-9965.EPI-12-1138>.

478. McCann SE, Hootman KC, Weaver AM, Thompson LU, Morrison C, Hwang H, Edge SB, Ambrosone CB, Horvath PJ, Kulkarni SA. Dietary Intakes of Total and Specific Lignans Are Associated with Clinical Breast Tumor Characteristics. Journal of Nutrition 2012;142(1):91-8. doi: 10.3945/jn.111.147264.

479. Williams ALM, Bonner M, Ochs-Balcom HM, Hwang H, Morrison C, McCann SE. Dietary lignan intake and androgen receptor expression in breast tumors. Cancer Causes and Control 2015;26(2):311-7. doi: <http://dx.doi.org/10.1007/s10552-014-0504-1>.

480. Yao Z, Gu Y, Zhang Q, Liu L, Meng G, Wu H, Xia Y, Bao X, Shi H, Sun S, et al. Estimated daily quercetin intake and association with the prevalence of type 2 diabetes mellitus in Chinese adults. European Journal of Nutrition 2019;58(2):819-30. doi: <https://dx.doi.org/10.1007/s00394-018-1713-2>.

481. Yao Z, Li C, Gu Y, Zhang Q, Liu L, Meng G, Wu H, Bao X, Zhang S, Sun S, et al. Dietary myricetin intake is inversely associated with the prevalence of type 2 diabetes mellitus in a Chinese population. Nutrition Research 2019;68:82-91. doi: <http://dx.doi.org/10.1016/j.nutres.2019.06.004>.

482. Vitale M, Vaccaro O, Masulli M, Bonora E, Del Prato S, Giorda CB, Nicolucci A, Squatrito S, Auciello S, Babini AC, et al. Polyphenol intake and cardiovascular risk factors in a population with type 2 diabetes: The TOSCA.IT study. Clin Nutr 2017;36(6):1686-92. doi: <https://dx.doi.org/10.1016/j.clnu.2016.11.002>.

483. Vitale M, Masulli M, Rivellese AA, Bonora E, Cappellini F, Nicolucci A, Squatrito S, Antenucci D, Barrea A, Bianchi C, et al. Dietary intake and major food sources of polyphenols in people with type 2 diabetes: The TOSCA.IT Study. European Journal of Nutrition 2018;57(2):679-88. doi: <https://dx.doi.org/10.1007/s00394-016-1355-1>.

484. Jennings A, Welch AA, Fairweather-Tait SJ, Kay C, Minihane AM, Chowienczyk P, Jiang BY, Cecelja M, Spector T, Macgregor A, et al. Higher anthocyanin intake is associated with lower arterial stiffness and central blood pressure in women. American Journal of Clinical Nutrition 2012;96(4):781-8. doi: 10.3945/ajcn.112.042036.

485. Jennings A, MacGregor A, Spector T, Cassidy A. Higher dietary flavonoid intakes are associated with lower objectively measured body composition in women: evidence from discordant monozygotic twins. American Journal of Clinical Nutrition 2017;105(3):626-34. doi: 10.3945/ajcn.116.144394.

486. Welch A, MacGregor A, Jennings A, Fairweather-Tait S, Spector T, Cassidy A. Habitual flavonoid intakes are positively associated with bone mineral density in women. J Bone Miner Res 2012;27(9):1872-8. doi: <https://dx.doi.org/10.1002/jbmr.1649>.

487. Noorwali E, Hardie L, Cade J. Fruit and Vegetable Consumption and Their Polyphenol Content Are Inversely Associated with Sleep Duration: Prospective Associations from the UK Women's Cohort Study. Nutrients 2018;10(11):18. doi: 10.3390/nu10111803.

488. Petrick JL, Steck SE, Bradshaw PT, Trivers KF, Abrahamson PE, Engel LS, He K, Chow WH, Mayne ST, Risch HA, et al. Dietary intake of flavonoids and oesophageal and gastric cancer: incidence and survival in the United States of America (USA). British Journal of Cancer 2015;112(7):1291-300. doi: 10.1038/bjc.2015.25.

489. McCann SE, Muti P, Vito D, Edge SB, Trevisan M, Freudenheim JL. Dietary Lignan intakes and risk of pre- and postmenopausal breast cancer. International Journal of Cancer 2004;111(3):440-3. doi: 10.1002/jc.20262.

490. McCann SE, Kulkarni S, Trevisan M, Vito D, Nie J, Edge SB, Muti P, Freudenheim JL. Dietary lignan intakes and risk of breast cancer by tumor estrogen receptor status. Breast Cancer Res Treat 2006;99(3):309-11. doi: 10.1007/s10549-006-9196-x.

491. McCann SE, Thompson LU, Nie J, Dorn J, Trevisan M, Shields PG, Ambrosone CB, Edge SB, Li HF, Kasprzak C, et al. Dietary lignan intakes in relation to survival among women with breast cancer: the Western New York Exposures and Breast Cancer (WEB) Study. Breast Cancer Res Treat 2010;122(1):229-35. doi: 10.1007/s10549-009-0681-x.

492. McCann SE, Freudenheim JL, Marshall JR, Graham S. Risk of human ovarian cancer is related to dietary intake of selected nutrients, phytochemicals and food groups. Journal of Nutrition 2003;133(6):1937-42.

493. McCann SE, Ambrosone CB, Moysich KB, Brasure J, Marshall JR, Freudenheim JL, Wilkinson GS, Graham S. Intakes of selected nutrients, foods, and phytochemicals and prostate cancer risk in western New York. Nutr Cancer 2005;53(1):33-41. doi: 10.1207/s15327914nc5301_4.

494. Sesso HD, Gaziano JM, Liu S, Buring JE. Flavonoid intake and the risk of cardiovascular disease in women. American Journal of Clinical Nutrition 2003;77(6):1400-8.

495. Song Y, Manson JE, Buring JE, Sesso HD, Liu S. Associations of dietary flavonoids with risk of type 2 diabetes, and markers of insulin resistance and systemic inflammation in women: a prospective study and cross-sectional analysis. Journal of the American College of Nutrition 2005;24(5):376-84.

496. Wang L, Lee IM, Zhang SM, Blumberg JB, Buring JE, Sesso HD. Dietary intake of selected flavonols, flavones, and flavonoid-rich foods and risk of cancer in middle-aged and older women. American Journal of Clinical Nutrition 2009;89(3):905-12. doi: 10.3945/ajcn.2008.26913.

497. Caan BJ, Natarajan L, Parker B, Gold EB, Thomson C, Newman V, Rock CL, Pu MY, Al-Delaimy W, Pierce JP. Soy Food Consumption and Breast Cancer Prognosis. Cancer Epidemiol Biomarkers Prev 2011;20(5):854-8. doi: 10.1158/1055-9965.Epi-10-1041.

498. Hedelin M, Lof M, Olsson M, Adlercreutz H, Sandin S, Weiderpass E. Dietary phytoestrogens are not associated with risk of overall breast cancer but diets rich in coumestrol are inversely associated with risk of estrogen receptor and progesterone receptor negative breast tumors in Swedish women. Journal of Nutrition 2008;138(5):938-45.

499. Hedelin M, Lo M, Andersson TML, Adlercreutz H, Weiderpass E. Dietary phytoestrogens and the risk of ovarian cancer in the women's lifestyle and health cohort study. Cancer Epidemiology Biomarkers and Prevention 2011;20(2):308-17. doi: <http://dx.doi.org/10.1158/1055-9965.EPI-10-0752>.

500. Hedelin M, Lof M, Sandin S, Adami HO, Weiderpass E. Prospective Study of Dietary Phytoestrogen Intake and the Risk of Colorectal Cancer. Nutr Cancer 2016;68(3):388-95. doi: 10.1080/01635581.2016.1152380.

501. Frankenfeld CL, Patterson RE, Horner NK, Neuhouser ML, Skor HE, Kalhorn TF, Howald WN, Lampe JW. Validation of a soy food-frequency questionnaire and evaluation of correlates of plasma isoflavone concentrations in postmenopausal women. American Journal of Clinical Nutrition 2003;77(3):674-80.

502. Boucher BA, Wanigaratne S, Harris SA, Cotterchio M. Postdiagnosis Isoflavone and Lignan Intake in Newly Diagnosed Breast Cancer Patients: Cross-Sectional Survey Shows Considerable Intake from Previously Unassessed High-Lignan Foods. Curr Dev Nutr 2018;2(3):7. doi: 10.3945/cdn.117.002063.

503. Christensen KY, Naidu A, Parent ME, Pintos J, Abrahamowicz M, Siemiatycki J, Koushik A. The Risk of Lung Cancer Related to Dietary Intake of Flavonoids. Nutr Cancer 2012;64(7):964-74. doi: 10.1080/01635581.2012.717677.

504. Garcia-Larsen V, Amigo H, Bustos P, Bakolis I, Rona RJ. Ventilatory function in young adults and dietary antioxidant intake. Nutrients 2015;7(4):2879-96. doi: <https://dx.doi.org/10.3390/nu7042879>.

505. Guthrie JR, Ball M, Murkies A, Dennerstein L. Dietary phytoestrogen intake in mid-life Australian-born women: relationship to health variables. Climacteric 2000;3(4):254-61.

506. Lagiou P, Samoli E, Lagiou A, Katsouyanni K, Peterson J, Dwyer J, Trichopoulos D. Flavonoid intake in relation to lung cancer risk: A case-control study among women in Greece. Nutr Cancer 2004;49(2):139-43. doi: 10.1207/s15327914nc4902_4.

507. Lagiou P, Samoli E, Lagiou A, Peterson J, Tzonou A, Dwyer J, Trichopoulos D. Flavonoids, vitamin C and adenocarcinoma of the stomach. Cancer Causes Control 2004;15(1):67-72. doi: 10.1023/B:CACO.0000016619.18041.b0.

508. Lagiou P, Samoli E, Lagiou A, Tzonou A, Kalandidi A, Peterson J, Dwyer J, Trichopoulos D. Intake of specific flavonoid classes and coronary heart disease - a case-control study in Greece. European Journal of Clinical Nutrition 2004;58(12):1643-8. doi: 10.1038/sj.ejcn.1602022.

509. Lagiou P, Samoli E, Lagiou A, Skalkidis Y, Katsouyanni K, Petridou E, Trichopoulos D. Flavonoid classes and risk of peripheral arterial occlusive disease: a case-control study in Greece. European Journal of Clinical Nutrition 2006;60(2):214-9. doi: 10.1038/sj.ejcn.1602291.

510. Lagiou P, Rossi M, Lagiou A, Tzonou A, La Vecchia C, Trichopoulos D. Flavonoid intake and liver cancer: a case-control study in Greece. Cancer Causes Control 2008;19(8):813-8. doi: 10.1007/s10552-008-9144-7.

511. Linseisen J, Piller R, Hermann S, Chang-Claude J. Dietary phytoestrogen intake and premenopausal breast cancer risk in a German case-control study. International Journal of Cancer 2004;110(2):284-90. doi: 10.1002/ijc.20119.

512. Murphy KJ, Walker KM, Dyer KA, Bryan J. Estimation of daily intake of flavonoids and major food sources in middle-aged Australian men and women. Nutrition Research 2019;61:64-81. doi: <http://dx.doi.org/10.1016/j.nutres.2018.10.006>.

513. Mykoniatis I, Grammatikopoulou MG, Bouras E, Karampasi E, Tsionga A, Kogias A, Vakalopoulos I, Haidich AB, Chourdakis M. Sexual Dysfunction Among Young Men: Overview of Dietary Components Associated With Erectile Dysfunction. J Sex Med 2018;15(2):176-82. doi: 10.1016/j.jsxm.2017.12.008.

514. Peterson J, Lagiou P, Samoli E, Lagiou A, Katsouyanni K, La Vecchia C, Dwyer J, Trichopoulos D. Flavonoid intake and breast cancer risk: a case-control study in Greece. British Journal of Cancer 2003;89(7):1255-9. doi: 10.1038/sj.bjc.6601271.

515. Praud D, Parpinel M, Guercio V, Bosetti C, Serraino D, Facchini G, Montella M, La Vecchia C, Rossi M. Proanthocyanidins and the risk of prostate cancer in Italy. Cancer Causes Control 2018;29(2):261-8. doi: 10.1007/s10552-018-1002-7.

516. Rossi M, Bosetti C, Negri E, Lagiou P, La Vecchia C. Flavonoids, Proanthocyanidins, and Cancer Risk: A Network of Case-Control Studies From Italy. Nutr Cancer 2010;62(7):871-7. doi: 10.1080/01635581.2010.509534.

517. Veldink JH, Kalmijn S, Groeneveld GJ, Wunderink W, Koster A, de Vries JHM, van der Luyt J, Wokke JHJ, Van den Berg LH. Intake of polyunsaturated fatty acids and vitamin E reduces the risk of developing amyotrophic lateral sclerosis. J Neurol Neurosurg Psychiatry 2007;78(4):367-71. doi: 10.1136/jnnp.2005.083378.

518. Youseflu S, Sadatmahalleh SJ, Mottaghi A, Kazemnejad A. Dietary phytoestrogen intake and the risk of endometriosis in iranian women: A case-control study. International Journal of Fertility and Sterility 2020;13(4):296-300. doi: <http://dx.doi.org/10.22074/ijfs.2020.5806>.

519. Yahya HM, Day A, Lawton C, Myrissa K, Croden F, Dye L, Williamson G. Dietary intake of 20 polyphenol subclasses in a cohort of UK women. European Journal of Nutrition 2016;55(5):1839-47. doi: <https://dx.doi.org/10.1007/s00394-015-1001-3>.

520. Hertog MG, Kromhout D, Aravanis C, Blackburn H, Buzina R, Fidanza F, Giampaoli S, Jansen A, Menotti A, Nedeljkovic S, et al. Flavonoid intake and long-term risk of coronary heart disease and cancer in the seven countries study.[Erratum appears in Arch Intern Med 1995 Jun 12;155(11):1184]. Arch Intern Med 1995;155(4):381-6.

521. Lin J, Zhang SMM, Wu KN, Willett WC, Fuchs CS, Giovannucci E. Flavonoid intake and colorectal cancer risk in men and women. American Journal of Epidemiology 2006;164(7):644-51. doi: 10.1093/aje/kwj296.

522. Carrion-Garcia CJ, Guerra-Hernandez EJ, Garcia-Villanova B, Molina-Montes E. Non-enzymatic antioxidant capacity (NEAC) estimated by two different dietary assessment methods and its relationship with NEAC plasma levels. European Journal of Nutrition 2017;56(4):1561-76. doi: <https://dx.doi.org/10.1007/s00394-016-1201-5>.

523. Chavez-Suarez KM, Ortega-Velez MI, Valenzuela-Quintanar AI, Galvan-Portillo M, Lopez-Carrillo L, Esparza-Romero J, Saucedo-Tamayo MS, Robles-Burgueno MR, Palma-Duran SA, Gutierrez-Coronado ML, et al. Phytoestrogen Concentrations in Human Urine as Biomarkers for Dietary Phytoestrogen Intake in Mexican Women. Nutrients 2017;9(10):14. doi: 10.3390/nu9101078.

524. Bhakta D, dos Santos Silva I, Higgins C, Sevak L, Kassam-Khamis T, Mangtani P, Adlercreutz H, McMichael A. A semiquantitative food frequency questionnaire is a valid indicator of the usual intake of phytoestrogens by south Asian women in the UK relative to multiple 24-h dietary recalls and multiple plasma samples. Journal of Nutrition 2005;135(1):116-23.

525. Maskarinec G, Singh S, Meng LX, Franke AA. Dietary soy intake and urinary isoflavone excretion among women from a multiethnic population. Cancer Epidemiol Biomarkers Prev 1998;7(7):613-9.

526. Horn-Ross PL, Barnes S, Lee VS, Collins CN, Reynolds P, Lee MM, Stewart SL, Canchola AJ, Wilson L, Jones K. Reliability and validity of an assessment of usual phytoestrogen consumption (United States). Cancer Causes Control 2006;17(1):85-93. doi: 10.1007/s10552-005-0391-6.

527. Goni I, Hernandez-Galiot A. Intake of Nutrient and Non-Nutrient Dietary Antioxidants. Contribution of Macromolecular Antioxidant Polyphenols in an Elderly Mediterranean Population. Nutrients 2019;11(9):10. doi: <https://dx.doi.org/10.3390/nu11092165>.

528. Nishimuro H, Ohnishi H, Sato M, Ohnishi-Kameyama M, Matsunaga I, Naito S, Ippoushi K, Oike H, Nagata T, Akasaka H, et al. Estimated daily intake and seasonal food sources of quercetin in Japan. Nutrients 2015;7(4):2345-58. doi: <http://dx.doi.org/10.3390/nu7042345>.

529. Hoge A, Guillaume M, Albert A, Tabart J, Dardenne N, Donneau AF, Kevers C, Defraigne JO, Pincemail J. Validation of a food frequency questionnaire assessing dietary polyphenol exposure using the method of triads. Free Radic Biol Med 2019;130:189-95. doi: 10.1016/j.freeradbiomed.2018.11.001.

530. Wilson RT, Wang J, Chinchilli V, Richie JP, Virtamo J, Moore LE, Albanes D. Fish, vitamin D, and flavonoids in relation to renal cell cancer among smokers. American Journal of Epidemiology 2009;170(6):717-29. doi: <https://dx.doi.org/10.1093/aje/kwp178>.

531. Somerset S, Papier K. A Food Frequency Questionnaire Validated for Estimating Dietary Flavonoid Intake in an Australian Population. Nutr Cancer 2014;66(7):1200-10. doi: 10.1080/01635581.2014.951728.

532. Huang MH, Harrison GG, Mohamed MM, Gornbein JA, Henning SM, Go VLW, Greendale GA. Assessing the accuracy of a food frequency questionnaire for estimating usual intake of phytoestrogens. Nutr Cancer 2000;37(2):145-54. doi: 10.1207/s15327914nc372_5.

533. French MR, Thompson LU, Hawker GA. Validation of a phytoestrogen food frequency questionnaire with urinary concentrations of isoflavones and lignan metabolites in premenopausal women. Journal of the American College of Nutrition 2007;26(1):76-82. doi: 10.1080/07315724.2007.10719588.

534. Ishihara J, Iwasaki M, Kunieda CM, Hamada GS, Tsugane S. Food frequency questionnaire is a valid tool in the nutritional assessment of Brazilian women of diverse ethnicity. Asia Pacific Journal of Clinical Nutrition 2009;18(1):76-80.

535. Hakim IA, Hartz V, Harris RB, Balentine D, Weisgerber UM, Graver E, Whitacre R, Alberts D. Reproducibility and relative validity of a questionnaire to assess intake of black tea polyphenols in epidemiological studies. Cancer Epidemiol Biomarkers Prev 2001;10(6):667-78.

536. Cao J, Chen W, Yang J, Hao D, Zhang Y, Chang P, Zhao X. Reproducibility and relative validity of a food frequency questionnaire to assess intake of dietary flavonol and flavone in Chinese university campus population. Nutrition Research 2010;30(8):520-6. doi: <http://dx.doi.org/10.1016/j.nutres.2010.07.001>.

537. Bingham S, Luben R, Welch A, Low YL, Khaw KT, Wareham N, Day N. Associations between dietary methods and biomarkers, and between fruits and vegetables and risk of ischaemic heart disease, in the EPIC Norfolk Cohort Study. Int J Epidemiol 2008;37(5):978-87. doi: <https://dx.doi.org/10.1093/ije/dyn111>.

538. Yamamoto S, Sobue T, Sasaki S, Kobayashi M, Arai Y, Uehara M, Adlercreutz H, Watanabe S, Takahashi T, Iitoi Y, et al. Validity and reproducibility of a self-administered food-frequency questionnaire to assess isoflavone intake in a Japanese population in comparison with dietary records and blood and urine isoflavones. Journal of Nutrition 2001;131(10):2741-7.

539. Yang M, Wang Y, Davis CG, Lee SG, Fernandez ML, Koo SI, Cho E, Chun OK. Validation of an FFQ to assess antioxidant intake in overweight postmenopausal women. Public Health Nutrition 2014;17(7):1467-75. doi: 10.1017/s1368980013001638.

540. Verkasalo PK, Appleby PN, Allen NE, Davey G, Adlercreutz H, Key TJ. Soya intake and plasma concentrations of daidzein and genistein: validity of dietary assessment among eighty British women (Oxford arm of the European Prospective Investigation into Cancer and Nutrition). British Journal of Nutrition 2001;86(3):415-21. doi: 10.1079/bjn2001424.

541. Kreijkamp-Kaspers S, Kok L, Bots ML, Grobbee DE, van der Schouw YT. Dietary phytoestrogens and plasma lipids in Dutch postmenopausal women; a cross-sectional study. Atherosclerosis 2005;178(1):95-100. doi: 10.1016/j.atherosclerosis.2004.06.002.

542. Vian I, Zielinsky P, Zilio AM, Mello A, Lazzeri B, Oliveira A, Lampert KV, Piccoli A, Nicoloso LH, Bubols GB, et al. Development and validation of a food frequency questionnaire for consumption of polyphenol-rich foods in pregnant women. Matern Child Nutr 2015;11(4):511-24. doi: 10.1111/mcn.12025.

543. Kalmijn S, Feskens EJ, Launer LJ, Kromhout D. Polyunsaturated fatty acids, antioxidants, and cognitive function in very old men. American Journal of Epidemiology 1997;145(1):33-41.

544. Keli SO, Hertog MGL, Feskens EJM, Kromhout D. Dietary flavonoids, antioxidant vitamins, and incidence of stroke: The Zutphen study. Arch Intern Med 1996;156(6):637-42. doi: <http://dx.doi.org/10.1001/archinte.156.6.637>.

545. Dower JI, Geleijnse JM, Hollman PCH, Soedamah-Muthu SS, Kromhout D. Dietary epicatechin intake and 25-y risk of cardiovascular mortality: The Zutphen Elderly Study. American Journal of Clinical Nutrition 2016;104(1):58-64. doi: <http://dx.doi.org/10.3945/ajcn.115.128819>.

546. Arts IC, Hollman PC, Bueno De Mesquita HB, Feskens EJ, Kromhout D. Dietary catechins and epithelial cancer incidence: the Zutphen elderly study. International Journal of Cancer 2001;92(2):298-302.

547. Milder IEJ, Feskens EJM, Arts ICW, Bueno-de-Mesquita HB, Hollman PCH, Kromhout D. Intakes of 4 dietary lignans and cause-specific and all-cause mortality in the Zutphen Elderly Study. American Journal of Clinical Nutrition 2006;84(2):400-5.

548. Ruidavets J, Teissedre P, Ferrieres J, Carando S, Bougard G, Cabanis J. Catechin in the Mediterranean diet: vegetable, fruit or wine? Atherosclerosis 2000;153(1):107-17.

549. Brat P, George S, Bellamy A, Du Chaffaut L, Scalbert A, Mennen L, Arnault N, Amiot MJ. Daily polyphenol intake in France from fruit and vegetables. J Nutr 2006;136(9):2368-73. doi: 10.1093/jn/136.9.2368.
